# Photoswitchable Ambiphilicity: Light‐Gated Frustrated Lewis Pair Reactivity and Z‐Type Interaction

**DOI:** 10.1002/anie.5038244

**Published:** 2026-09-24

**Authors:** Lennart Stoess, Emil L. M. Paulin, Fabian Michel, Lutz Greb

**Affiliations:** ^1^ Anorganisch‐Chemisches Institut Ruprechts‐Karls Universität Heidelberg Heidelberg Germany

## Abstract

Ambiphilic molecules, species containing both Lewis acidic and basic sites, enable a broad range of phenomena in chemistry, from frustrated Lewis pairs (FLPs) to supported Z‐type interactions. However, the balance between their Lewis acidic and basic functions is normally fixed by molecular synthesis. Combining these features with photoswitchable architectures offers untapped potential for reversible substrate binding and control of transition metal properties. Here, we demonstrate that the incorporation of a photoswitchable Lewis acid into intramolecular phosphorus/boron Lewis pairs allows for stimuli‐responsive modulation of ambiphilicity. In the context of FLP‐type reactivity, both thermodynamic and kinetic control over substrate activation is achieved, culminating in the light‐gated binding and release of CO_2_. Implementing these ambiphiles as ligands in transition metal chemistry enables control over metal‐boron Z‐type interactions. Photoswitchable ambiphilicity thus provides a common light‐addressable handle for controlling substrate‐binding thermodynamics, reaction kinetics, and transition‐metal electronic structure.

## Graphical abstract

Light‐controlled ambiphilicity is realized in photoswitchable intramolecular phosphorus/boron Lewis pairs. Reversible switching modulates both the thermodynamics and kinetics of frustrated Lewis‐pair reactivity, enabling light‐gated CO_2_ binding and release. Coordination to transition metals further regulates metal–boron Z‐type interactions, establishing photoswitchable ambiphilicity as a general tool for controlling molecular function.

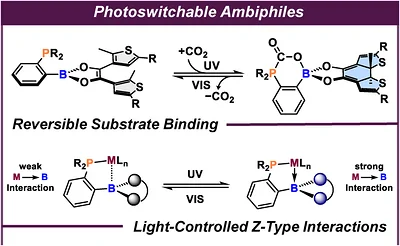

## 1 Introduction

Ambiphilic molecules, which combine Lewis acidic and Lewis basic functionalities within a single framework, have unlocked a broad range of concepts in chemistry, including frustrated Lewis pairs (FLP) [1], element‐ligand cooperativity [2], and Z‐type interactions [1, 2, 3, 4]. Intramolecular FLPs have been extensively studied for the activation of small molecules [5]. Prominent examples include the heterolytic cleavage of H_2_ [6, 7, 8, 9], the binding and transformation of CO_2_ [10, 11, 12, 13], or the activation of olefins (Figure 1a) [14, 15]. In many cases, a sufficient threshold of Lewis acidity is required to enable substrate activation [16]. At the same time, this high acceptor strength can limit reversibility under mild conditions, which constrains the development of systems in which substrate binding and release can be dynamically controlled. For example, CO_2_ binding to classical intramolecular FLPs has been shown to be reversible only under varying temperature, pressure, or solvent polarity (Figure 1a) [10].

**FIGURE 1:**
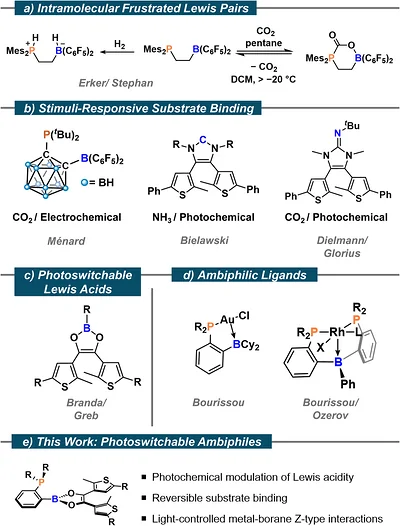
(a) Activation of H_2_ and CO_2_ by intramolecular frustrated Lewis pairs. (b) Modulation of substrate binding equilibria in response to external stimuli. (c) Photoswitchable Lewis acids. (d) Ambiphiles as Z‐type ligands in transition metal complexes. (e) Photoswitchable phosphinoborane presented in this work.

One strategy to address this challenge is the incorporation of switchable elements that enable modulation of Lewis acidity or basicity in response to an external stimulus. In the context of CO_2_ binding, such approaches have demonstrated reversible capture and release, for example in *ortho*‐carborane‐appended FLPs using an electrochemical stimulus (Figure 1b) [17]. Among the possible stimuli, light is especially attractive, as it allows for non‐invasive control without the need for chemical additives. In this regard, photoswitchable systems have been developed in which substrate uptake and release can be reversibly controlled via irradiation at specific wavelengths. Examples include hydrogen [18, 19, 20] or halogen [21, 22] bond donors for anion binding, and photoswitchable supramolecular assemblies for reversible host‐guest binding [23, 24]. Switchable Lewis bases are also well established: Photoswitchable carbenes are capable of reversible ammonia activation [25], and N‐heterocyclic imine superbases can reversibly capture CO_2_ (Figure 1b) [26]. The field of photoswitchable Lewis acids is less developed. Previous work by *Branda* and, more recently, our group has demonstrated that boron‐functionalized dithienylethenes can exhibit substantial changes in Lewis acidity between photochemical states, providing a suitable platform for light‐controlled catalysis (Figure 1c) [27, 28]. However, although computational studies have proposed photoswitchable FLPs for light‐controlled H_2_ activation and release [29, 30], an experimental molecular platform that reversibly switches FLP substrate binding has remained elusive.

Such a system would provide a non‐invasive means of controlling substrate binding and release under otherwise unchanged reaction conditions. Beyond their use as FLPs, ambiphilic molecules have also been explored as ligands with great success, for example with Au(I) [31] or Rh(I) [32, 33, 34] metal centers (Figure 1d). Complexes with ambiphilic ligands have enabled unprecedented metal electronic structures, such as Rh(‐I) species [35, 36], and novel reactivity, for example in Lewis acid assisted reductive elimination reactions [37, 38, 39]. Again, the exploration of photoswitchable units in such species has not been followed. Building on our photoswitchable boron‐based Lewis acid, we sought the combination with a vicinally linked Lewis base to realize ambiphilic systems with light‐responsive reactivity. Herein, we report the design of phosphine‐tethered photoswitchable boronates, introducing light‐controlled ambiphilicity to enable reversible FLP chemistry and Z‐Type interactions (Figure 1e).

## 2 Results and Discussion

### 2.1 Synthesis

Reaction of **L^1^** with *ortho*‐phosphino boronates **A** or **B** [40] at room temperature in benzene under azeotropic removal of methanol resulted in the formation of the open‐form compounds **1‐o** and **2‐o** (Figure 2). These were isolated as off‐white solids in good yield after recrystallization from *n*‐hexane (**1‐o**) or DCM/*n*‐hexane (**2‐o)** at −40°C. ^31^P and ^11^B NMR shifts suggested the absence of a phosphorus‐boron interaction in solution (Figure 2). The solid‐state structure of **2‐o** confirms the trigonal‐planar coordination environment of the boron center. No classical P···B interaction is observed in the solid state (P‐B distance: 3.304 Å), with the linking phenyl ring being coplanar to the dioxaborole unit. The dithienylethene ligand adopts a non‐photoactive parallel arrangement. For **1‐o**, a similar conformation is observed in the solid state (ESI Section S5), but insufficient data quality precludes detailed discussions of structural parameters.

**FIGURE 2:**
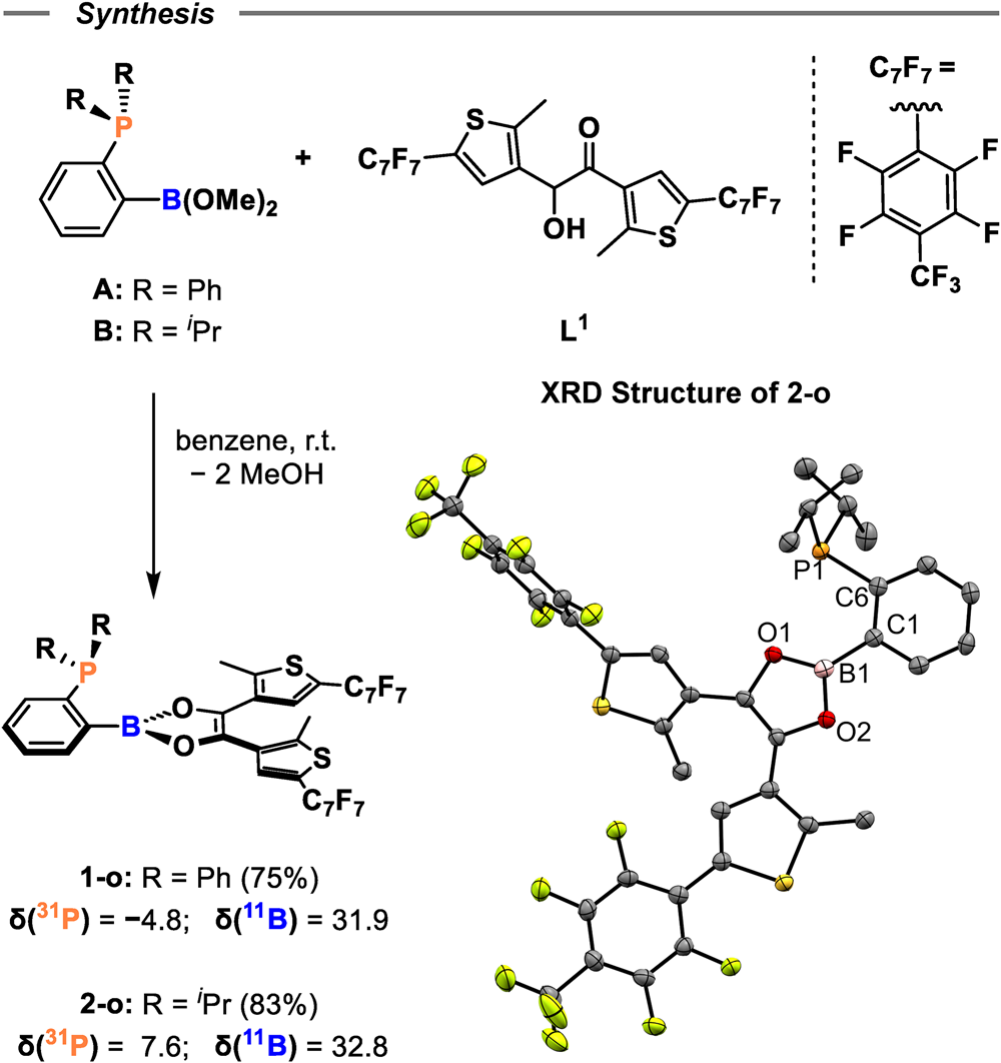
Synthesis of photoswitchable *ortho*‐phosphinoboranes **1‐o** and **2‐o**. Solid state structure of **2‐o**. Hydrogen atoms and cocrystallized DCM are omitted for clarity. Thermal ellipsoids are displayed at the 50% probability level. Selected bond lengths [pm], angles [°], and torsions [°]: P1‐C6: 185.0(2), B1‐C1: 155.6(4), B1‐O1: 138.1(3), O1‐B1‐O2: 109.5(2), O1‐B1‐C1: 130.8(2), O1‐B1‐C1‐C6: −1.6(4).

### 2.2 Photochemistry

The photochemical properties of **1** and **2** were evaluated by UV‐vis spectroscopy. In the open forms, **1‐o** and **2‐o** form colorless solutions in DCM with an absorption maximum at 290 (**1‐o**) and 294 (**2‐o**) nm. Upon irradiation at 300 nm, the compounds were converted to their closed isomers **1‐c** and **2‐c**, along with a color change to dark blue, as expected for the formation of an extended *π*‐conjugated system (Figure 3a). The absorption maximum of **2‐c** (575 nm) is slightly redshifted compared to **1‐c** (572 nm). In DCM, pronounced differences in the photocyclization quantum yields were observed, with **2** exhibiting a significantly lower value (Φ = 0.23 ± 0.05) compared to **1** (Φ = 0.71 ± 0.08). Differences in the equilibria between photoactive and non‐photoactive open‐form conformations in solution may contribute to this behavior. In addition, considering that the primary difference between **1** and **2** is the basicity of the phosphine moiety, the reduced quantum yield of **2** may indicate the presence of a competing, non‐productive P→B charge transfer, which is a well‐described phenomenon for FLPs [41, 42]. When probing photocyclization quantum yields in less polar benzene, similar values were observed for both compounds (Φ = 0.75 ± 0.08 for **1** and Φ = 0.84 ± 0.09 for **2**). The pronounced solvent dependence observed for **2** is consistent with the involvement of a polarity‐stabilized charge‐transfer deactivation pathway. However, time‐dependent DFT calculations show that the lowest‐energy singlet excitation is localized on the dithienylethene core for both compounds, with no direct involvement of phosphorus‐centered orbitals (HOMO‐1) in the S1 state (Figure 3b). This indicates that such effects, strongly depending on conformation, cannot be captured by this static computational approximation. Irradiation with broadband visible light for 60 s induced cycloreversion, restoring the initial UV–vis spectra. The fatigue behavior was assessed by repeated irradiation at 300 nm and with visible light. Both **1** and **2** exhibit high fatigue resistance, with a decrease in signal intensity of only 9% and 5% for **1** and **2**, respectively, over 10 cycles (Figure 3c). The photocyclization was assessed additionally by NMR spectroscopy. In C_6_D_6_, conversions of 94% to the respective closed forms were observed for both **1** and **2** after irradiation at 300 nm. In DCM, the lower cyclization quantum yield of **2** leads to an 82% conversion to the closed form, compared to 92% for **1** (Figure 3d for **2** in DCM, Sections S4.1 and S4.2 for further details). The changes of the ^31^P NMR resonances are small (1‐2 ppm), indicating no significant changes in phosphorus‐boron interactions after photocyclization. The thermal stability of the closed form was assessed by storing C_6_D_6_ solutions of **1‐c** and **2‐c** in the dark overnight, which showed no thermal ring‐opening (Section S4.3).

**FIGURE 3:**
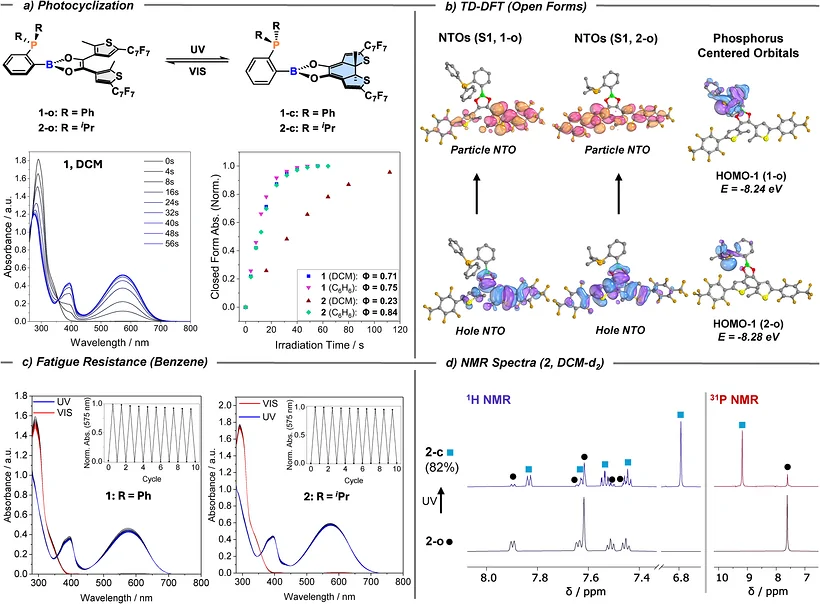
(a) Photocyclization of compounds **1** and **2**. Representative UV‐vis spectra of **1** (DCM, 3.1 · 10^−5^ M) during irradiation with 300 nm light. Normalized Absorbance of the closed isomers with irradiation time. (b) Natural transition orbitals of the S1 transition of **1‐o** and **2‐o** and phosphorus centered HOMO‐1 of **1‐o** and **2‐o**. Method: ωB97X‐D3/def2‐TZVPP/SMD(DCM). Structures were optimized in the photoactive antiparallel conformation (PBEh‐3c). (c) UV‐vis spectra of compounds **1** (left, 2.7 · 10^−5^ M, benzene) and **2** (right, 3.8 · 10^−5^ M, benzene) during consecutive switching cycles. UV irradiation: 300 nm, 60 s (**1**). VIS irradiation: broadband, 60 s. Inset: Changes in the normalized closed form absorbance during switching cycles. (d) ^1^H (600 MHz, 298K) and ^31^P (243 MHz, 298K) NMR spectral changes upon conversion of **2‐o** to **2‐c** (CD_2_Cl_2_).

### 2.3 Modulation of Ambiphilicity

Having established their photochemical properties, the ambiphilic character of both photoisomers was investigated by computational methods (DSD‐BLYP/def2‐QZVPP//PBEh‐3c). The Lewis acidity was assessed by the gas phase fluoride ion affinities (FIAs) of the open (FIA(o)) and closed (FIA(c)) forms. The changes in basicity of the phosphorus center were gauged by changes in proton affinity (PA) between open (PA(o)) and closed (PA(c)) forms (Figure 4). The photoisomerization‐induced FIA changes are pronounced, with differences of 76 and 90 kJ mol^−1^ for **1** and **2**, respectively. The differences in PA are much lower (−10 and −13 kJ mol^−1^ for **1** and **2**), with the basicity of phosphorus decreasing in the closed forms. This can be attributed to an increased mesomeric effect of the boron center in *ortho* position to phosphorus, modulating the electron density via the linking phenyl ring. The strong modulation of Lewis acidity between photochemical states, while leaving the Lewis basicity of the vicinal phosphine much less affected, results in pronounced differences of the overall ambiphilicity and prompted the investigation of photoreversible substrate activations.

**FIGURE 4:**
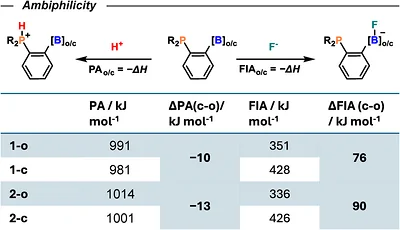
Fluoride ion affinities (FIA) and proton affinities (PA) of **1** and **2** in both photoisomers. Method: DSD‐BLYP(D3BJ)/def2‐QZVPP/PBEh‐3c. FIAs with TMS/TMSF anchor [43].

### 2.4 Reversible Substrate Binding and Kinetic Gating

As an initial probe of reversible FLP reactivity, we examined the interaction of compound **1** with representative polar substrates known to undergo cooperative binding by FLPs. To this end, compound **1‐o** was mixed with benzaldehyde (PhCHO) or phenyl isothiocyanate (PhNCS) in C_6_D_6_. For benzaldehyde, an equilibrium between the free FLP and the cooperatively bound substrate was observed, with a ratio of 6:4 for **1‐o‐PhCHO** versus **1‐o**. After UV‐induced ring‐closure, the equilibrium is shifted completely to **1‐c‐PhCHO**, with no free **1‐c** detected. For **1‐c‐PhCHO**, two diastereomers with a ratio of 53:47 are formed, due to the chirality of the closed dithienylethene and the bound aldehyde, resulting in two ^31^P NMR resonances (Figure 5a). For the combination of **1‐o** and PhNCS, a ratio of 20:1 between **1‐o** and **1‐o‐PhNCS** is formed. Upon ring‐closure, quantitative formation of **1‐c‐PhNCS** is observed, with a single ^31^P NMR resonance (Figure 5b). The cooperative binding for both substrates is supported by upfield shifts of the ^11^B NMR resonances to 8.7 ppm (PhNCS) and 10.4 ppm (PhCHO), as expected for tetracoordinate boron environments. Upon irradiation with visible light, the open forms and the initial equilibria were restored for both substrates, demonstrating fully reversible, light‐controlled substrate binding.

**FIGURE 5:**
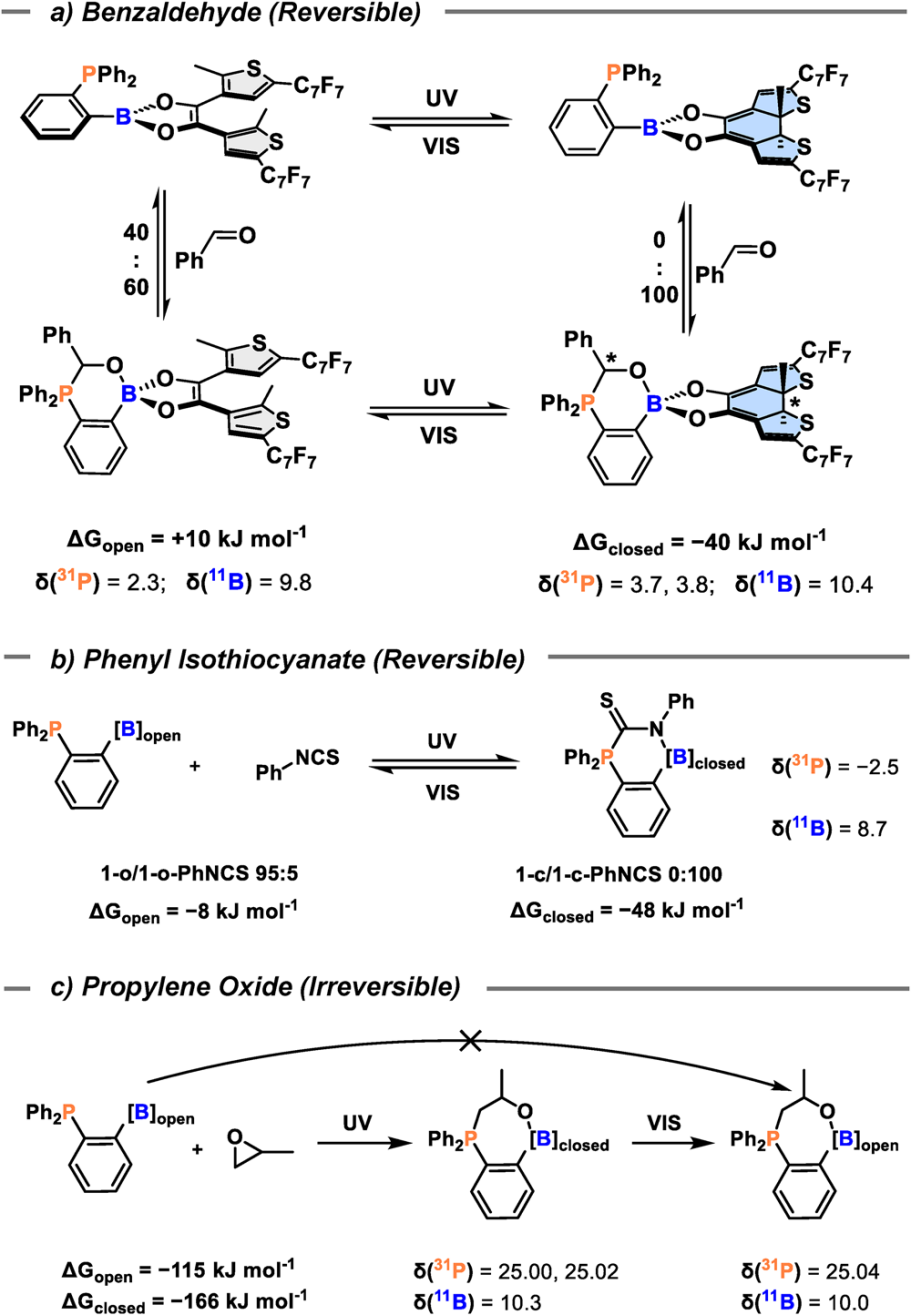
Reversible binding of (a) Benzaldehyde (PhCHO) and (b) phenyl isothiocyanate (PhNCS) by photoisomerization. Thermodynamic data obtained by DFT (DSD‐BLYP(D3BJ)/def2‐QZVPP/SMD(C_6_H_6_)//PBEh‐3c). (c) Irreversible light‐gated ring opening of propylene oxide upon photocyclization. All reactions were carried out in C_6_D_6_.

The observed equilibria are supported by DFT calculations within the computational error, giving close‐to‐thermoneutral binding free energies in the open forms, while exergonic binding is predicted in the closed forms. To investigate substrate activation over multiple cycles, a solution of **1** and PhNCS in C_6_D_6_ was subjected to alternating UV and VIS irradiation. After 5 cycles, substrate activation capability was almost entirely retained, with only 6% of the complex decomposed (Section S4.5). With compound **2**, quantitative adduct formation with aldehydes and PhNCS is already observed in the open form, owing to the more basic phosphine moiety (Section S4.6). The quantitative binding of *p*‐tolylaldehyde to the open form **2‐o** allowed for its crystallization, supporting the proposed cooperative substrate activation mode (Section S5). In crystallization attempts for FLP adducts in the closed form, only amorphous material were obtained, which is consistent with the general scarcity and limited quality of closed‐form diarylethene x‐ray structures [44].

While the reversible activation of PhCHO and PhNCS demonstrated photomodulation of substrate binding thermodynamics, the low activation barriers precluded insight into the impact of photoswitching on the kinetics of substrate activations. We therefore investigated a substrate activation process in which modulation of the *effective* Lewis acidity would influence the reaction barrier. In this context, the ring‐opening of epoxides by intramolecular FLPs has been extensively studied [45]. In contrast to the reversible binding of PhCHO and PhNCS, epoxide binding is strongly exergonic for both photochemical states (−115 and −167 kJ mol^−1^ for **1‐o** and **1‐c**). Consequently, differences in reactivity between the open and closed forms should primarily reflect differences in activation energies rather than thermodynamic driving force. **1‐o** was treated with propylene oxide (PO) in C_6_D_6_. No conversion was observed after 24 h at room temperature, indicating that the Lewis acidity of the open form is insufficient to promote epoxide activation. Upon irradiation at 300 nm, however, photocyclization to **1‐c** triggered formation of the epoxide adduct **1‐c‐PO** (as a mixture of two diastereomers, Figure 5c) and **1‐o‐PO** within the irradiation time, with small amounts of residual **1‐o**. The formation of **1‐o‐PO** is likely a result of cycloreversion of **1‐c‐PO** at the irradiation wavelength. Subsequent irradiation with visible light regenerated the open form while retaining the bound substrate, affording the ring‐opened product **1‐o‐PO**. These observations demonstrate that photoswitching in this system can influence not only substrate binding equilibria, but also the kinetics of substrate activation. Of note, the photoisomerization back to **1‐o‐PO** provides access to a kinetically otherwise inaccessible state under ambient conditions.

The results for compound **1** demonstrate photoswitchable FLP reactivity in an ambiphilicity regime suitable for reversible activation of polar substrates. We next examined compound **2**, whose increased phosphine basicity was expected to favor more challenging substrate activation while preserving the pronounced modulation of Lewis acidity upon switching. Pressurizing a CD_2_Cl_2_ solution of **2‐o** with 4 atm CO_2_ resulted in no detectable changes in the ^1^H or ^31^P NMR spectra. Irradiation of the same sample at 300 nm led to the quantitative formation of **2‐c‐CO_2_**, with a ^31^P NMR shift of 12.7 ppm. Subsequent irradiation with visible light restored the open form **2‐o** with release of CO_2_. To confirm the proposed binding mode, the experiment was repeated using ^13^C‐enriched CO_2_ (Figure 6a). Formation of **2‐c‐^13^CO_2_** resulted in a doublet in the ^31^P NMR spectrum with a ^1^J_P‐C_ coupling constant of 93 Hz, accompanied by a corresponding doublet at 161.5 ppm in the ^13^C NMR spectrum.

**FIGURE 6:**
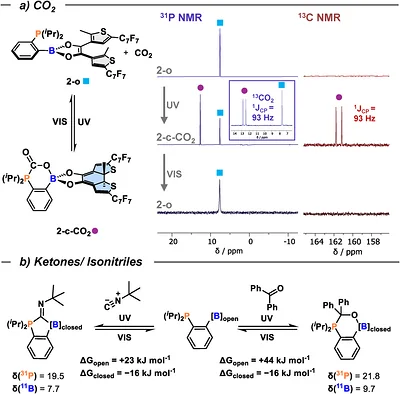
(a) Reversible binding of CO_2_ by photoisomerization of **2**. ^31^P NMR spectra in CD_2_Cl_2_ of **2‐o** (top), after irradiation and conversion to **2‐c‐CO_2_** (middle) and subsequent vis irradiation, regenerating **2‐o** (bottom). Inset: ^31^P NMR of **2‐c‐CO_2_** spectrum with ^13^CO_2_. Right, ^13^C NMR spectra during photoisomerization of **2** with ^13^CO_2_. (b) Reversible binding of benzophenone and *tert*‐butylisonitrile by photoisomerization. Reactions were carried out in C_6_D_6_.

Fully reversible substrate activation was also observed for benzophenone (BP) and *tert*‐butyl isonitrile (*^t^* BuNC) (Figure 6b). In both cases, no binding is observed in the open form, while UV irradiation resulted in quantitative formation of **2‐c‐BP** and **2‐c‐*^t^* BuNC**, respectively, with the expected changes in the ^31^P and ^11^B NMR spectra (Figure 6b). Irradiation with visible light restored the initial NMR spectra in both cases. Direct photochemical activation of the substrates was excluded by control experiments (Section S4.6). The proposed binding modes are well supported by DFT calculations, reproducing the observed switch from endergonic to exergonic substrate binding. For benzophenone, the steric demand results in slow dynamics and broadened ^1^H NMR resonances in the closed form (Section S4.6). The binding of *^t^* BuNC potentially gives rise to *E* and *Z* conformations of the resulting imine, but only one set of signals is observed in the ^1^H NMR spectrum. The *^t^* Bu‐phosphorus *E* conformation (displayed in Figure 6b) is favored by 34 kJ mol^−1^, supporting the observed selectivity. The observed stereoselectivity for *^t^* BuNC illustrates how substrate effects can influence binding equilibria. Sterically less demanding isonitriles might lead to different E/Z ratios, and overall steric crowding can disfavor reversible substrate uptake altogether.

The reversible activation of CO_2_, benzophenone, and *^t^* BuNC demonstrates that photoswitching of ambiphilicity remains effective also in compound **2**. Across all substrates studied, photoisomerization alone is sufficient to switch between the activated and unbound states, without variation of temperature, pressure, or chemical composition. For CO_2_, this behavior results in reversible uptake and release of the substrate under otherwise constant environmental conditions, representing a rare example of light‐controlled, reversible CO_2_ binding by an FLP. The reversible uptake and release of *^t^* BuNC is noteworthy given the ubiquity of isonitriles as ligands in transition‐metal chemistry. In principle, such behavior could provide a photochemical means of dynamically controlling ligand availability without changing the overall chemical composition of the system.

### 2.5 Photoswitchable Z‐Type Interactions

While the preceding examples utilize photoswitchable ambiphilicity for reversible substrate activations, ambiphilic ligands can also bind to electron‐rich metal centers through supported Z‐type interactions [3]. Compound **2‐o** readily coordinates to gold(I) chloride, allowing the synthesis and crystallization of the complex **2‐o‐AuCl**. In the solid‐state structure (Figure 7a), the Au‐B distance is 3.100(7) Å, which is lower than the sum of the Van‐der‐Waals radii (3.7 Å). This, along with the position of the gold center perpendicular to the trigonal borane plane, suggests a weak, but non‐zero Z‐type interaction in the open form. The photoisomerization proceeds with remarkably high quantum yield (Φ = 0.96), even in DCM. This may be reasoned by the following: i) **2‐o‐AuCl** preferably adopts a photoactive conformation, which is supported by the solid‐state structure, ii) The Lewis basic phosphine, which was proposed as the reason for the low quantum yield of **2‐o** in DCM, is quenched by the coordination to gold, and iii) the d^10^ metal center and the lack of low‐energy MLCT states (Section S3.5 for TDDFT) does not provoke any interference with the photochemistry of the diarylethene. Crystallization attempts of **2‐c‐AuCl** only led to thin plates unsuitable for x‐ray diffraction, but the Au‐B distance is predicted to contract in the closed form based on DFT calculations (3.19 Å → 2.93 Å, ESI Section S3.4).

**FIGURE 7:**
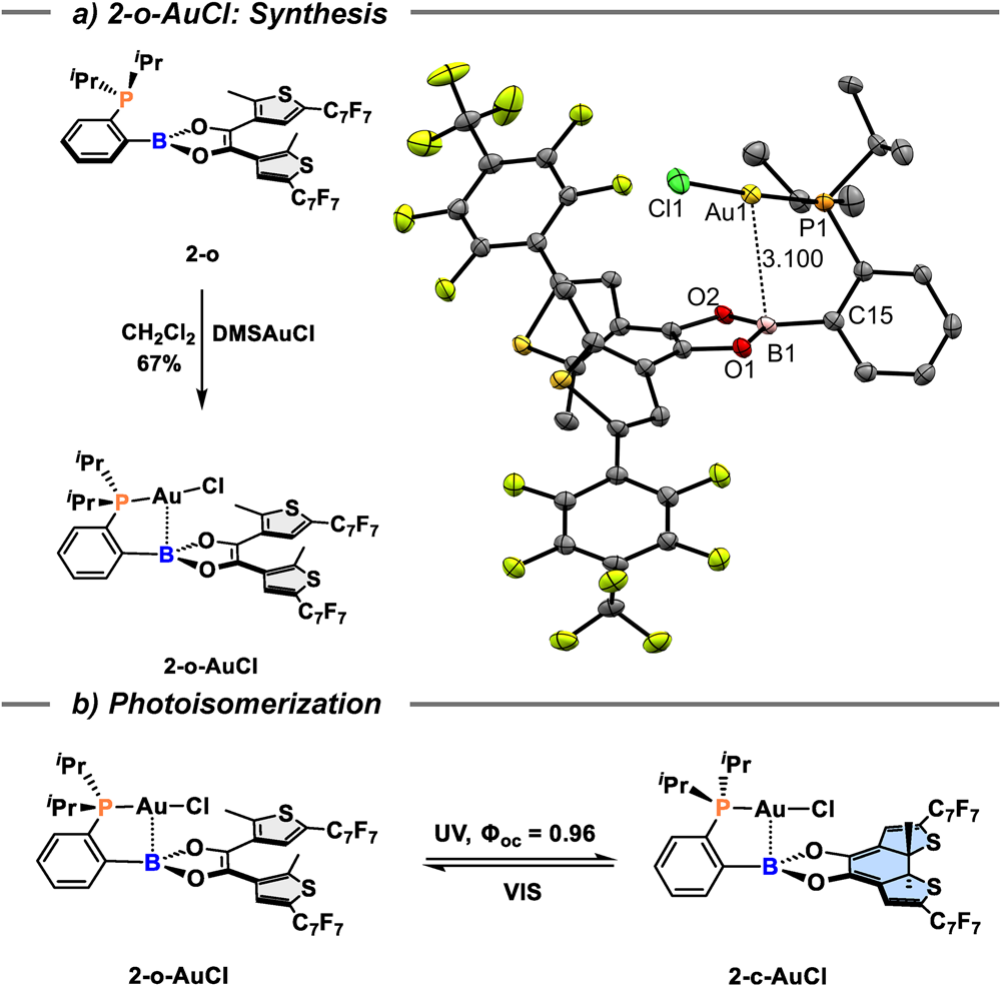
(a) Synthesis and solid‐state structure of **2‐o‐AuCl**. Hydrogen atoms are omitted for clarity. Thermal ellipsoids are displayed at the 50% probability level. Selected bond lengths [pm] and angles [°]: P1‐Au1: 223.9(2), Au1‐Cl1: 228.2(2), Au1‐B1: 310.0(7), O1‐B1‐C15: 123.9(5), O2‐B1‐C15: 125.2(5), O1‐B1‐O2: 110.3(5), P1‐Au1‐Cl1: 177.02(6). (b) Photoisomerization to **2‐c‐AuCl** in DCM.

This prompted us to investigate a coordination motif in which changes in Z‐type bonding could be followed more directly. Rh(I)→B Z‐type interactions are well documented [34] and allow for additional ligands to probe the transition metal's electronic features. Isonitrile ligands provide a sensitive spectroscopic handle, as their CN stretching frequencies directly reflect changes in metal electron density and are therefore expected to respond to modulation of the Rh→B interaction. For this purpose, Rh(I) complex **2‐o‐Rh(CNCy)_2_Cl** (Figure 8a) was synthesized by reaction of **2‐o** with [Rh(COD)Cl]_2_ and subsequent ligand exchange of cyclooctadiene by cyclohexyl isonitrile (CNCy). The ^1^H NMR spectrum shows one set of signals for the thiophene residues and the cyclohexyl groups, suggesting a trans‐conformation of the isonitrile ligands in a square‐planar coordination environment. A second isomer is present in solution (4%), which shows similar signals in the ^1^H NMR spectra but a reduced symmetry: likely the result of cis‐trans isomerization (Section S1.5 for further details). The conformation of the main isomer was confirmed by the solid‐state structure (Figure 8a, left). A moderate Z‐Type interaction can be observed in the open form, with a Rh‐B distance of 2.744 Å and pyramidalization of the boron center (ΣB_α_ = 356.3°). Dissolving the crystallized product results in a lower percentage of the minor isomer (1%), confirming the solid‐state structure as belonging to the main isomer. In the ^31^P NMR spectrum, a doublet (^1^ *J*_Rh‐P_ = 162 Hz) at 70.5 ppm can be observed. Upon irradiation at 365 nm, a new signal appears at 78.2 ppm (^1^ *J*_Rh‐P_ = 146 Hz), assigned to the closed form complex **2‐c‐Rh(CNCy)_2_Cl**. The shifted ^11^B NMR signal indicates further pyramidalization of the boron center in the closed form (29.9 (open) → 24.6 (closed) ppm). The CN stretching frequency is blue‐shifted by 33 cm^−1^ in the closed form (Figure 8a, right), indicating a decrease in Rh→CN *π*‐backbonding, which can be attributed to a strengthened Z‐Type interaction. Irradiation of **2‐c‐Rh(CNCy)_2_Cl** with visible light restored the open form, along with light‐induced cis‐trans isomerization as a side reaction. Complete cis‐trans isomerization upon photocyclization was estimated as less likely based on the single major CN stretching band in both major forms. DFT calculations give further support for the proposed trans‐conformation of the closed form, well‐reproducing the experimentally observed CN frequency blueshift (+27 cm^−1^, Figure 8b), and predicting a shortening of the Rh‐B distance from 2.65 Å to 2.30 Å. Based on the computed structure, the Z‐Type interaction was gauged by second‐order perturbation theory analysis, which showed Rh→B donation from the d_z_^2^ orbital of rhodium into the boron‐centered NBO (95% p‐character) with an energy of 80.2 kcal mol^−1^, while an energy of only 29.0 kcal mol^−1^ is predicted in the open form. In contrast to the gold(I) complex, the d^8^ metal center of Rh(I) and the *π*‐accepting isonitrile ligands potentially give rise to competing non‐productive electronic excited states. Indeed, for **2‐o‐Rh(CNCy)_2_Cl**, a low‐energy MLCT excitation into the CyNC‐Rh‐CNCy *π* system can be observed with a maximum at 382 nm (Figure 8c and Section S3.5 for TDDFT). Nevertheless, although longer irradiation times are required for the conversion to the closed form compared to the other cases, the high‐energy π─π* excitations of the ligand are still productive for the photocyclization, suggesting a substantial barrier for internal conversion to the metal‐centered excited state. In the closed form, the S1 transition is redshifted to 608 nm compared to the free ligand **2‐c** at 575 nm. The S2 transition is even more strongly redshifted (395→437 nm), with an increased intensity of the transition in relation to S1. Both the increased oscillator strength and the red‐shift of the S2 transition are predicted by TDDFT and likely originate from the mixing of π─π* ligand and MLCT transitions (NTOs in Figure 8c). The good agreement of the observed UV–vis spectral features with computational results further supports the proposed structure of the closed isomer.

**FIGURE 8:**
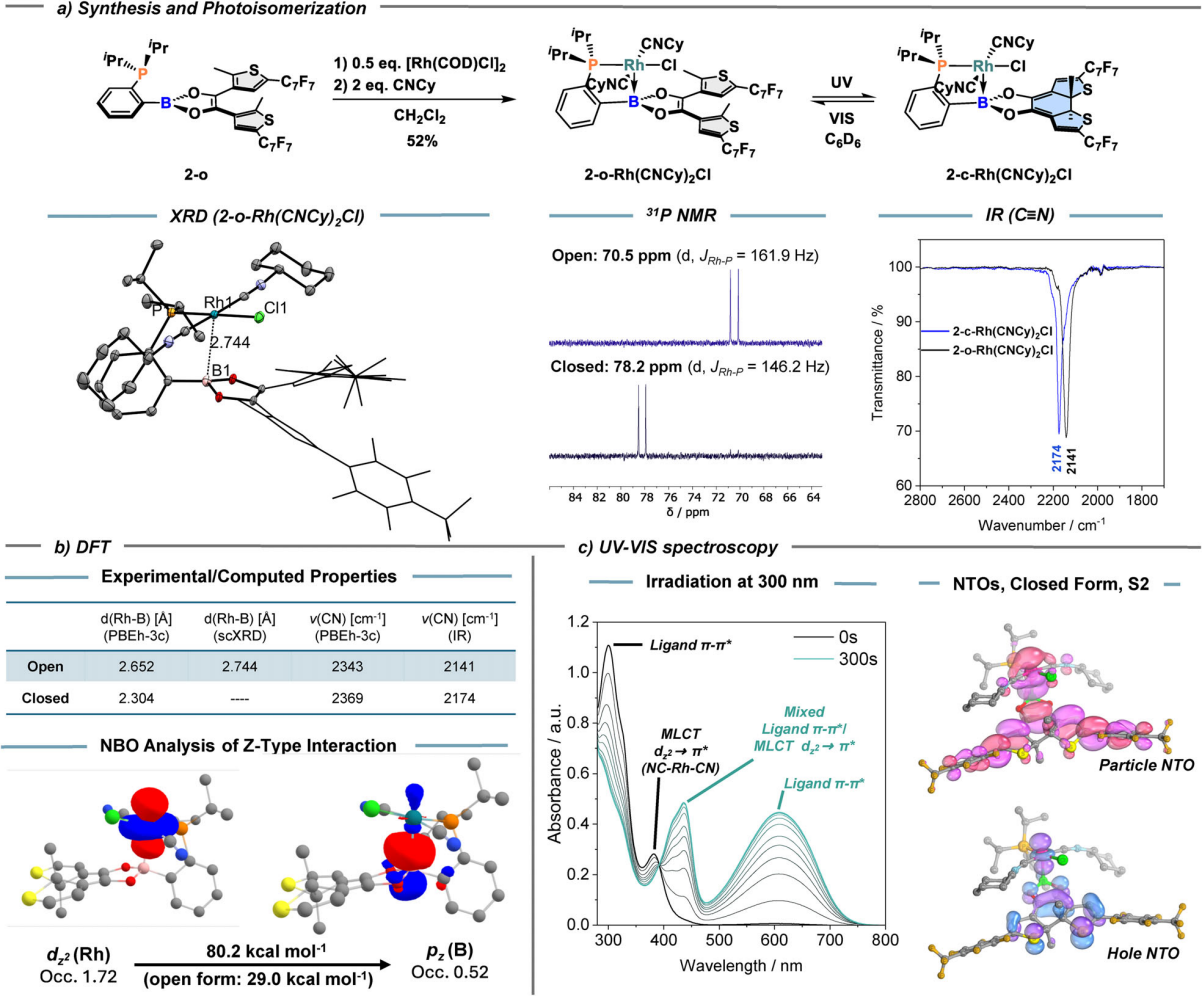
(a) Synthesis and solid‐state structure of **2‐o‐Rh(CNCy)_2_Cl**. Hydrogen atoms are omitted for clarity. Thermal ellipsoids are displayed at the 50% probability level. Selected bond lengths [pm]: P1‐Rh1: 222.18(8), Rh1‐Cl1: 238.62(8), Rh1‐B1: 274.4(3). Planarity of the boron and rhodium environments: ΣB_α_ = 356.3, ΣRh_α_ = 360.0. Right: ^31^P NMR and IR spectral changes upon photoisomerization to **2‐c‐Rh(CNCy)_2_Cl**. (b) Comparison of computed (PBEh‐3c) and experimental properties. NBO analysis (PBE0/def2‐TZVP) of the rhodium‐boron interaction (Section S3.4 for further details). (c) UV‐vis spectra (2.6 · 10^−5^ M, benzene) of **2‐Rh(CNCy)_2_Cl** during irradiation with 300 nm light. Right: natural transition orbitals of the S2 transition of **2‐c‐Rh(CNCy)_2_Cl**.

## 3 Conclusions

In this study, we introduced photoswitchable ambiphilicity by integrating a dithienylethene‐based photoswitch into the Lewis acidic boron fragment of intramolecular vicinal phosphorus/boron pairs. Photoisomerization induces pronounced changes in Lewis acidity while largely preserving the Lewis basicity of the phosphine, resulting in modulation of the overall ambiphilic character. The light‐induced change in ambiphilicity enables control over both kinetics and thermodynamics of substrate activations. Depending on the basicity of the phosphine moiety (**1**: PPh_2_, **2**: P(*^i^* Pr)_2_), different ambiphilicity regimes can be addressed, allowing for reversible activation of diverse substrates, including the light‐controlled binding and release of CO_2_ under constant external conditions. Additionally, the switching of Lewis acidity can provide kinetic control over substrate activation in the ring‐opening of epoxides. Beyond substrate activation, the ambiphilic framework also functions as a photoswitchable ligand in transition‐metal complexes, enabling reversible modulation of the metal‐boron Z‐type interaction of a Rh(I) complex and, consequently, the electronic properties of the metal center, opening up opportunities in light‐driven transition metal catalysis. Overall, this work establishes photoswitchable ambiphilicity as a versatile tool for externally controlling properties and reactivities in both main‐group and transition metal chemistry.

## Author Contributions

**L. S**.: Conceptualization, investigation, writing – original draft, methodology, validation, visualization, data curation, formal analysis. **E. L. M. P**.: Investigation, validation, data curation. **F. M**.: Investigation, validation, data curation. **L. G**.: Conceptualization, funding acquisition, writing – review and editing, project administration, supervision, resources.

## Conflicts of Interest

The authors declare no conflicts of interest.

## Supporting information



Full experimental procedures, characterization data, and computational details are provided in the Supporting Information. The authors have cited additional references within the Supporting Information [28, 40, 43, 46, 47, 48, 49, 50, 51, 52, 53, 54, 55, 56, 57, 58, 59, 60, 61]. CCDC 2574720‐2574723 contains the supplementary crystallographic data for this paper [62].**Supporting File**: anie74983‐sup‐0001‐SuppMat.pdf.

## Data Availability

The data that supports the findings of this study are available in the supplementary material of this article.
