# Supplementary material for: Photoswitchable Ambiphilicity: Light‐Gated Frustrated Lewis Pair Reactivity and Z‐Type Interaction
Source: Angew Chem Int Ed Engl. 2026 Sep 24;65(46):e5038244. doi: 10.1002/anie.5038244 (PMC13749345; doi:10.1002/anie.5038244)
Supplement: Supplementary file 1 [file ANIE-65-e5038244-s001.pdf]

Supporting Information

Photoswitchable Ambiphilicity: Light-Gated Frustrated Lewis Pair Reactivity and Z-Type Interaction

Lennart Stoess[a], Emil L. M. Paulin[a], Fabian Michel[a], and Lutz Greb*[a]

[a] Lennart Stoess, Emil Luca Matthias Paulin, Fabian Michel, Prof. Dr. Lutz Greb
Anorganisch-Chemisches Institut,
Ruprechts-Karls Universität Heidelberg
Im Neuenheimer Feld 270, 69120 Heidelberg (Germany)
E-mail: greb@uni-heidelberg.de

Table of Contents

1 Synthesis and Characterization Data 1
1.1 General Considerations 1
1.2 1-o 2
1.3 2-o 6
1.4 2-o-AuCl 10
1.5 2-o-Rh(CNCy)$_2$Cl 14
2 UV-vis spectroscopy 20
2.1 Extinction Coefficients 20
2.2 Photocyclization Quantum Yields 22
2.3 Fatigue Resistance 26
2.4 2-Rh(CNCy)$_2$Cl 28
3 Computational Section 29
3.1 General Information 29
3.2 Fluoride Ion Affinities and Proton Affinities 30
3.3 Substrate Binding Thermodynamics 31
3.4 Computed Structures and NBO analysis of 2-AuCl and 2-Rh(CNCy)$_2$Cl 32
3.4.1 2-AuCl 32
3.4.2 2-Rh(CNCy)$_2$Cl 33
3.5 TD-DFT 35
4 Reactivity Studies 40
4.1 Photocyclization (1) 40
4.1.1 1, Benzene 40
4.1.2 1, DCM 41
4.2 Photocyclization (2) 42
4.2.1 2, Benzene 42
4.2.2 2, DCM 43
4.3 Thermal Stability of the Closed Isomers 44
4.4 Substrate Activation (1) 45
4.4.1 Benzaldehyde 45
4.4.2 Phenyl Isothiocyanate 48
4.4.3 Propylene Oxide 51
4.5 Impact of Multiple Switching Cycles 54
4.6 Substrate Activation (2) 56
4.6.1 CO_2 56
4.6.2 Benzophenone 60
4.6.3 tert-Butyl Isonitrile 63
4.6.4 p-Tolylaldehyde/Phenyl Isothiocyanate 66
4.6.5 Control Experiments with tBuNC and Benzophenone 68
4.7 Photocyclization (2-AuCl) 69

4.8 Photocyclization (2-Rh(CNCy)$_2$Cl) ... 70

5 X-Ray Crystallography ... 74

6 Coordinates ... 80

7 References ... 107

1 Synthesis and Characterization Data

1.1 General Considerations

All experimental manipulations were performed under inert and anhydrous conditions using standard Schlenk techniques with either argon or nitrogen atmospheres. Air- and moisture-sensitive compounds were handled and stored in a nitrogen-filled Sylatech glovebox. Unless stated otherwise, solvents were purified by established drying methods, stored over 3 Å or 4 Å molecular sieves, and degassed immediately before use by three consecutive freeze–pump–thaw cycles. Trimethylborate for the phosphinoboronate ester synthesis[1] was stirred over sodium and distilled prior to use. **L¹** was synthesized according to published procedures.[2] All other commercially available reagents were used without further purification. The identity and purity of newly prepared compounds were verified by multinuclear NMR spectroscopy, high-resolution mass spectrometry, UV-vis spectroscopy and scXRD if possible. ^{1}H, ^{13}C, ^{19}F, ^{31}P, and ^{11}B NMR spectra were acquired at 298 K on either a *Bruker Ascend 400* or *Bruker Avance III 600* spectrometer unless otherwise noted. ^{13}C, ^{31}P, and ^{11}B NMR spectra were recorded with ^{1}H decoupling. Chemical shifts are reported in parts per million (δ, ppm) and referenced to the residual solvent resonances for ^{1}H and ^{13}C nuclei. Coupling constants (J) are expressed in hertz (Hz). Data processing and visualization were carried out using MestReNova 14.3. MALDI mass spectra were obtained using a Bruker timsTOFfleX instrument, LIFDI mass spectra on a JEOL AccuTOF GCx instrument, both at the mass spectrometry facility of the Institute of Organic Chemistry, University of Heidelberg. UV-vis spectral measurements were carried out on a JASCO V-570 UV/VIS/NIR spectrophotometer in a 2 mm or 10 mm quartz cuvette with a J-Young valve. ATR-IR spectra were recorded on an Agilent Cary 630 spectrometer inside a nitrogen-filled glovebox.

1.2 1-o

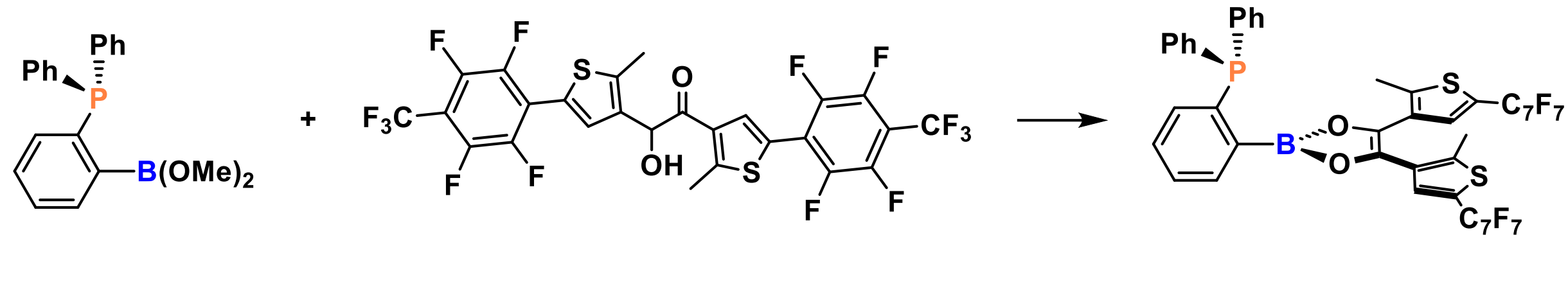

o-Ph$_2$P(Ph)B(OMe)$_2$ **L[1]** **1-o**

o-Ph$_2$P(Ph)B(OMe)$_2$ (0.532 g, 1.59 mmol, 1.10 eq) and **L**[1] (0.991 g, 1.45 mmol, 1.00 eq) were suspended in benzene (20 ml). The mixture was stirred for 10 minutes and the solvent subsequently removed *in vacuo*. The residue was redissolved in benzene (20 ml) and the solvent removed by lyophilization overnight, giving a yellow solid. The solid was suspended in *n*-hexane (20 ml), stirred vigorously for 10 minutes, and the solvent removed in vacuo. Suspension in *n*-hexane and removal of solvent was repeated two more times to remove traces of methanol. Afterwards, the solid was suspended in *n*-hexane (20 ml), filtered off, and washed with *n*-hexane (4x5 ml). Drying in vacuo gave **1-o** as an off-white powder with a faint blue hue (1.04 g, 75%). The product contains 0.6 equivalents of *n*-hexane after overnight drying.

^{1}H NMR (600 MHz, CD_2Cl_2) δ 8.12 – 8.08 (m, 1H, phenyl-C***H***), 7.49 (s, 2H, thiophene-C***H***), 7.47 – 7.40 (m, 2H, phenyl-C***H***), 7.35 – 7.28 (m, 10H, PPh$_2$-C***H***), 7.08 – 7.03 (m, 1H, phenyl-C***H***), 2.37 (s, 6H, thiophene-C***H***$_3$).

^{19}F NMR (377 MHz, CD_2Cl_2) δ -56.5 (t, *J* = 21.3 Hz, 6F, C***F***$_3$), -138.9 – -139.5 (m, 4F, C***F***), -141.4 – -142.3 (m, 4F, C***F***).

^{13}C NMR (151 MHz, CD_2Cl_2) δ 145.0 (dm, J_{CF} = 259.0 Hz, ***C***F), 144.5 (d, J_{CP} = 19.1 Hz, phenyl-***C***P), 144.1 (dm, J_{CF} = 251.7 Hz, ***C***F) 143.8 (t, J_{CF} = 4.5 Hz, ***C***$_q$), 138.2 (d, J_{CP} = 12.2 Hz, PPh$_2$-***C***P), 136.7 (s, ***C***$_q$), 136.5 (d, J_{CP} = 9.7 Hz, phenyl-***C***H), 134.3 (d, J_{CP} = 19.9 Hz, PPh$_2$-***C***H), 133.9 (s, phenyl-***C***H), 132.4 (t, J_{CF} = 6.4 Hz, thiophene-***C***H), 131.7 (s, phenyl-***C***H), 129.1 (s, PPh$_2$-***C***H), 128.9 (d, J_{CP} = 6.9 Hz, PPh$_2$-***C***H), 128.3 (s, phenyl-***C***H), 128.0 (s, ***C***$_q$), 123.6 (s, ***C***$_q$), 121.4 (q, J_{CF} = 274.5 Hz, ***C***F$_3$), 118.6 (t, J_{CF} = 14.3 Hz, ***C***$_q$), 107.6 – 106.7 (m, ***C***-CF$_3$), 14.7 (s, ***C***H$_3$).

Boron bound carbon atom is not observed.

^{11}B NMR (193 MHz, CD_2Cl_2) δ 31.9.

^{31}P NMR (243 MHz, CD_2Cl_2) δ -4.8.

HRMS (MALDI): [$C_{44}H_{22}BF_{14}O_2PS_2$], calcd.: 954.0668, found: 954.0673.

UV-vis (DCM): λ_{max} (ε) = 290 nm (58000 M^{-1}cm^{-1}).

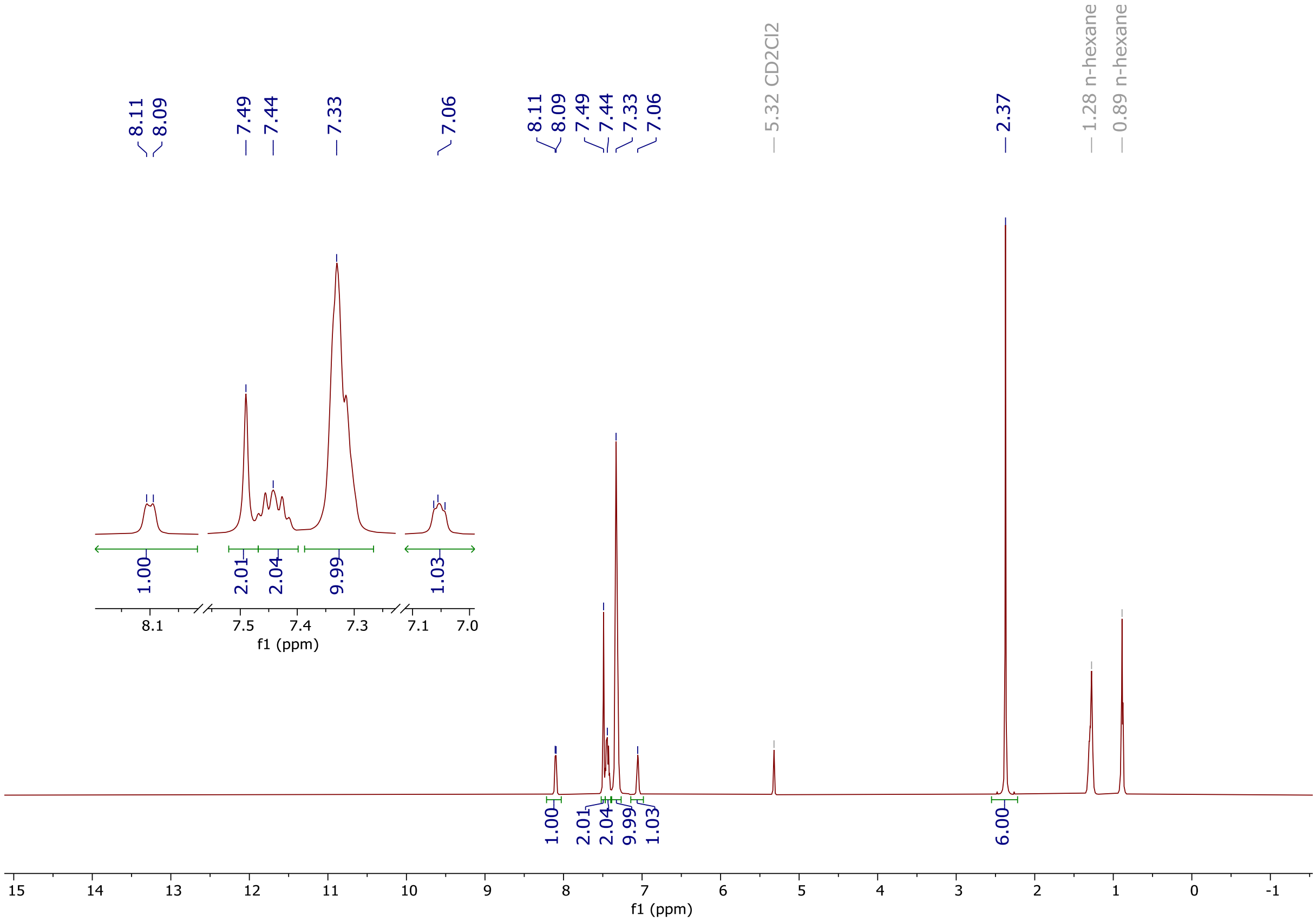

Figure S1. ^{1}H NMR spectrum (600 MHz, CD_2Cl_2) of **1-o**.

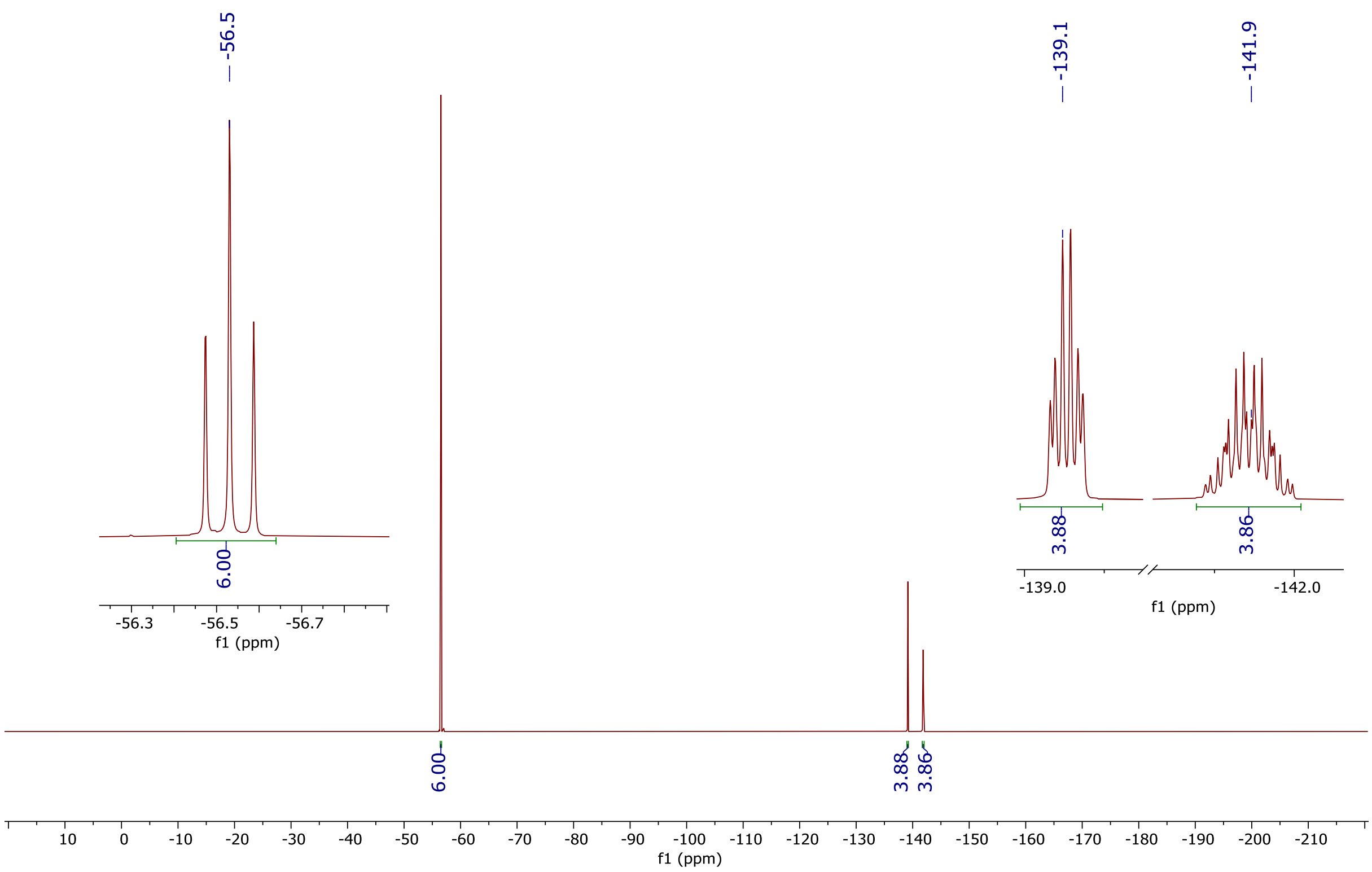

Figure S2. ^{19}F NMR spectrum (377 MHz, CD_2Cl_2) of **1-o**.

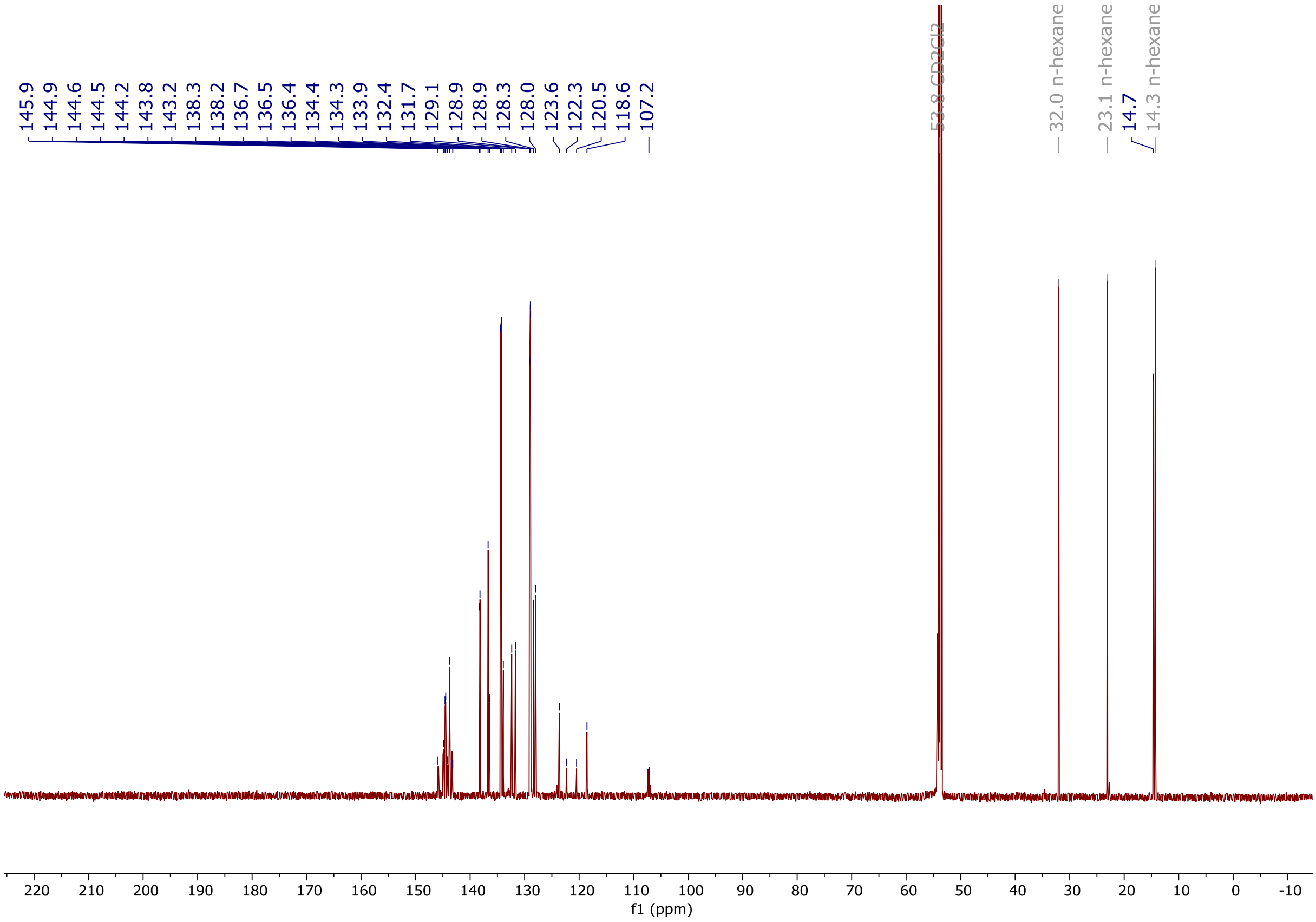

Figure S3. ^{13}C NMR spectrum (151 MHz, CD_2Cl_2) of **1-o**.

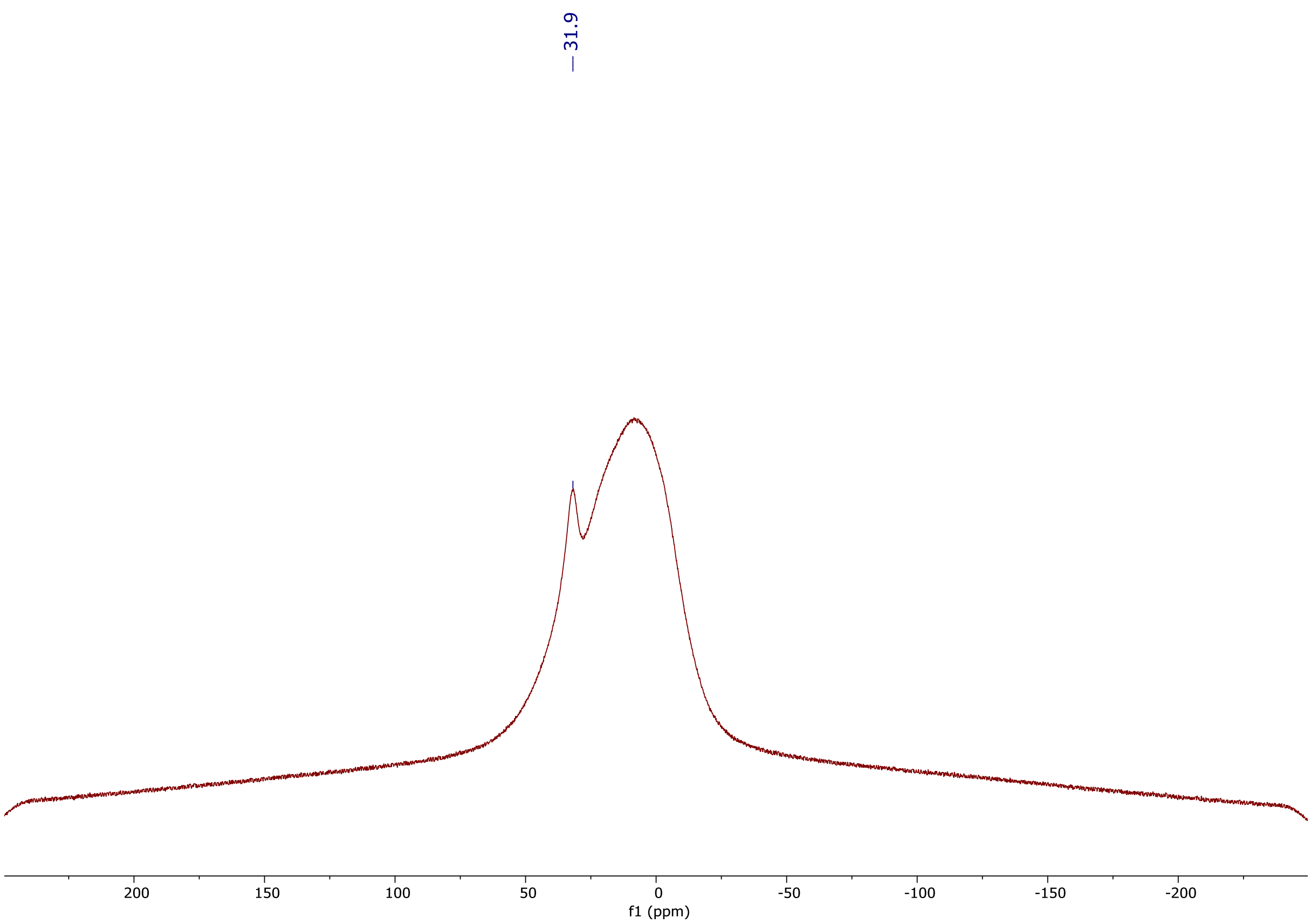

Figure S4. ^{11}B NMR spectrum (193 MHz, CD_2Cl_2) of **1-o**.

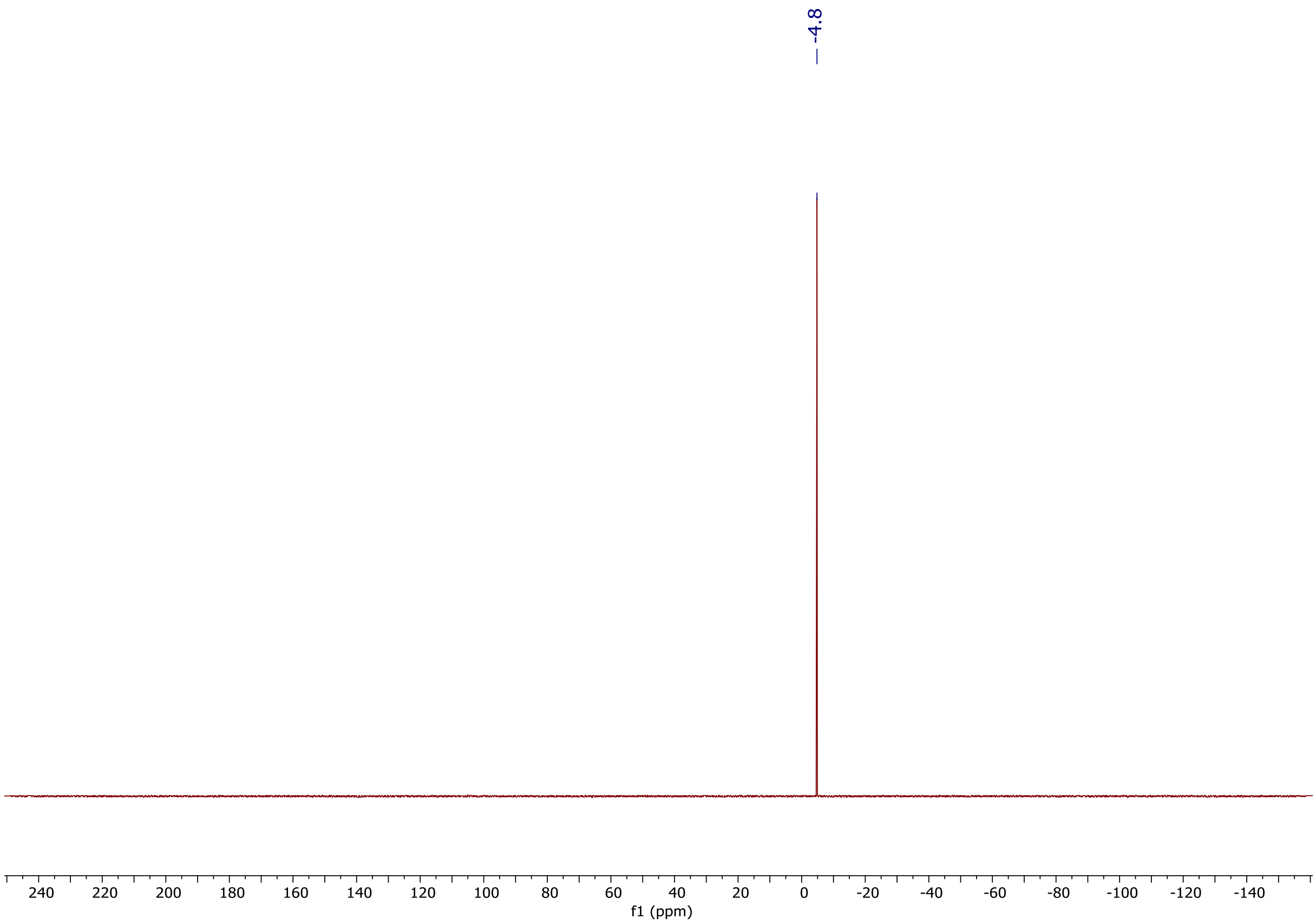

Figure S5. ^{31}P NMR spectrum (243 MHz, CD_2Cl_2) of **1-o**.

1.3 2-o

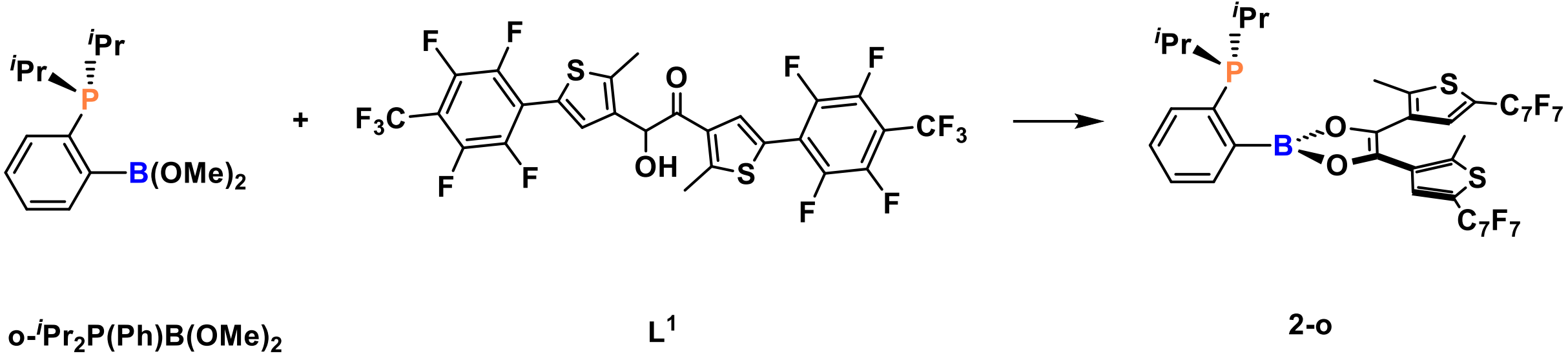

o-$^{i}Pr_2P(Ph)B(OMe)_2$ (0.710 g, 2.67 mmol, 1.05 eq) and **L¹** (1.741 g, 2.54 mmol, 1.00 eq) were dissolved in benzene (20 ml). The mixture was stirred for 10 minutes and the solvent subsequently removed *in vacuo*. The residue was redissolved in benzene (20 ml) and the solvent removed by lyophilization overnight, giving a light green foam. This residue was suspended in *n*-hexane (20 ml), stirred vigorously for 10 minutes and the solvent removed *in vacuo*. Suspension in *n*-hexane and removal of solvent was repeated two more times to remove traces of methanol. Afterwards, the solid was dissolved in a 1:2 DCM/n-hexane mixture (ca. 20 ml). After storage at -40 °C overnight, the precipitated solid was filtered off and washed with *n*-hexane (2x5 ml). Drying *in vacuo* overnight gave **2-o** as a light green powder, containing small amounts of residual DCM and *n*-hexane (1.86 g, 83%). Crystals suitable for SCXRD were obtained by cooling a saturated DCM/*n*-hexane solution to -40 °C.

^{1}H NMR (600 MHz, CD_2Cl_2) δ 7.94 – 7.86 (m, 1H, phenyl-C***H***), 7.68 – 7.63 (m, 1H, phenyl-C***H***), 7.62 (s, 2H, thiophene-C***H***), 7.57 – 7.49 (m, 1H, phenyl-C***H***), 7.48 – 7.41 (m, 1H, phenyl-C***H***), 2.54 (s, 6H, thiophene-C***H***$_3$), 2.18 (m, 2H, ^{i}Pr-C***H***), 1.14 (dd, $^{3}J_{PH}$ = 14.5, $^{3}J_{HH}$ = 6.9 Hz, 6H, ^{i}Pr-C***H***$_3$), 0.96 (dd, $^{3}J_{PH}$ = 11.8, $^{3}J_{HH}$ = 6.9 Hz, 6H, ^{i}Pr-C***H***$_3$).

^{19}F NMR (377 MHz, CD_2Cl_2) δ -56.5 (t, *J* = 21.3 Hz, 6F, C***F***$_3$), -139.0 – -139.4 (m, 4F, C***F***), -141.7 – -142.1 (m, 4F, C***F***).

^{13}C NMR (151 MHz, CD_2Cl_2) δ 145.0 (dm, J_{CF} = 257.9 Hz, ***C***F), 144.1 (dm, J_{CF} = 251.8 Hz, ***C***F), 143.7 (t, J_{CF} = 4.6 Hz, ***C***$_q$), 143.1 (d, J_{CP} = 21.6 Hz, ***C***P), 136.6 (s, ***C***$_q$), 135.1 (d, J_{CP} = 12.7 Hz, phenyl-***C***H), 132.6 (d, J_{CP} = 2.7 Hz, phenyl-***C***H), 132.5 (t, J_{CF} = 6.5 Hz, thiophene-***C***H), 130.3 (s, phenyl-***C***H), 128.5 (s, phenyl-***C***H), 128.2 (s, ***C***$_q$), 123.7 (s, ***C***$_q$), 121.4 (q, J_{CF} = 274.1 Hz, ***C***F$_3$), 118.6 (t, J_{CF} = 14.2 Hz, ***C***$_q$), 108.0 – 106.5 (m, ***C***-CF$_3$), 24.7 (d, J_{CP} = 13.6 Hz, ^{i}Pr-***C***H), 20.3 (d, J_{CP} = 18.3 Hz, ^{i}Pr-***C***H$_3$), 19.8 (d, J_{CP} = 10.8 Hz, ^{i}Pr-***C***H$_3$), 14.9 (s, thiophene-***C***H$_3$).

Boron bound carbon atom is not observed.

^{11}B NMR (193 MHz, CD_2Cl_2) δ 32.8.

^{31}P NMR (243 MHz, CD_2Cl_2) δ 7.6.

HRMS (MALDI): [$C_{38}H_{26}BF_{14}O_2PS_2$], calcd.: 886.0981, found: 886.0973.

UV-vis (DCM): λ_{max} (ε) = 294 nm (47000 $M^{-1}cm^{-1}$).

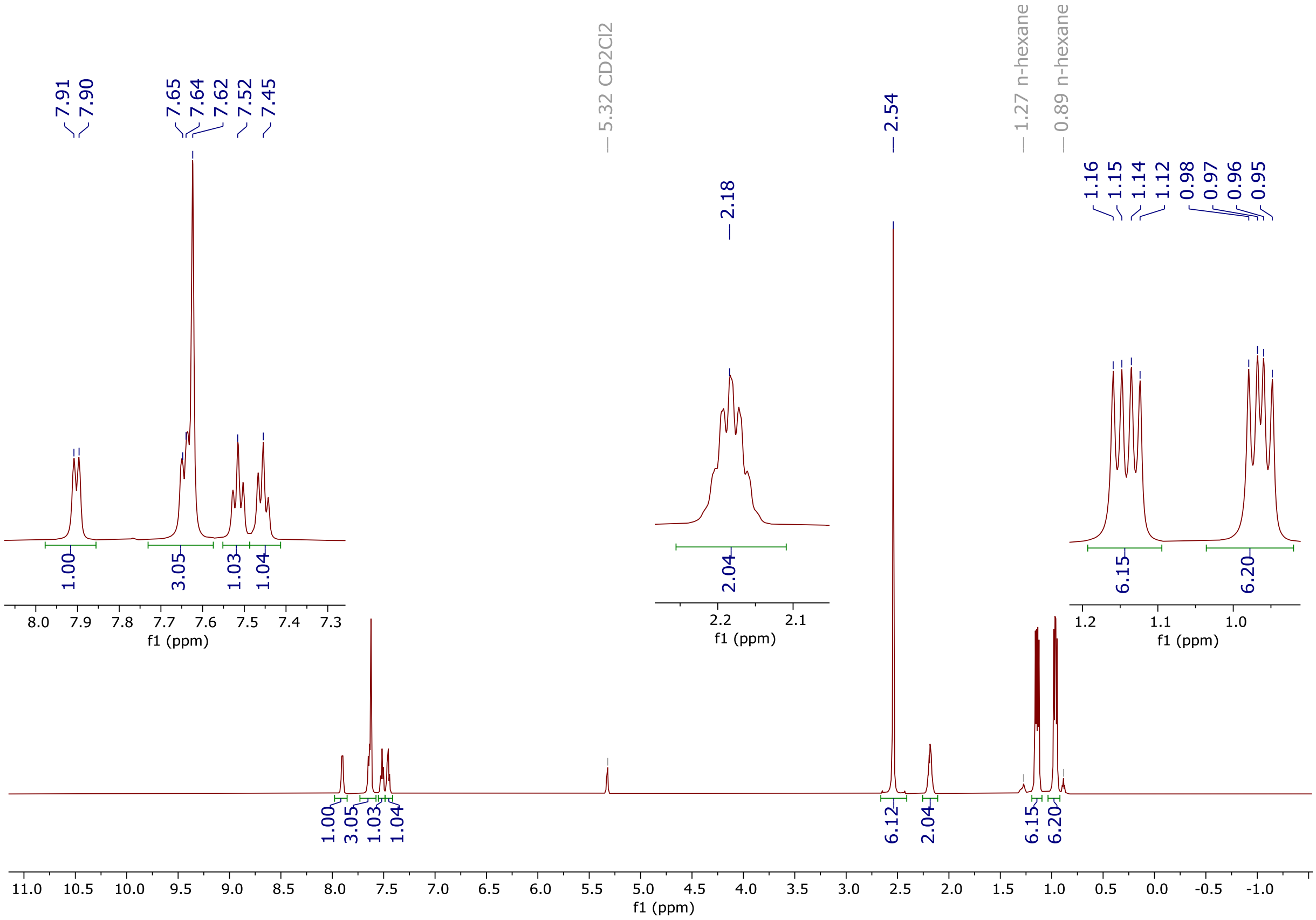

Figure S6. ^{1}H NMR spectrum (600 MHz, CD_2Cl_2) of **2-o**.

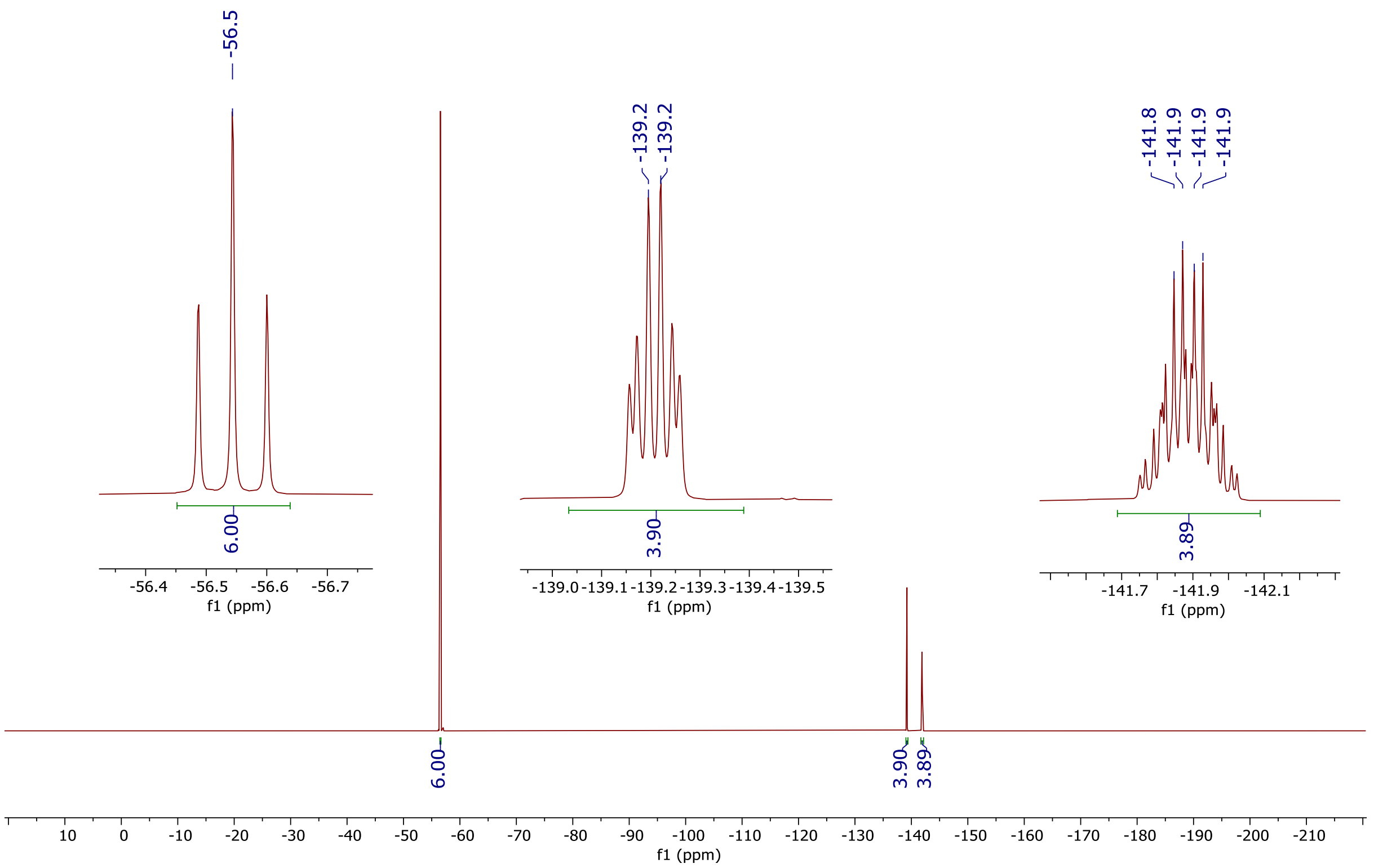

Figure S7. ^{19}F NMR spectrum (377 MHz, CD_2Cl_2) of **2-o**.

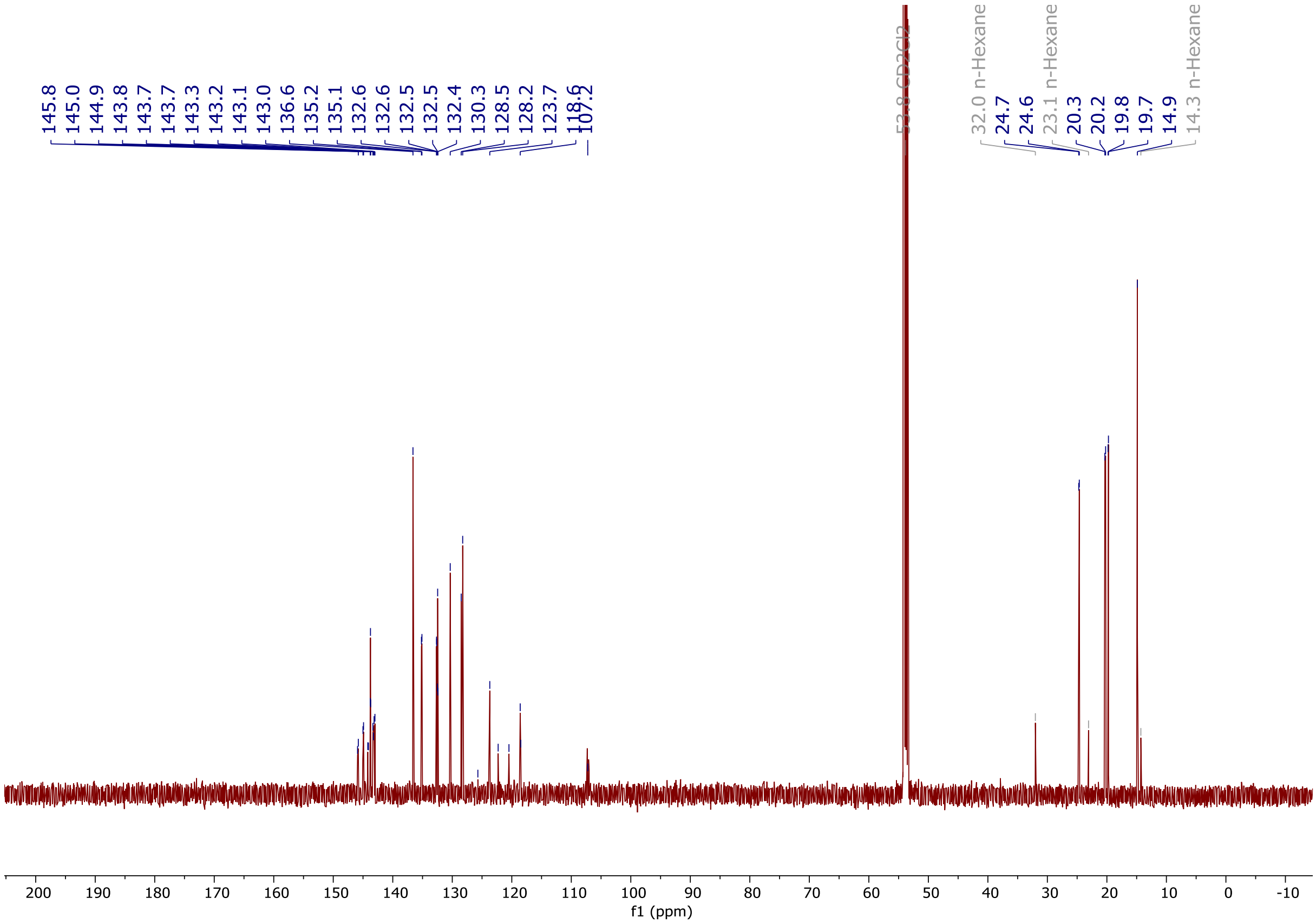

Figure S8. ^{13}C NMR spectrum (151 MHz, CD_2Cl_2) of **2-o**.

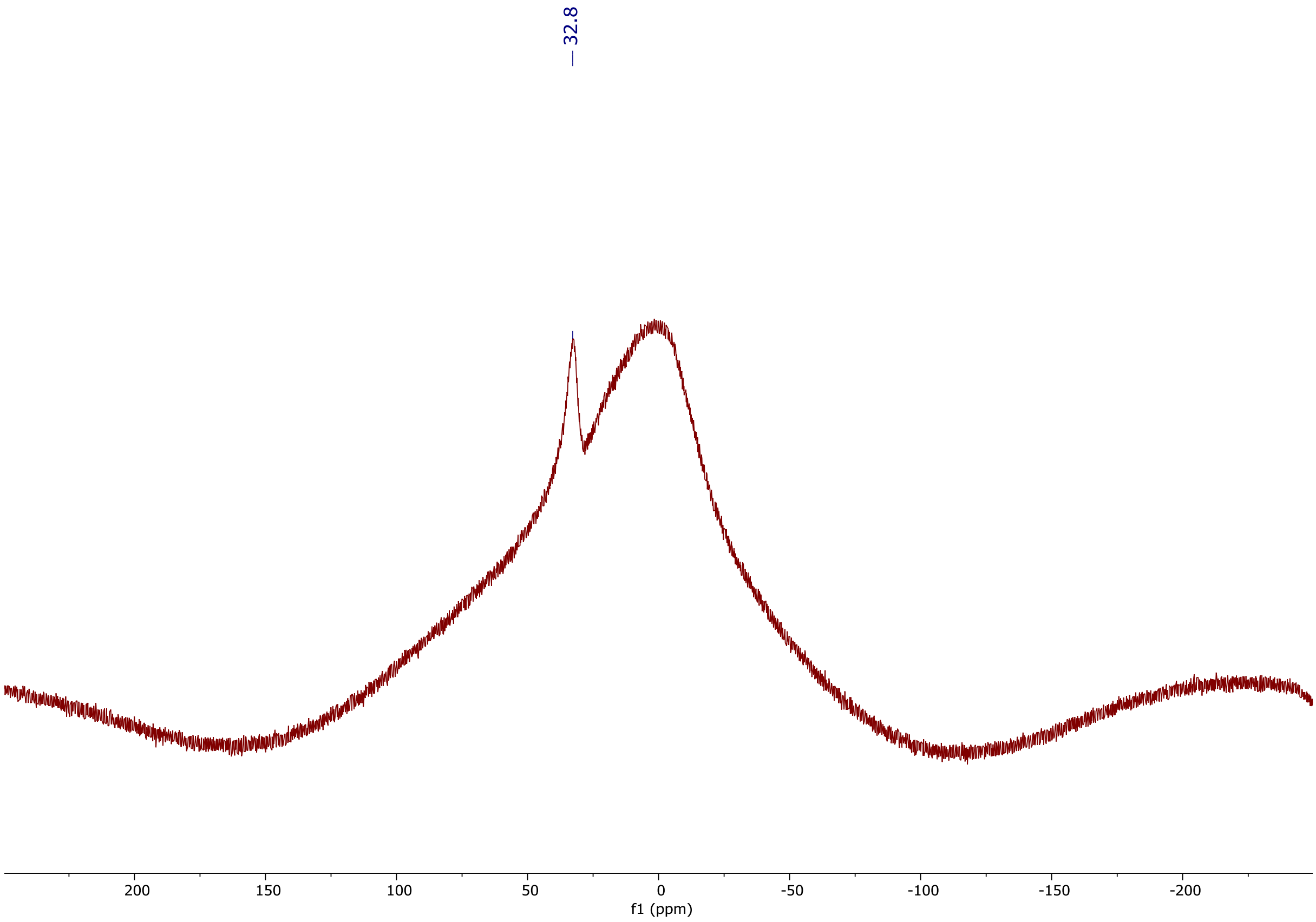

Figure S9. ^{11}B NMR spectrum (193 MHz, CD_2Cl_2) of **2-o**.

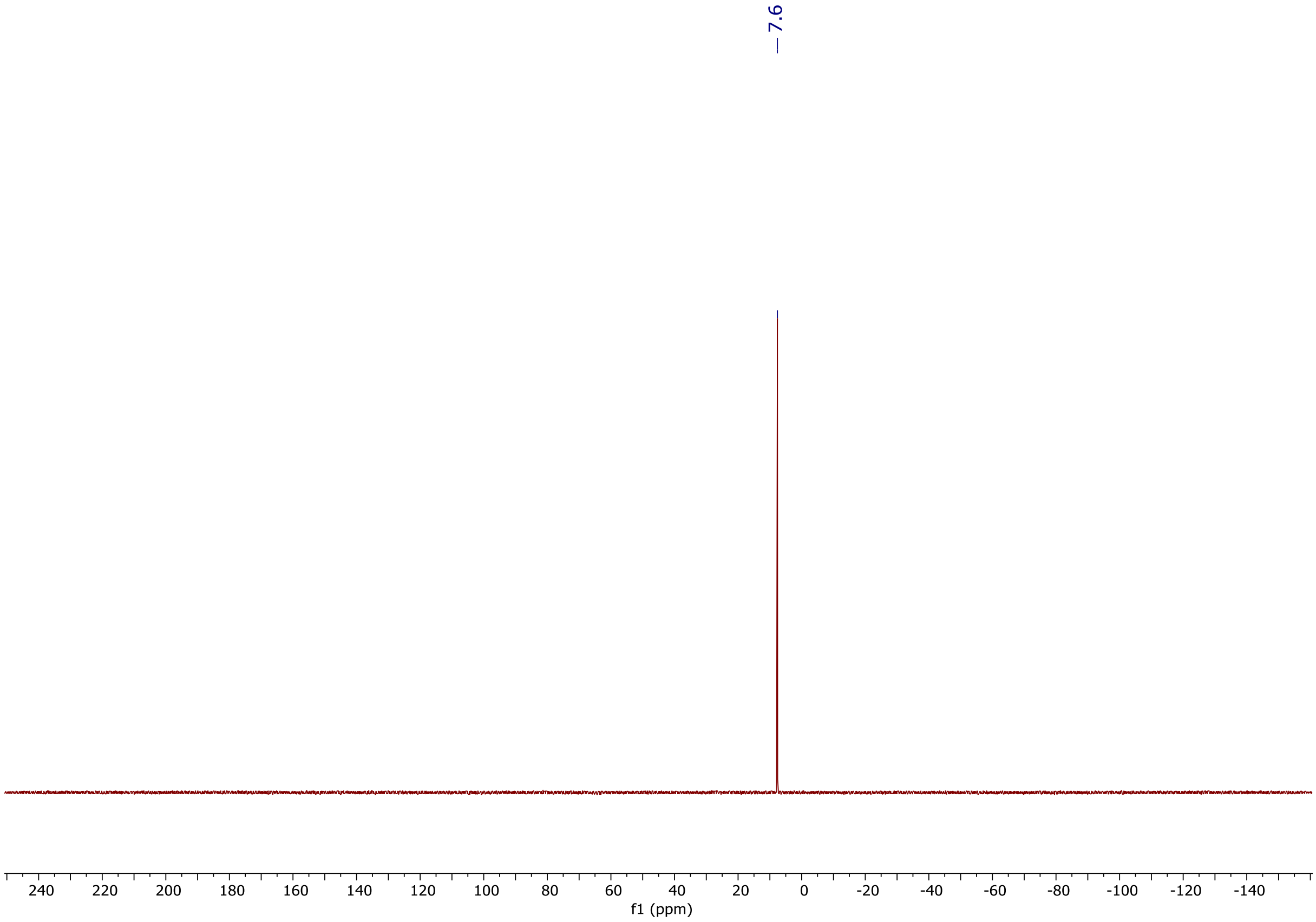

Figure S10. ^{31}P NMR spectrum (243 MHz, CD_2Cl_2) of **2-o**.

1.4 2-o-AuCl

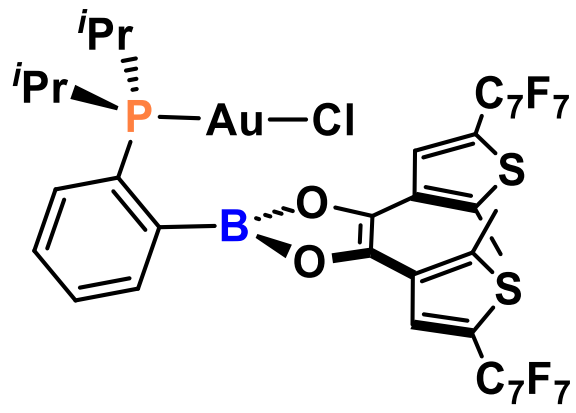

2-o (148.0 mg, 167 µmol, 1.0 eq) was added in portions at -40 °C to a solution of dimethylsulfide gold(I)chloride (49.2 mg, 167 µmol, 1.0 eq) in DCM (1 ml). The solution was stirred for 10 minutes before passing through a syringe filter to remove small amounts of formed gold powder. *n*-hexane (4 ml) was added and the solution was stored at -40 °C for 6 days, resulting in the precipitation of the product as a colorless solid. The solid was filtered off, washed with *n*-hexane (4x2 ml) and dried *in vacuo* overnight. The product contains 0.35 equivalents of DCM after drying *in vacuo* (126 mg, 67%). Crystals suitable for scXRD were obtained by gaseous diffusion of n-pentane into a saturated DCM solution at -40 °C.

^{1}H NMR (600 MHz, CD_2Cl_2) δ 7.89 – 7.83 (m, 1H, phenyl-C***H***), 7.73 (s, 2H, thiophene-C***H***), 7.67 – 7.60 (m, 3H, phenyl-C***H***), 2.67 (dsept, $^{3}J_{PH}$ = 8.8, $^{3}J_{HH}$ = 6.8 Hz, 2H, ^{i}Pr-C***H***), 2.47 (s, 6H, thiophene-C***H***$_3$), 1.35 (dd, $^{3}J_{PH}$ = 18.5, $^{3}J_{HH}$ = 6.9 Hz, 6H, ^{i}Pr-C***H***$_3$), 1.16 (dd, $^{3}J_{PH}$ = 17.3, $^{3}J_{HH}$ = 7.0 Hz, 6H, ^{i}Pr-C***H***$_3$).

^{19}F NMR (377 MHz, CD_2Cl_2) δ -56.5 (t, *J* = 21.4 Hz, 6F, C***F***$_3$), -134.9 – -140.4 (m, 4F, C***F***), -140.9 – -144.7 (m, 4F, C***F***).

^{13}C NMR (151 MHz, CD_2Cl_2) δ 145.0 (dm, J_{CF} = 258.1 Hz, ***C***F), 144.1 (dm, J_{CF} = 251.6 Hz, ***C***F), 143.9 (t, J_{CF} = 4.6 Hz, ***C***$_q$), 136.9 (s, ***C***$_q$), 135.5 (d, J_{CP} = 13.8 Hz, phenyl-***C***H), 133.3 (t, J_{CF} = 6.5 Hz, thiophene-***C***H), 132.6 (s, phenyl-***C***H), 131.8 (d, J_{CP} = 53.0 Hz, phenyl-***C***P), 131.2 (d, J_{CP} = 2.7 Hz, phenyl-***C***H), 130.6 (d, J_{CP} = 8.0 Hz, phenyl-***C***H), 128.3 (s, ***C***$_q$), 123.4 (s, ***C***$_q$), 121.4 (q, J_{CF} = 274.0 Hz, ***C***F$_3$), 118.7 (t, J_{CF} = 14.1 Hz, ***C***$_q$), 108.2 – 106.1 (m, ***C***-CF$_3$), 26.4 (d, J_{CP} = 34.0 Hz, ^{i}Pr-***C***H), 19.7 (d, J_{CP} = 4.2 Hz, ^{i}Pr-***C***H$_3$), 19.3 (s, ^{i}Pr-***C***H$_3$), 14.9 (s, thiophene-***C***H$_3$).

Boron bound carbon atom is not observed.

^{11}B NMR (193 MHz, CD_2Cl_2) δ 32.3.

^{31}P NMR (243 MHz, CD_2Cl_2) δ 58.5.

MS (LIFDI): [$C_{38}H_{26}AuBClF_{14}O_2PS_2$], calcd.: 1118.03, found: 1118.14.

UV-vis (DCM): λ_{max} (ε) = 292 nm (45000 $M^{-1}cm^{-1}$).

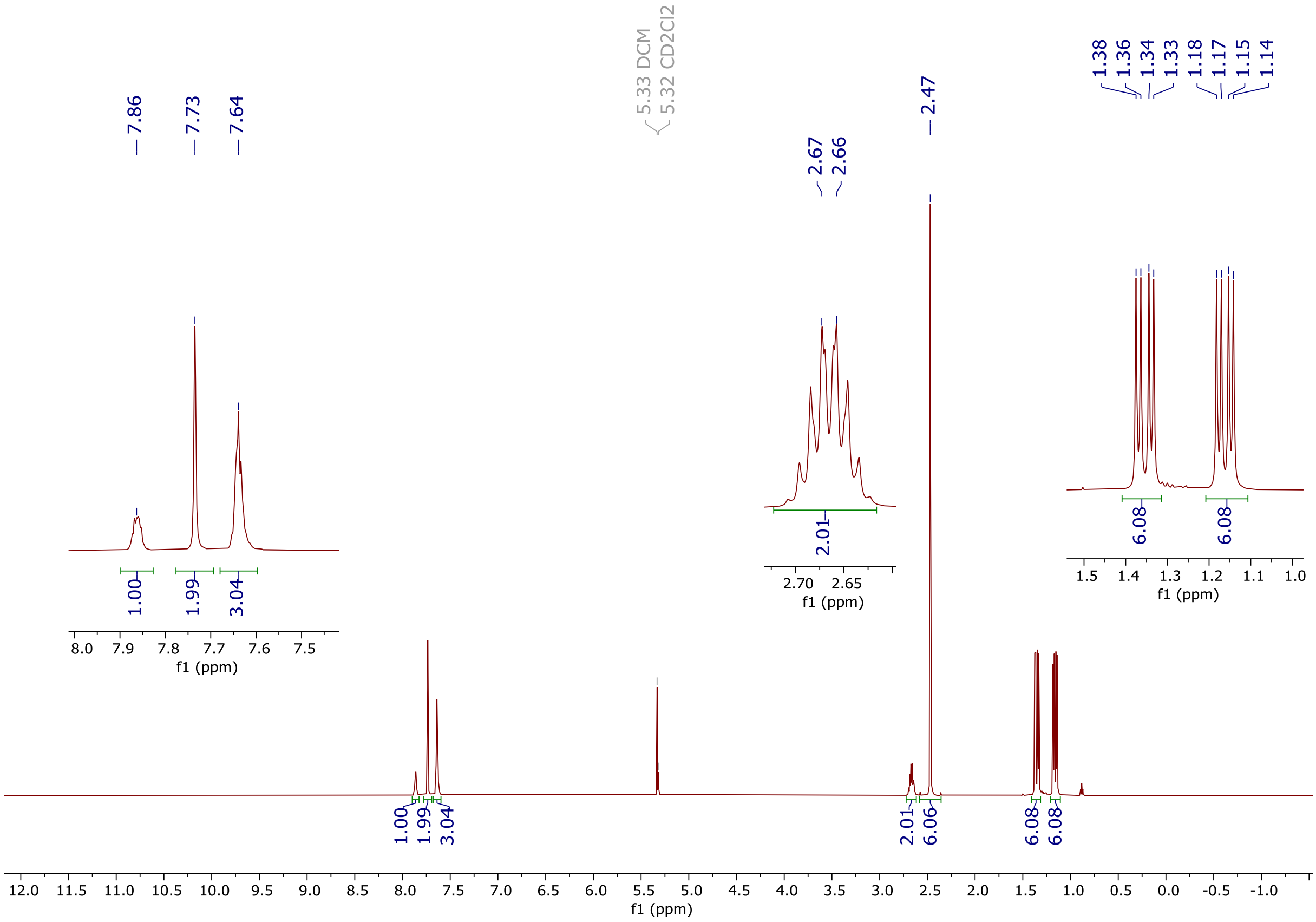

Figure S11. ^{1}H NMR spectrum (600 MHz, CD_2Cl_2) of **2-o-AuCl**.

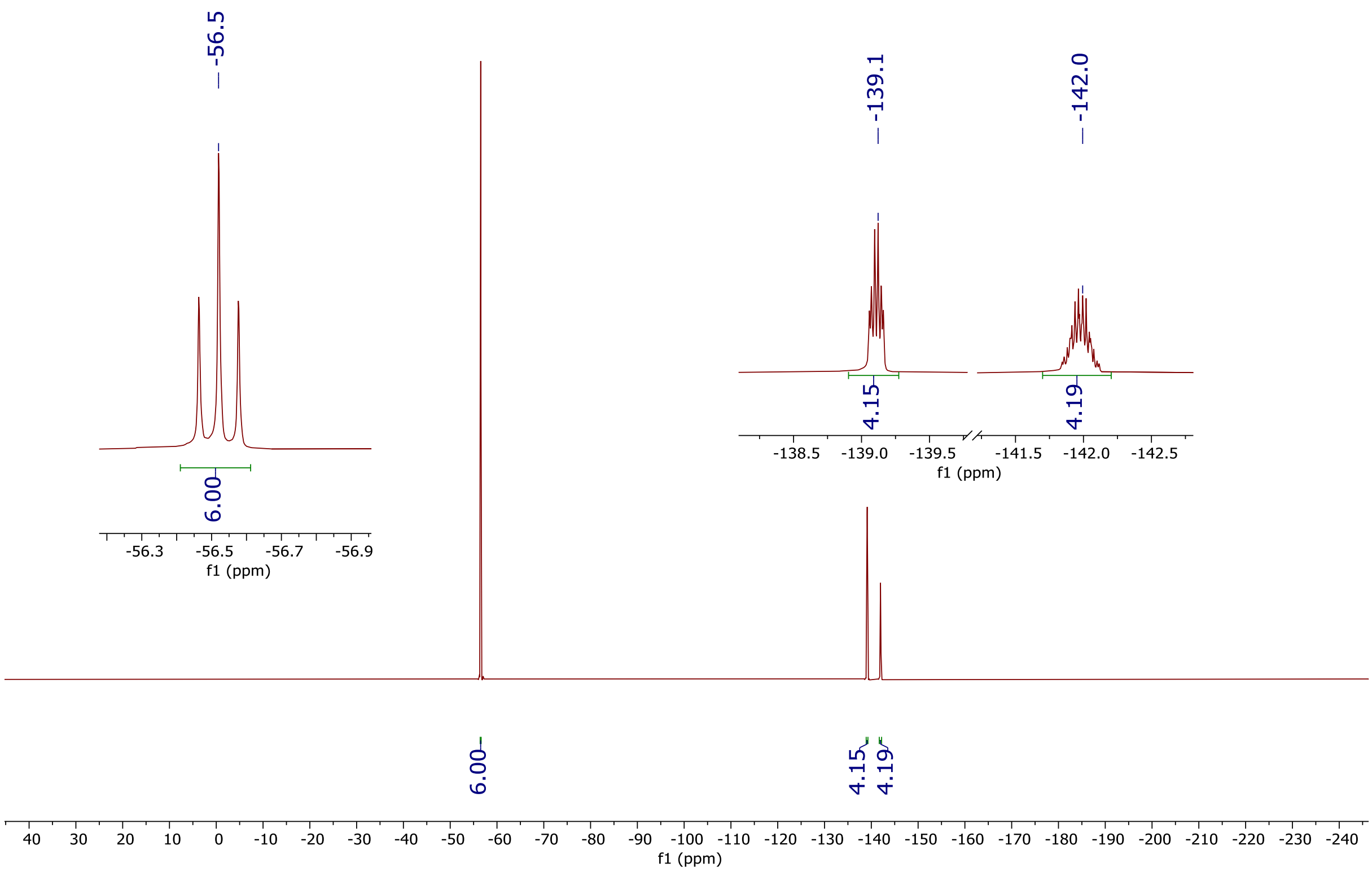

Figure S12. ^{19}F NMR spectrum (377 MHz, CD_2Cl_2) of **2-o-AuCl**.

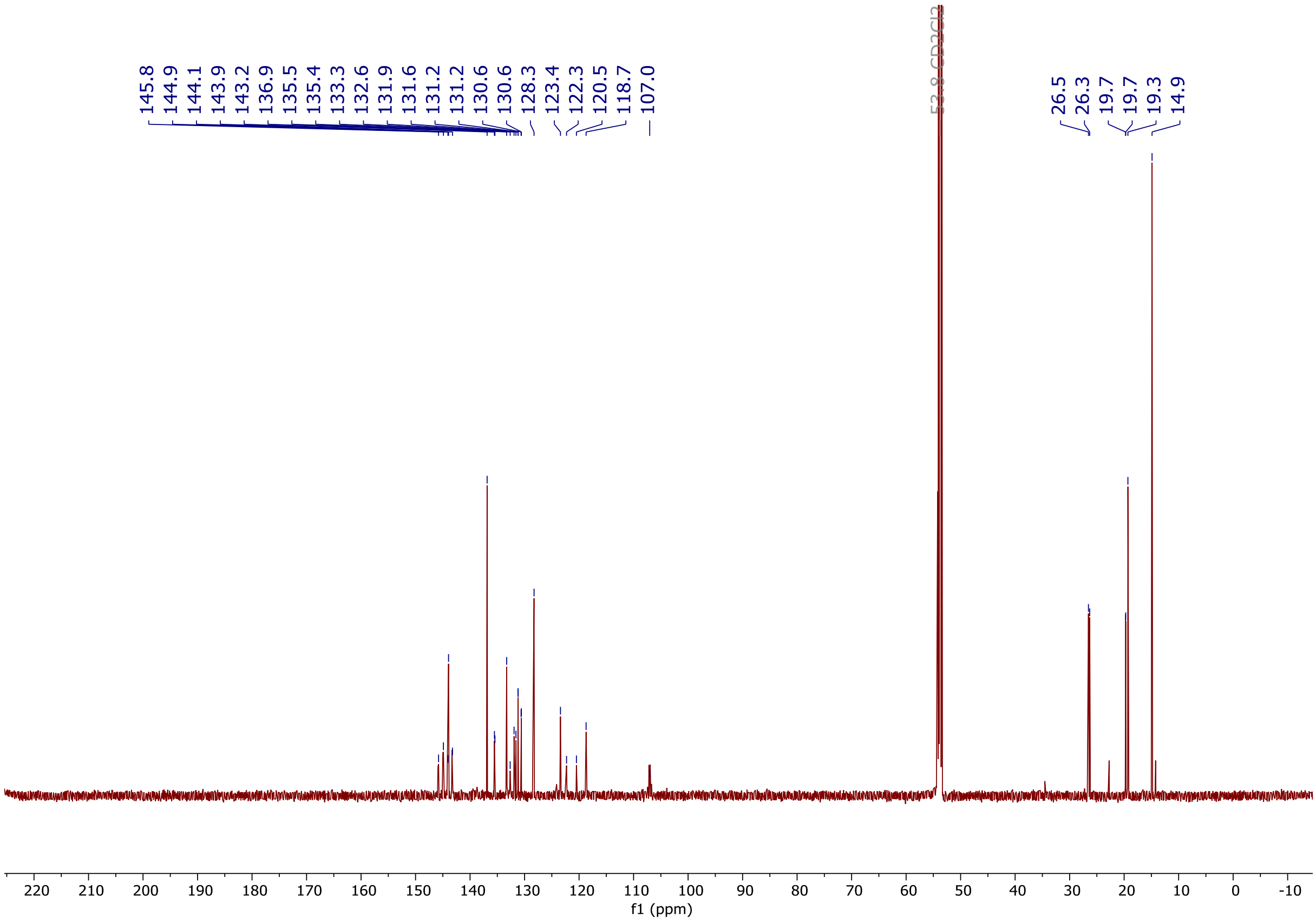

Figure S13. ^{13}C NMR spectrum (151 MHz, CD_2Cl_2) of **2-o-AuCl**.

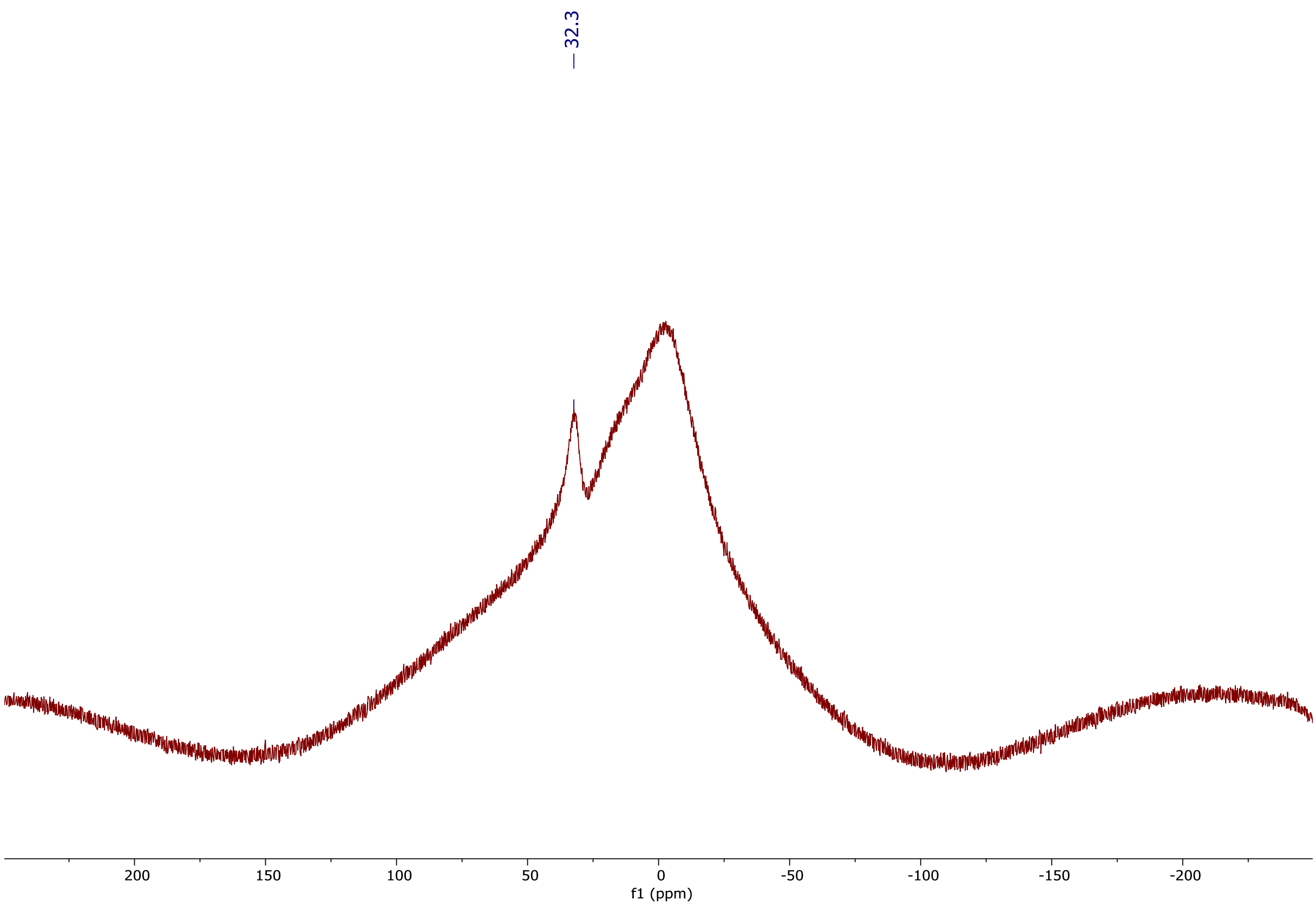

Figure S14. ^{11}B NMR spectrum (193 MHz, CD_2Cl_2) of **2-o-AuCl**.

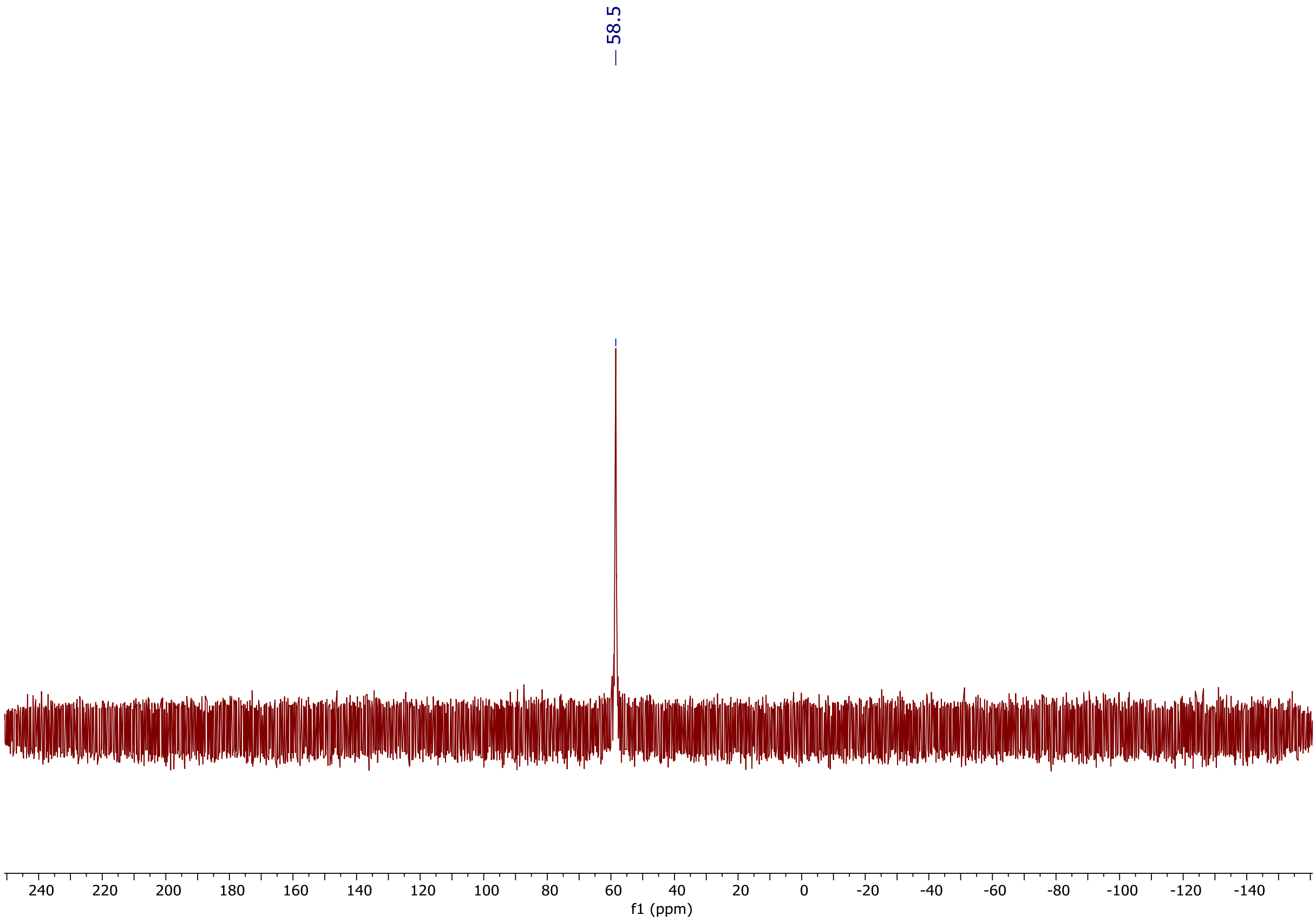

Figure S15. ^{31}P NMR spectrum (243 MHz, CD_2Cl_2) of **2-o-AuCl**.

1.5 2-o-Rh(CNCy)$_2$Cl

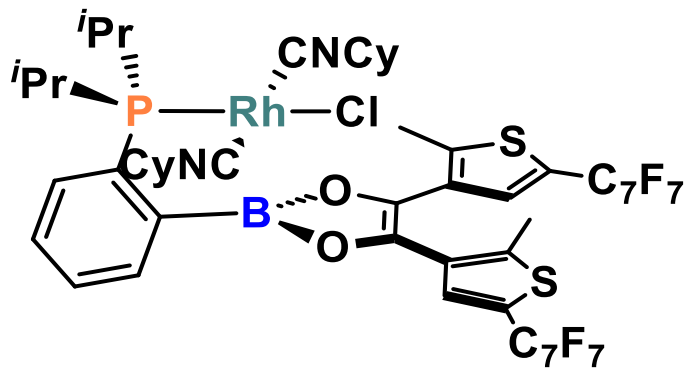

2-o (162 mg, 183 µmol, 1.0 eq) was dissolved in DCM (1 ml). $Rh_2(COD)_2Cl_2$ (45 mg, 90 µmol, 0.5 eq) was added, resulting in a light yellow solution. To this solution, cyclohexyl isonitrile (44 µL, 0.36 mmol 2.0 eq) was added, resulting in a color change to light green. n-hexane (4 ml) was added, and the solution was stored at -40 °C for 2 weeks, resulting in the formation of light green crystals. The solid was separated by decantation, washed with n-hexane, and dried in vacuo (121 mg, 52%) The product is obtained as a mixture of trans/cis isomers in a ratio of 96:4. Crystals suitable for scXRD were obtained by slow evaporation of a DCM/n-hexane solution.

Spectral data for the main trans isomer:

^{1}H NMR (600 MHz, C_6D_6) δ 8.29 (s, 2H, thiophene-C***H***), 7.81 (d, $^3J_{HH}$ = 7.3 Hz, 1H, phenyl-C***H***), 7.20 (t, $^3J_{HH}$ = 7.0 Hz, 1H, phenyl-C***H***), 7.15 – 7.12 (m, 1H, phenyl-C***H***), 7.12 – 7.08 (m, 1H, phenyl-C***H***), 3.04 – 2.88 (m, 2H, cHex-C***H***), 2.55 (s, 6H, thiophene-C***H***$_3$), 2.30 (dsept, $^3J_{HH}$ = 7.0 Hz, $^2J_{PH}$ = 7.0 Hz, 2H, iPr-C***H***), 1.39 (dd, $^3J_{PH}$ = 16.2 Hz, $^3J_{HH}$ = 7.0 Hz, 6H, iPr-C***H***$_3$), 1.31 – 1.15 (m, cHex-C***H***$_2$), 1.09 (dd, $^3J_{PH}$ = 13.9 Hz, $^3J_{HH}$ =7.0 Hz, 6H, iPr-C***H***$_3$), 1.03 – 0.99 (m, cHex-C***H***$_2$), 0.89 – 0.76 (m, cHex-C***H***$_2$).

Note: No integral values for cyclohexyl resonances are given due to signal overlap with iPr-CH_3 and residual n-hexane signals.

^{19}F NMR (377 MHz, C_6D_6) δ -56.0 (t, *J* = 21.2 Hz, 6F, C***F***$_3$), -133.8 – -140.6 (m, 4F, C***F***), -140.9 – -143.5 (m, 4F, C***F***).

^{13}C NMR (151 MHz, CD_2Cl_2) δ 146.4 (br d, J_{RhC} ~ 60 Hz, Rh-***C***NR), 145.0 (dm, J_{CF} = 256.9 Hz, ***C***F), 144.1 (dm, J_{CF} = 251.0 Hz, ***C***F) 142.6 (t, J_{CF} = 4.2 Hz, ***C***$_q$), 139.1 (d, J_{CP} = 39.3 Hz, ***C***$_q$), 136.0 (s, ***C***$_q$), 134.2 (t, J_{CF} = 6.2 Hz, thiophene-***C***H) 133.5 (d, J_{CP} = 15.5 Hz, phenyl-***C***H), 130.7 (s, ***C***$_q$), 130.0 (s, phenyl-***C***H), 129.1 (d, J_{CP} = 2.2 Hz, phenyl-***C***H), 127.6 (d, J_{CP} = 5.8 Hz, phenyl-***C***H), 122.5 (s, ***C***$_q$), 121.4 (d, J_{CF} = 273.9 Hz, ***C***F$_3$) 119.1 (t, J_{CF} = 14.3 Hz, ***C***$_q$), 108.3 – 105.6 (m, ***C***-CF$_3$), 54.1 (s, cHex-***C***H), 32.6 (s, cHex-***C***H$_2$), 26.6 (d, J_{CP} = 27.4 Hz, iPr-***C***H), 25.2 (s, cHex-***C***H$_2$), 23.6 (s, cHex-***C***H$_2$), 19.5 (d, J_{CP} = 4.4 Hz, iPr-***C***H$_3$), 18.9 (s, iPr-***C***H$_3$), 14.9 (s, thiophene-***C***H$_3$).

^{31}P NMR (243 MHz, C_6D_6) δ 70.5 (d, $^1J_{RhP}$ = 161.9 Hz).

Note: Two signals with 0.04 ppm difference are observed. The integral ratio of 1:3.1 points to isotope effects of $^{35}Cl/^{37}Cl$.

^{11}B NMR (193 MHz, CD_2Cl_2) δ 29.9.

MS (LIFDI): [$C_{52}H_{48}BClF_{14}N_2O_2PRhS_2$], calcd.: 1242.15, found: 1242.30.

UV-vis (Benzene): λ_{max} (ε) = 300 nm (42000 $M^{-1}cm^{-1}$), 382 nm (11000 $M^{-1}cm^{-1}$).

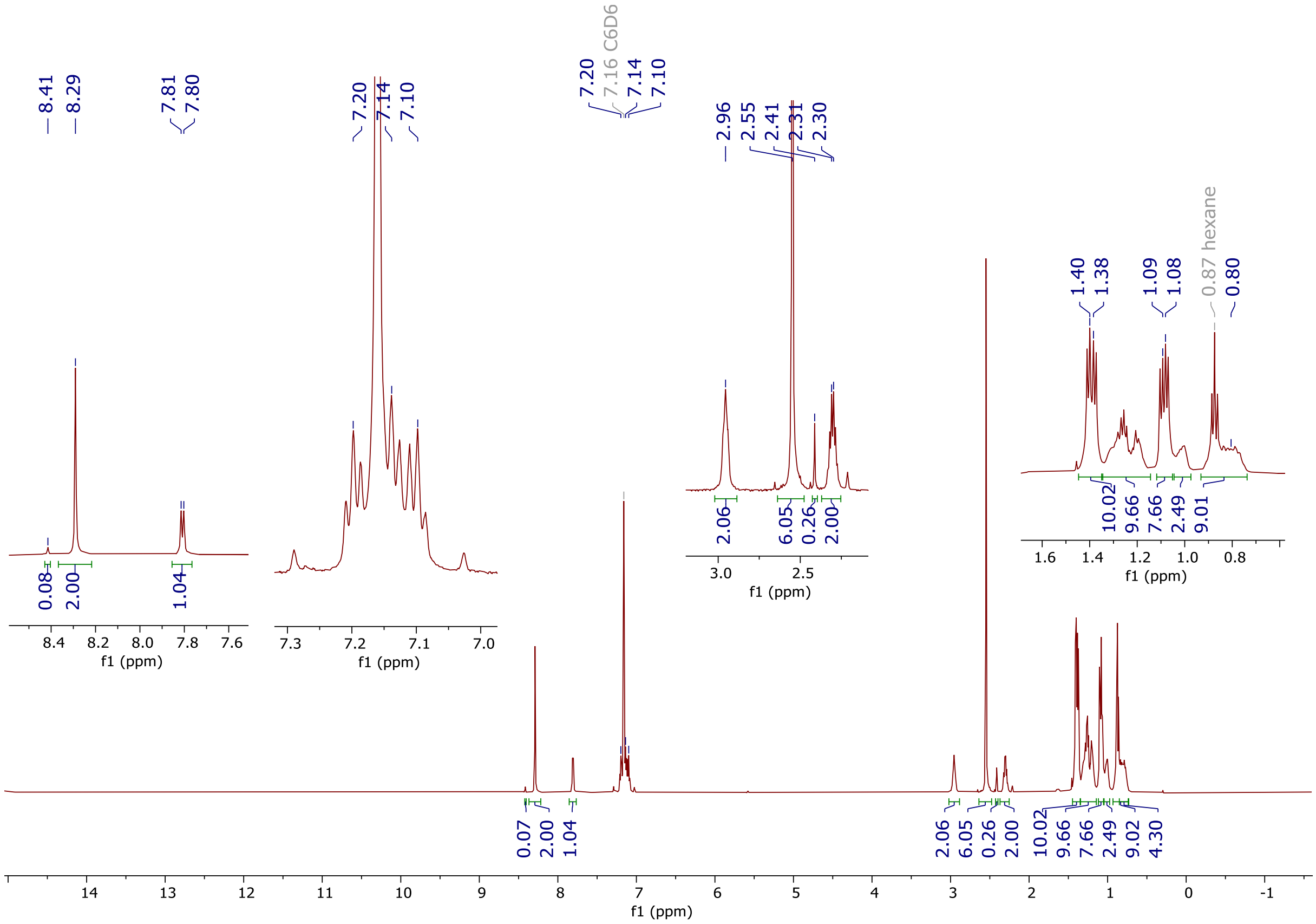

Figure S16. ^{1}H NMR spectrum (600 MHz, C_6D_6) of **2-o-Rh(CNCy)$_2$Cl**.

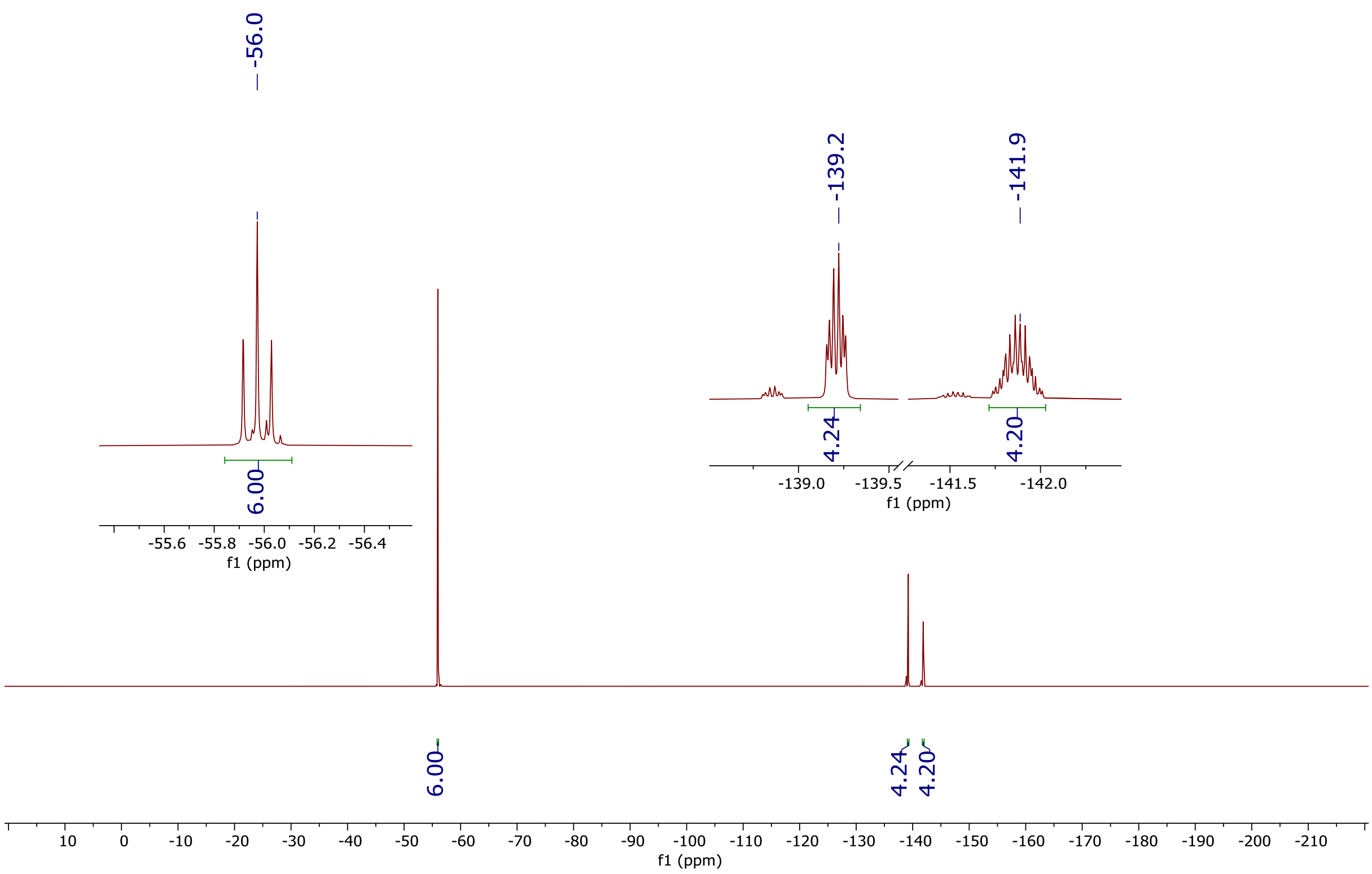

Figure S17. ^{19}F NMR spectrum (377 MHz, C_6D_6) of **2-o-Rh(CNCy)$_2$Cl**.

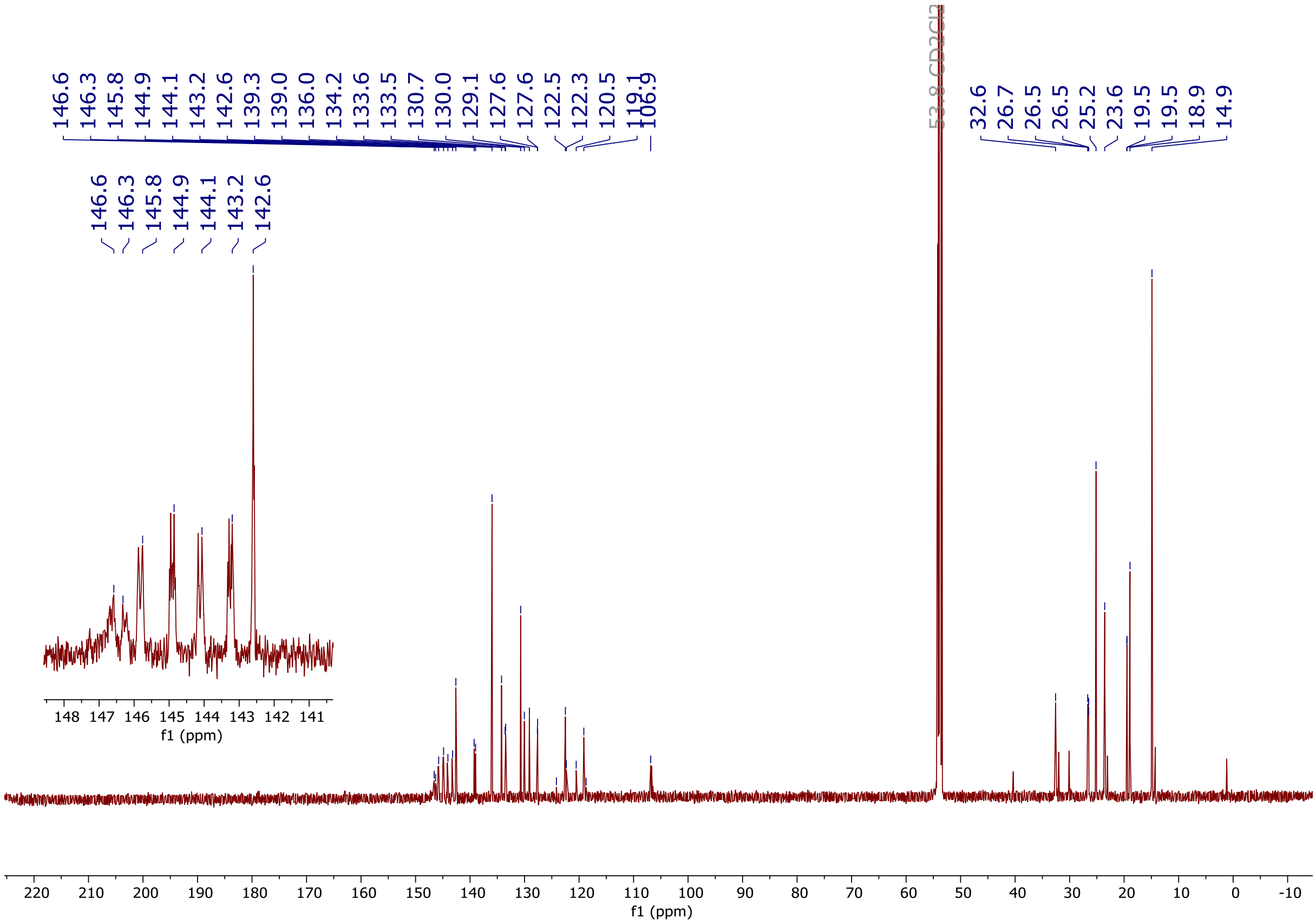

Figure S18. ^{13}C NMR spectrum (151 MHz, CD_2Cl_2) of **2-o-Rh(CNCy)₂Cl**.

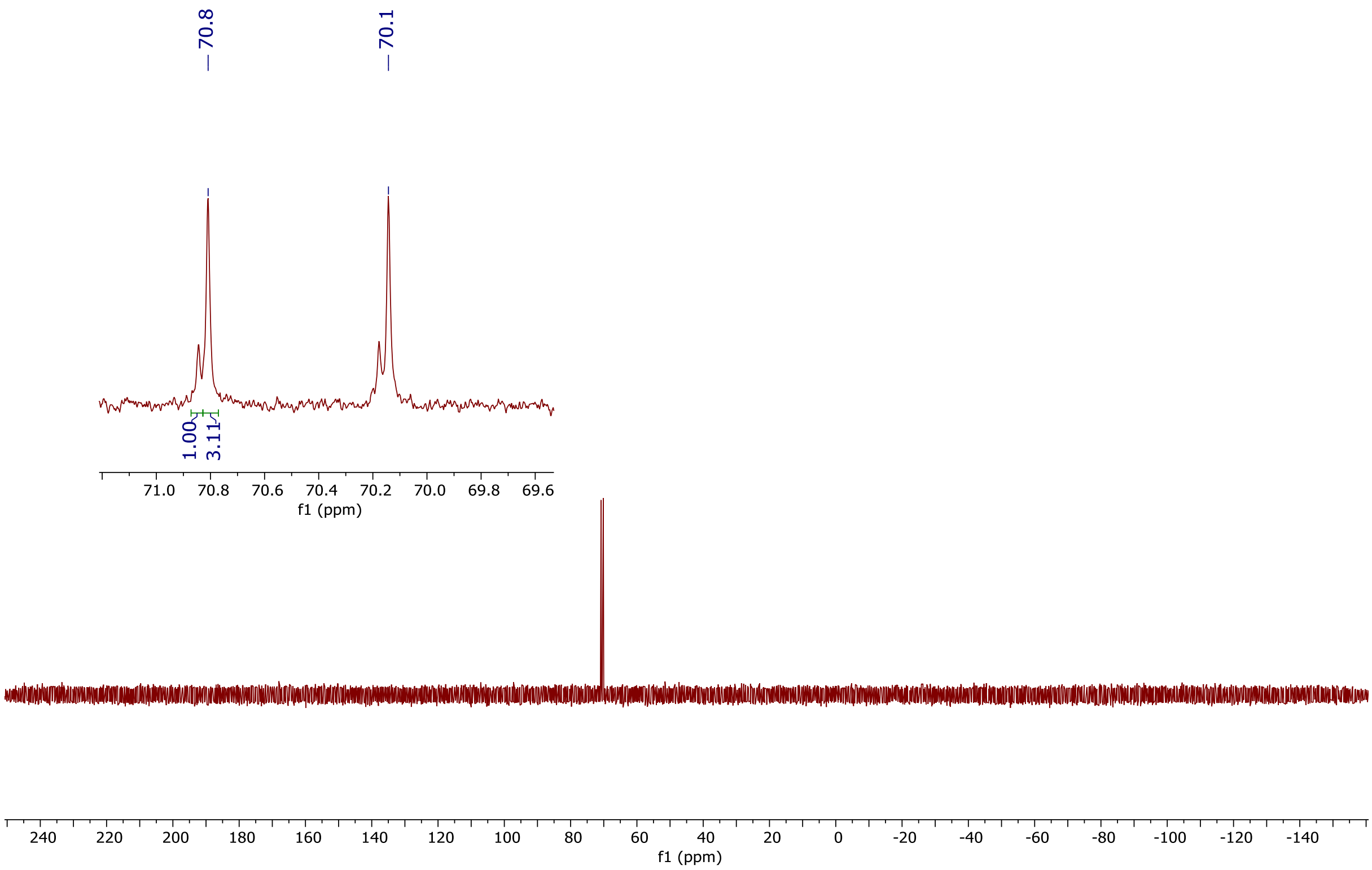

Figure S19. ^{31}P NMR spectrum (243 MHz, C_6D_6) of **2-o-Rh(CNCy)$_2$Cl**.

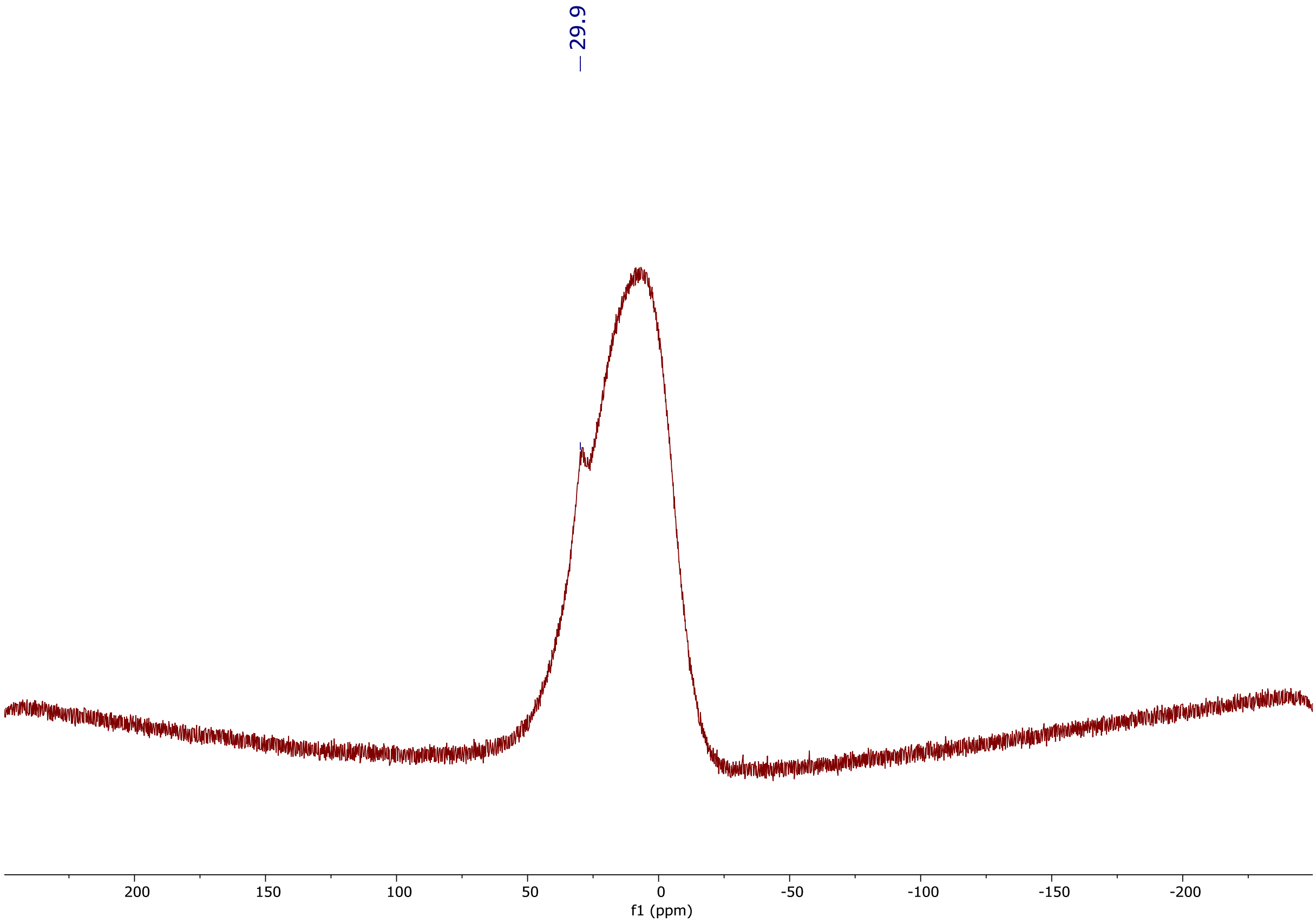

Figure S20. ^{11}B NMR spectrum (193 MHz, CD_2Cl_2) of **2-o-Rh(CNCy)$_2$Cl**.

Comments on the minor isomer of 2-o-Rh(CNCy)$_2$Cl

The compound is obtained with 4% of a minor isomer. After recrystallization, the ratio of this isomer is reduced to 1% (Figure S21). The minor isomer is in equilibrium with the main isomer: Heating the compound in C_6D_6 solution to 60 °C overnight results in a ratio of 3:7 of minor and major product (compared to 4:96 before). Further heating does not change this ratio. After photocyclization and cycloreversion, a similar 3:7 ratio is obtained, suggesting that the formation can be photochemically induced. The minor isomer shows a ^{31}P NMR signal at 47.0 ppm (d, J = 131.5 Hz). Based on the available evidence, cis-trans isomerization is most likely: In the ^{1}H NMR spectrum, two signals for the non-equivalent CNCy CH protons are observed for the minor isomer in CD_2Cl_2 (Figure S22). The lower Rh-P coupling constant of the minor isomer is consistent with a π accepting ligand *trans* to the phosphine. DFT calculations (cf. section 3.4) give low energetic differences between cis and trans isomer (trans favored by 4 kJ/mol), supporting the observed equilibrium.

Other isomerization/decomposition processes were estimated as less likely. Only one new species is formed, making ligand scrambling an unlikely explanation. Reaction with water/oxygen as the cause was ruled out with a control experiment: Exposing the solution to air only results in minor decomposition, but no formation of the minor isomer (Figure S23). The relative stability of both isomers at room temperature contradicts the presence of rotamers or other structural isomers, which are expected to equilibrate quickly at room temperature. Stability of cis and trans isomers of a Rh(I) complex without rapid interconversion at room temperature is unexpected, but might be enabled by the steric congestion of the complex.

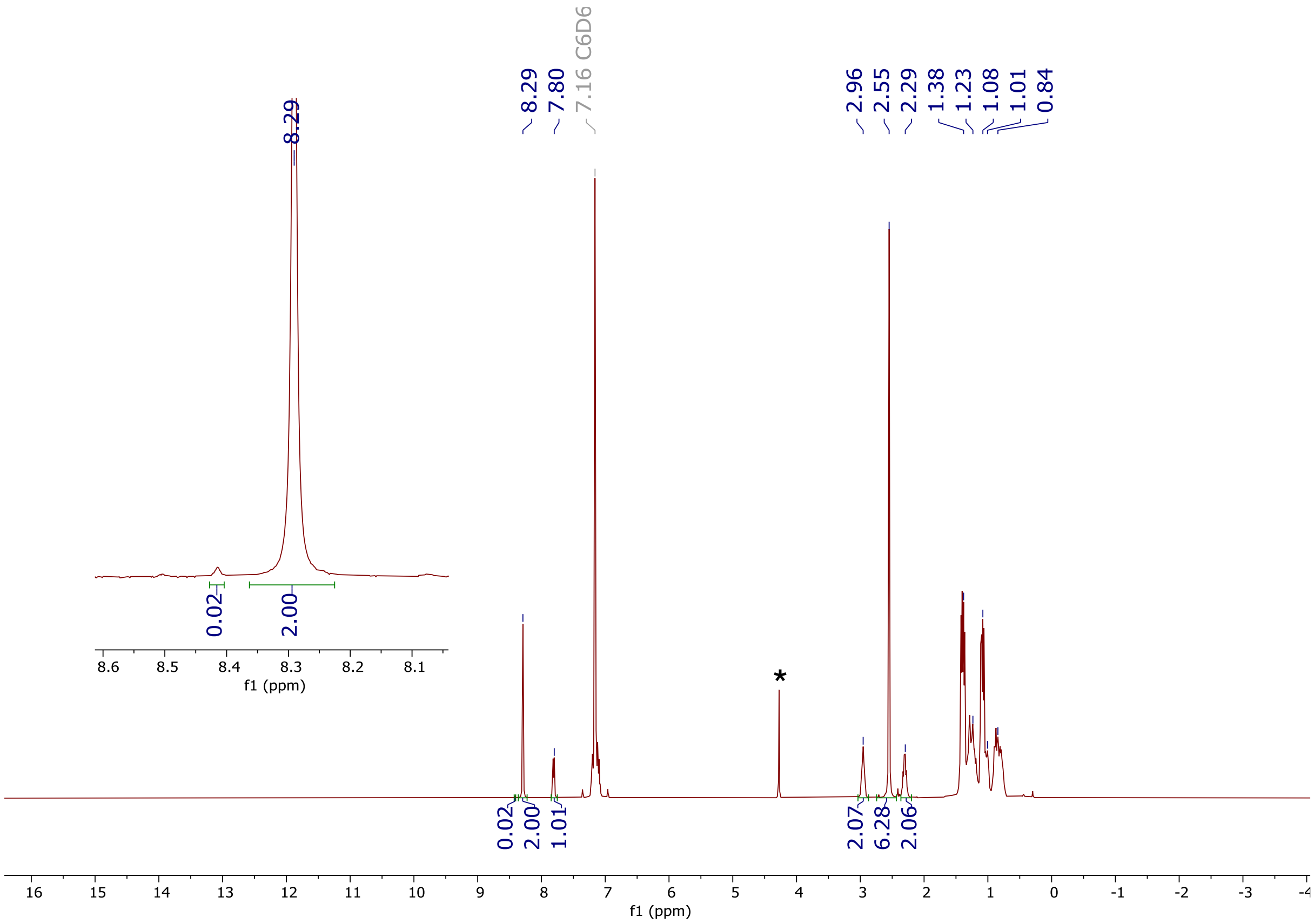

Figure S21. ^{1}H NMR spectrum (400 MHz) of single crystals of **2-o-Rh(CNCy)$_2$Cl** dissolved in C_6D_6 (1% cis). *DCM

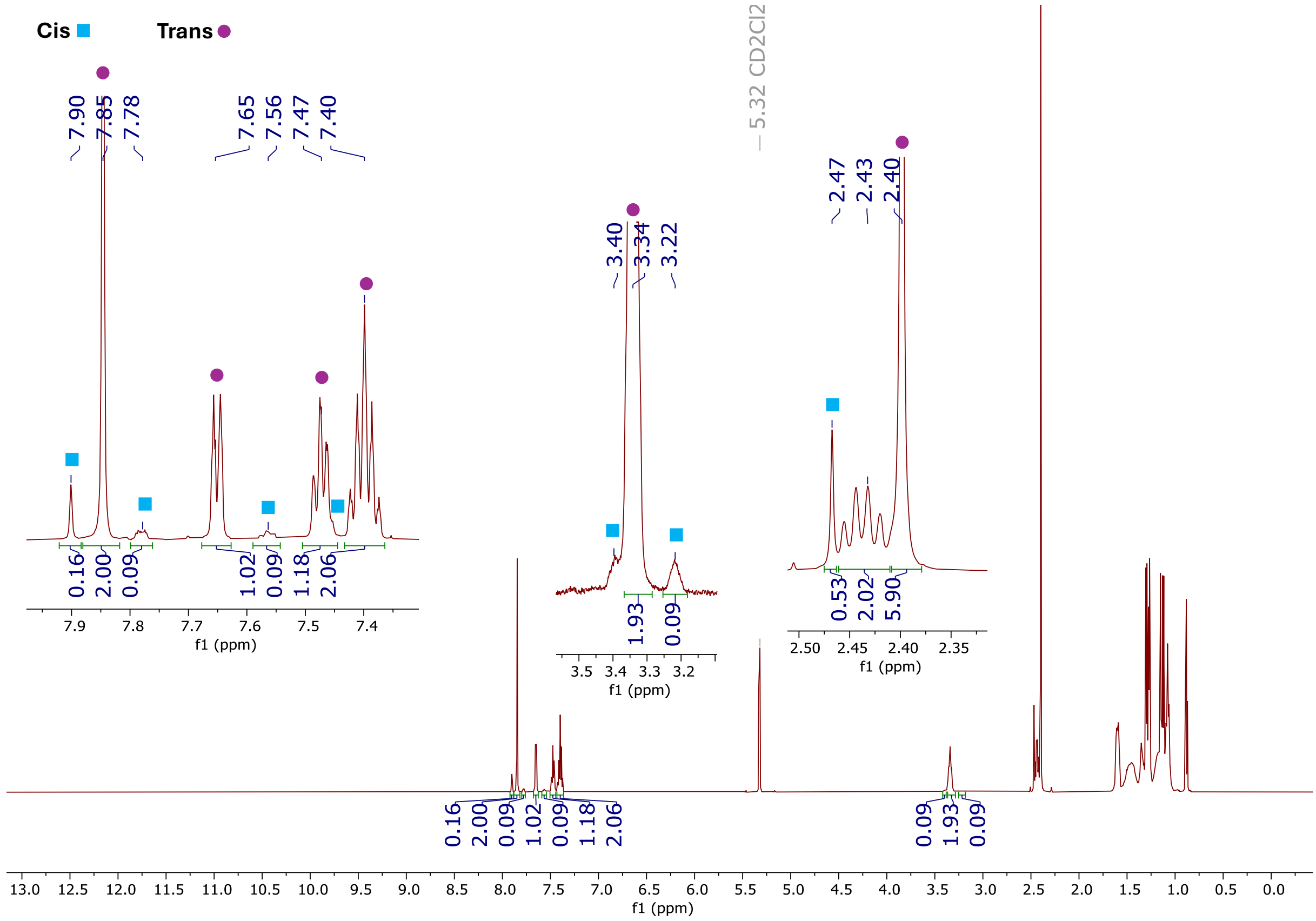

Figure S22. ^{1}H NMR spectrum (400 MHz) of **2-o-Rh(CNCy)$_2$Cl** (8% cis) in CD_2Cl_2. For the cis isomer, inequivalent CH resonances for the cyclohexyl groups are observed at 3.40 and 3.22 ppm.

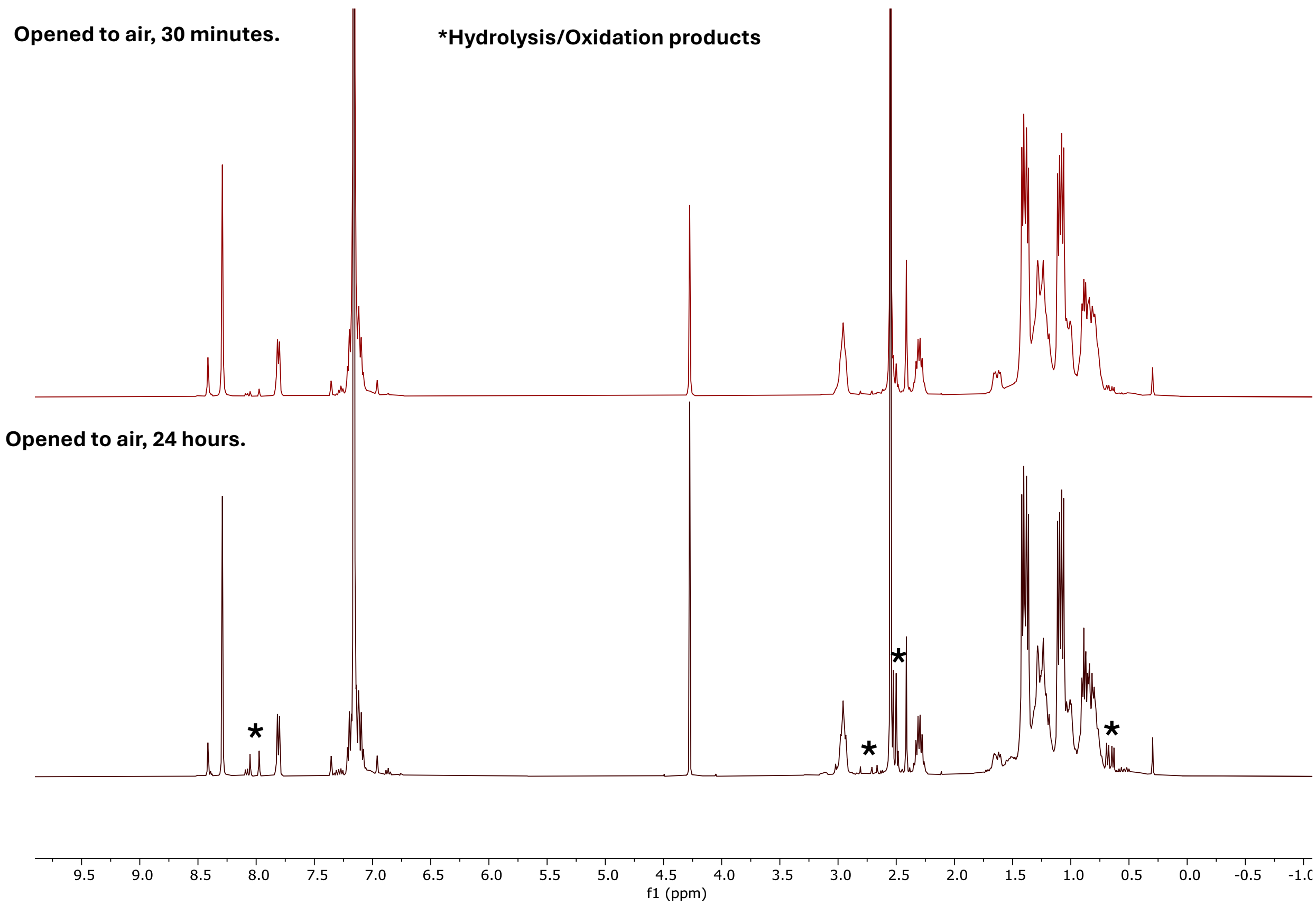

Figure S23. ^{1}H NMR spectrum (400 MHz) of a C_6D_6 solution opened to air after 30 minutes (top) and after 24 hours (bottom).

2 UV-vis spectroscopy

All UV–vis spectra were recorded on a JASCO V-570 UV/VIS/NIR spectrophotometer. Data analysis and plotting was done using OriginLab 2025. For UV irradiation, a MAX-303 light source (Asahi Spectra) was used, consisting of a 300 W xenon arc lamp coupled to a mirror module (250–385 nm) and a 10 nm band-pass filter centered at 300 nm. Visible-light irradiation was performed using commercial white LEDs.

2.1 Extinction Coefficients

1-o

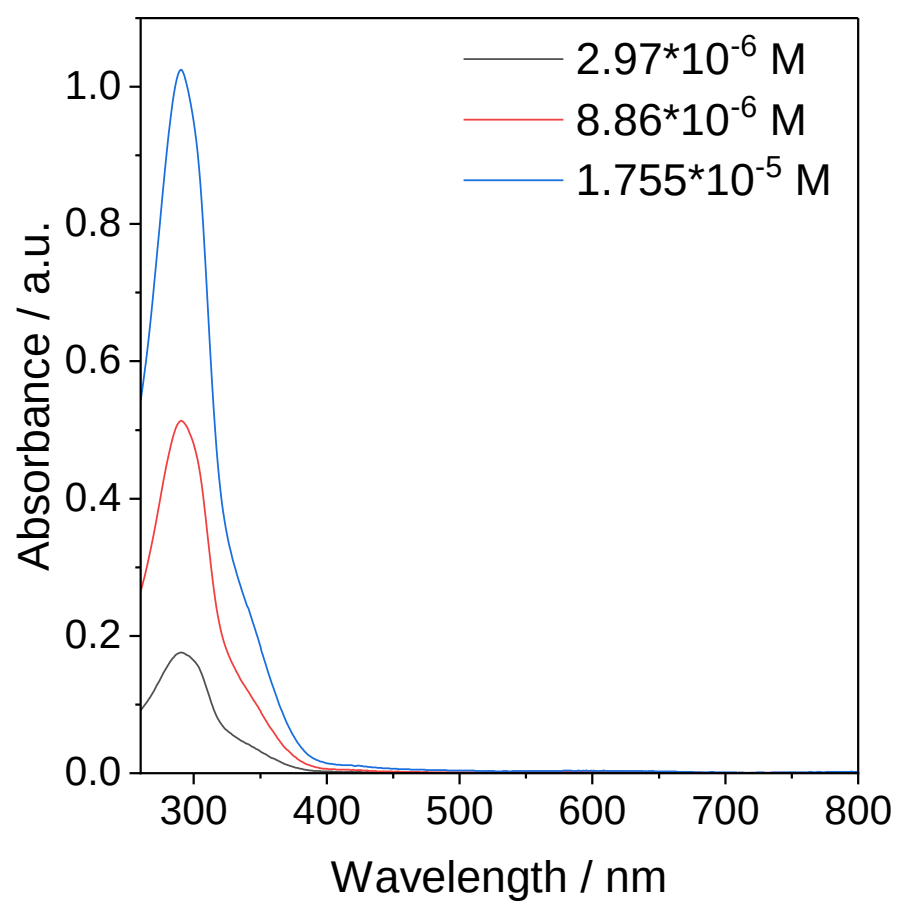

Figure S24. UV-vis spectra of **1-o** in DCM.

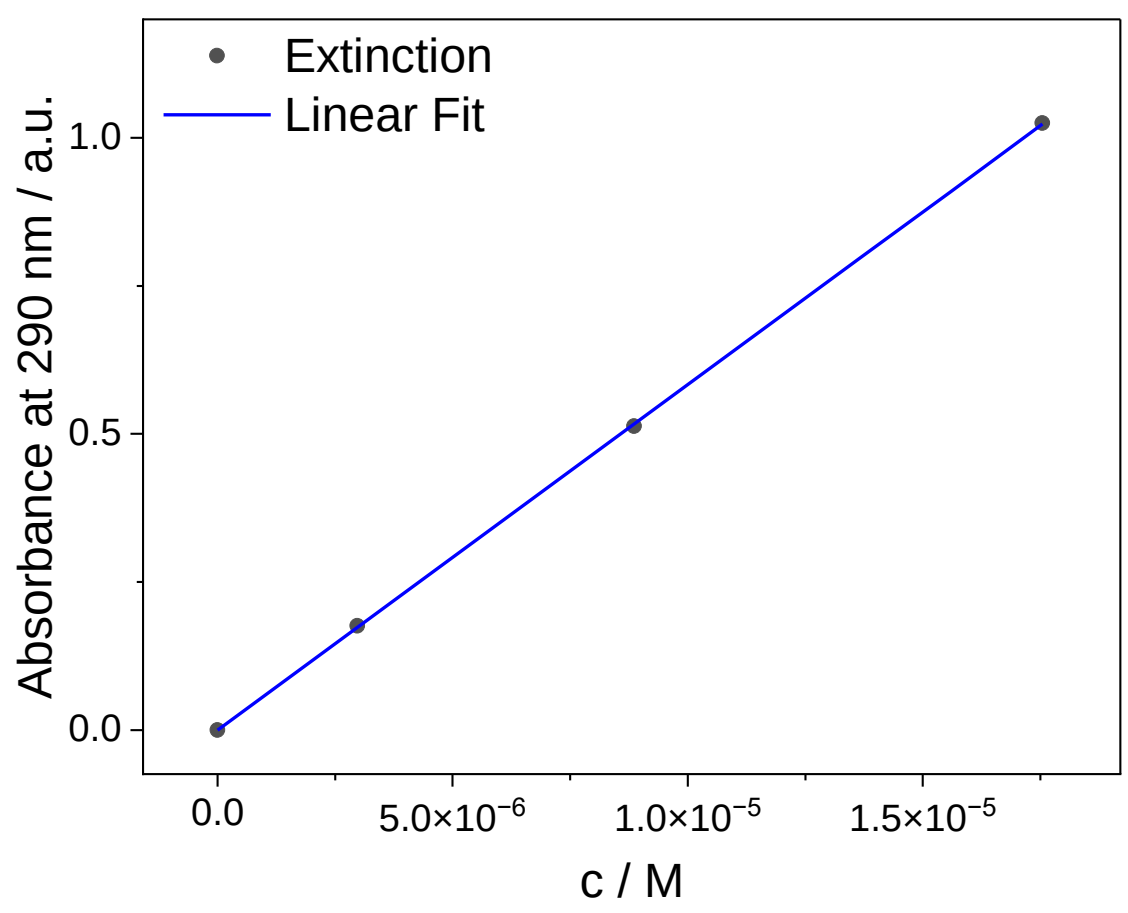

Figure S25. Extinction of **1-o** in DCM at 290 nm. Linear Fit: y = 58325x-0.00005. Slope error: Δε = 232 M^{-1} cm^{-1}.

2-o

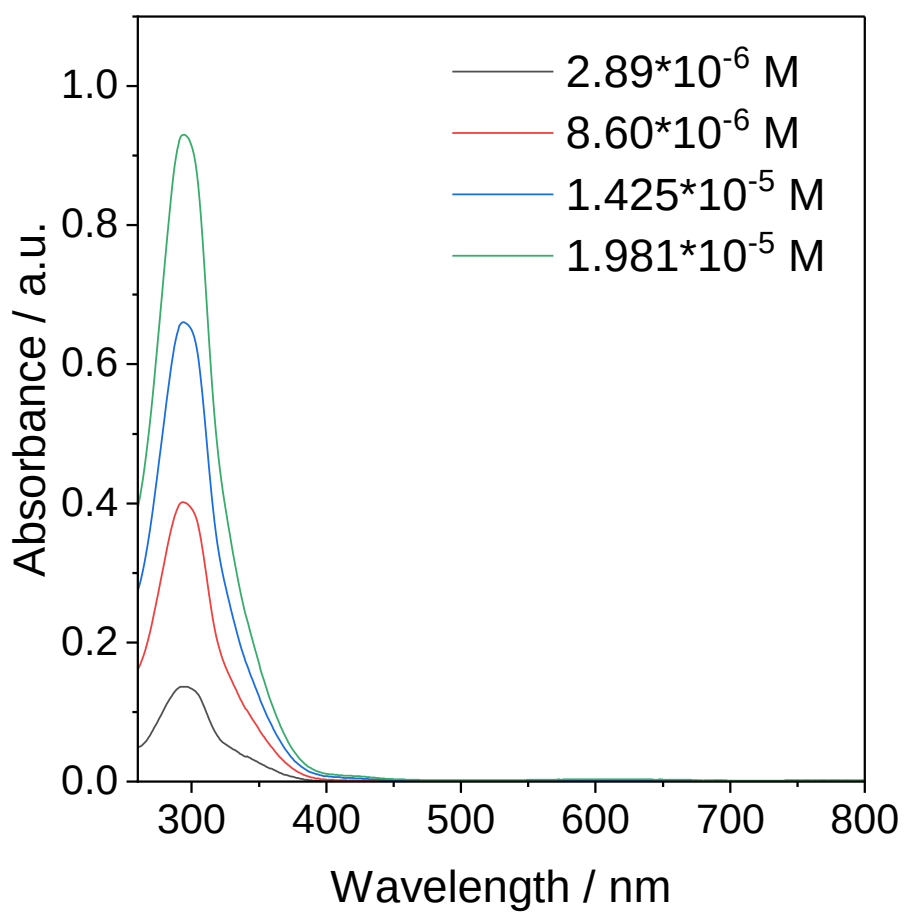

Figure S26. UV-vis spectra of **2-o** in DCM.

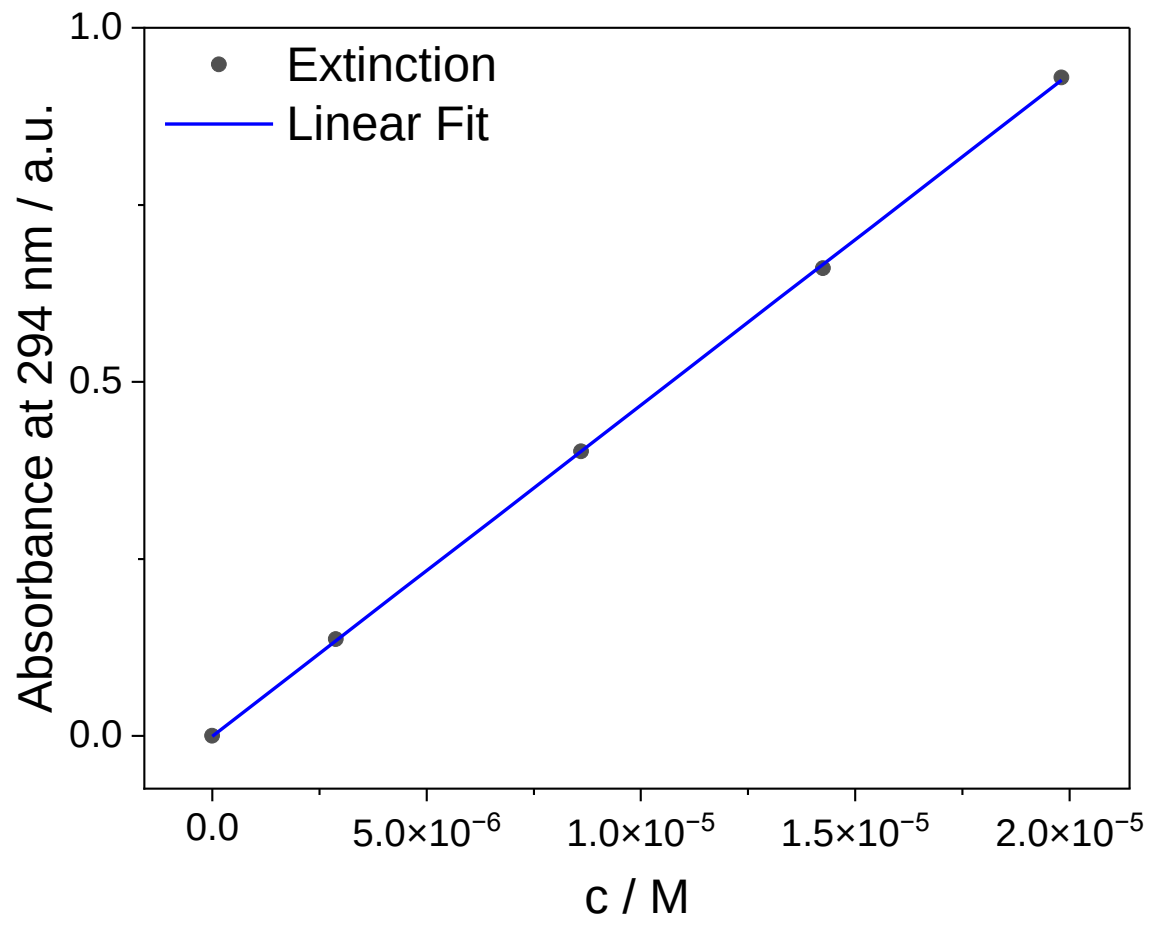

Figure S27. Extinction of **2-o** in DCM at 294 nm. Linear Fit: y = 46752x-0.00021. Slope error: Δε = 247 M^{-1} cm^{-1}.

2-o-AuCl

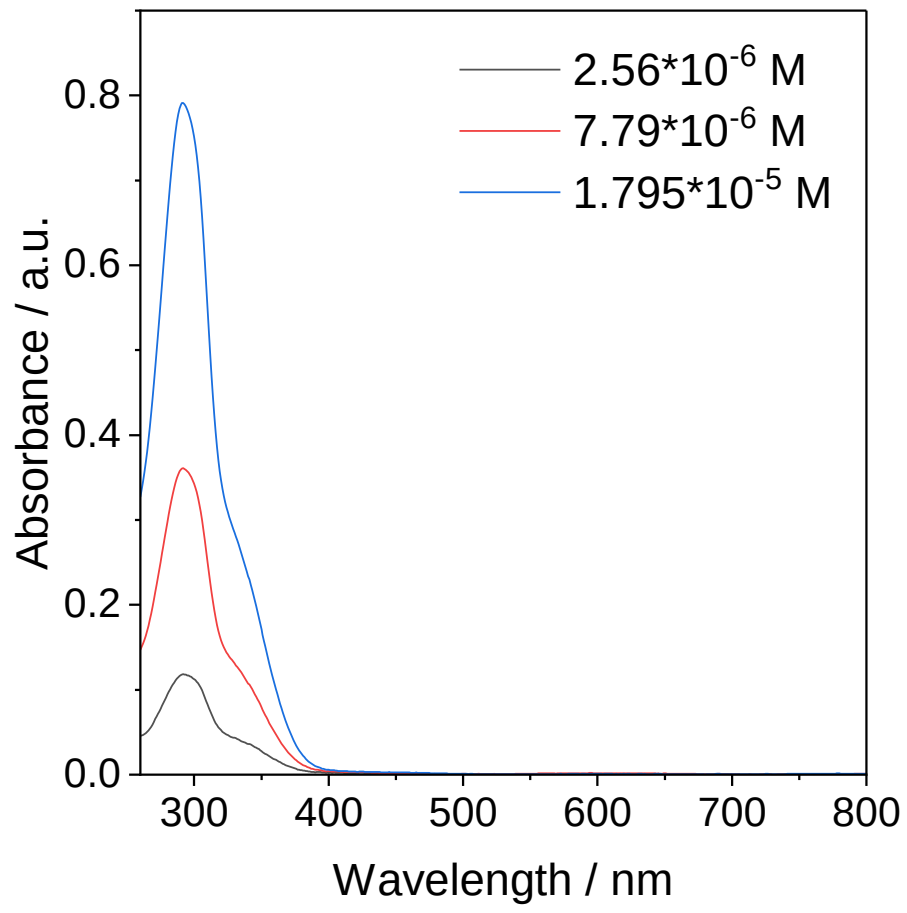

Figure S28. UV-vis spectra of **2-o-AuCl** in DCM.

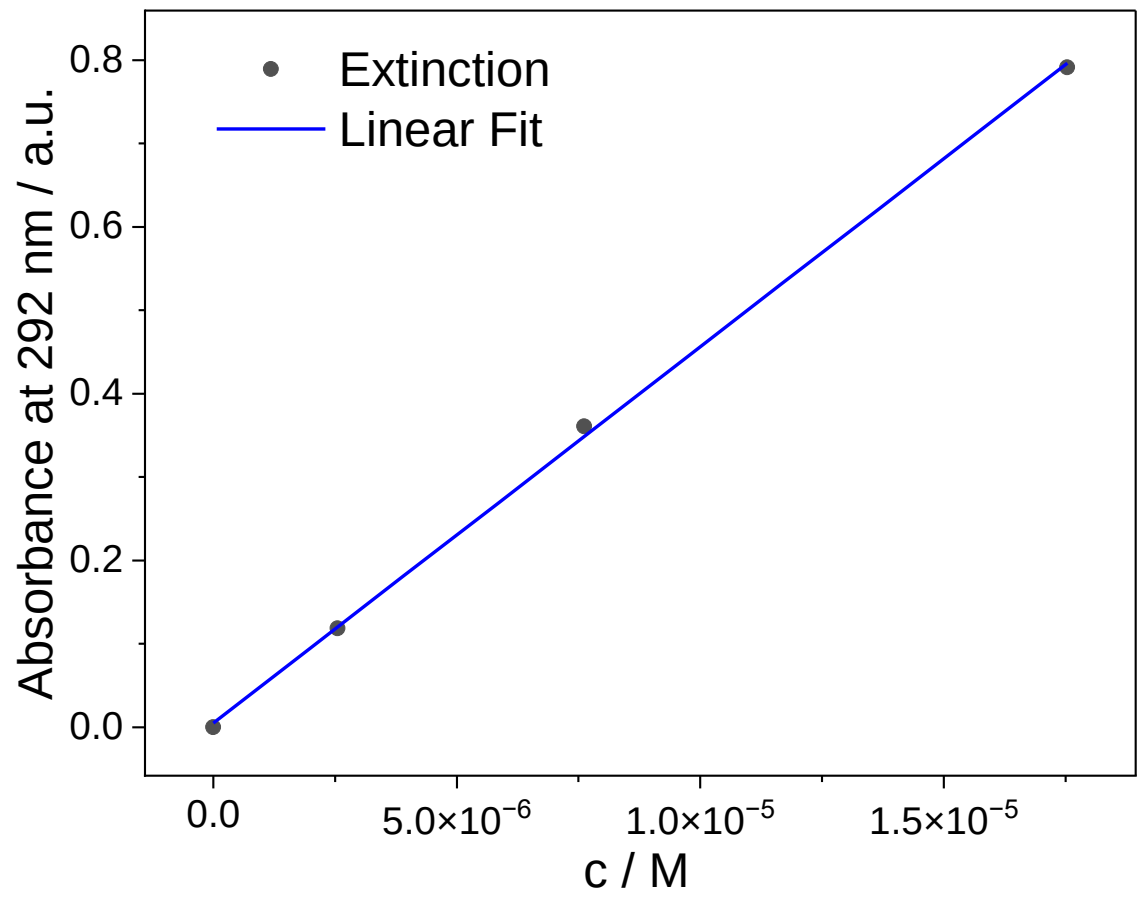

Figure S29. Extinction of **2-o-AuCl** in DCM at 292 nm. Linear Fit: y = 45098x-0.0052. Slope error: Δε = 755 M^{-1} cm^{-1}.

2-o-Rh(CNCy)$_2$Cl

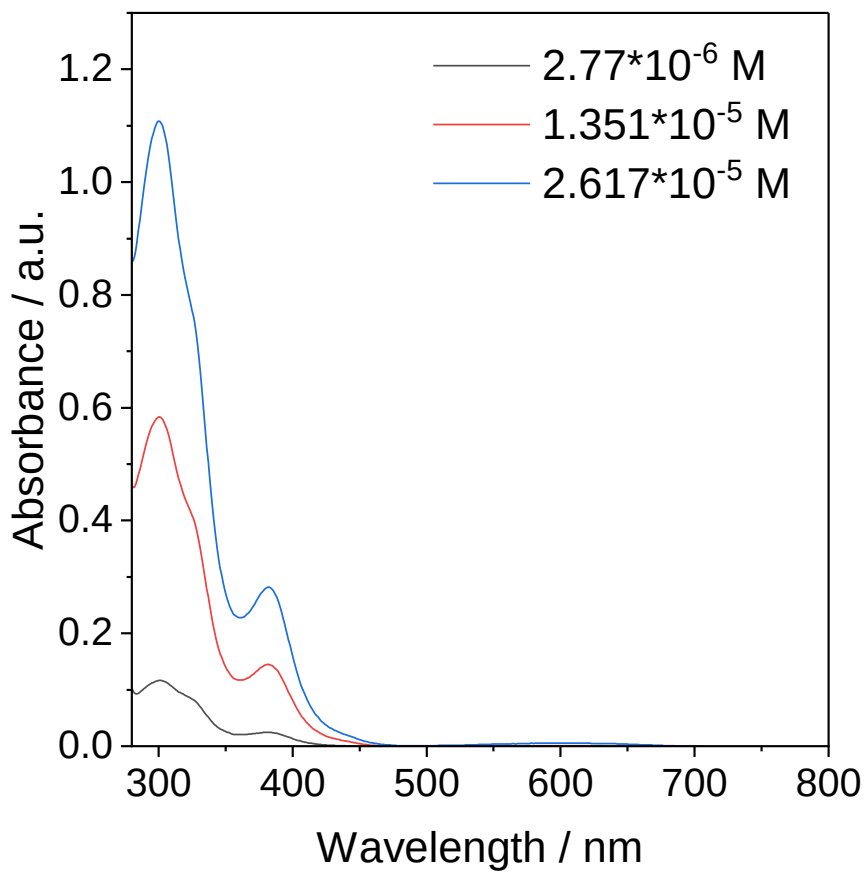

Figure S30. UV-vis spectra of **2-o-Rh(CNCy)$_2$Cl** in benzene.

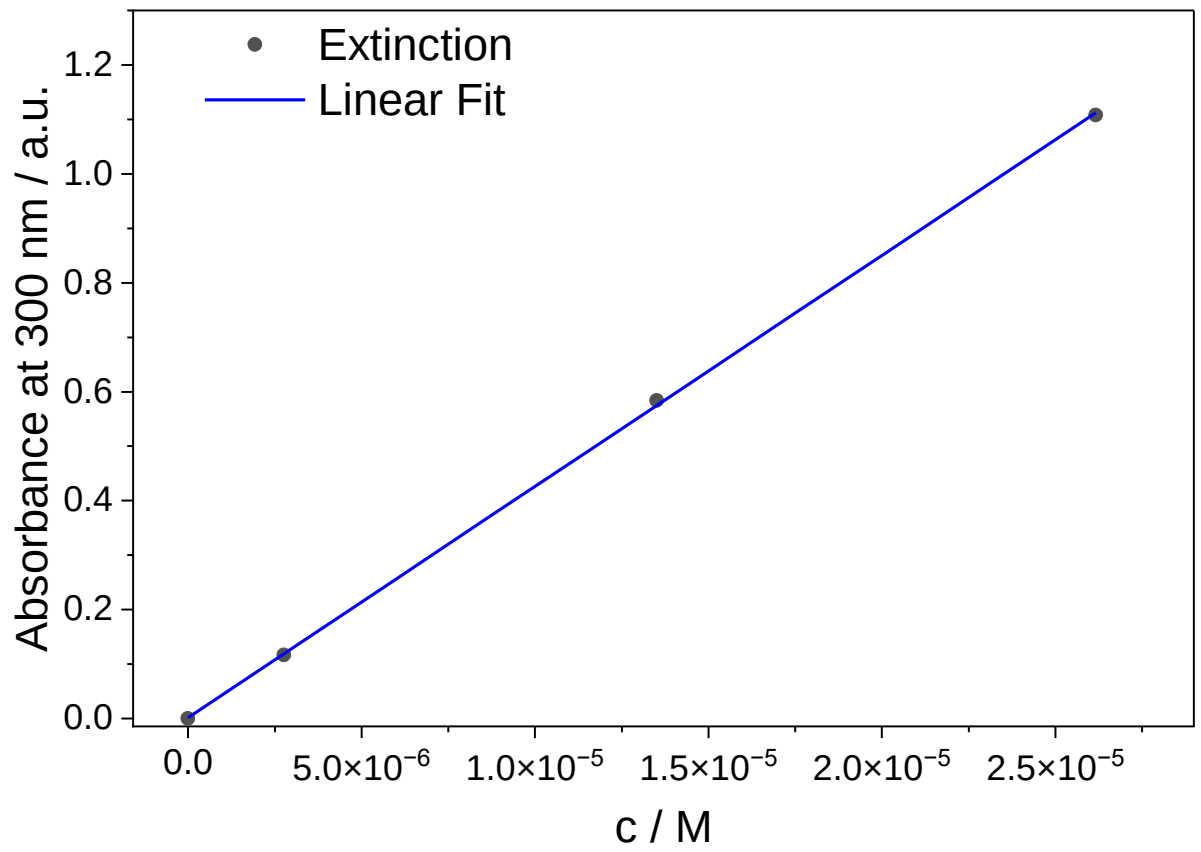

Figure S31. Extinction of **2-o-Rh(CNCy)$_2$Cl** in benzene at 300 nm. Linear Fit: y = 42435x+0.0017. Slope error: Δε = 352 M^{-1} cm^{-1}.

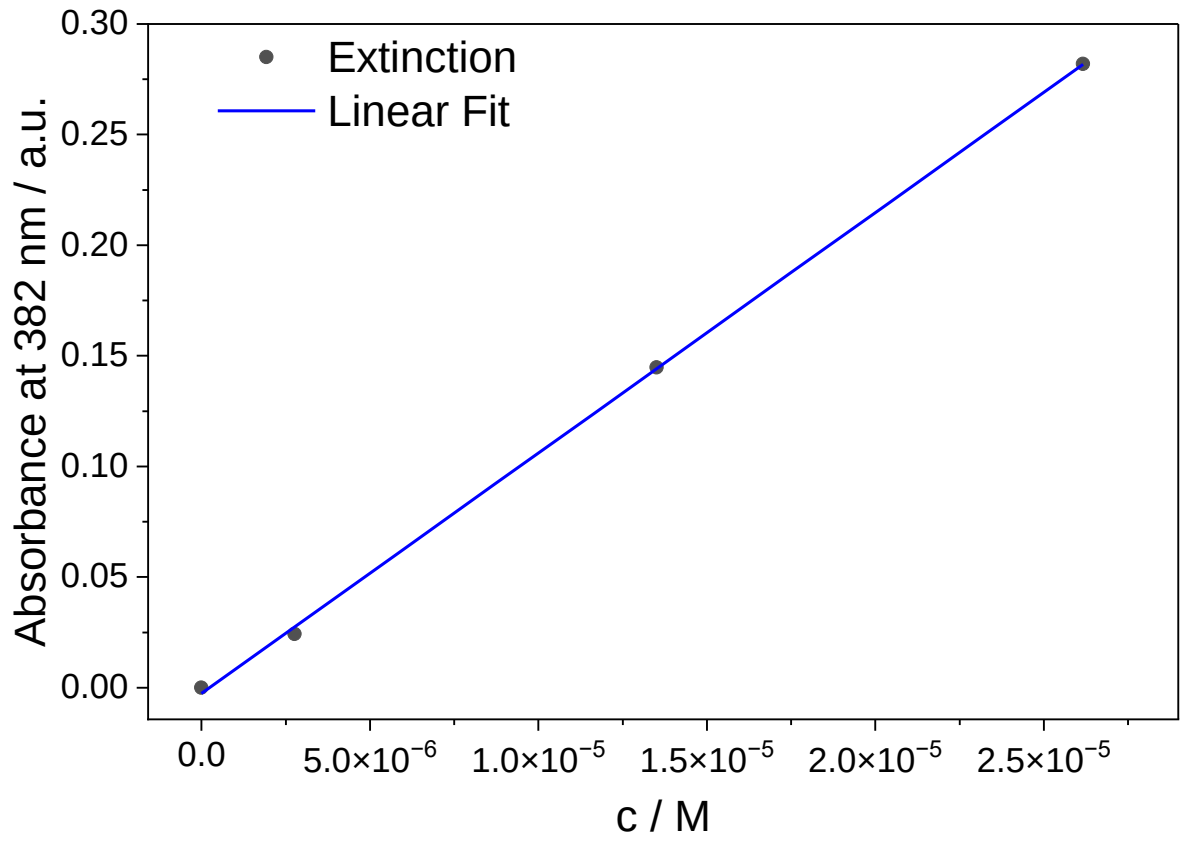

Figure S32. Extinction of **2-o-Rh(CNCy)$_2$Cl** in benzene at 382 nm. Linear Fit: y = 10867x-0.0026. Slope error: Δε = 143 M^{-1} cm^{-1}.

2.2 Photocyclization Quantum Yields

The quantum yields of the ring-closing reactions were determined by UV-vis spectroscopy. The absorbance data was modelled according to the following:

Simplified relation for cyclization and cycloreversion:

$$A \underset{\Phi_B}{\overset{\Phi_A}{\rightleftharpoons}} B$$

Determination of [B] and [A] by absorbance in the visible region (e.g. 550 nm):

$$[B] = \frac{E^{550}}{\varepsilon_B^{550} \cdot l} \quad and \quad [A] = [A]_0 - [B]$$

Differential equation:

$$\frac{d[B]}{dt} = I_o \cdot l \cdot \frac{(1 - 10^{-E^{300}})}{E^{300}} \cdot (\Phi_A \cdot \varepsilon_A^{300}([A]_0 - [B]) - \Phi_B \cdot \varepsilon_B^{300}[B])$$

E^{300}: Total extinction at irradiation wavelength (300 nm), $E^{300} = \varepsilon_A^{300}([A]_o - [B]) + \varepsilon_B^{300}[B]$

E^{550}: Total extinction at vis maximum (e.g. 550 nm)

I_o: Light intensity (determined by ferrioxalate actinometry)

Error of light intensity was estimated from the linear fit of ferrioxalate actinometry (6 data points, 0-50 seconds irradiation

ε_A^{300}: Extinction coefficient of A at 300 nm.

ε_B^{300}: Extinction coefficient of B at 300 nm.

ε_B^{550}: Extinction coefficient of B at vis maximum (e.g. 550 nm)

Errors for the extinction coefficients were estimated by combined measurement errors for the stock solutions and the linear fit error of the dilution series (section 2.1).

l: Cuvette path length (1 cm)

Extinction coefficients of B (closed form) were determined by evaluating the photostationary ratio of A and B by NMR spectroscopy and applying this ratio to the observed absorbance.

Fitting was done using a least-squares method in Python using SciPy.

1 (DCM)

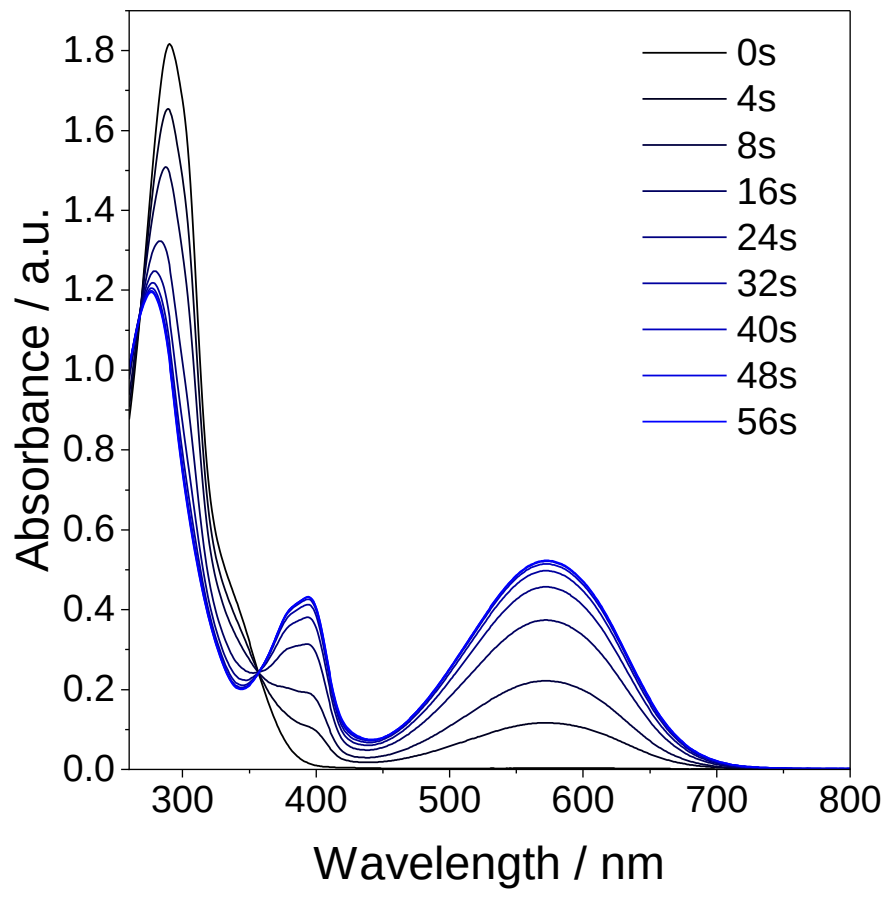

Figure S33. UV-vis spectra of **1** ($3.12 \cdot 10^{-5}\ M$, DCM) during irradiation with UV light (300 nm, 0-56s).

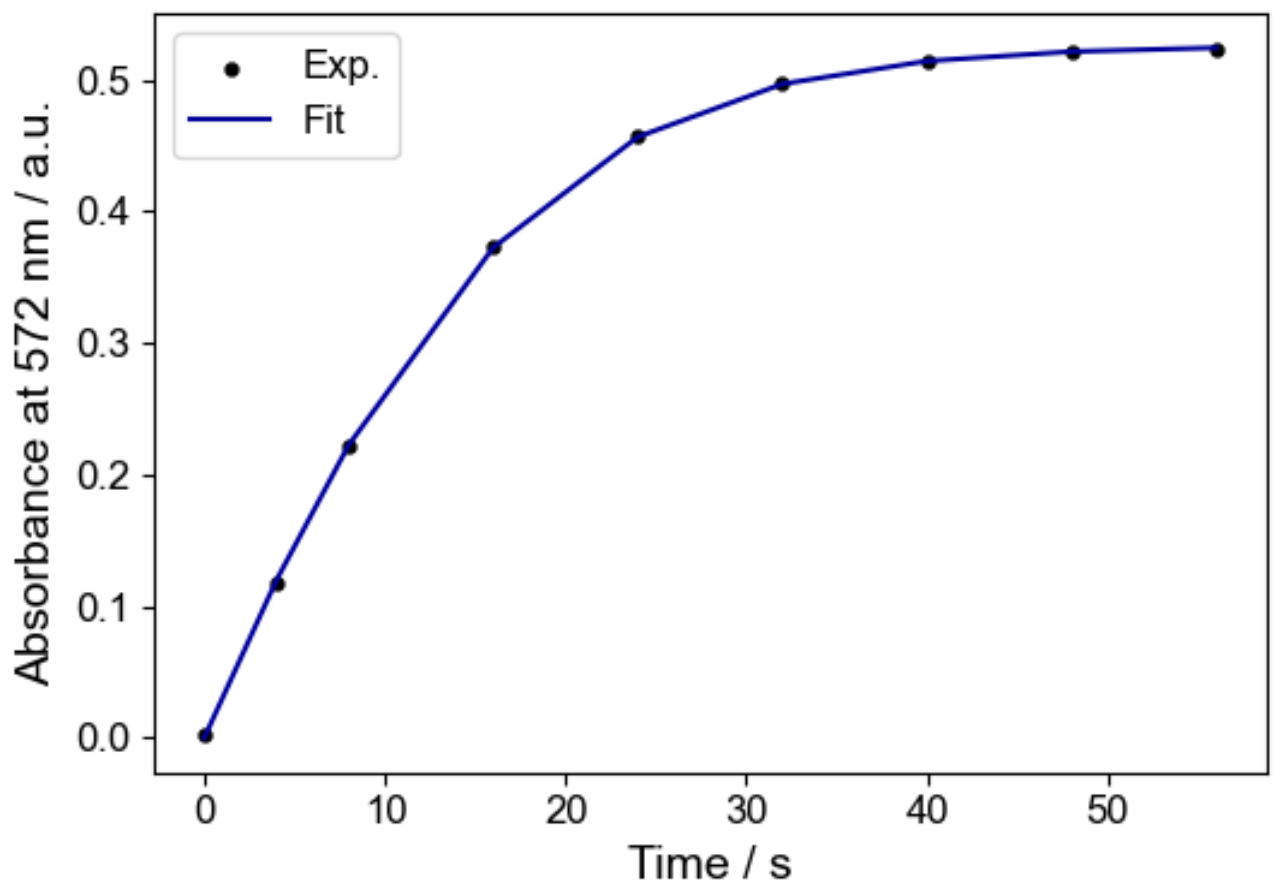

Figure S34. Absorbance of the closed form of **1** (**1-c**) (572 nm) during irradiation with UV light (300 nm, 0–56 s, black dots). Fit by least squares method (blue line).

$c_0 = (3.12 \pm 0.15) \cdot 10^{-5}\ M$
$I_0 = (2.57 \pm 0.06) \cdot 10^{-6}\ E\ L^{-1}\ s^{-1}$
$\varepsilon(open, 300\ \mathrm{nm}) = 54000 \pm 2000\ M^{-1}\ cm^{-1}$
$\varepsilon(closed, 300\ \mathrm{nm}) = 22000 \pm 1000\ M^{-1}\ cm^{-1}$
$\varepsilon(closed, 572\ \mathrm{nm}) = 18000 \pm 1000\ M^{-1}\ cm^{-1}$

$\Phi_{o \to c}(300nm) = \mathbf{0.71 \pm 0.08}$
$\Phi_{c \to o}(300nm) = \mathbf{0.10 \pm 0.11}$

1 (Benzene)

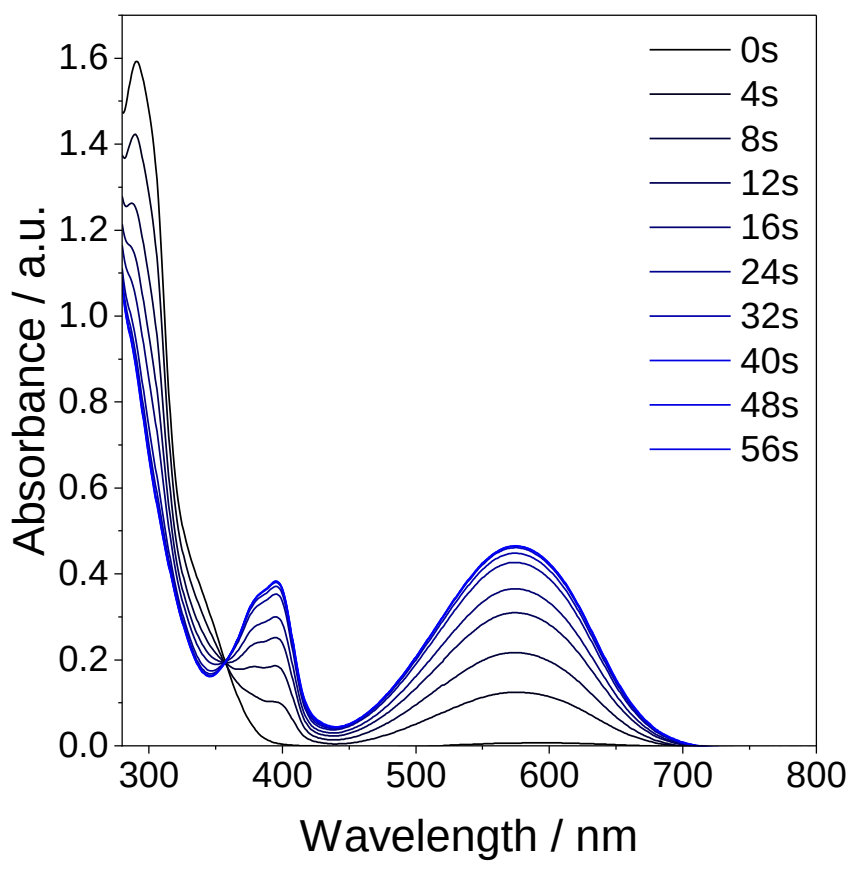

Figure S35. UV-vis spectra of **1** ($2.73 \cdot 10^{-5}\ M$, Benzene) during irradiation with UV light (300 nm, 0-56s).

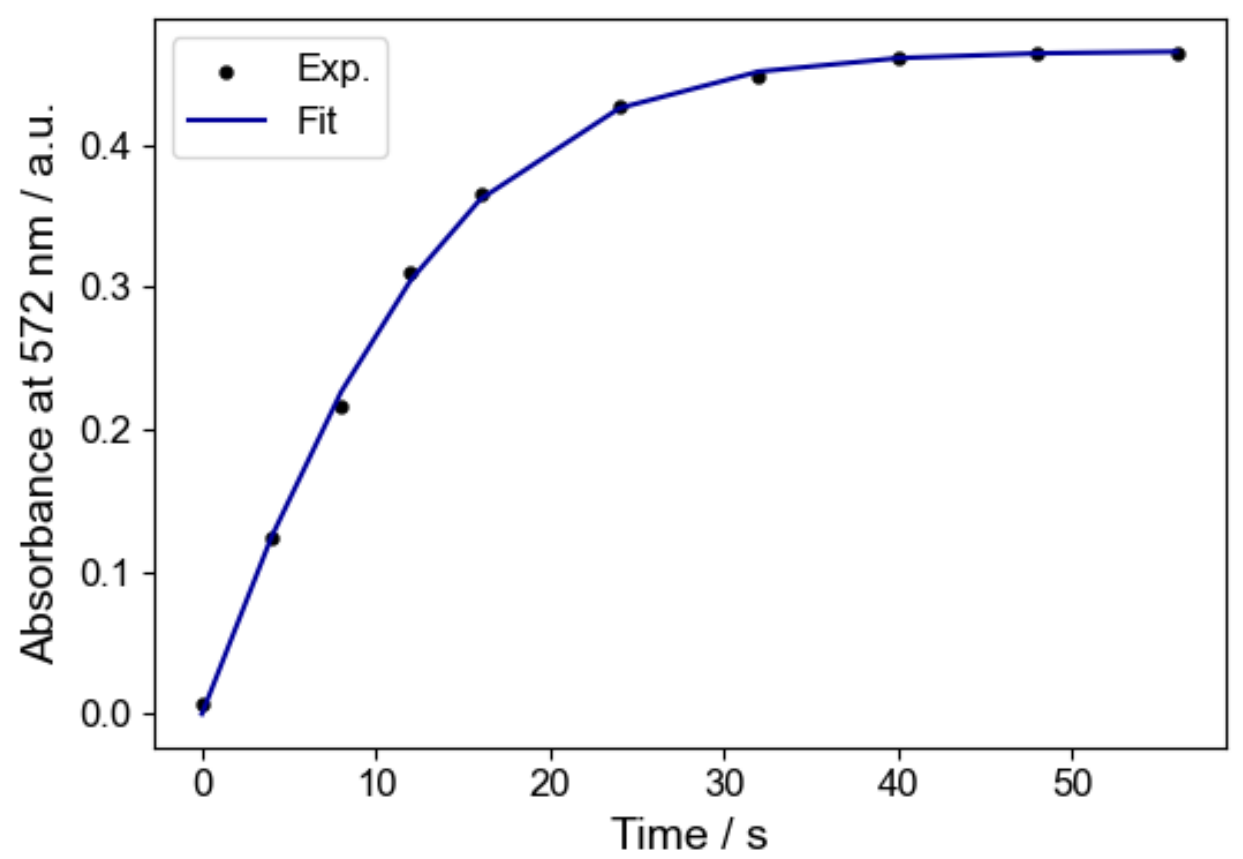

Figure S36. Absorbance of the closed form of **1** (**1-c**) (572 nm) during irradiation with UV light (300 nm, 0–56 s, black dots). Fit by least squares method (blue line).

$c_0 = (2.73 \pm 0.15) \cdot 10^{-5}\ M$
$I_0 = (2.57 \pm 0.06) \cdot 10^{-6}\ E\ L^{-1}\ s^{-1}$
$\varepsilon(open, 300\ \mathrm{nm}) = 54000 \pm 2000\ M^{-1}\ cm^{-1}$
$\varepsilon(closed, 300\ \mathrm{nm}) = 22000 \pm 1000\ M^{-1}\ cm^{-1}$
$\varepsilon(closed, 572\ \mathrm{nm}) = 18000 \pm 1000\ M^{-1}\ cm^{-1}$

$\Phi_{o \to c}(300nm) = \mathbf{0.75 \pm 0.08}$
$\Phi_{c \to o}(300nm) = \mathbf{0.10 \pm 0.11}$

2 (DCM)

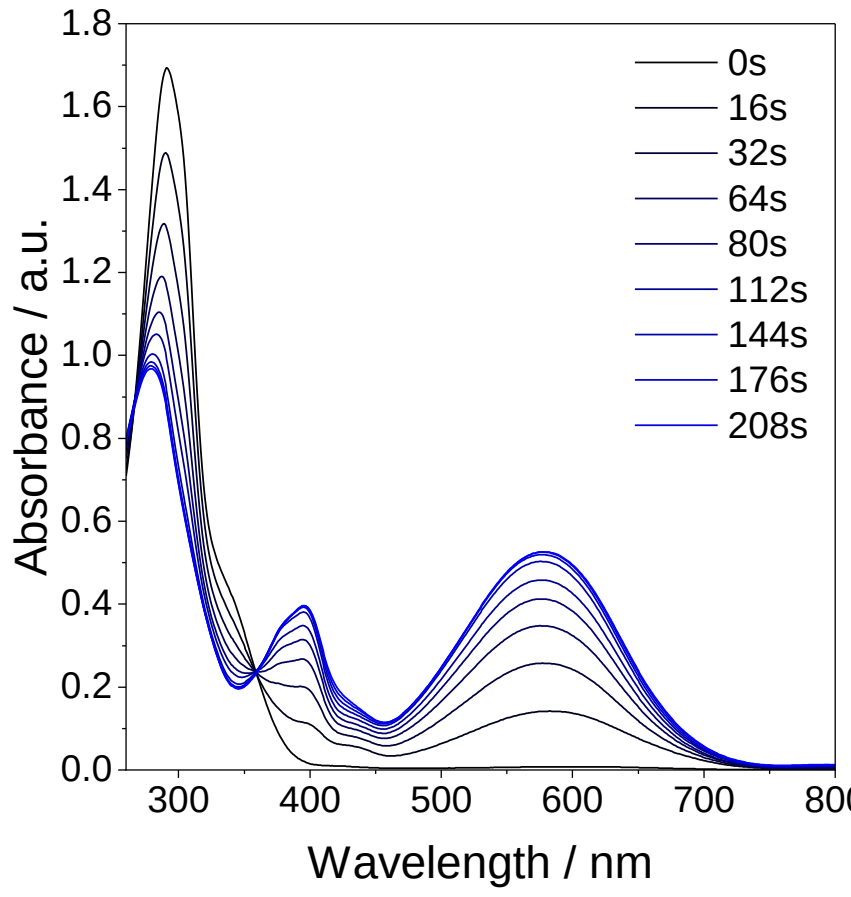

Figure S37. UV-vis spectra of **2** ($3.59 \cdot 10^{-5}\ M$, DCM) during irradiation with UV light (300 nm, 0-208s).

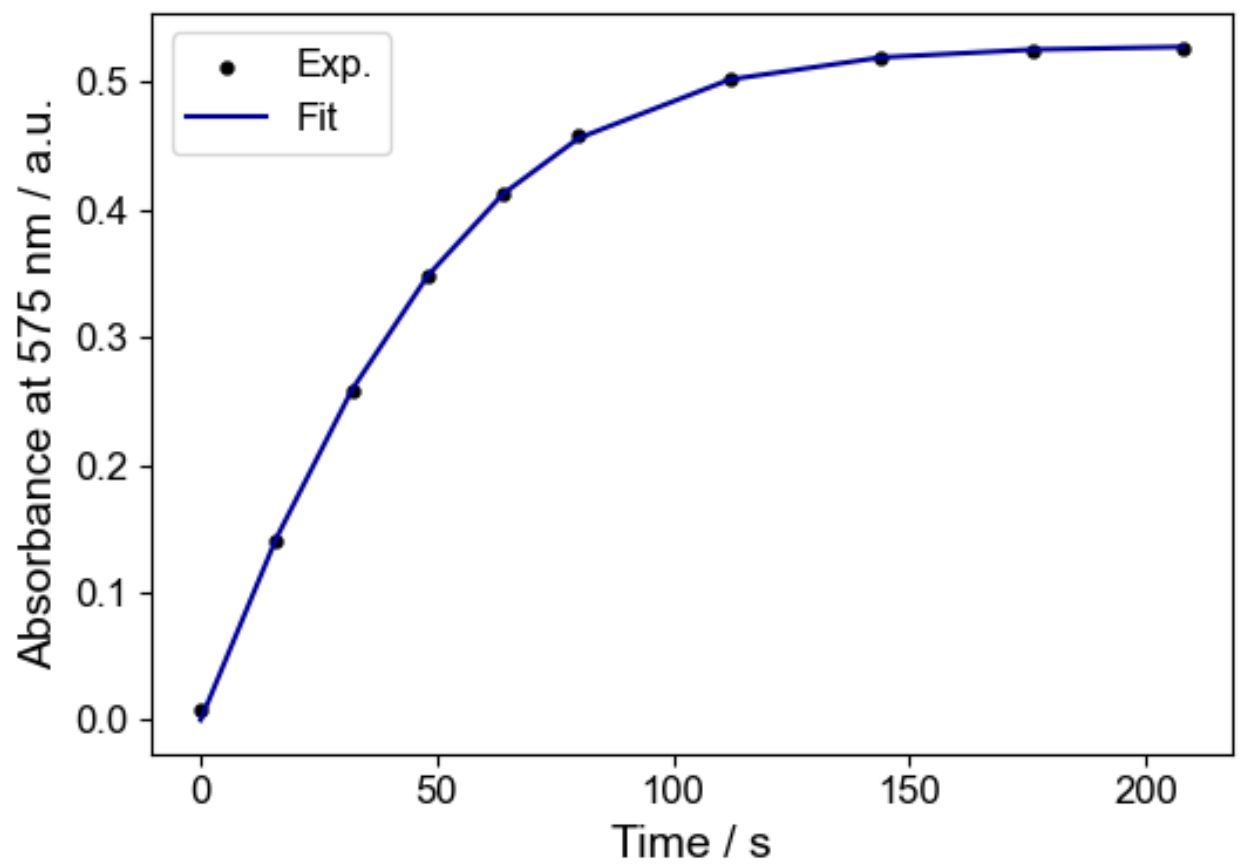

Figure S38. Absorbance of the closed form of **2** (**2-c**) (575 nm) during irradiation with UV light (300 nm, 0–208 s, black dots). Fit by least squares method (blue line).

$c_0 = (3.59 \pm 0.15) \cdot 10^{-5}\ M$
$I_0 = (2.57 \pm 0.06) \cdot 10^{-6}\ E\ L^{-1}\ s^{-1}$
$\varepsilon(open, 300\ \text{nm}) = 44100 \pm 2000\ M^{-1}\ cm^{-1}$
$\varepsilon(closed, 300\ \text{nm}) = 16900 \pm 1000\ M^{-1}\ cm^{-1}$
$\varepsilon(closed, 572\ \text{nm}) = 16700 \pm 1000\ M^{-1}\ cm^{-1}$

$\Phi_{o\rightarrow c}(300nm) = \mathbf{0.23 \pm 0.05}$
$\Phi_{c\rightarrow o}(300nm) = \mathbf{0.08 \pm 0.05}$

2 (Benzene)

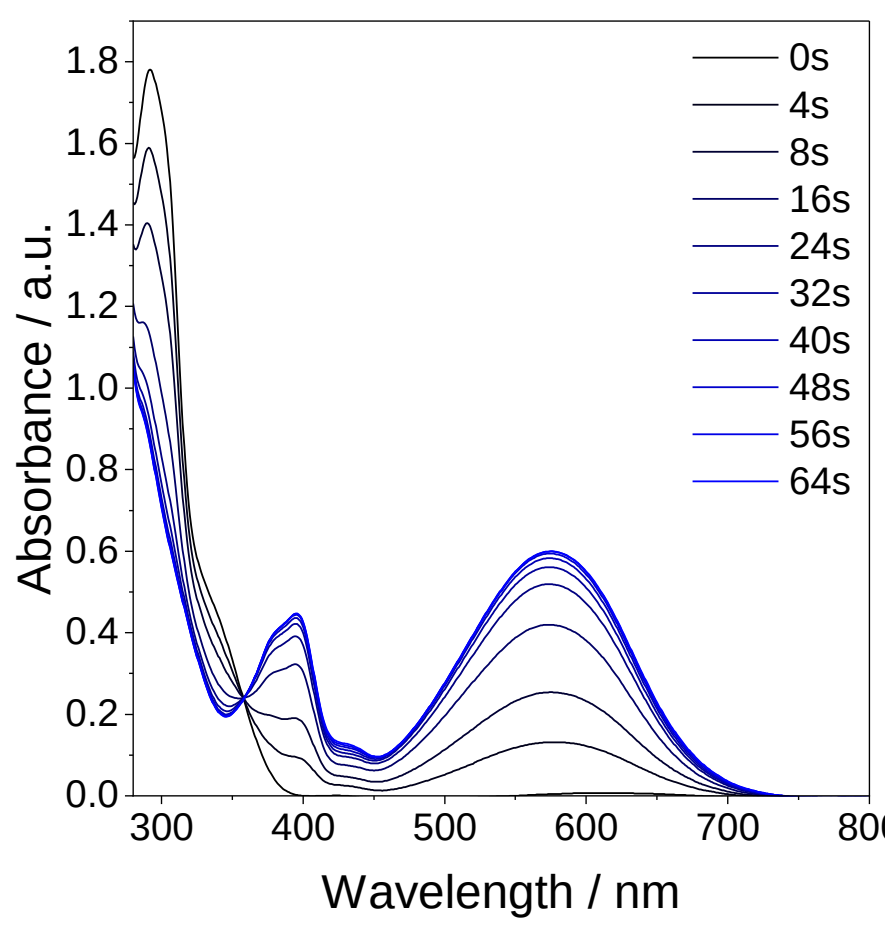

Figure S39. UV-vis spectra of **2** ($3.81 \cdot 10^{-5}\ M$, Benzene) during irradiation with UV light (300 nm, 0-64s).

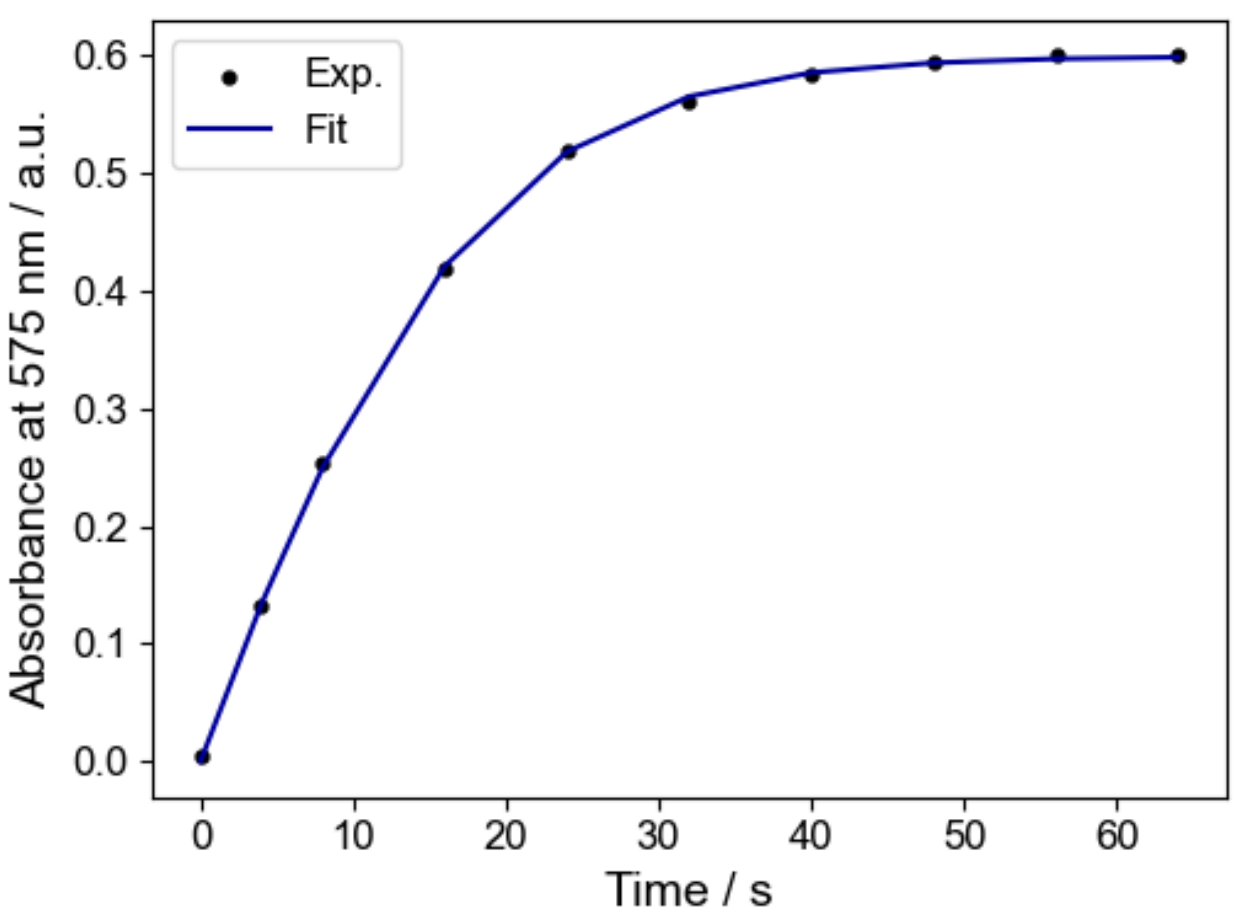

Figure S40. Absorbance of the closed form of **2** (**2-c**) (575 nm) during irradiation with UV light (300 nm, 0–64 s, black dots). Fit by least squares method (blue line).

$c_0 = (3.81 \pm 0.15) \cdot 10^{-5}\ M$
$I_0 = (2.57 \pm 0.06) \cdot 10^{-6}\ E\ L^{-1}\ s^{-1}$
$\varepsilon(open, 300\ \text{nm}) = 44100 \pm 2000\ M^{-1}\ cm^{-1}$
$\varepsilon(closed, 300\ \text{nm}) = 16900 \pm 1000\ M^{-1}\ cm^{-1}$
$\varepsilon(closed, 572\ \text{nm}) = 16700 \pm 1000\ M^{-1}\ cm^{-1}$

$\Phi_{o\rightarrow c}(300nm) = \mathbf{0.84 \pm 0.09}$
$\Phi_{c\rightarrow o}(300nm) = \mathbf{0.14 \pm 0.12}$

2-AuCl (DCM)

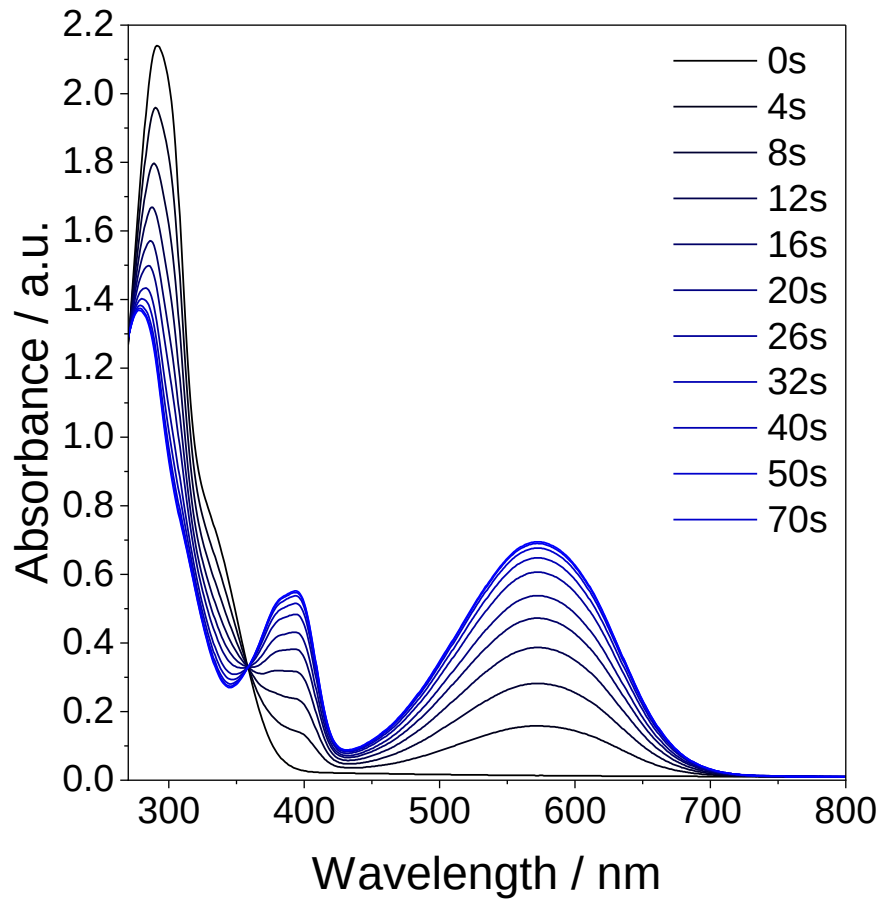

Figure S41. UV-vis spectra of **2-AuCl** ($4.75 \cdot 10^{-5}$ M, DCM) during irradiation with UV light (300 nm, 0-70s).

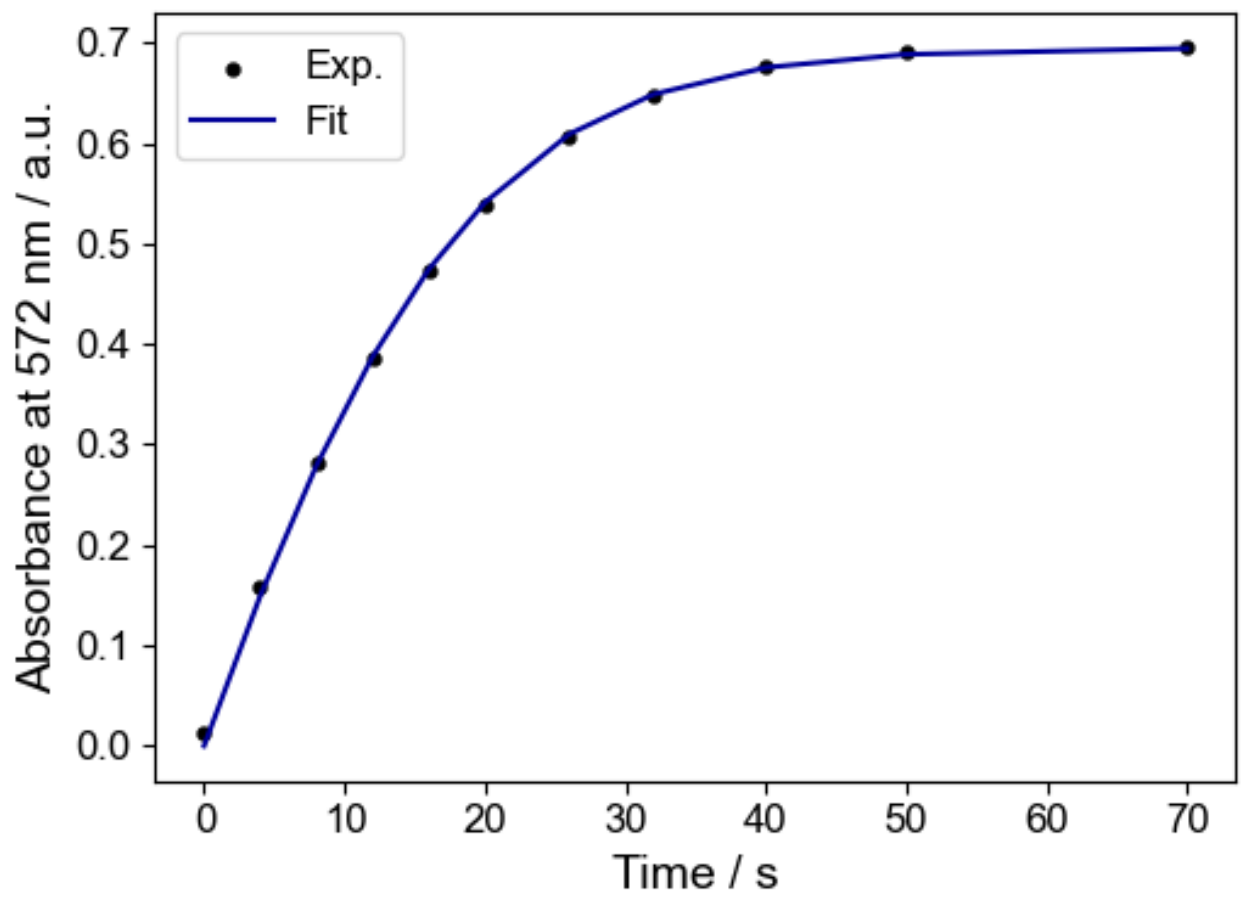

Figure S42. Absorbance of the closed form of **2-AuCl** (**2-c-AuCl**) (572 nm) during irradiation with UV light (300 nm, 0–70 s, black dots). Fit by least squares method (blue line).

$c_0 = (4.75 \pm 0.15) \cdot 10^{-5}\, M$
$I_0 = (2.78 \pm 0.06) \cdot 10^{-6}\, E\, L^{-1}\, s^{-1}$
$\varepsilon(open, 300\text{ nm}) = 42800 \pm 2000\, M^{-1}\, cm^{-1}$
$\varepsilon(closed, 300\text{ nm}) = 18900 \pm 1000\, M^{-1}\, cm^{-1}$
$\varepsilon(closed, 572\text{ nm}) = 15000 \pm 1000\, M^{-1}\, cm^{-1}$

$\Phi_{o\rightarrow c}(300nm) = \mathbf{0.96 \pm 0.09}$
$\Phi_{c\rightarrow o}(300nm) = \mathbf{0.06 \pm 0.10}$

2.3 Fatigue Resistance

Fatigue resistance of **1** and **2** was measured in benzene by repeated irradiation at 300 nm for 60 seconds and broadband visible light for 30 seconds.

1

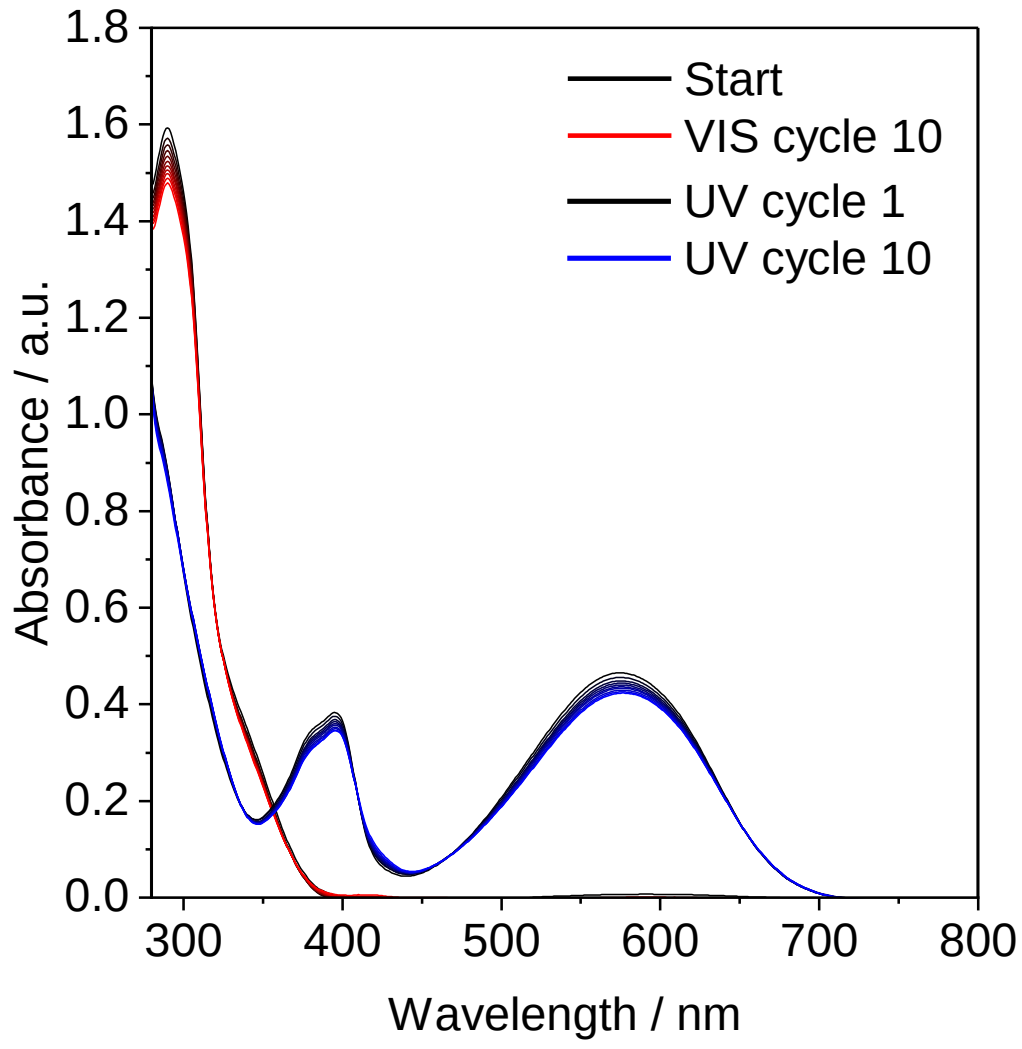

Figure S43. UV-vis spectra of **1** during consecutive switching cycles between open (black→red) and closed (black→blue) form. UV irradiation at 300 nm for 60s, vis irradiation for 30s.

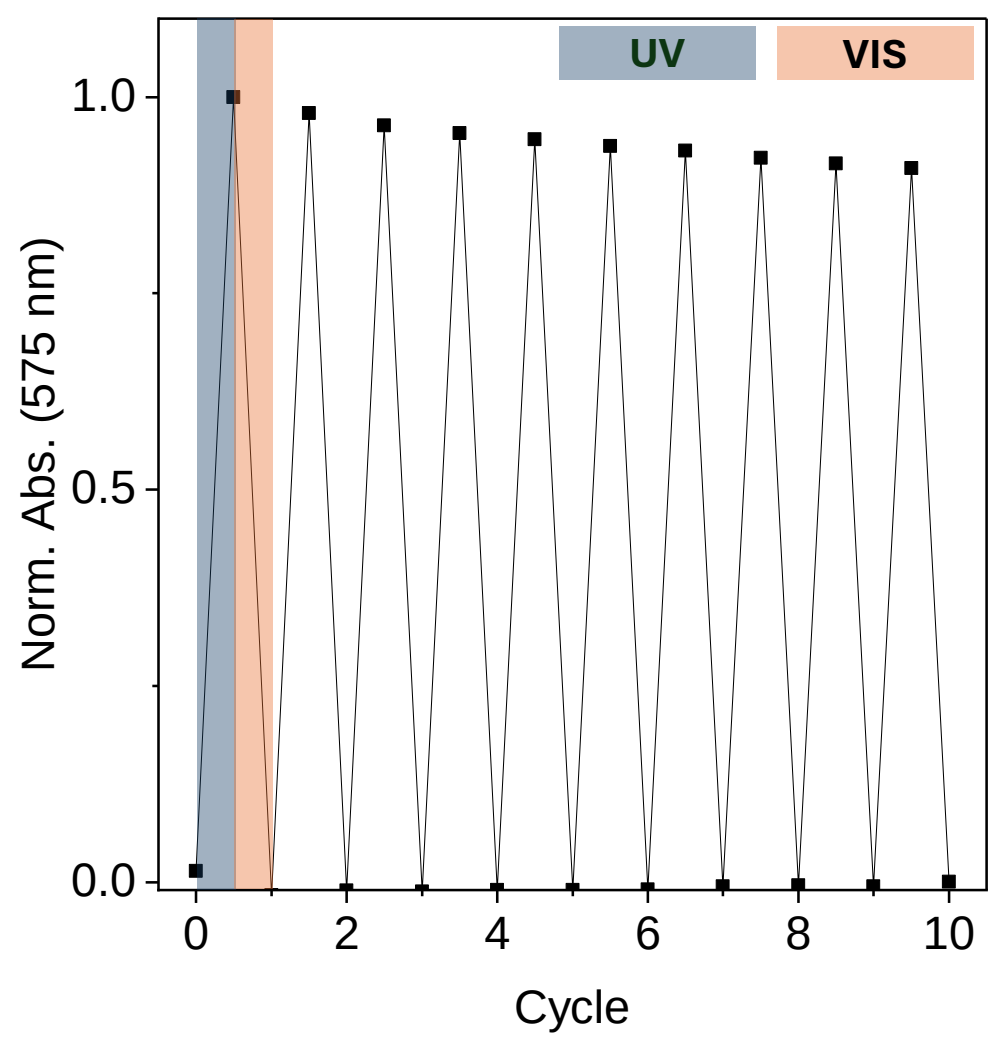

Figure S44. Normalized Absorption at the maximum of the closed form (575 nm) of **1** during 10 switching cycles.

2

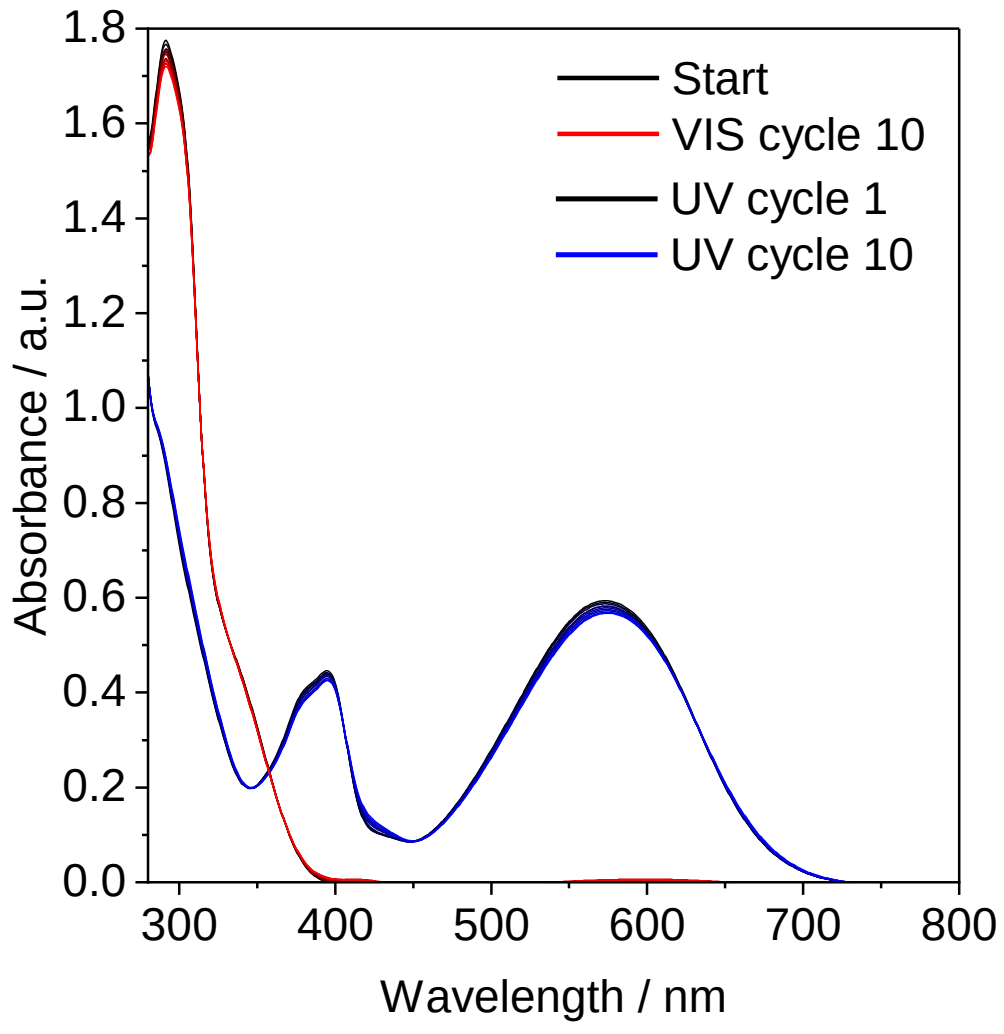

Figure S45. UV-vis spectra of **2** during consecutive switching cycles between open (black→red) and closed (black→blue) form. UV irradiation at 300 nm for 60s, vis irradiation for 30s.

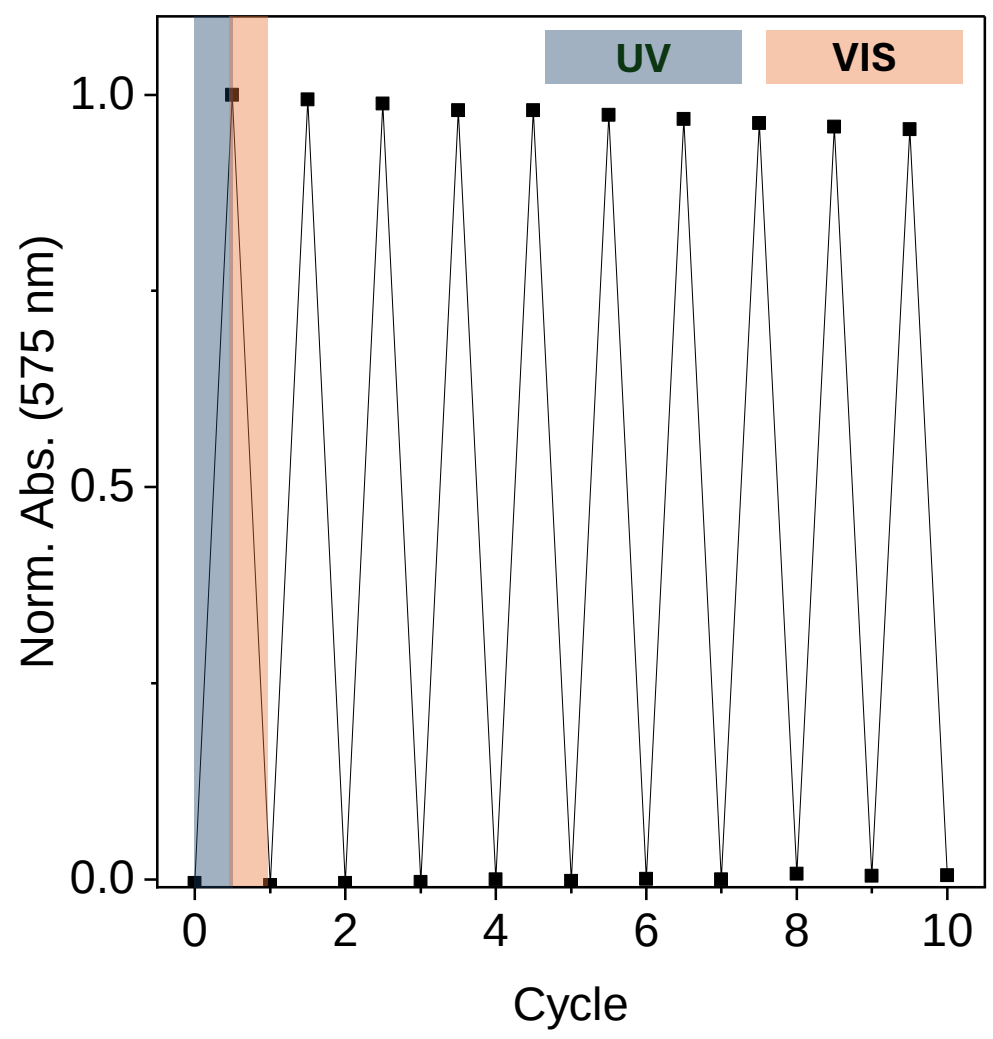

Figure S46. Normalized Absorption at the maximum of the closed form (575 nm) of **2** during 10 switching cycles.

2-AuCl

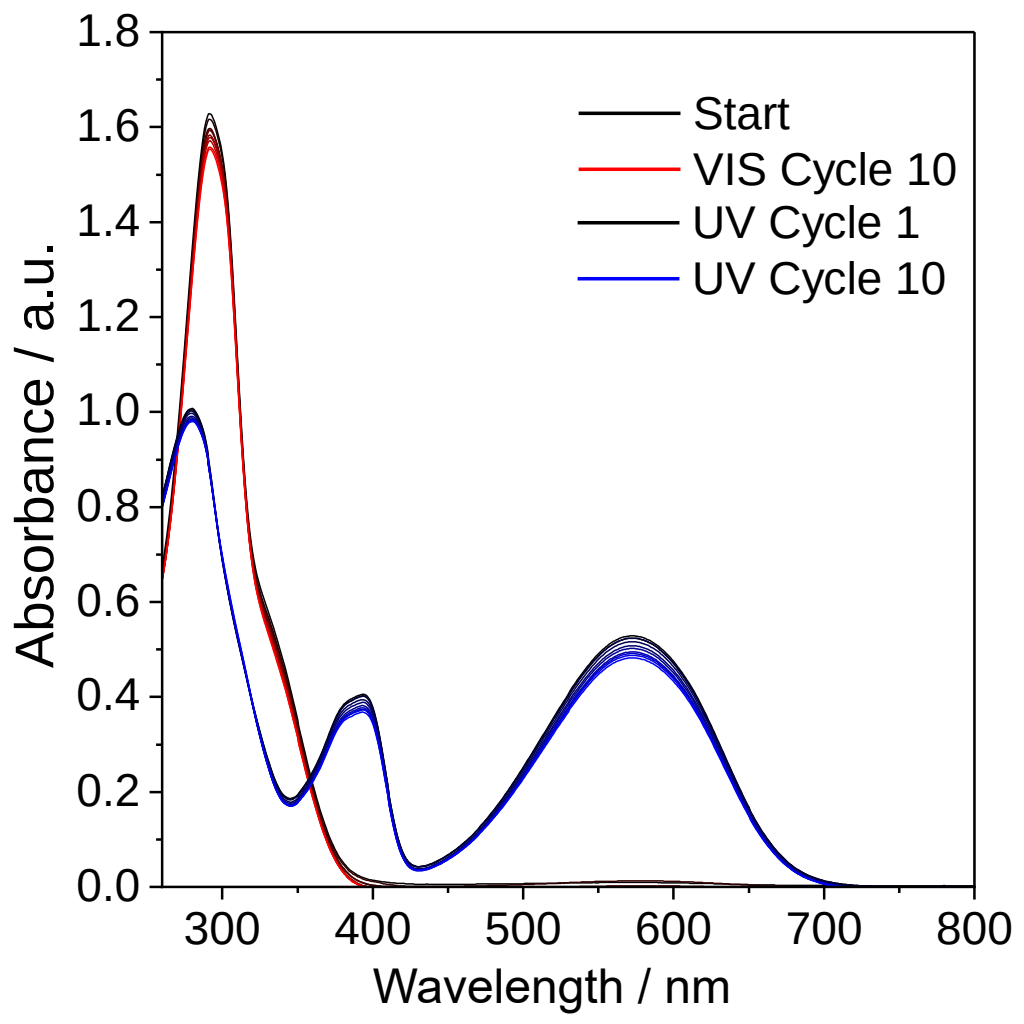

Figure S47. UV-vis spectra of **2** during consecutive switching cycles between open (black→red) and closed (black→blue) form. UV irradiation at 300 nm for 60s, vis irradiation for 30s.

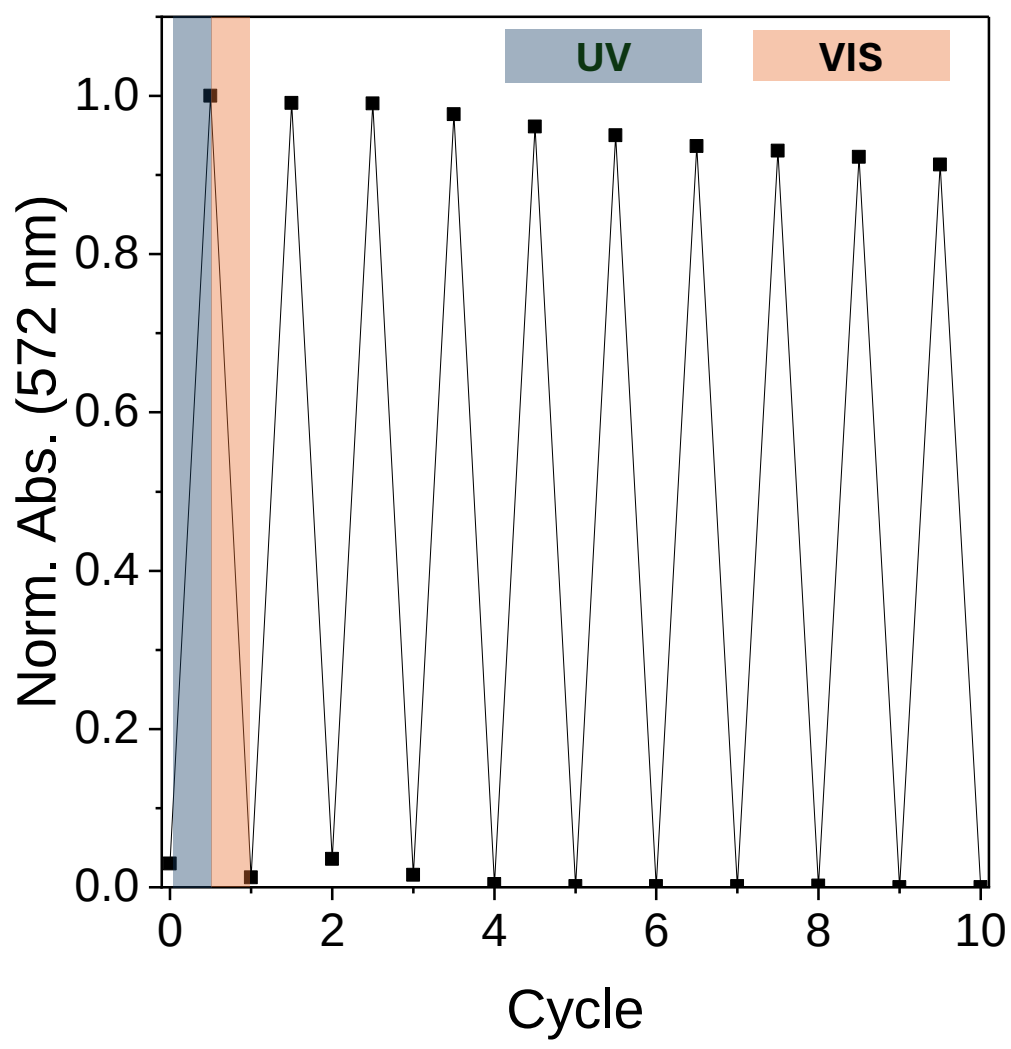

Figure S48. Normalized Absorption at the maximum of the closed form (572 nm) of **2-AuCl** during 10 switching cycles.

2.4 2-Rh(CNCy)₂Cl

For **2-Rh(CNCy)$_2$Cl**, no quantum yield was determined due to partial isomerization during irradiation complicating analysis. Experiments were carried out with the same irradiation setup as for all other compounds. Therefore, the required irradiation time of 300 seconds to reach saturation suggests a lower photocyclization quantum yield compared to the free phosphine **2** or the gold complex **2-AuCl**.

Photocyclization

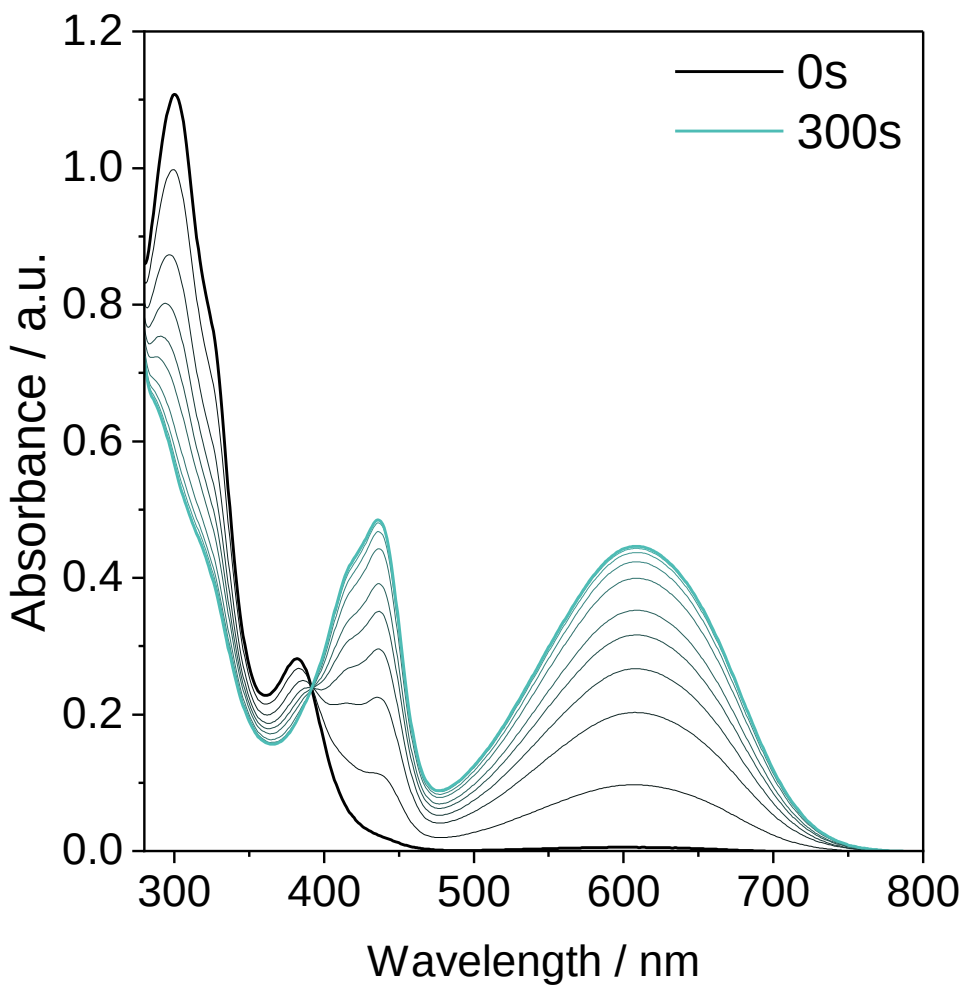

Figure S49. UV-vis spectra of **2-Rh(CNCy)$_2$Cl** ($2.62 \cdot 10^{-5}$ M, DCM) during irradiation with UV light (300 nm, 0-300s).

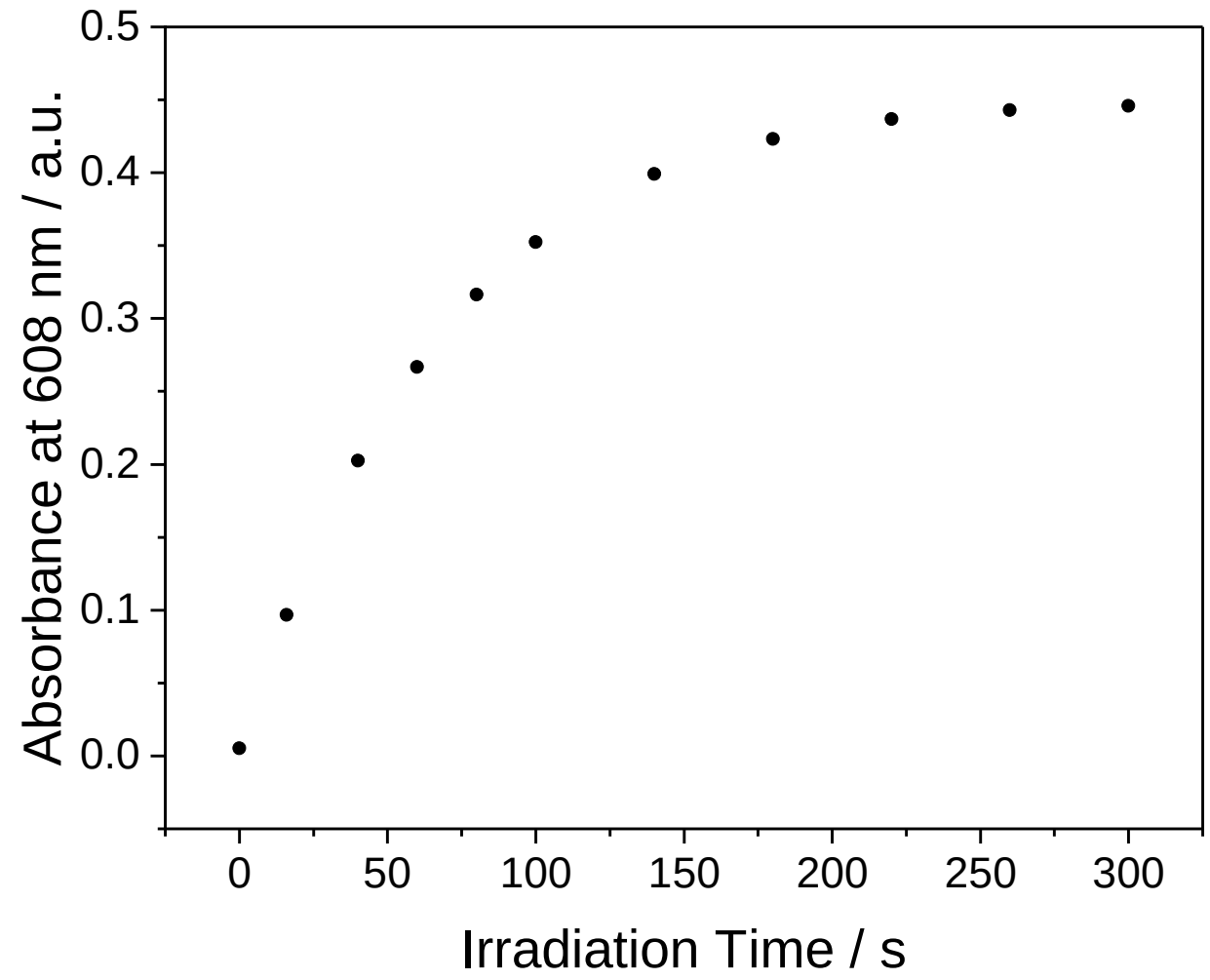

Figure S50. Absorbance of the closed form of **2-Rh(CNCy)$_2$Cl** (**2-c-Rh(CNCy)$_2$Cl**) (608 nm) during irradiation with UV light (300 nm, 0–300 s)

Photocyclization/Cycloreversion Cycles

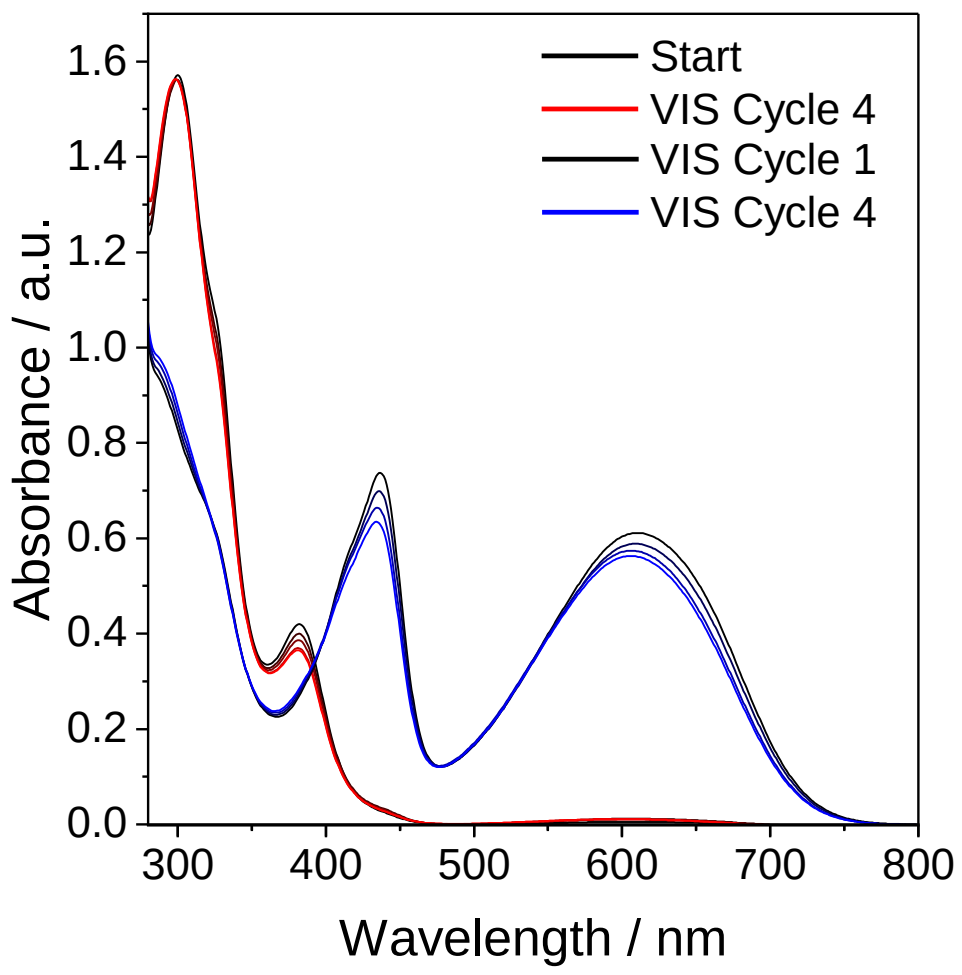

Figure S51. UV-vis spectra of **2-Rh(CNCy)$_2$Cl** during consecutive switching cycles between open (black→red) and closed (black→blue) form. UV irradiation at 300 nm for 300s, vis irradiation for 60s.

3 Computational Section

3.1 General Information

All quantum chemical calculations were carried out with the Orca 6.1.0 program package.[3] High-performance computing resources were provided by JUSTUS2 (Ulm University) through the Baden-Württemberg High Performance Computing (bwHPC) program. For all calculations, the RI approximation for the coulomb integral (RIJCOSX) together with the corresponding auxiliary basis sets were used.[4] SCXRD structures were used as the starting point for geometry optimizations if possible, otherwise conformational search was carried out with the GOAT algorithm with gfn2-xtb as implemented in orca 6.1.0. Geometries were optimized with the PBEh-3c method (or BP86-D4/def2-TZVP for gold complexes), and subsequent vibrational frequency analyses confirmed the structures as minima by the absence of imaginary modes. All single point calculations were carried out at the DSD-BLYP(D3BJ)/def2-QZVPP[5] level of theory. Solvent corrected energies were obtained with the SMD solvent model.[6] Gibbs free energy values were derived from frequency calculations on the PBEh-3c[7] level of theory using the rigid rotor harmonic oscillator (RRHO) approximation.[8] Time-dependant calculations with the Tamm-Dancoff approximation (TDA)[9] were carried out with the long-range corrected hybrid functional ωB97X-D3,[10] the def2-TZVP basis set, and dichloromethane or benzene solvation correction with the SMD solvent model. The first 50 electronic singlet transitions were calculated. Natural transition orbitals were generated for the first 15 excitations. Molecular orbitals and natural transition orbitals were visualized with IboView v20211019-RevA. Natural bond orbitals (NBOs) were visualized with Chemcraft.

3.2 Fluoride Ion Affinities and Proton Affinities

Fluoride ion affinities (**FIA**) were calculated utilizing isodesmic reactions with the TMS/TMSF anchor system as proposed by *Krossing*[11] and *Greb*.[12, 13] The FIA values are given as the negative binding enthalpy (FIA= -ΔH(LA + F → LA-F)).

Reaction 1

LA + TMSF → TMS^{+} + LA-F^{-} ΔH (DSD-BLYP(D3BJ)/def2-QZVPP//PBEh-3c)

Reaction 2

TMSF → TMS^{+} + F^{-} (ΔH = 952.5 kJ/mol[13])

FIA (vacuum) = ΔH(reaction 2) – ΔH(reaction 1)

Proton Affinities (**PA**) are given as the negative enthalpy of protonation at phosphorus (PA= -ΔH(LB + H → LB-H)).

Table S1. Thermodynamic data for the calculation of fluoride ion affinities and proton affinities for **1** and **2**. DSD-BLYP(D3BJ)/def2-QZVPP//PBEh-3c.

	Charge/ Multiplicity	Total Correction [kJ/mol]	SP Energy (vacuum) [kJ/mol]	Enthalpy (vacuum) [kJ/mol]
**1-c**	0/1	1685.65	-11548361.74	-11546673.61
**1-c-F**	-1/1	1690.87	-11810869.72	-11809176.37
**1-c-H**	1/1	1715.58	-11549372.81	-11547654.76
**1-o**	0/1	1684.98	-11548443.74	-11546756.28
**1-o-F**	-1/1	1689.11	-11810874.23	-11809182.64
**1-o-H**	1/1	1715.21	-11549464.99	-11547747.30
**2-c**	0/1	1700.36	-10954820.68	-10953117.84
**2-c-F**	-1/1	1706.16	-11217327.37	-11215618.73
**2-c-H**	1/1	1731.63	-10955852.68	-10954118.57
**2-o**	0/1	1700.74	-10954902.42	-10953199.21
**2-o-F**	-1/1	1704.36	-11217317.05	-11215610.21
**2-o-H**	1/1	1731.41	-10955947.01	-10954213.12
**TMS**	1/1	315.05	-1073010.79	-1072693.26
**TMSF**	0/1	328.84	-1336052.07	-1335720.75

Table S2. Fluoride ion affinities (FIA) and proton affinities (PA) of **1** and **2**.

Compound	FIA (vacuum) [kJ/mol]	PA (vacuum) [kJ/mol]
**1-o**	351	991
**1-c**	428	981
**2-o**	336	1014
**2-c**	428	1001

3.3 Substrate Binding Thermodynamics

Table S3. Thermodynamic data for the calculation of substrate binding thermodynamics for **1** and **2**. DSD-BLYP(D3BJ)/def2-QZVPP//PBEh-3c. Solvent correction with SMD(benzene) and additionally with SMD(DCM) for CO_2. Charge (0) and multiplicity (1) identical for all species.

Compound	Total Correction [kJ/mol]	Entropy Term [kJ/mol]	Single Point Energy [kJ/mol]	Final Enthalpy [kJ/mol]	Final Gibbs Free Energy [kJ/mol]
**Benzaldehyde (PhCHO)**	314.19	98.38	-906682.48	-906365.81	-906464.20
**Benzophenone (BP)**	546.58	124.98	-1512863.81	-1512314.75	-1512439.73
**CO_2**	39.08	65.27	-494927.83	-494886.27	-494951.55
**PhNCS**	294.62	107.86	-1896310.08	-1896012.98	-1896120.84
**Propylene Oxide (PO)**	243.07	82.78	-506675.96	-506430.40	-506513.19
**tBuNC**	370.38	100.21	-657628.57	-657255.71	-657355.92
**2-c**	1700.36	351.19	-10954897.36	-10953194.52	-10953545.71
**2-c-PhCHO**	2025.55	377.73	-11861725.94	-11859697.91	-11860075.64
**2-c-BP**	2256.70	395.93	-12467864.82	-12465605.64	-12466001.57
**2-c-CO_2**	1747.17	356.74	-11449879.99	-11448130.34	-11448487.08
**2-c-PhNCS**	2006.13	382.65	-12851349.31	-12849340.70	-12849723.35
**2-c-tBuNC_E**	2080.28	381.94	-11612618.44	-11610535.68	-11610917.62
**2-c-tBuNC_Z**	2080.44	379.87	-11612587.31	-11610504.39	-11610884.25
**2-o**	1700.74	358.58	-10954975.84	-10953272.63	-10953631.21
**2-o-PhCHO**	2023.95	388.44	-11861759.15	-11859732.72	-11860121.16
**2-o-BP**	2256.29	403.21	-12467882.83	-12465624.06	-12466027.27
**2-o-CO_2**	1746.12	380.97	-11449929.61	-11448181.01	-11448561.98
**2-o-PhNCS**	2004.61	392.45	-12851397.80	-12849390.71	-12849783.16
**2-o-tBuNC**	2078.56	391.94	-11612653.18	-11610572.14	-11610964.08
**1-c**	1685.65	352.23	-11548452.08	-11546763.95	-11547116.18
**1-c-PhCHO**	2008.53	382.12	-12455249.24	-12453238.23	-12453620.35
**1-c-PhNCS**	1988.83	389.70	-12043407.31	-12041674.52	-12042038.04
**1-c-PO**	1937.61	372.14	-13444886.17	-13442894.87	-13443284.57
**1-o**	1684.98	362.82	-12055363.85	-12053423.76	-12053795.90
**1-o-BenzAld**	2006.96	392.85	-11548534.65	-11546847.19	-11547210.01
**1-o-PhNCS**	1987.39	400.84	-12597410.31	-12595414.75	-12595814.43
**1-o-PO**	1936.24	383.18	-12043477.11	-12041744.49	-12042131.55
**2-o (DCM)**	1700.74	358.58	-10954990.67	-10953287.46	-10953646.04
**2-o-CO_2 (DCM)**	1746.12	380.97	-11449942.10	-11448193.50	-11448574.47
**2-c (DCM)**	1700.36	351.19	-10954913.27	-10953210.43	-10953561.62
**2-c-CO_2 (DCM)**	1747.17	356.74	-11449908.44	-11448158.79	-11448515.53
**CO_2 (DCM)**	39.08	65.27	-494924.80	-494883.24	-494948.52

Table S4. Substrate binding thermodynamics for **1-o**,**1-c**, **2-o**, and **2-c**. All values given in kJ/mol. All computations with SMD(benzene) unless otherwise noted.

Substrate	ΔH (1-o)	ΔG (1-o)	ΔH (1-c)	ΔG (1-c)	ΔH (2-o)	ΔG (2-o)	ΔH (2-c)	ΔG (2-c)
**PhCHO**	-57.76	10.60	-108.47	-39.97	-94.28	-25.75	-137.58	-65.73
**BP**	--	--	--	--	-36.68	43.67	-96.37	-16.13
**CO_2**	--	--	--	--	-22.11	20.78	-49.55	10.18
**CO_2 (DCM)**	--	--	--	--	-22.80	20.09	-65.12	-5.39
**PhNCS**	-77.56	-7.72	-117.94	-47.55	-105.10	-31.11	-133.20	-56.80
**PO**	-177.83	-115.39	-229.41	-166.53	--	--	--	--
**tBuNC E**	--	--	--	--	-43.80	23.05	-85.45	-15.99
**tBuNC Z**	--	--	--	--	--	--	-54.16	17.38

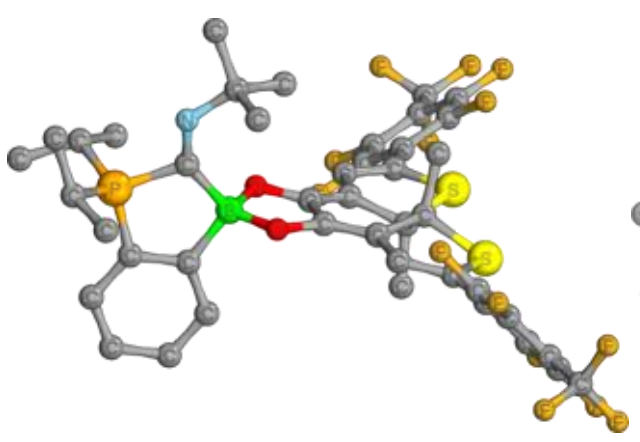

2-c-tBuNC_E

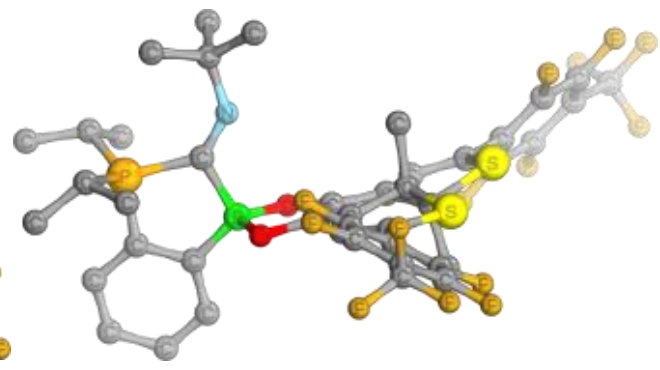

2-c-tBuNC_Z

3.4 Computed Structures and NBO analysis of 2-AuCl and 2-Rh(CNCy)$_2$Cl

3.4.1 2-AuCl

For the geometry optimization of **2-o-AuCl**, multiple functionals were tested to reproduce the Au-B distance observed by scXRD (3.100 Å). The BP86 functional best reproduced the distance (3.19 Å), while functionals with HF exchange tended to overestimate the distance (B3LYP-D4: 3.31 Å, PBEh-3c: 3.26, wB97X-D4: 3.36). Effective core potential (def2-ECP) was employed for relativistic treatment.

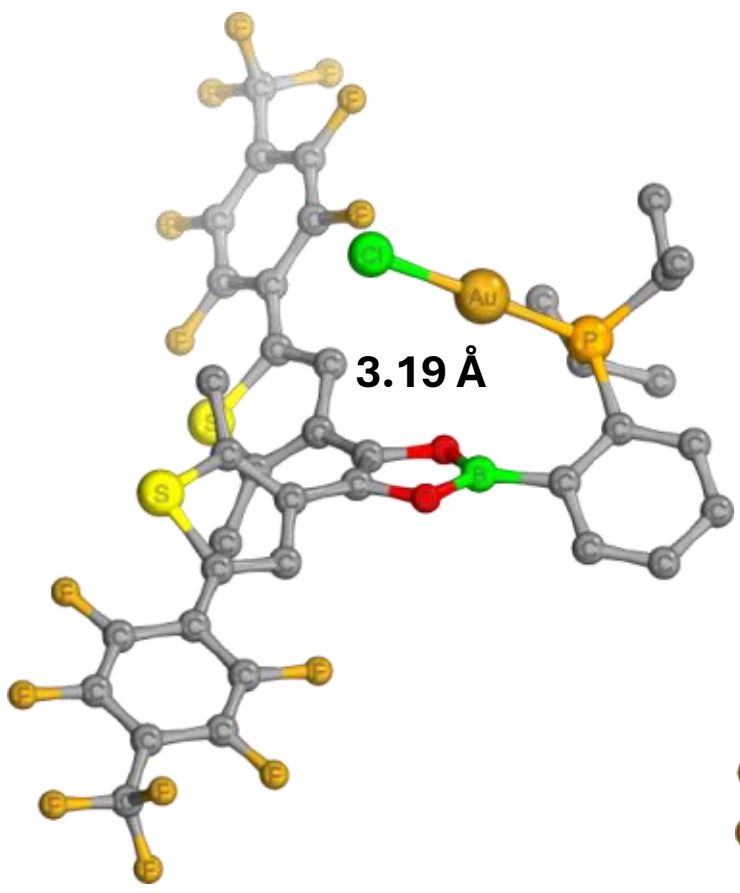

2-o-AuCl (BP86-D4/def2-TZVP)

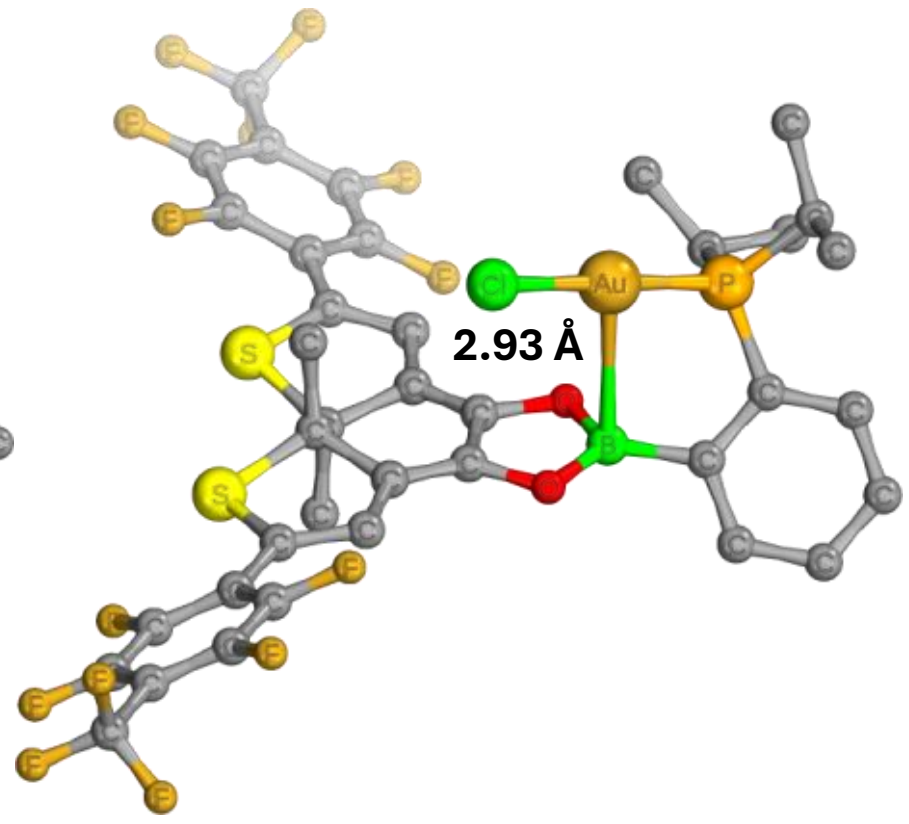

2-c-AuCl (BP86-D4/def2-TZVP)

<u>**NBO Analysis**</u>

Open

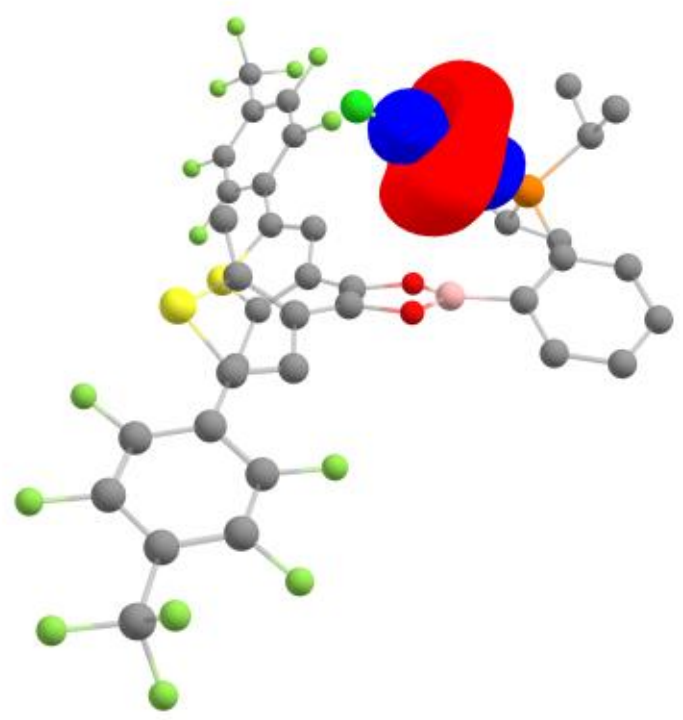

LP(5)Au, Occ. 1.98

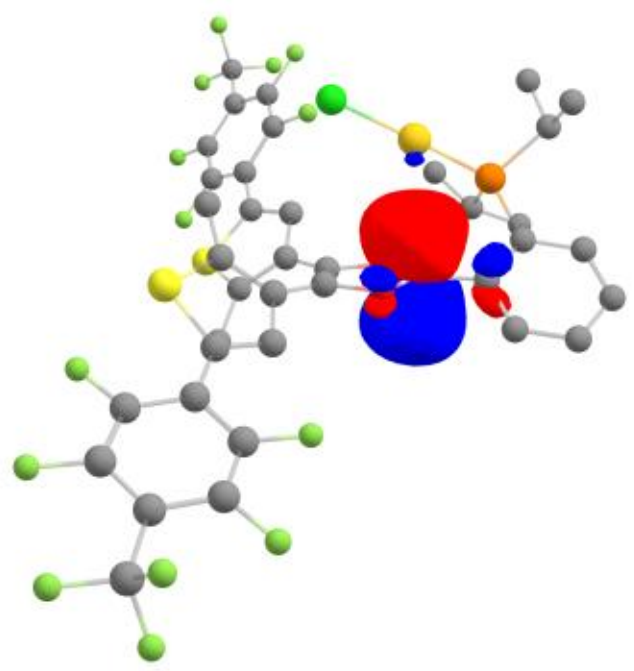

LV(1)B, Occ. 0.41

Au→B second order perturbation theory stabilization energy

LP(5)Au → LV(1)B 2.1 kcal/mol

Wiberg Bond Indices

Au-B: 0.02

Au-P: 0.62

Natural Charge

Au: 0.34

B: 1.07

Closed

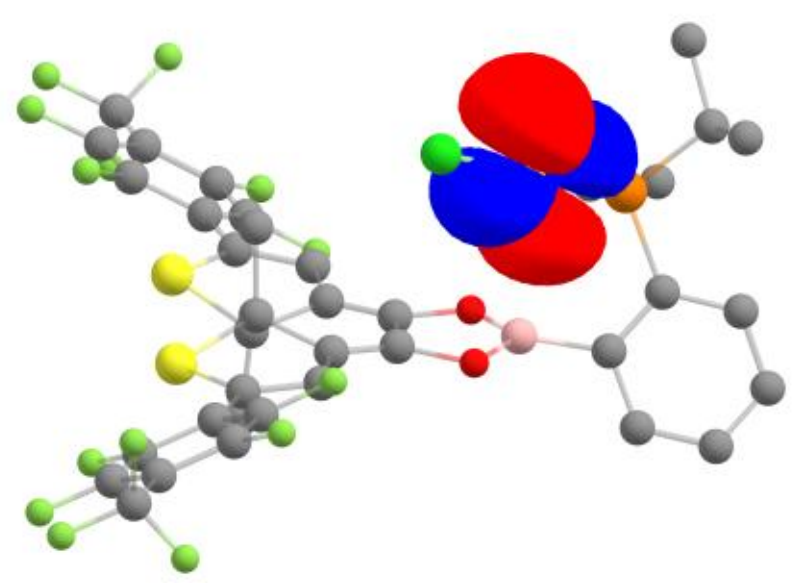

LP(5)Au, Occ. 1.94

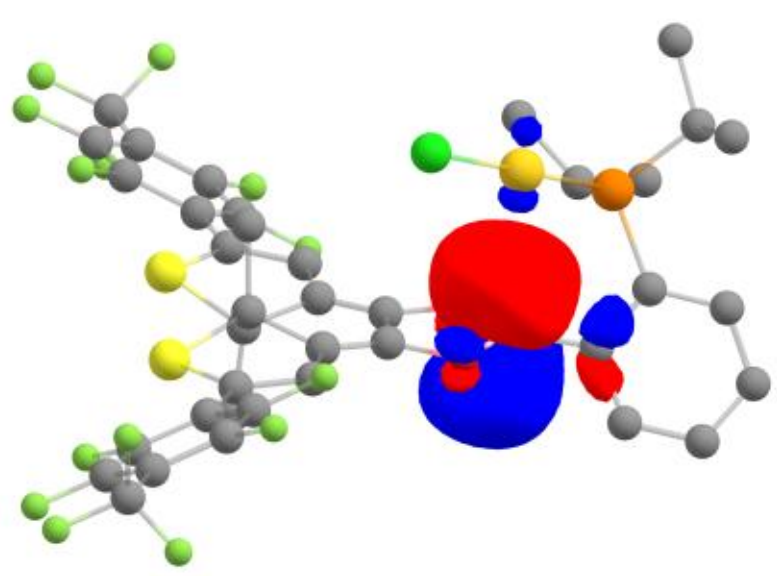

LV(1)B, Occ. 0.39

Au→B second order perturbation theory stabilization energies

LP(5)Au → LV(1)B 7.1 kcal/mol

Wiberg Bond Indices

Au-B: 0.07

Au-P: 0.61

Natural Charge

Au: 0.36

B: 1.09

3.4.2 2-Rh(CNCy)$_2$Cl

For cyclohexyl and thiophene residue conformations, conformer search with the GOAT algorithm (gfn2-xtb) as implemented in orca 6 was carried out (unless scXRD data was available as the starting point for geometry optimization). For the cis conformation in the closed form, a Cl→B coordination mode was identified as most stable.

Table S5. Thermodynamic data of the structural isomers of **2-Rh(CNCy)$_2$Cl**. DSD-BLYP(D3BJ)/def2-QZVPP/SMD(C_6H_6)//PBEh-3c.

Compound	Total Correction [kJ/mol]	Entropy Term [kJ/mol]	Single Point Energy [kJ/mol]	Final Enthalpy [kJ/mol]	Final Gibbs Free Energy [kJ/mol]
Closed_cis	2687.57	446.46	-14174842.02	-14172151.98	-14172598.44
Closed_trans	2687.96	447.41	-14174869.84	-14172179.39	-14172626.80
Open_cis	2688.84	457.26	-14174934.73	-14172243.41	-14172700.67
Open_trans	2687.97	454.97	-14174939.79	-14172249.33	-14172704.30

Open

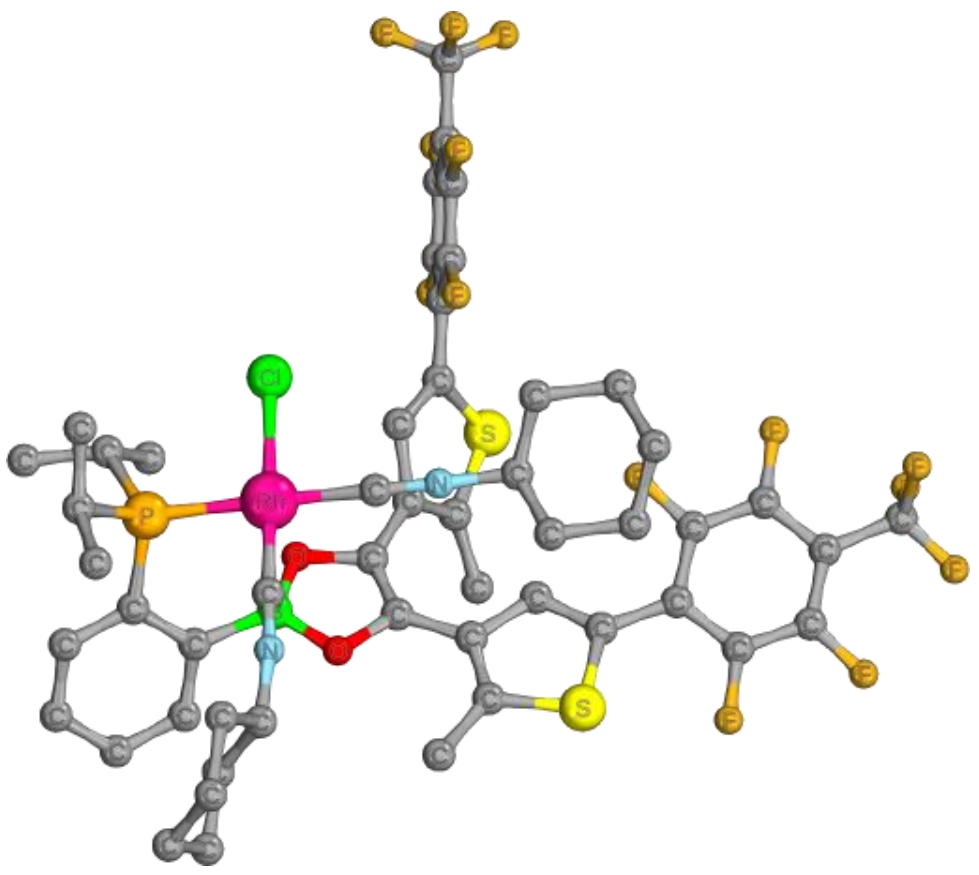

cis, *E* = +3.6 kJ mol^{-1}

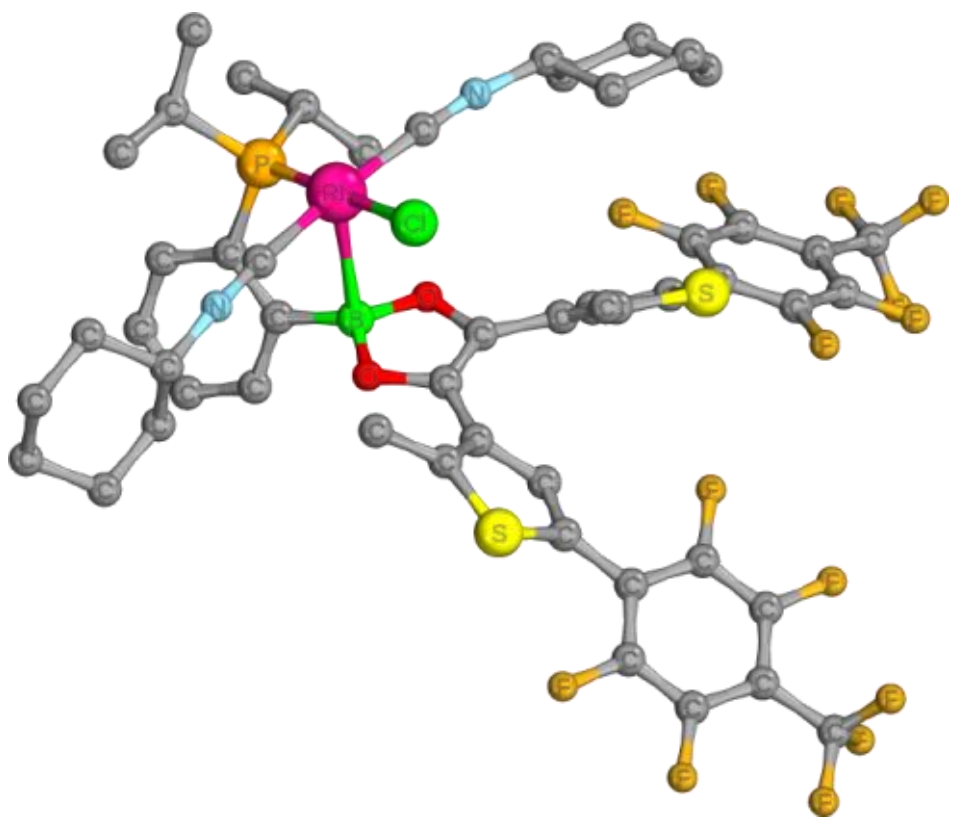

trans, *E* = 0.0 kJ mol^{-1}

Closed

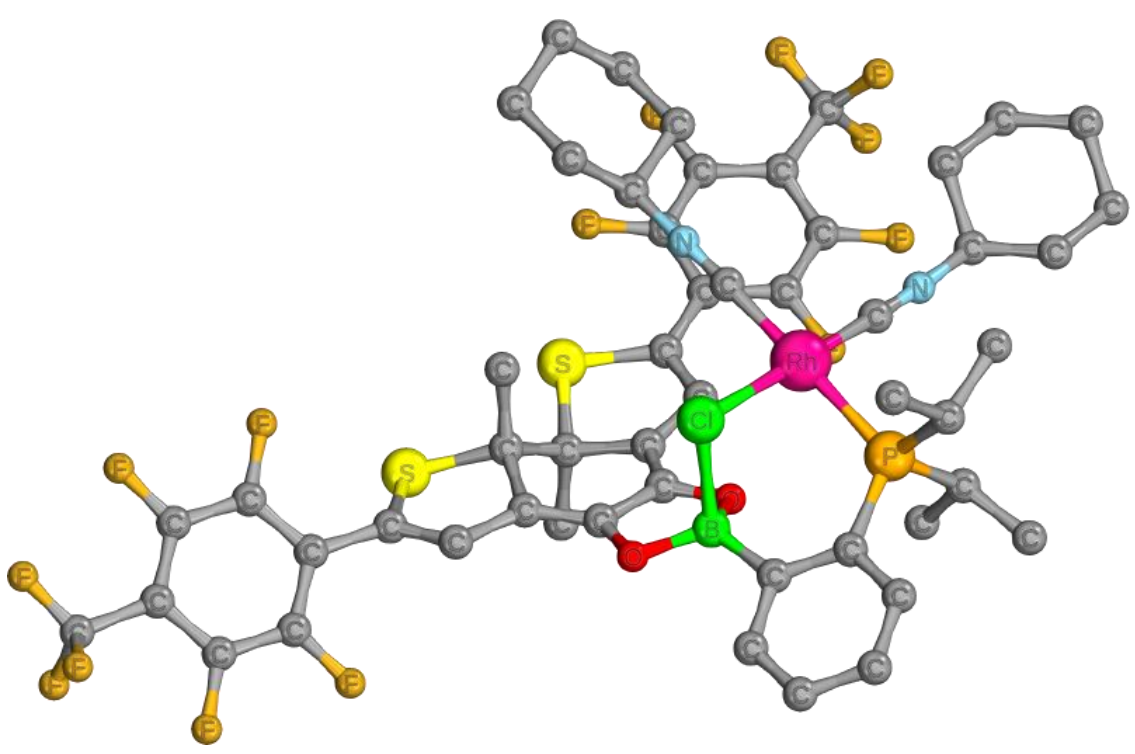

cis, *E* = +28.4 kJ mol^{-1}

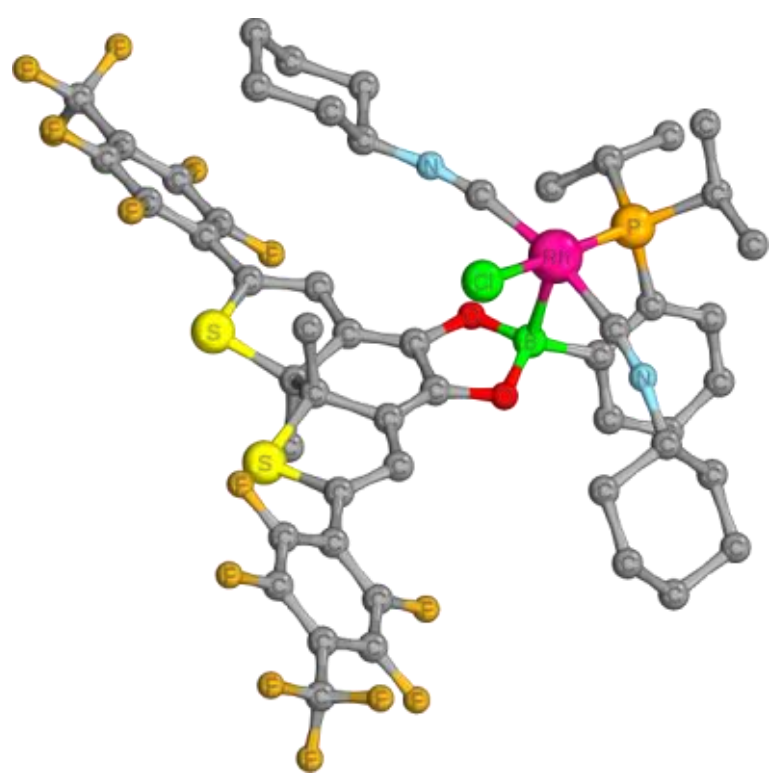

trans, *E* = 0.0 kJ mol^{-1}

NBO Analysis

Open

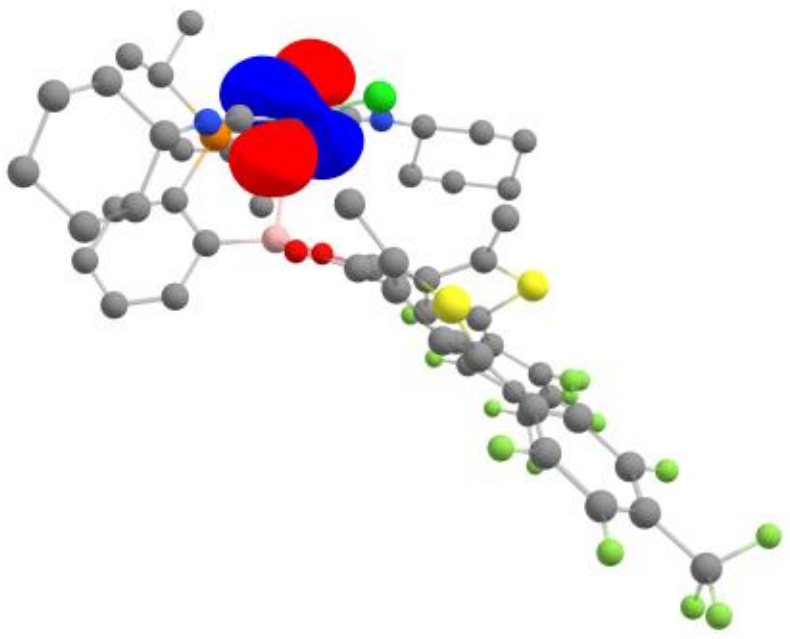

LP(1)Rh, Occ. 1.95

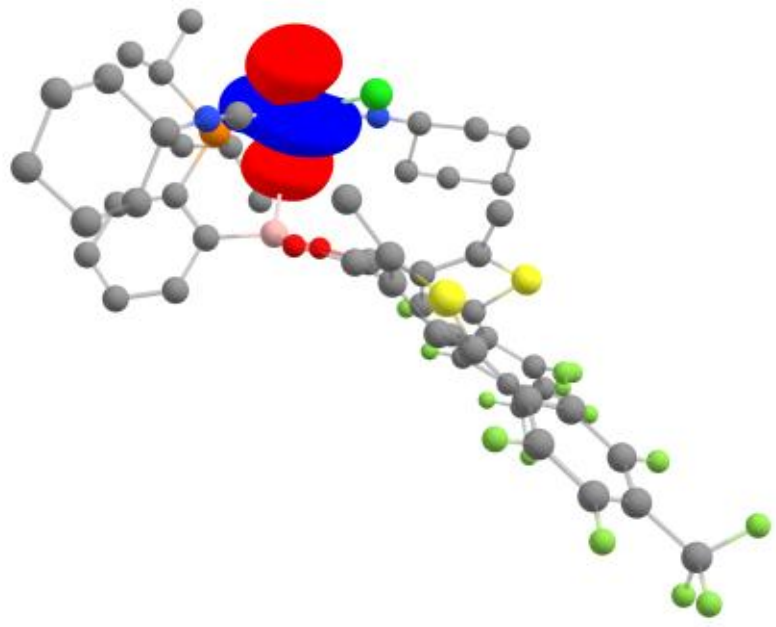

LP(2)Rh, Occ. 1.88

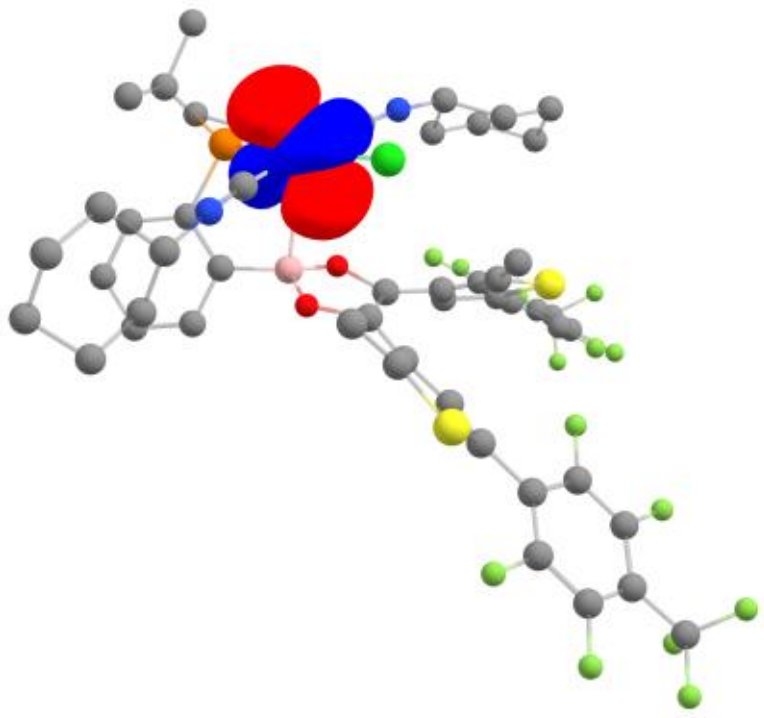

LP(4)Rh, Occ. 1.83

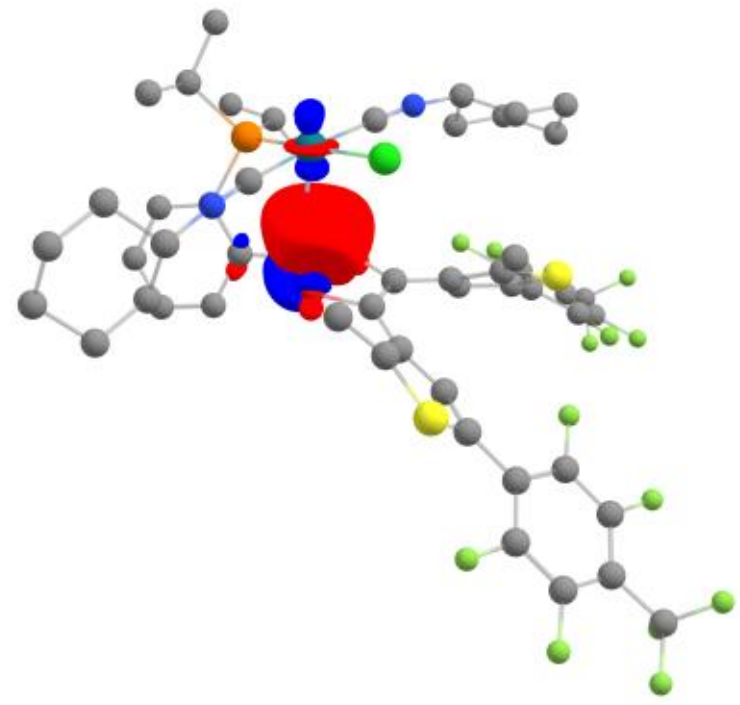

LV(1)B, Occ. 0.46

Rh→B second order perturbation theory stabilization energies

LP(1)Rh → LV(1)B 6.3 kcal/mol

LP(2)Rh → LV(1)B 16.2 kcal/mol

LP(4)Rh → LV(1)B 6.5 kcal/mol

Wiberg Bond Indices

Rh-B: 0.15

Rh-P: 0.59

Rh-C (CNCy): 0.61, 0.61

Natural Charges

Rh: -0.18

B: 1.08

Closed

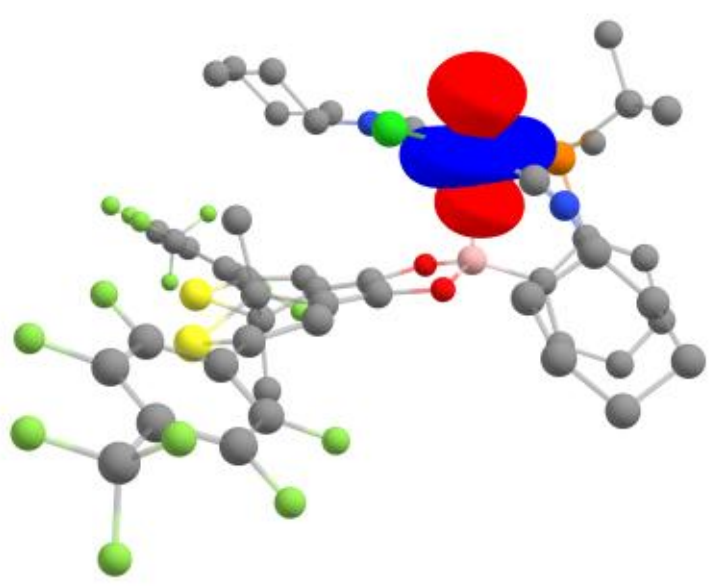

LP(4)Rh, Occ. 1.72

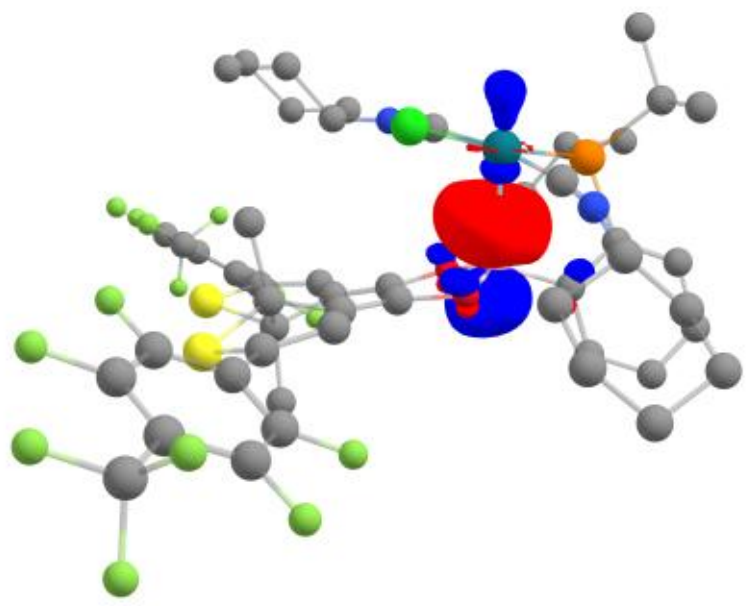

LV(1)B, Occ. 0.52

Rh→B second order perturbation theory stabilization energies

LP(4)Rh → LV(1)B 80.2 kcal/mol

Wiberg Bond Indices

Rh-B: 0.39

Rh-P: 0.55

Rh-C (CNCy): 0.57, 0.57

Natural Charges

Rh: -0.10

B: 1.0

3.5 TD-DFT

1

Table S6. Low energy transitions of the open and closed isomer of **1**. Method: ωB97X-D3/def2-TZVPP/SMD(DCM).

		E / eV	*f*	Orbital Contributions
**open**	S1	4.535	0.818	HOMO → LUMO 48%, HOMO-2 → LUMO 16 %,<br>HOMO →LUMO+1 8%. HOMO-2 → LUMO+1 6%
**closed**	S1	2.585	0.574	HOMO → LUMO 91%
	S2	3.849	0.566	HOMO-2 → LUMO 46%, HOMO → LUMO+1 37%

1-o

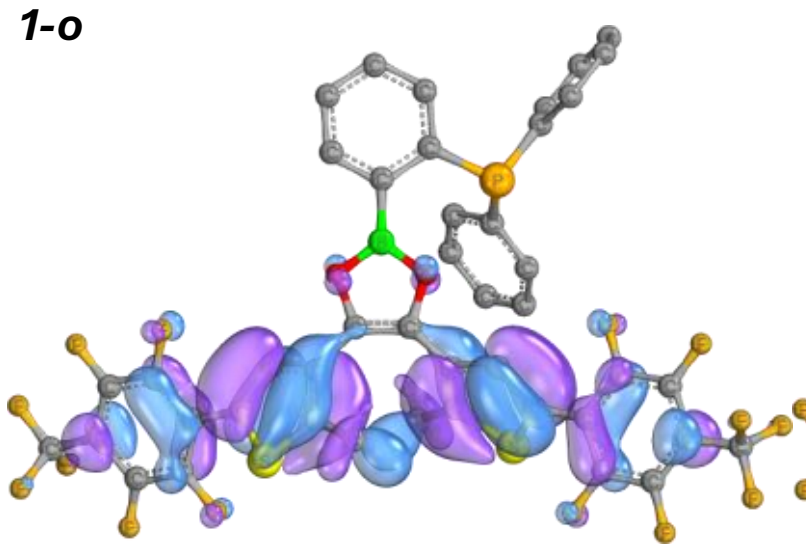

HOMO-2, E = –8.59 eV

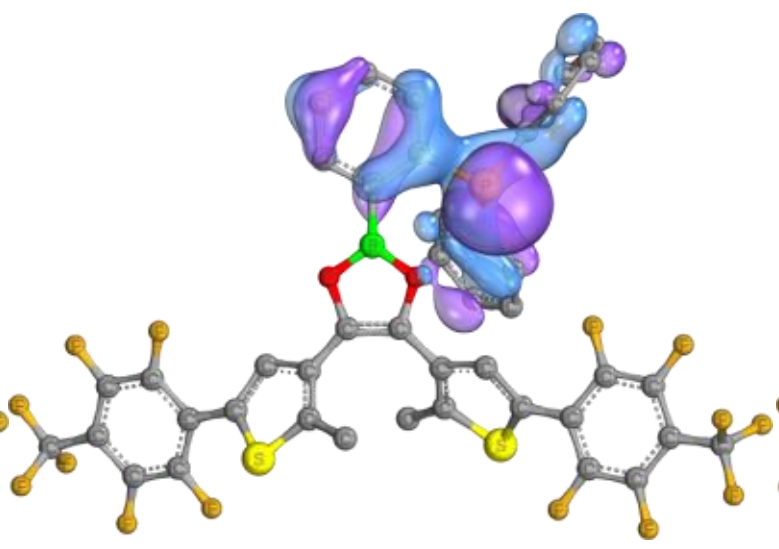

HOMO-1, E = –8.24 eV

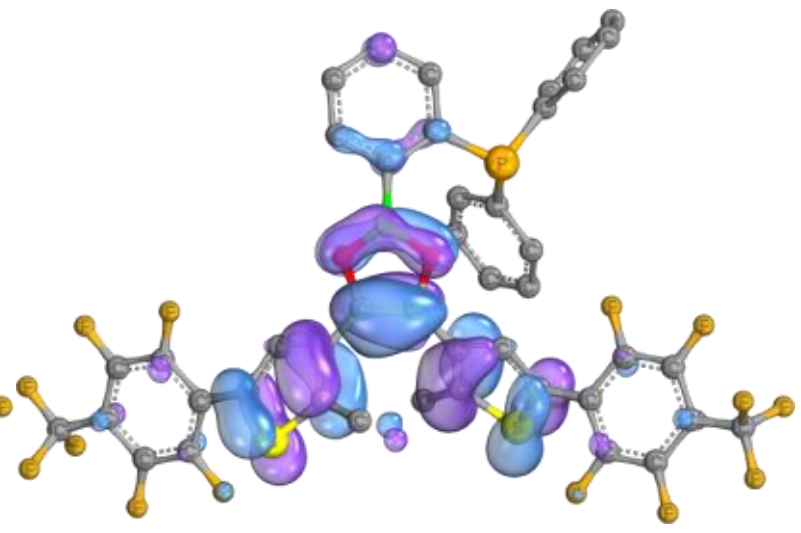

HOMO, E = –7.79 eV

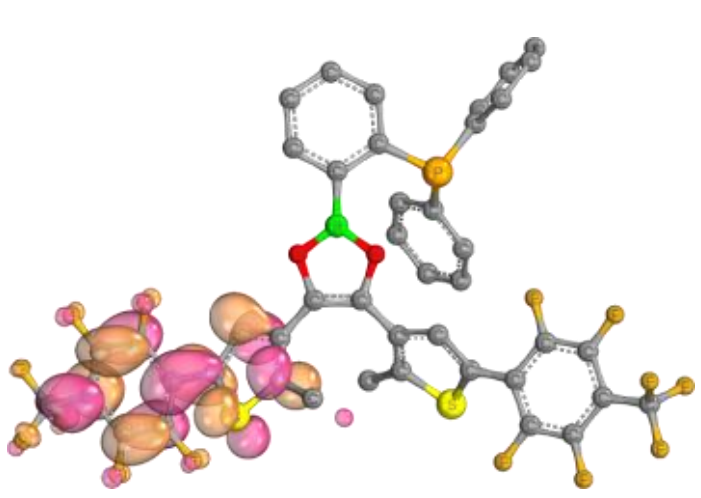

LUMO, E = 0.02 eV

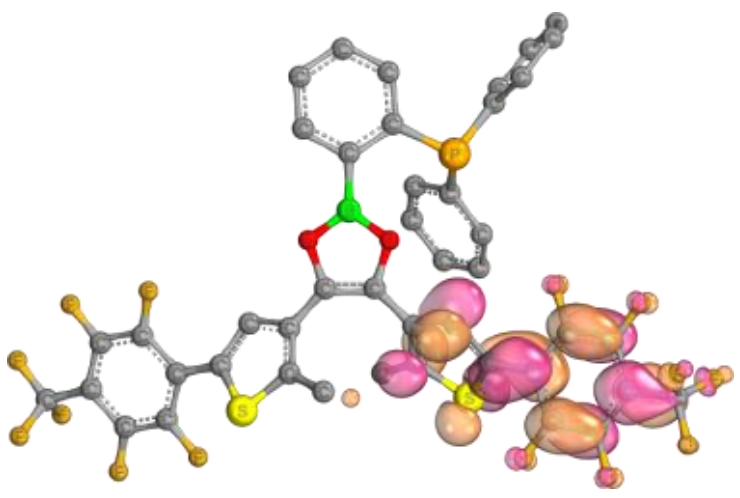

LUMO+1, E = 0.06 eV

1-c

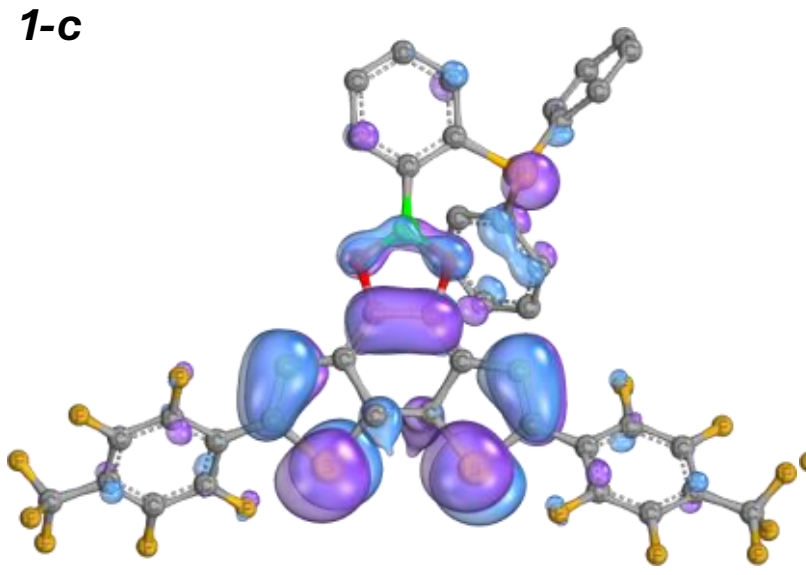

HOMO-2, E = –8.40 eV

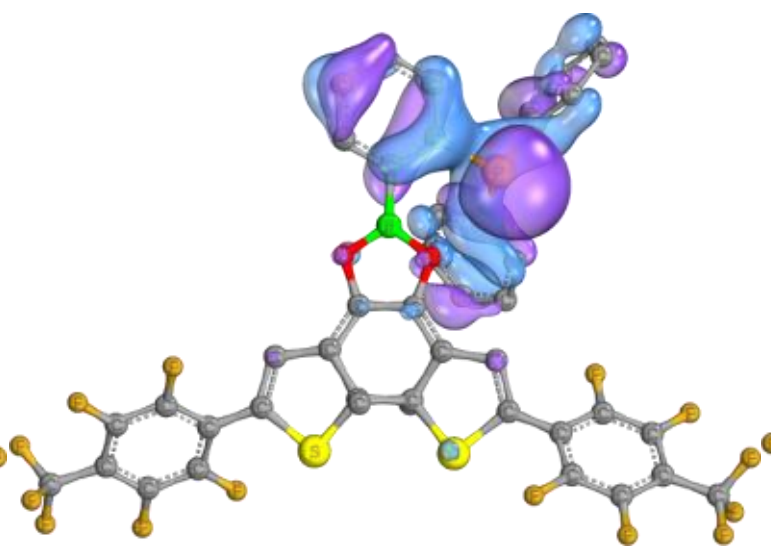

HOMO-1, E = –7.05

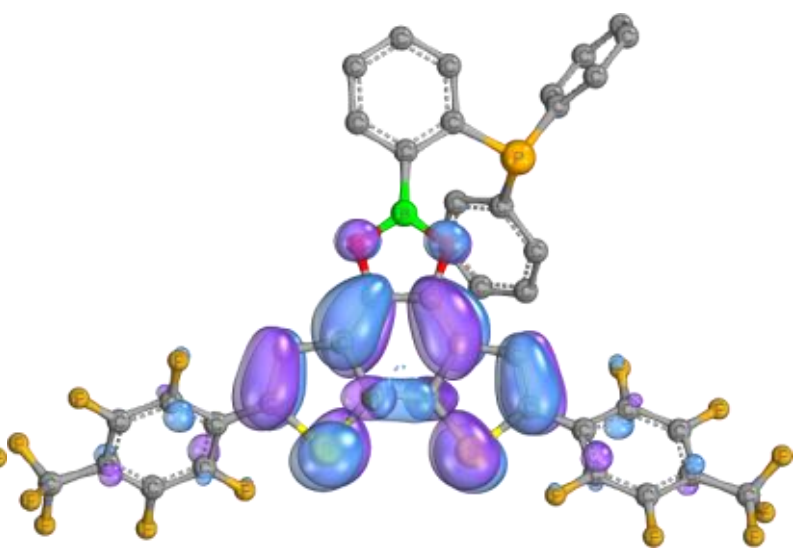

HOMO, E = –8.27 eV

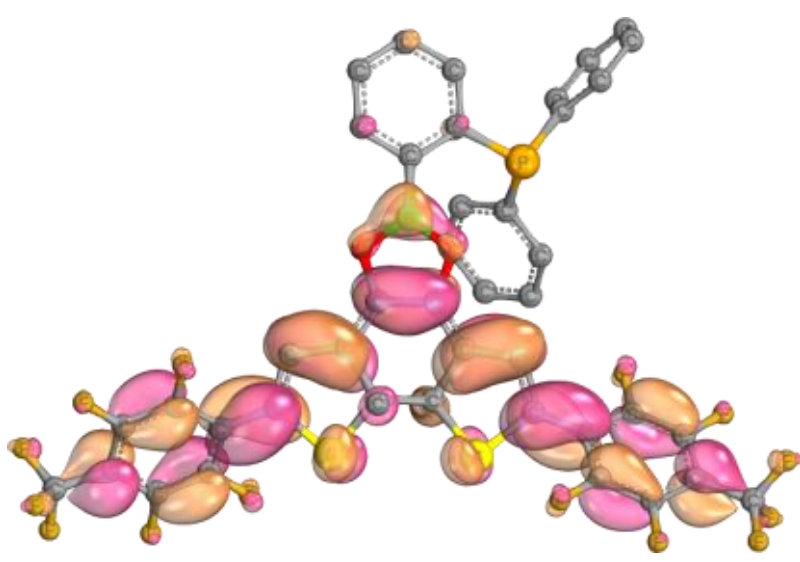

LUMO, E = –0.95 eV

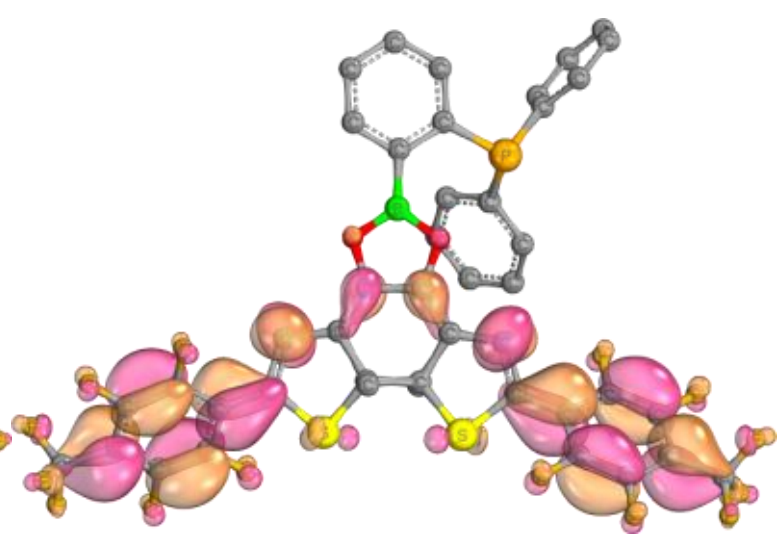

LUMO+1, E = 0.12 eV

2

Table S7. Low energy transitions of the open and closed isomer of **2**. Method: ωB97X-D3/def2-TZVPP/SMD(DCM).

		E / eV	*f*	Orbital Contributions
**open**	S1	4.517	0.884	HOMO → LUMO 32%, HOMO-2 → LUMO 6 %, HOMO →LUMO+1 24%. HOMO-2 → LUMO+1 15%
**closed**	S1	2.581	0.574	HOMO → LUMO 92%
	S2	3.853	0.566	HOMO-1 → LUMO 42%, HOMO → LUMO+1 39%

2-o

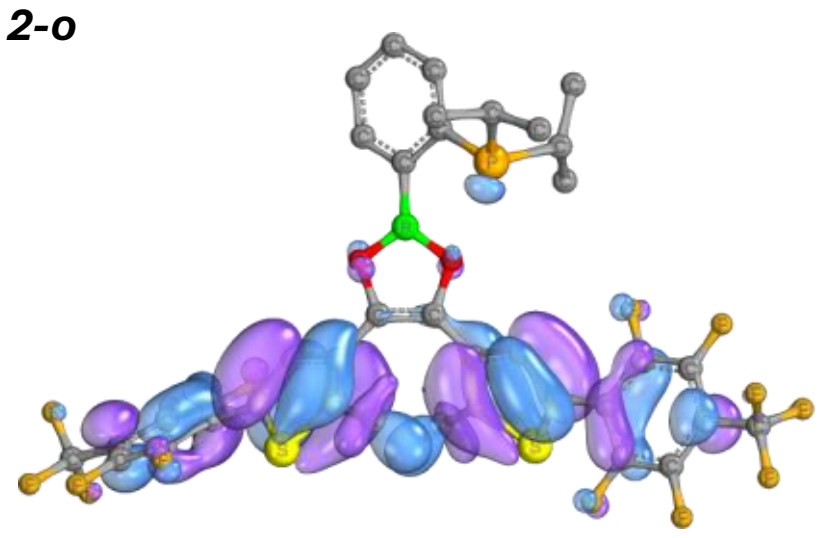

HOMO-2, E = –8.59 eV

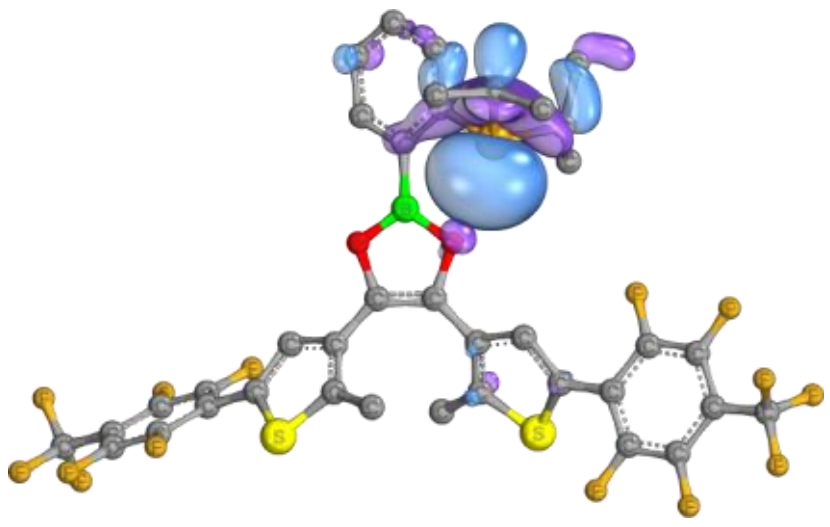

HOMO-1, E = –8.28 eV

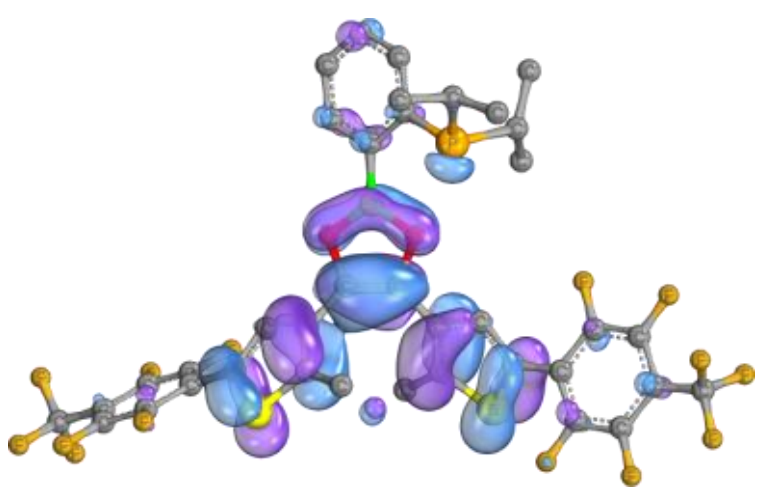

HOMO, E = –7.82 eV

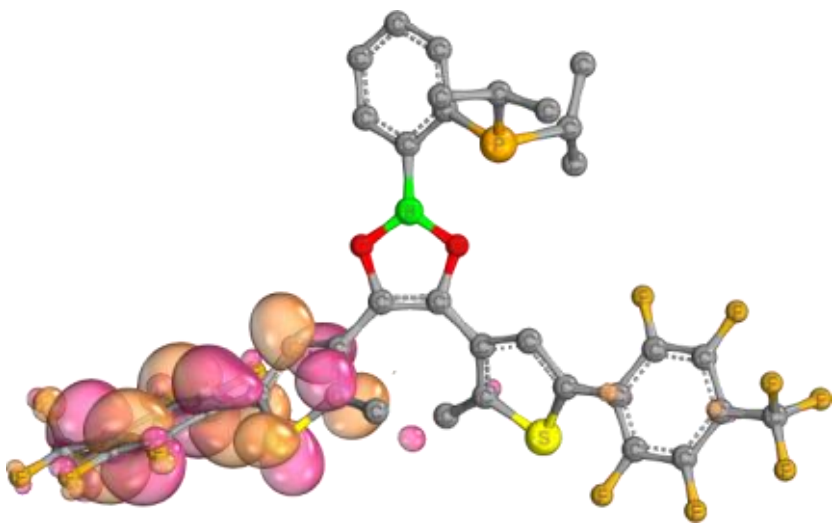

LUMO, E = 0.00 eV

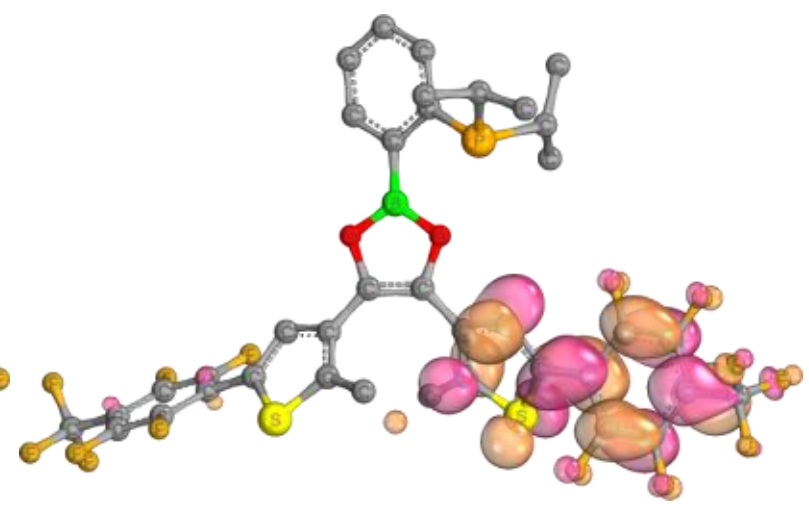

LUMO+1, E = 0.02 eV

2-c

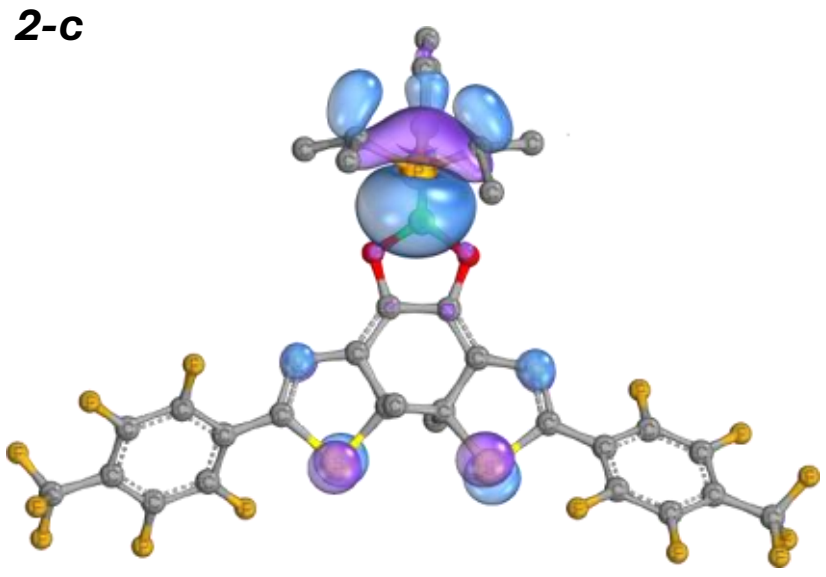

HOMO-2, E = –8.66 eV

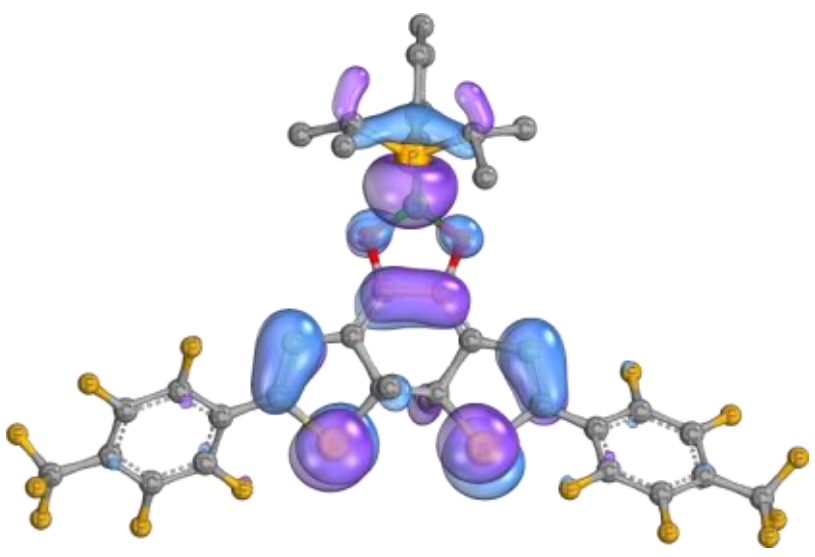

HOMO-1, E = –8.34 eV

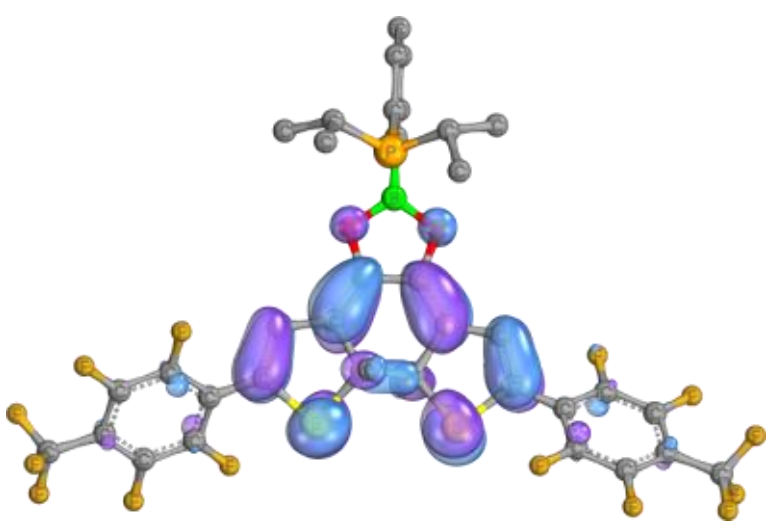

HOMO, E = –7.02 eV

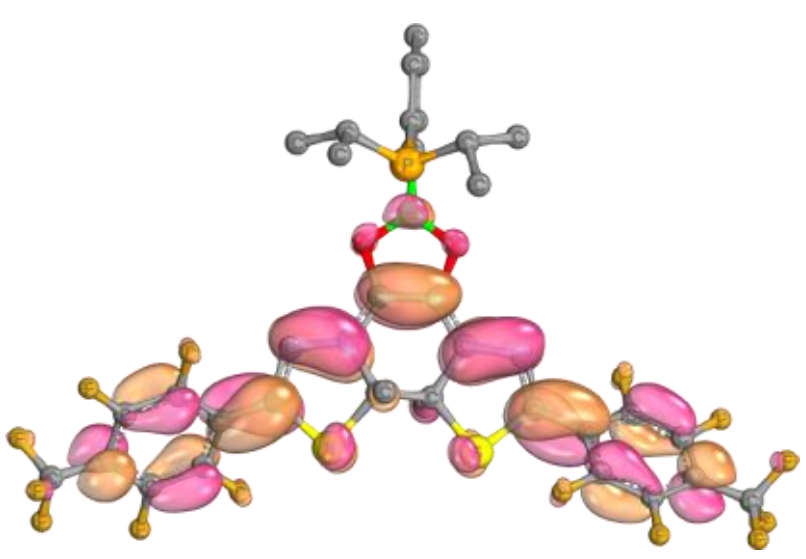

LUMO, E = –0.92 eV

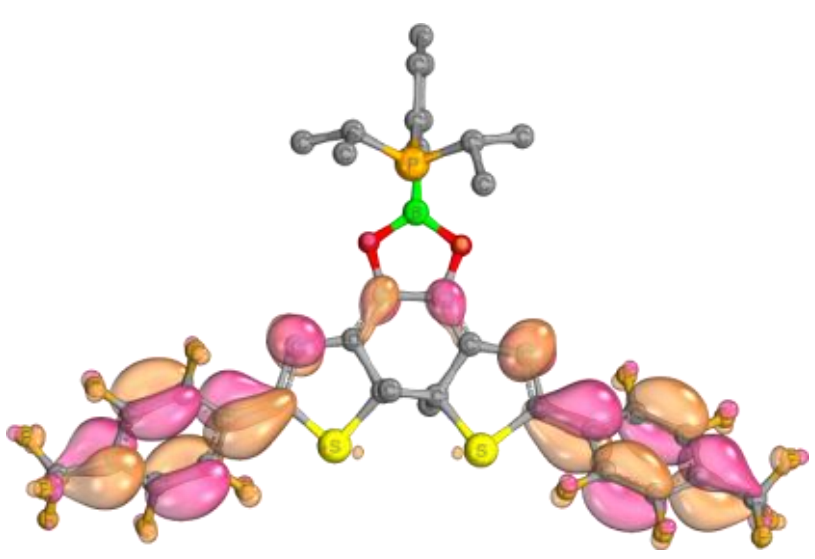

LUMO+1, E = 0.13 eV

2-AuCl

Table S8. Low energy transitions of the open and closed isomer of **2-AuCl**. Method: ωB97X-D3/def2-TZVPP/SMD(DCM).

		E / eV	*f*	Orbital Contributions
**open**	S1	4.208	1.583	HOMO → LUMO 16%, HOMO-1 → LUMO 13 %,<br>HOMO →LUMO+1 37%. HOMO-1 → LUMO+1 17%
**closed**	S1	2.134	0.725	HOMO → LUMO 93%
	S2	3.554	0.666	HOMO-1 → LUMO 42%, HOMO → LUMO+1 39%

2-o-AuCl

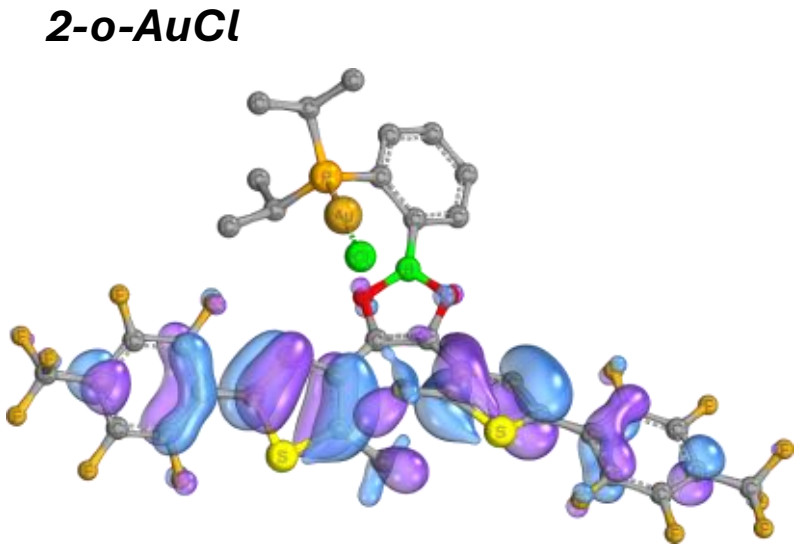

HOMO-1, E = –8.45 eV

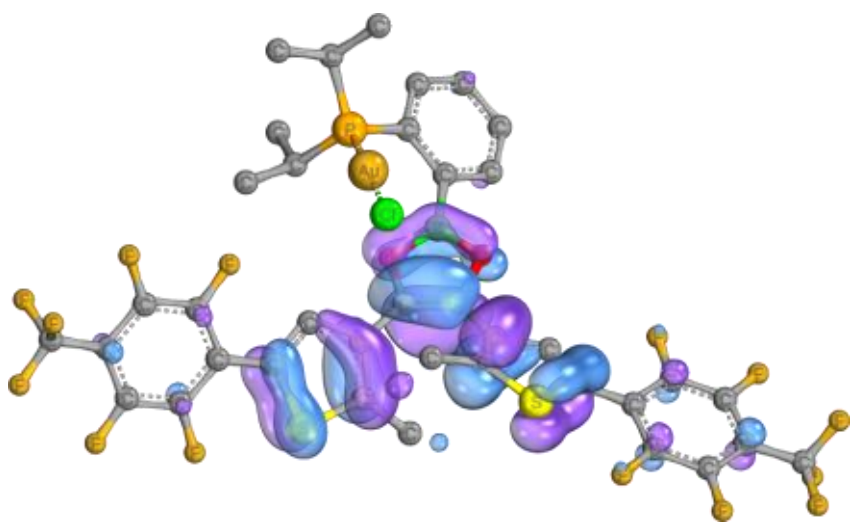

HOMO, E = –7.88 eV

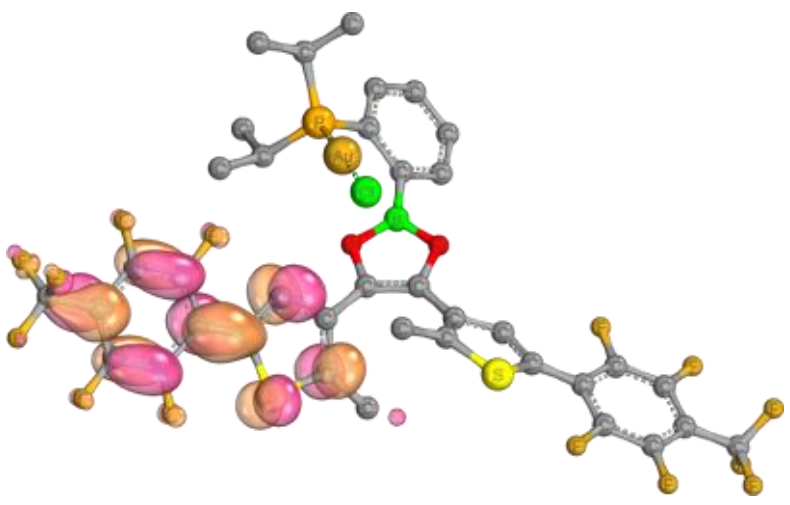

LUMO, E = –0.31 eV

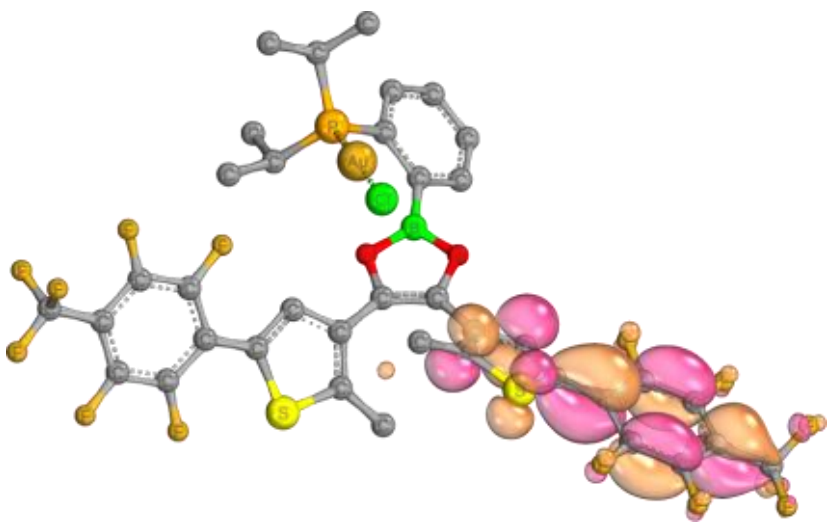

LUMO+1, E = 0.30 eV

2-c-AuCl

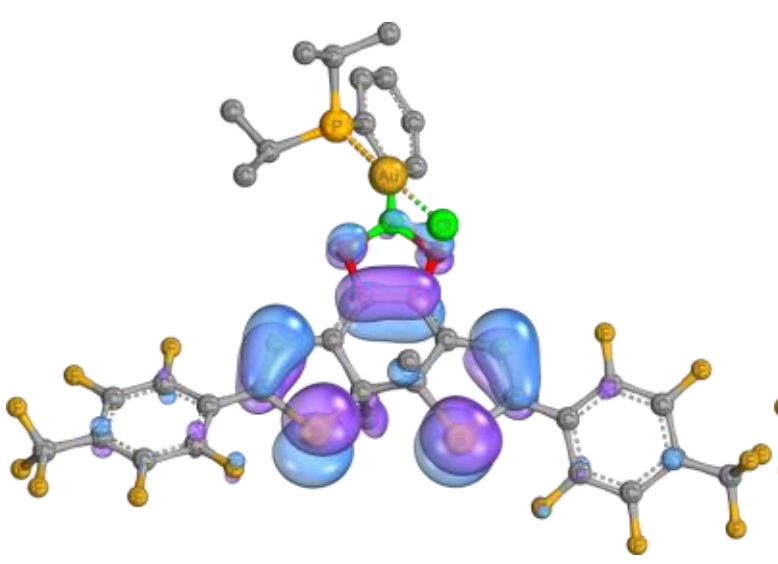

HOMO-1, E = –8.38 eV

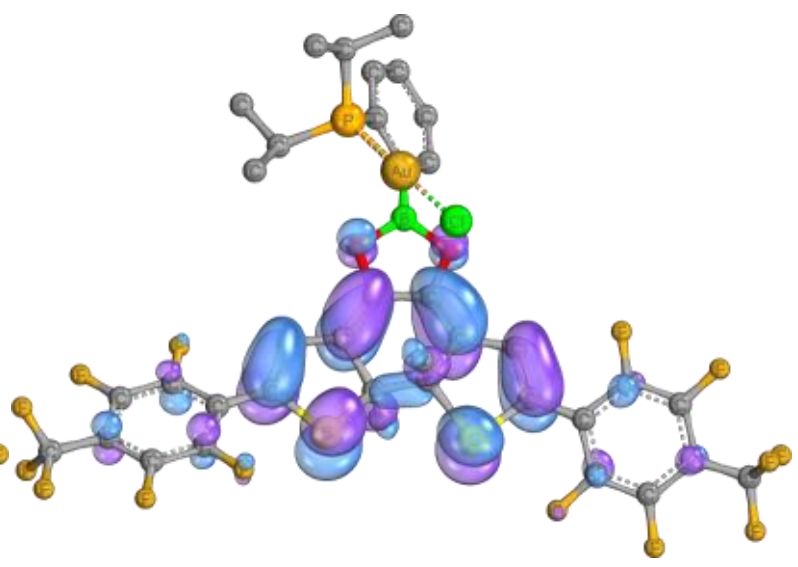

HOMO, E = –6.87 eV

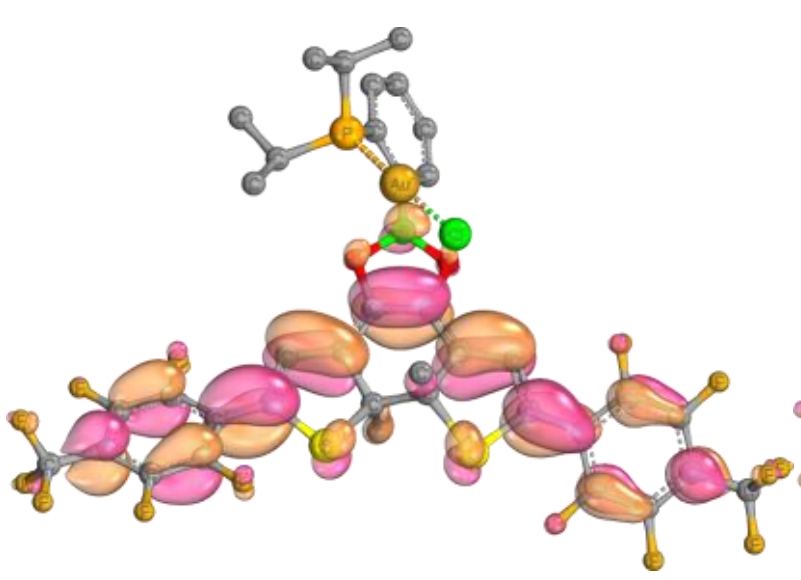

LUMO, E = –1.42 eV

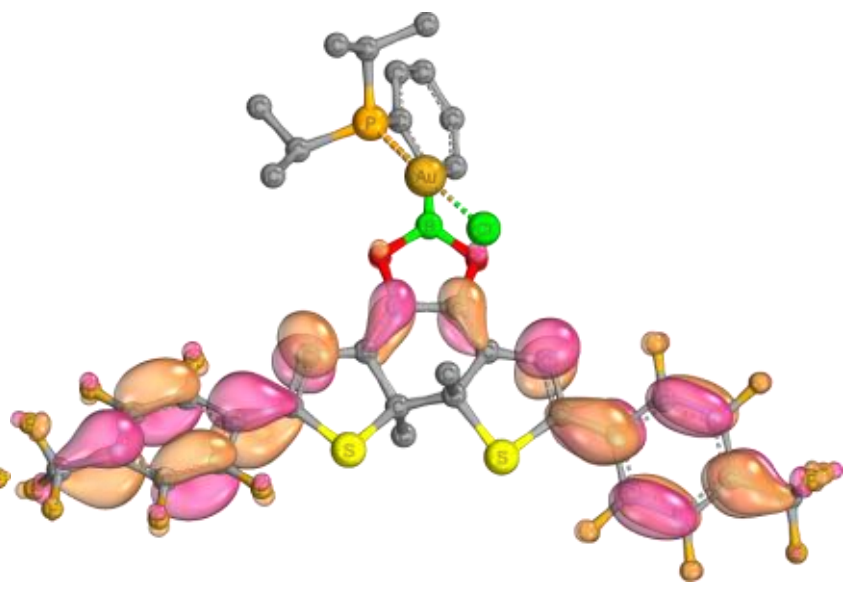

LUMO+1, E = –0.17 eV

2-Rh(CNCy)₂Cl

Table S9. Low energy transitions of the open and closed isomer of **2-Rh(CNCy)$_2$Cl**. Method: ωB97X-D3/def2-TZVPP/SMD(C_6H_6).

		E / eV	*f*	Orbital Contributions
**open**	S1	3.651	0.195	HOMO → LUMO+2 56%, HOMO-1 → LUMO+2 13%, HOMO-2 → LUMO+2 12%
	S4	4.283	0.225	HOMO → LUMO 55%, HOMO-2 → LUMO 14%
**closed**	S1	2.581	0.574	HOMO → LUMO 92%
	S2	3.853	0.566	HOMO-1 → LUMO 42%, HOMO → LUMO+1 39%, HOMO-1→ LUMO+2 4%

2-o-Rh(CNCy)$_2$Cl, Molecular Orbitals

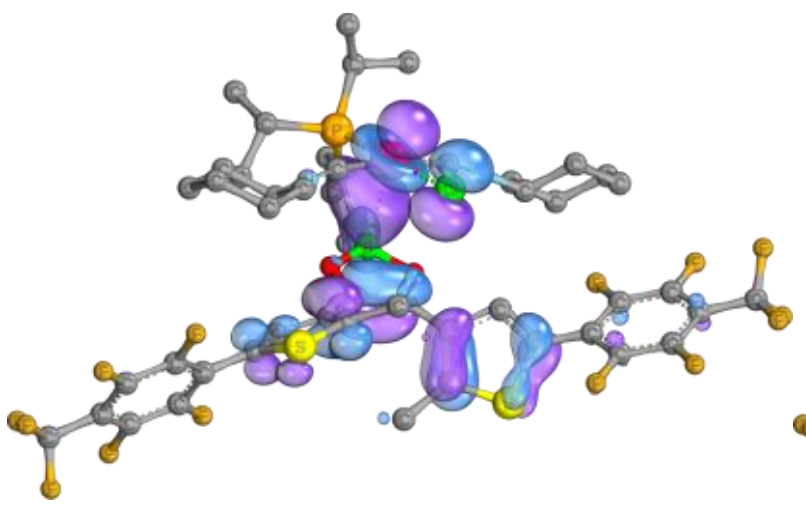

HOMO-2, E = 8.05– eV

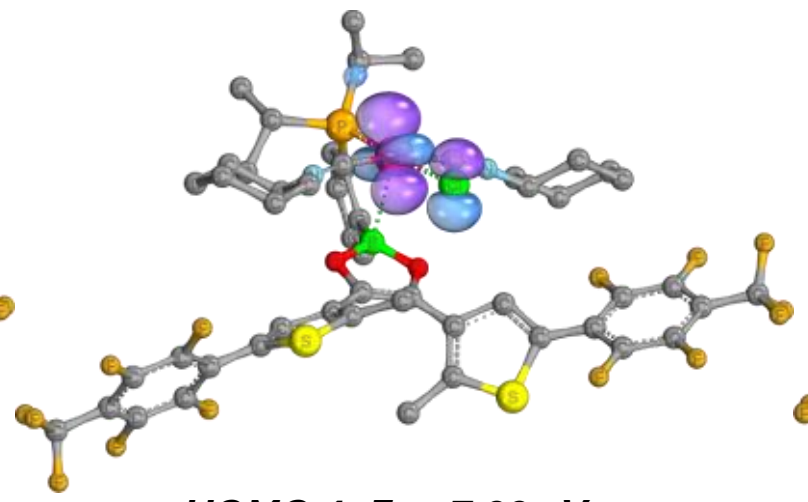

HOMO-1, E = –7.99 eV

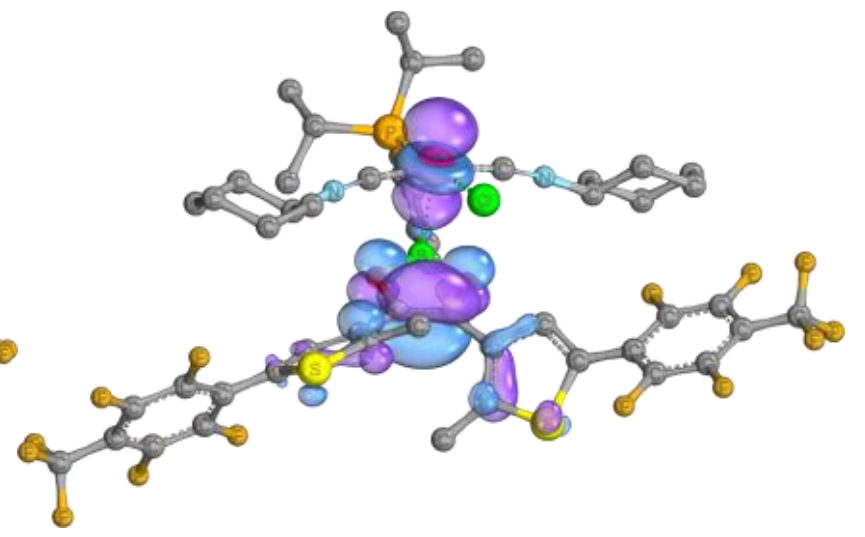

HOMO, E = –7.26 eV

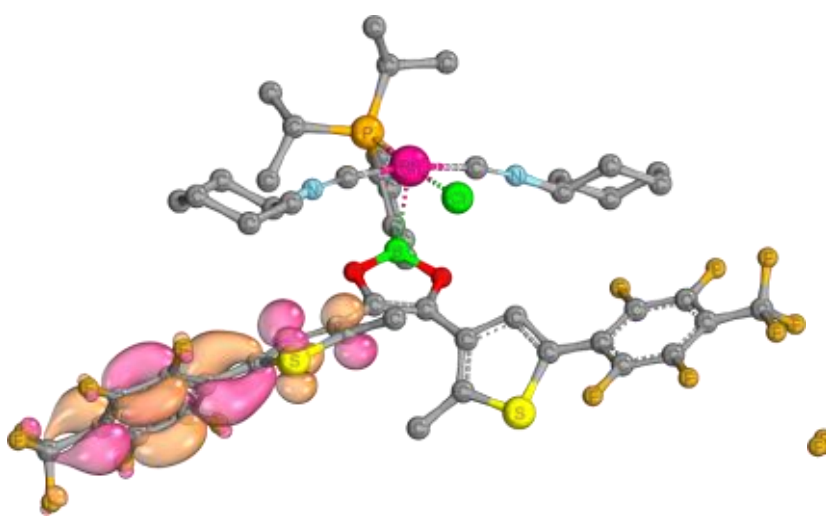

LUMO, E = –0.10 eV

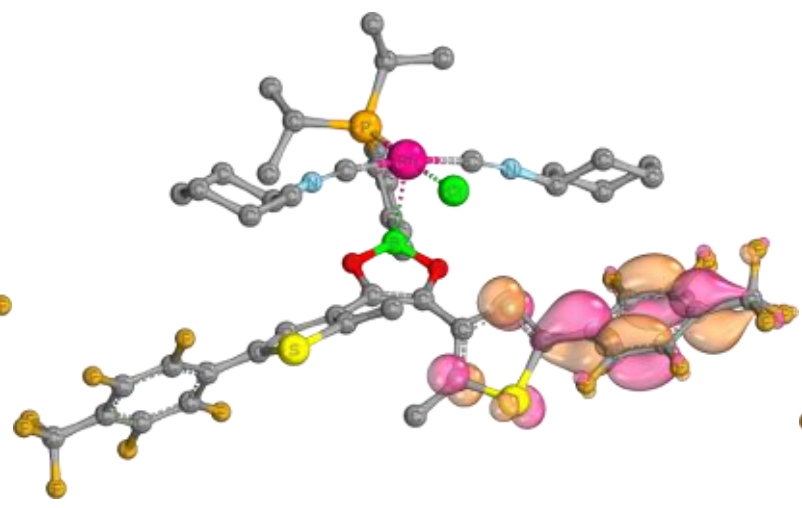

LUMO+1, E = 0.08 eV

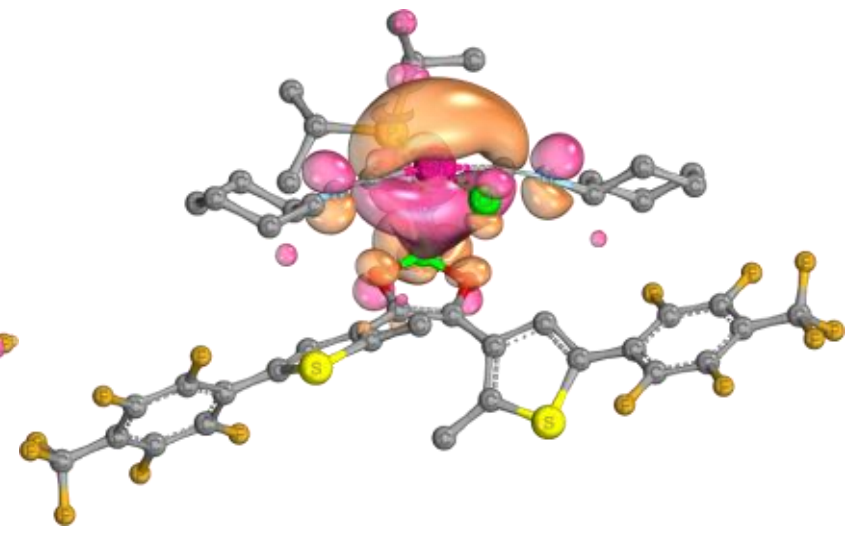

LUMO+2, E = 0.86 eV

2-o-Rh(CNCy)$_2$Cl, Natural Transition Orbitals

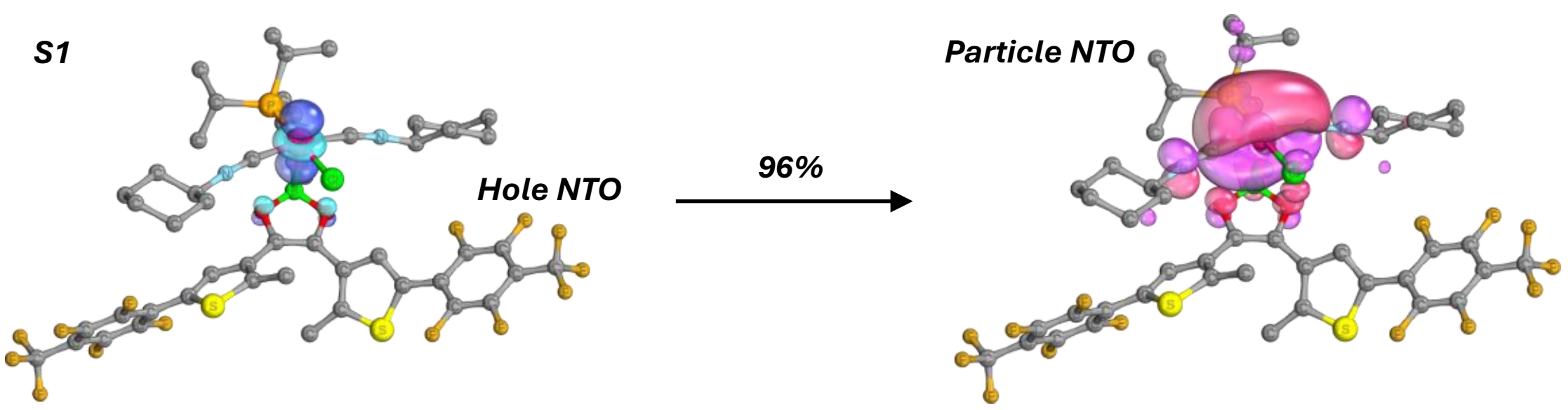

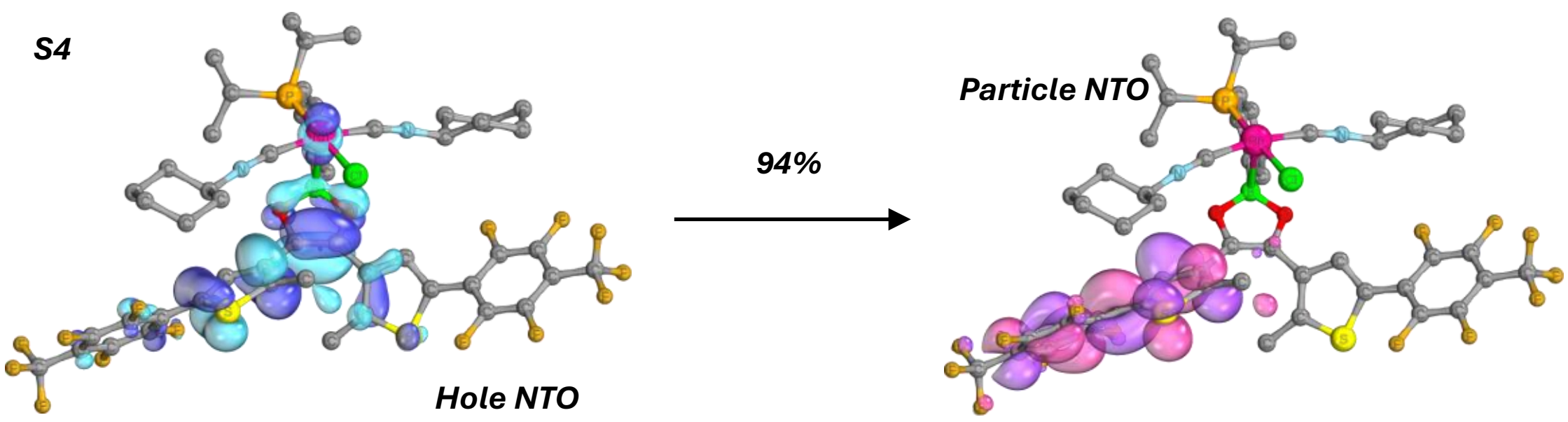

2-c-Rh(CNCy)$_2$Cl, Molecular Orbitals

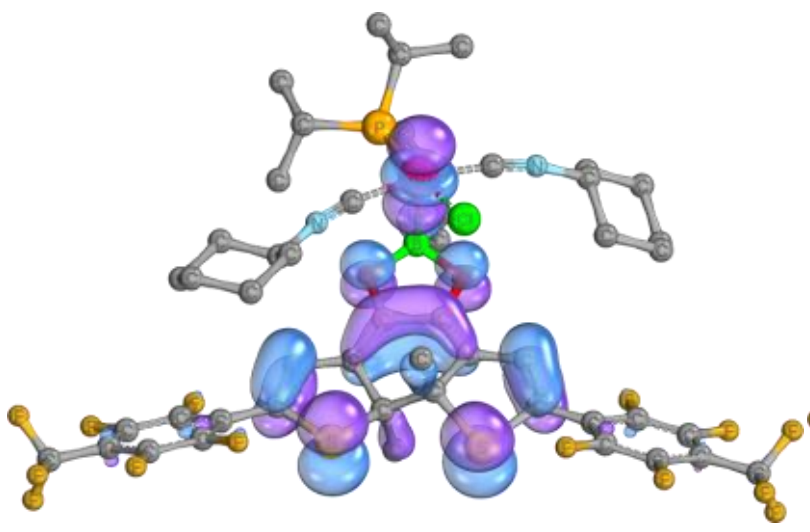

HOMO-1, E = –7.78 eV

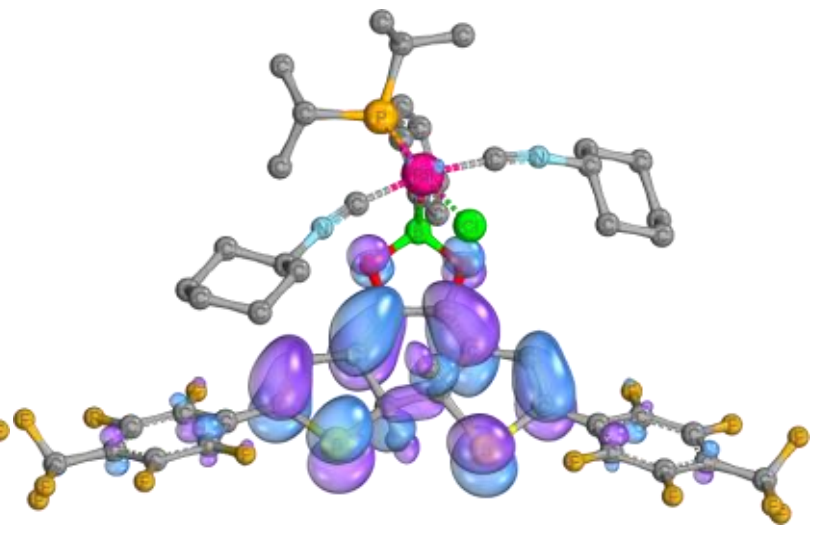

HOMO, E = –6.73 eV

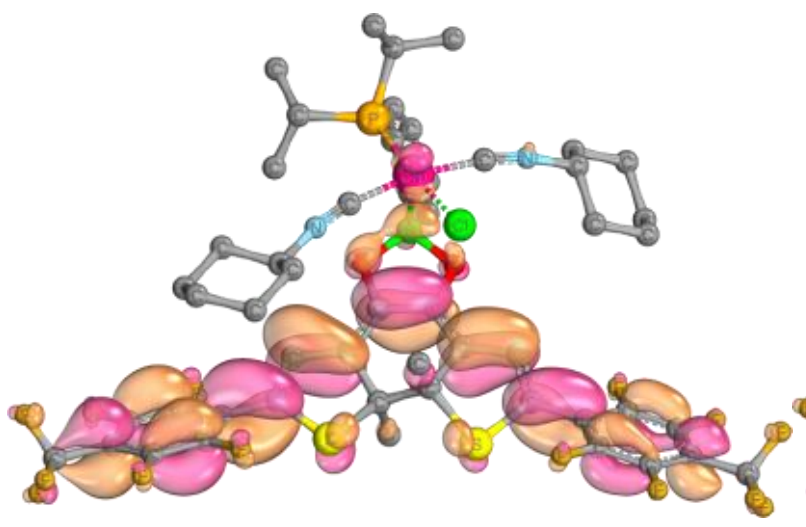

LUMO, E = –0.74 eV

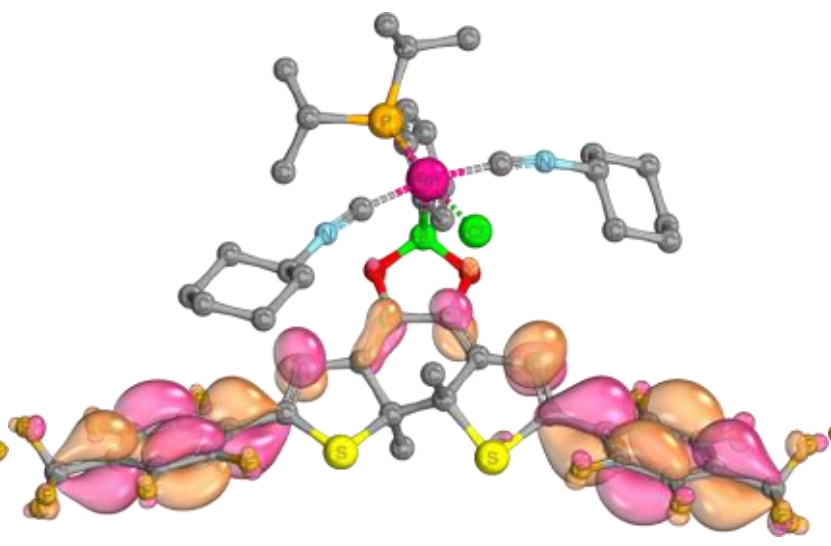

LUMO+1, E = 0.19 eV

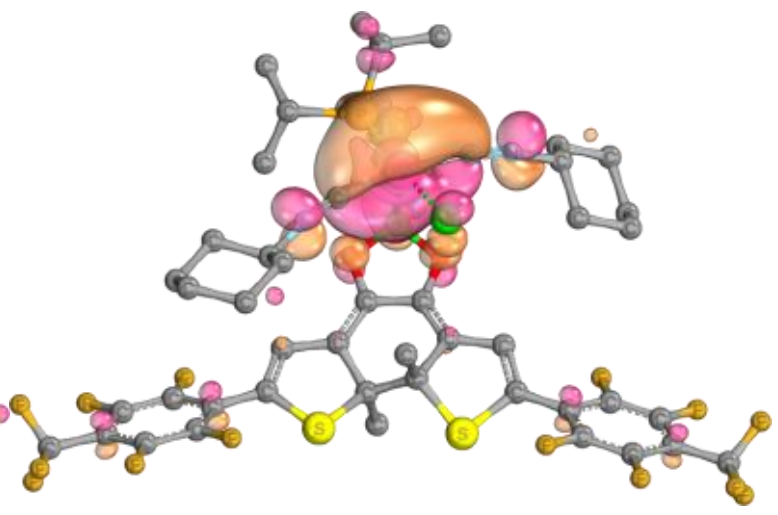

LUMO+2, E = 0.47 eV

2-c-Rh(CNCy)$_2$Cl, Natural Transition Orbitals

S1

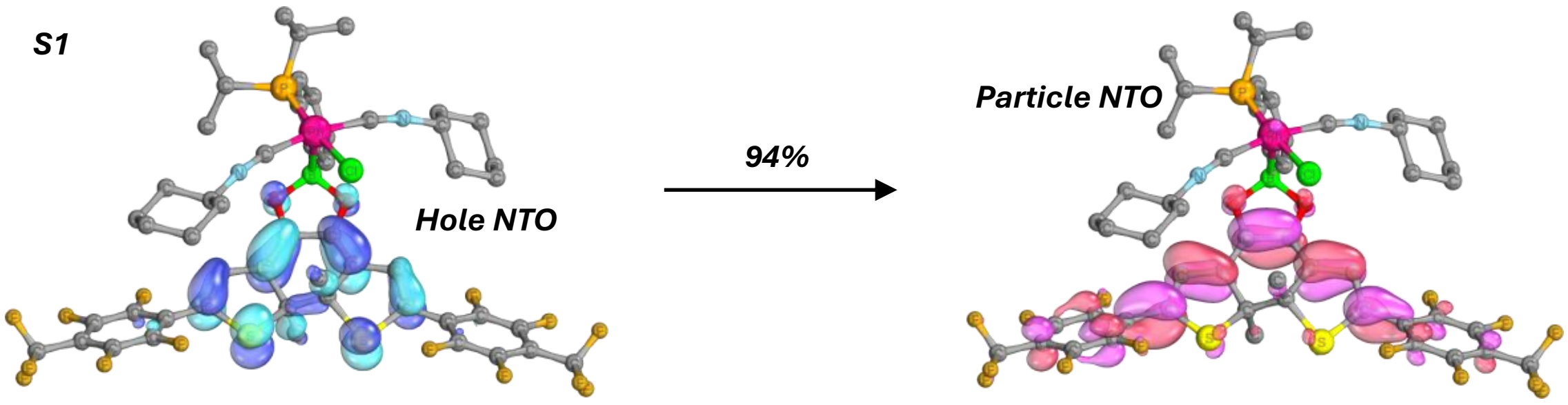

S2

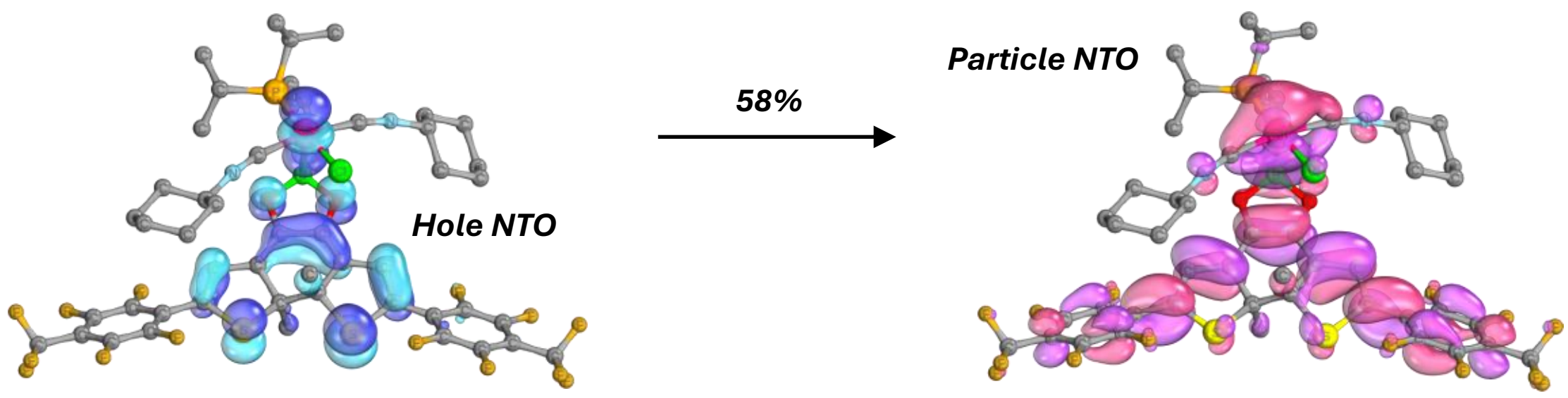

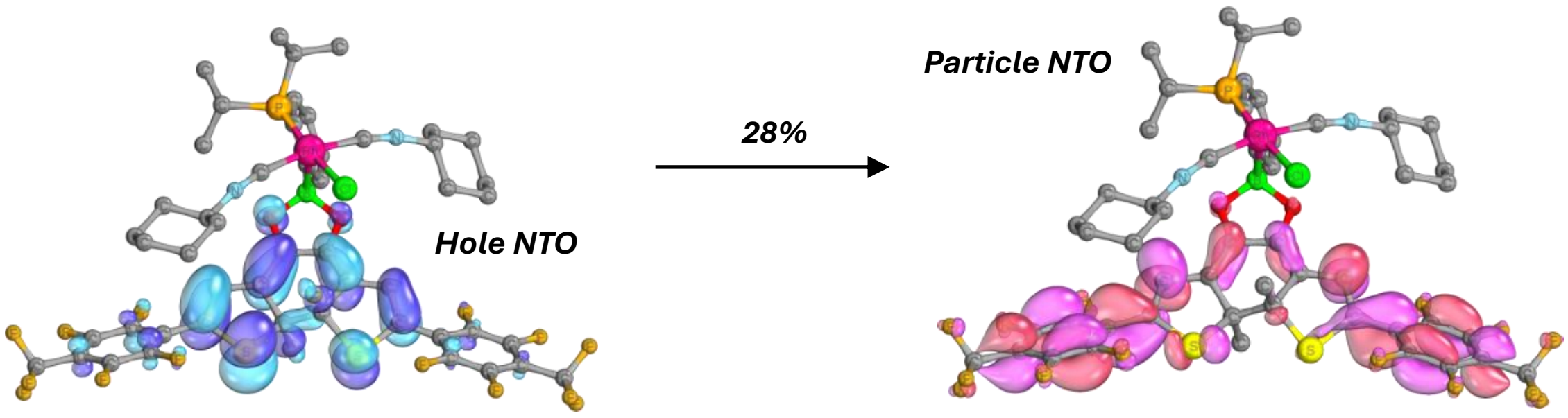

4 Reactivity Studies

4.1 Photocyclization (1)

4.1.1 1, Benzene

1-o (4.5 mg) was dissolved in C_6D_6 (0.5 ml). Cyclooctane was added as an internal standard. The sample was irradiated at 300 nm. Based on internal integration, a 94/6 ratio of **1-c/1-o** was obtained after 180 minutes of irradiation, with a combined integral of 97% compared to pre-irradiation (**1-o** and **1-c**). Characteristic ^{1}H NMR Resonances and ^{31}P Resonances are marked below.

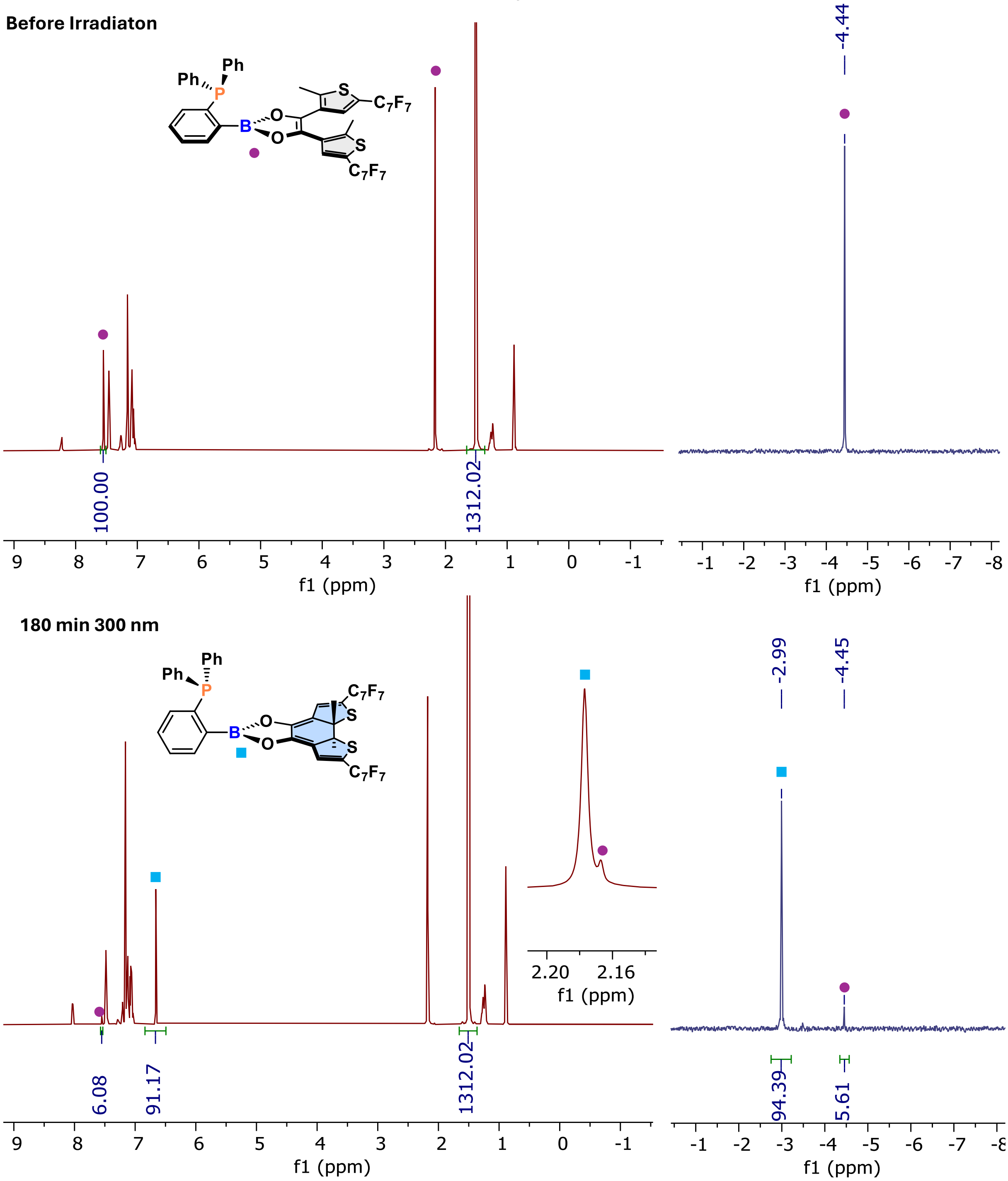

Figure S52. NMR spectra of **1** in C_6D_6 before Irradiation (**1-o**, top) and after conversion to **1-c** (bottom). Left: ^{1}H NMR (600 MHz); Right: ^{31}P NMR (243 MHz). Conversion was determined by integration of thiophene CH signals against cyclooctane.

4.1.2 1, DCM

1-o (8.8 mg) was dissolved in CD_2Cl_2 (0.5 ml). Cyclooctane was added as an internal standard. The sample was irradiated at 300 nm. Based on internal integration, a 92/8 ratio of **1-c/1-o** was obtained after 210 minutes of irradiation, with a combined integral of 98% compared to pre-irradiation (**1-o** and **1-c**). Characteristic ^{1}H NMR Resonances and ^{31}P Resonances are marked below.

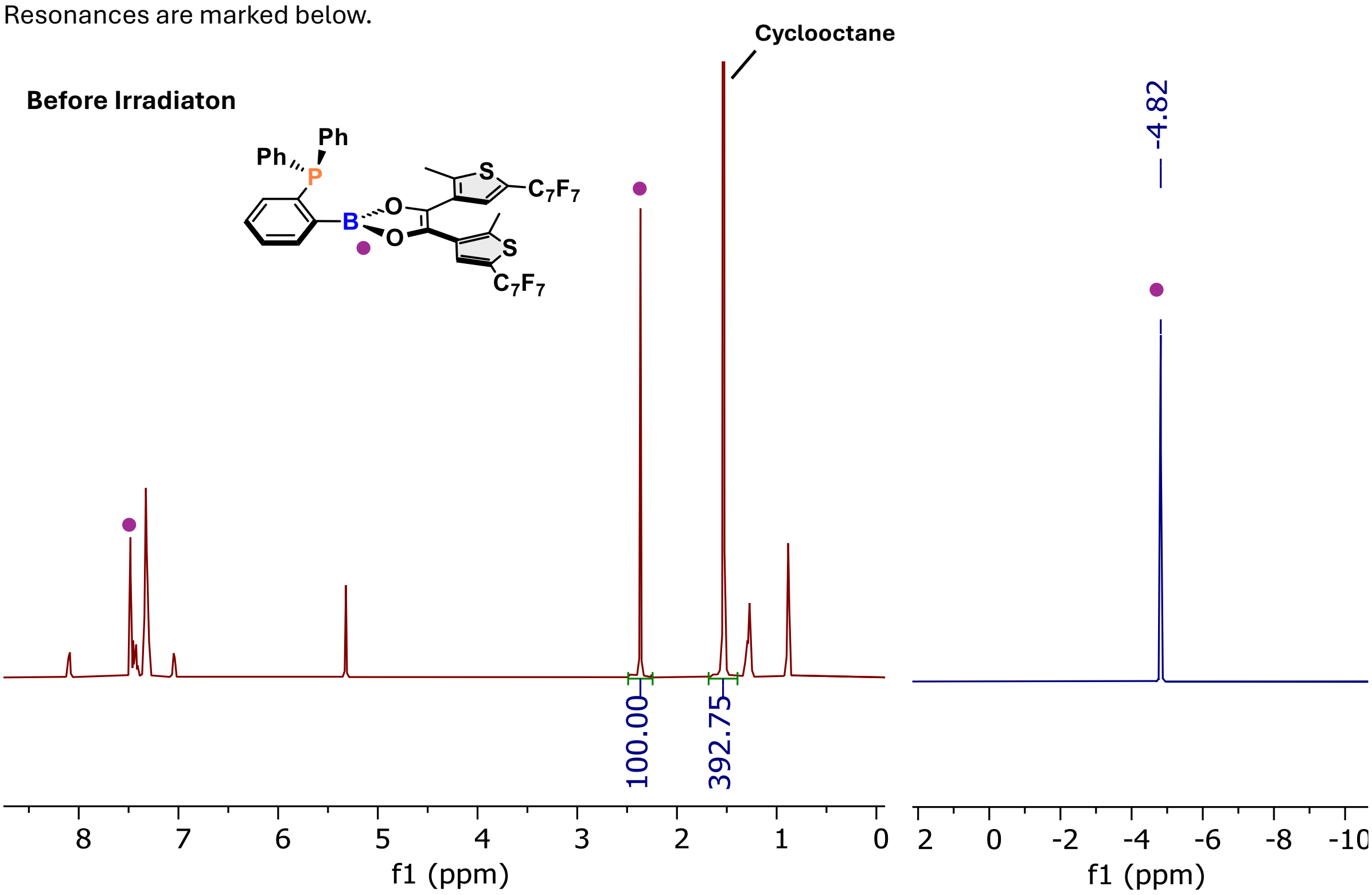

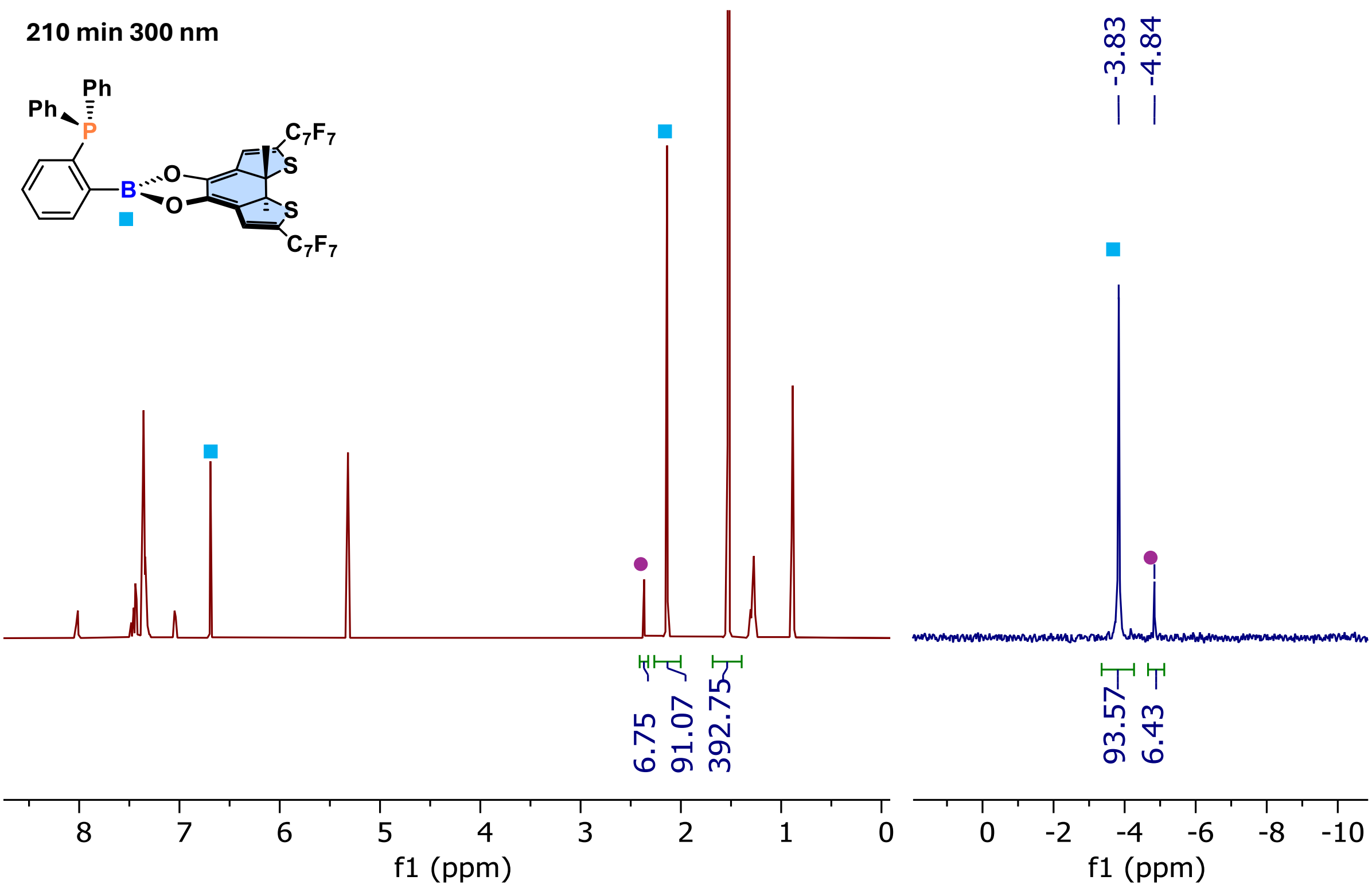

Figure S53. NMR spectra of **1** in CD_2Cl_2 before Irradiation (**1-o**, top) and after conversion to **1-c** (bottom). Left: ^{1}H NMR (600 MHz); Right: ^{31}P NMR (243 MHz). Conversion was determined by integration of thiophene CH_3 signals against cyclooctane.

4.2 Photocyclization (2)

4.2.1 2, Benzene

2-o (5.2 mg) was dissolved in C_6D_6 (0.5 ml). Cyclooctane was added as an internal standard. The sample was irradiated at 300 nm. Based on internal integration, 94/6 ratio of **2-c/2-o** was obtained after 180 minutes of irradiation, with a combined integral of 93% compared to pre-irradiation (**2-o** and **2-c**). Characteristic ^{1}H NMR Resonances and ^{31}P Resonances are marked below.

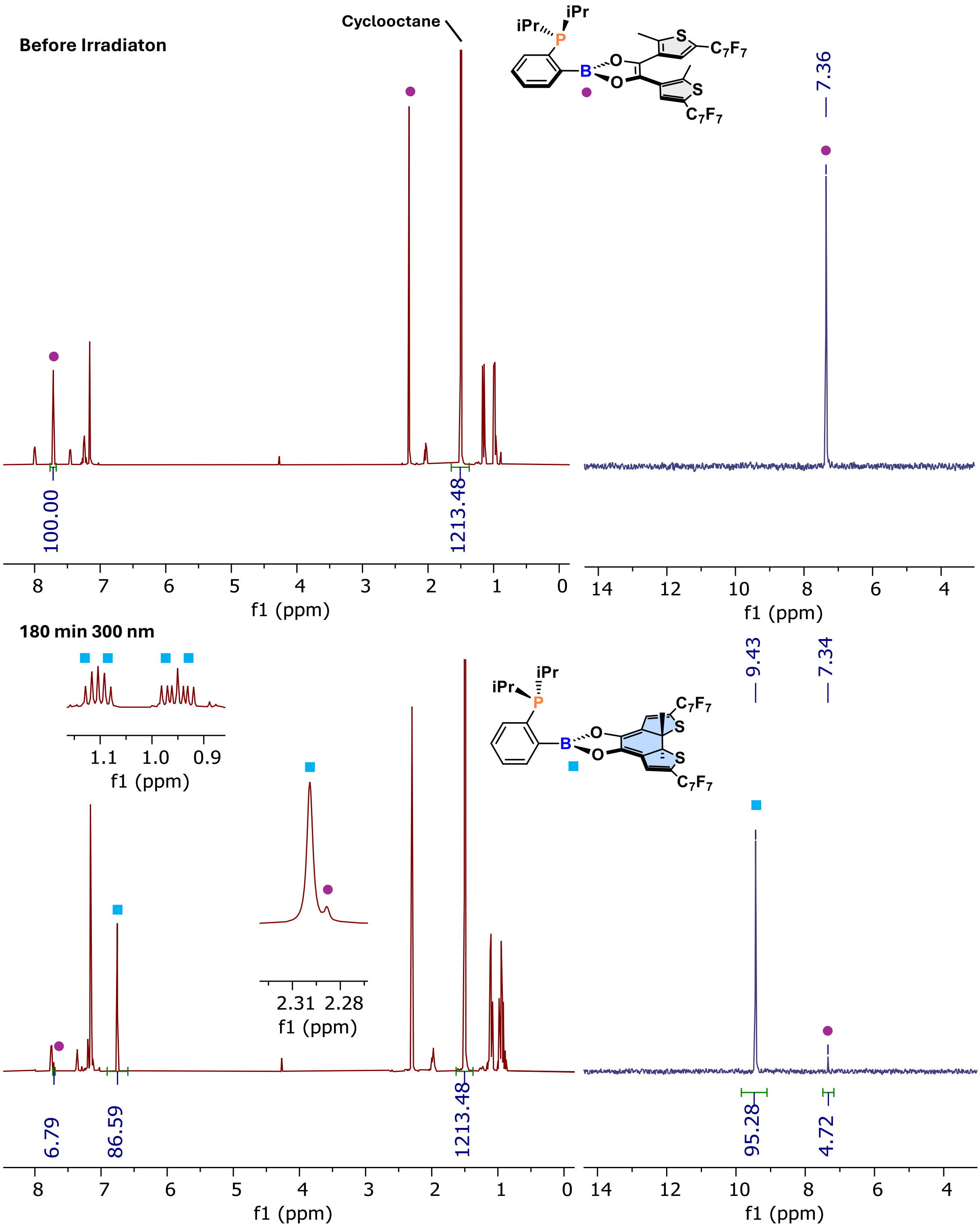

Figure S54. NMR spectra of **2** in C_6D_6 before Irradiation (**2-o**, top) and after conversion to **2-c** (bottom). Left: ^{1}H NMR (600 MHz); Right: ^{31}P NMR (243 MHz). Conversion was determined by integration of thiophene CH signals against cyclooctane.

4.2.2 2, DCM

2-o (10.2 mg) was dissolved in CD_2Cl_2 (0.5 ml). Cyclooctane was added as an internal standard. The sample was irradiated at 300 nm. Based on internal integration, an 81/19 ratio of **2-c/2-o** was obtained after 290 minutes of irradiation, with a combined integral of 95% compared to pre-irradiation (**2-o** and **2-c**). Characteristic ^{1}H NMR Resonances and ^{31}P Resonances are marked below.

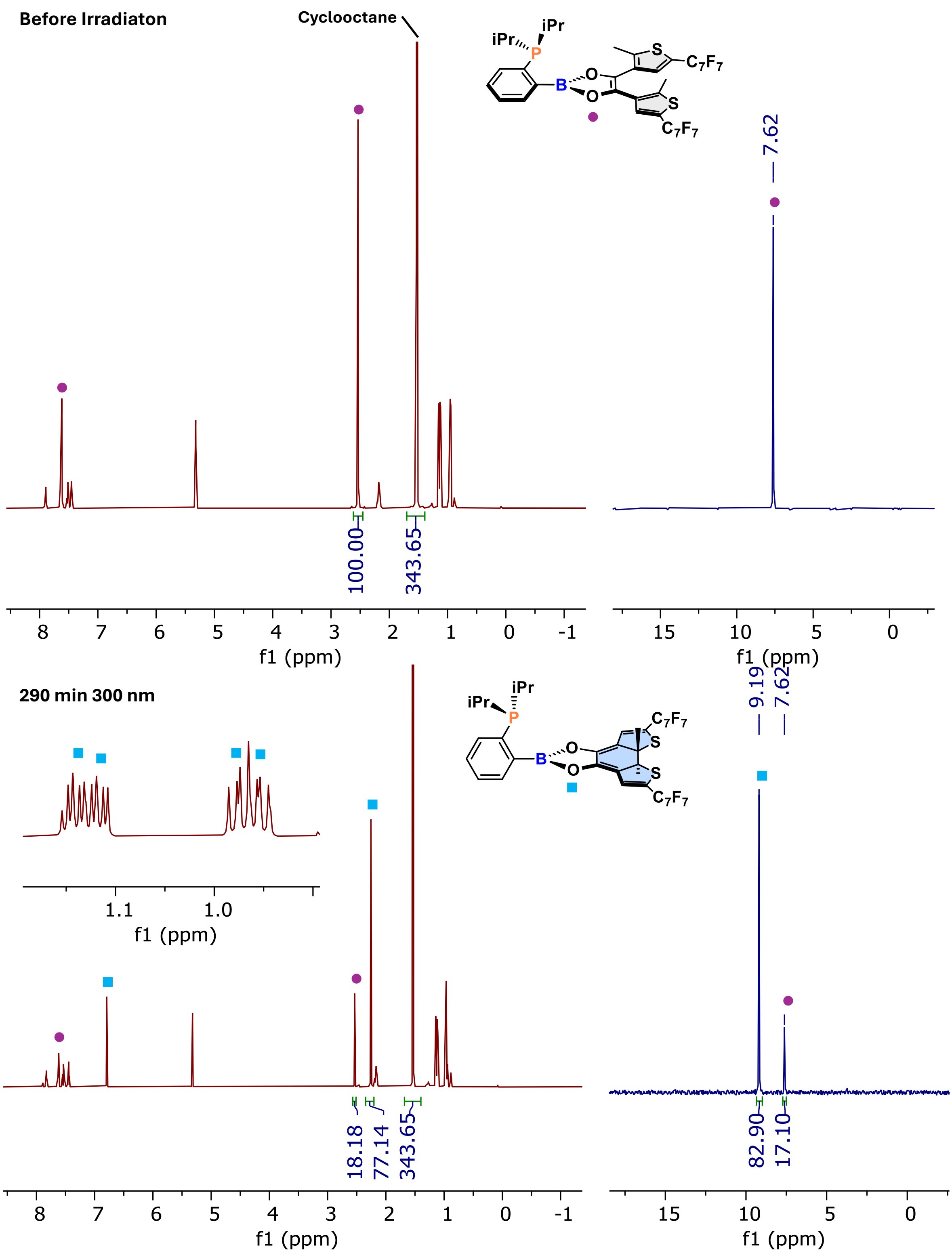

Figure S55. NMR spectra of **2** in CD_2Cl_2 before Irradiation (**2-o**, top) and after conversion to **2-c** (bottom). Left: ^{1}H NMR (600 MHz); Right: ^{31}P NMR (243 MHz). Conversion was determined by integration of thiophene CH_3 signals against cyclooctane.

4.3 Thermal Stability of the Closed Isomers

To evaluate the thermal stability of 1-c and 2-c in the dark, 1-o or 2-o were dissolved in C_6D_6 together with cyclooctane as an internal standard. The mixtures were irradiated at 365 nm for 30 minutes, and ^{1}H NMR spectra were measured over the course of 14 hours. For both compounds, no thermal ring-opening was observed.

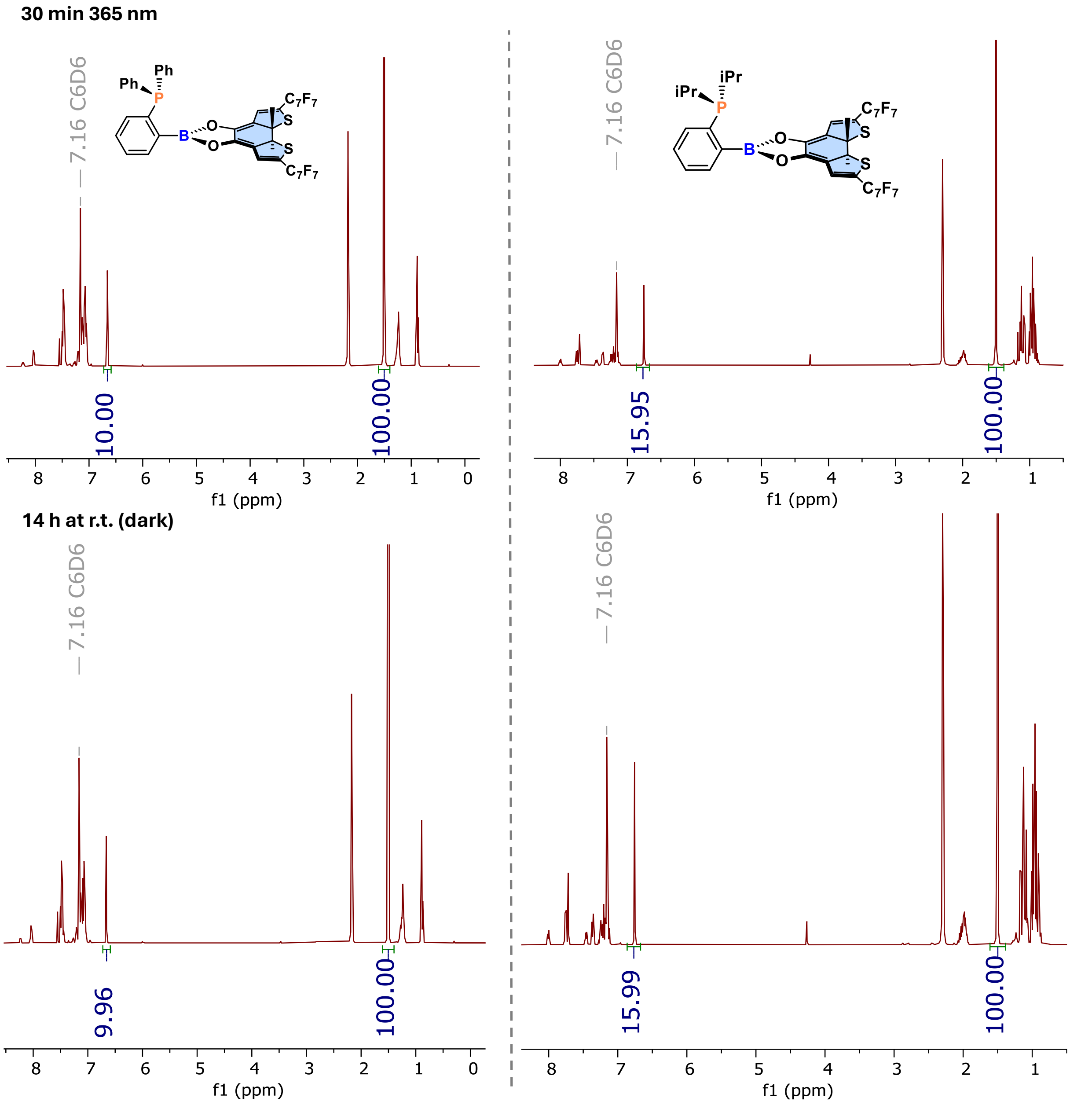

Figure S56. NMR spectra of **1** (left) and **2** (right) in C_6D_6, directly after UV irradiation (top) and after 14 hours at room temperature. Thiophene CH signals were integrated against cyclooctane.

4.4 Substrate Activation (1)

4.4.1 Benzaldehyde

1-o (11.1 mg) and benzaldehyde (1.5 eq) were dissolved in 0.5 ml C_6D_6. NMR spectra were taken pre-irradiation, after conversion to the closed form and again after ring-opening. Cyclization was induced at 300 nm, cycloreversion with broadband visible light. Due to the complexity of the ^{1}H NMR spectrum resulting from diastereomers and line broadening, analysis was focussed on ^{31}P NMR data. Nevertheless, characteristic signals were marked below.

Before Irradiation

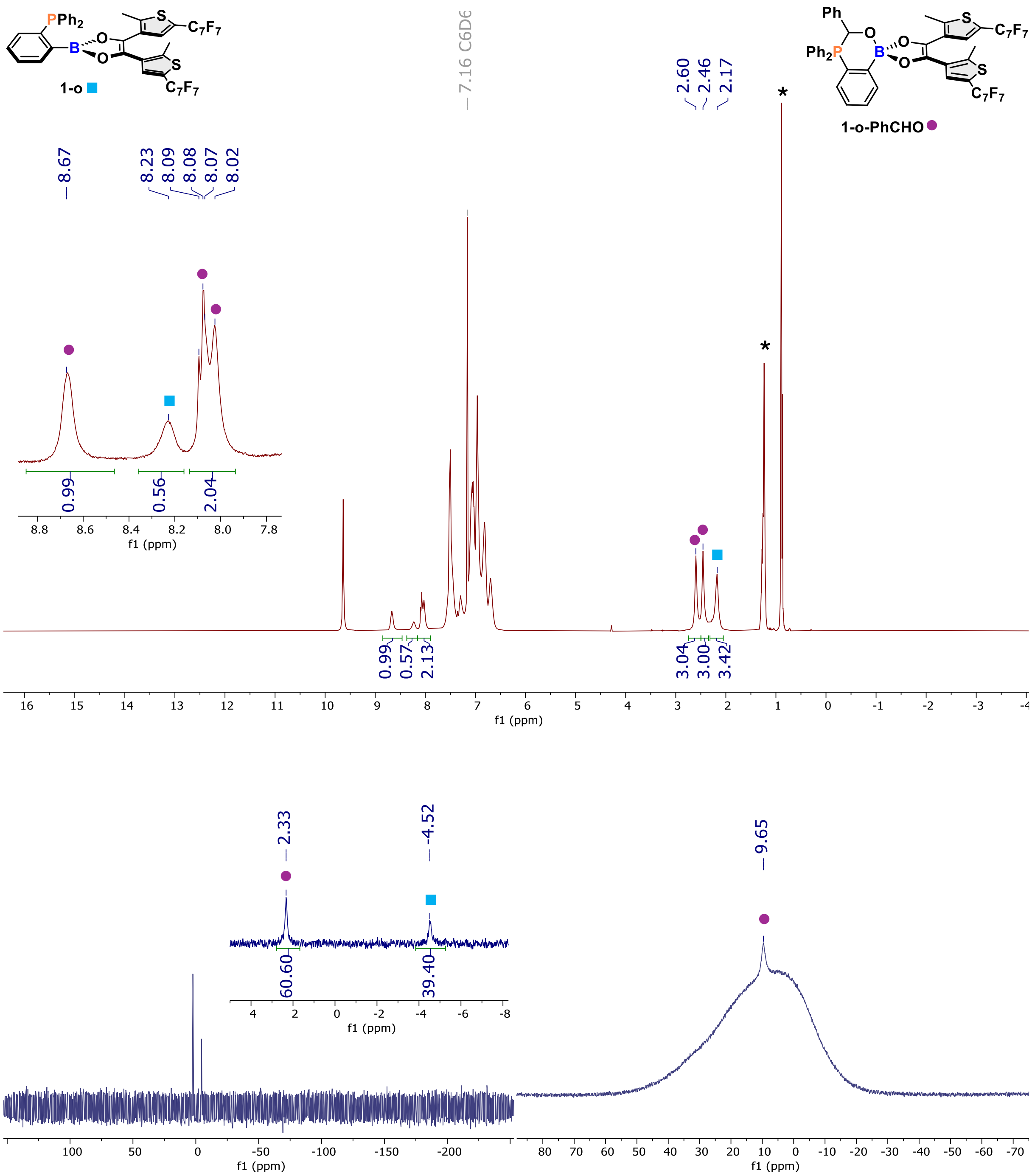

Figure S57. NMR spectra of a mixture of **1-o** and benzaldehyde in C_6D_6.Top: ^{1}H NMR (600 MHz), integrals set for **1-o-PhCHO**; Bottom left: ^{31}P NMR (243 MHz), total integral set to 100; Bottom Right: ^{11}B NMR (193 MHz). *n-hexane

2h 300 nm

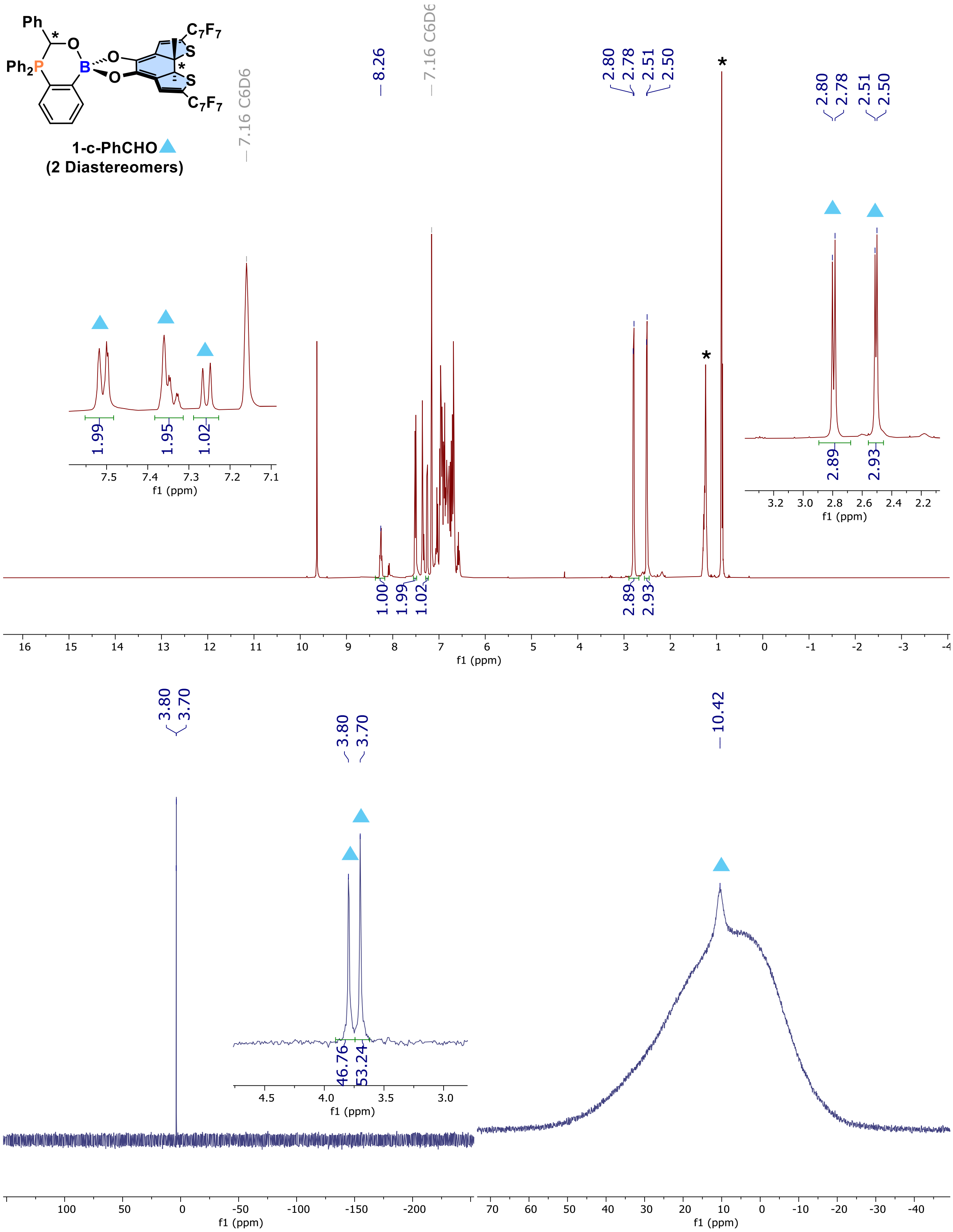

Figure S58. NMR spectra after irradiation at 300 nm, giving **1-c-PhCHO** as two diastereomers and residual benzaldehyde in C_6D_6. Top: ^{1}H NMR (600 MHz); Bottom left: ^{31}P NMR (243 MHz), total integral set to 100; Bottom Right: ^{11}B NMR (193 MHz).

30 minutes VIS

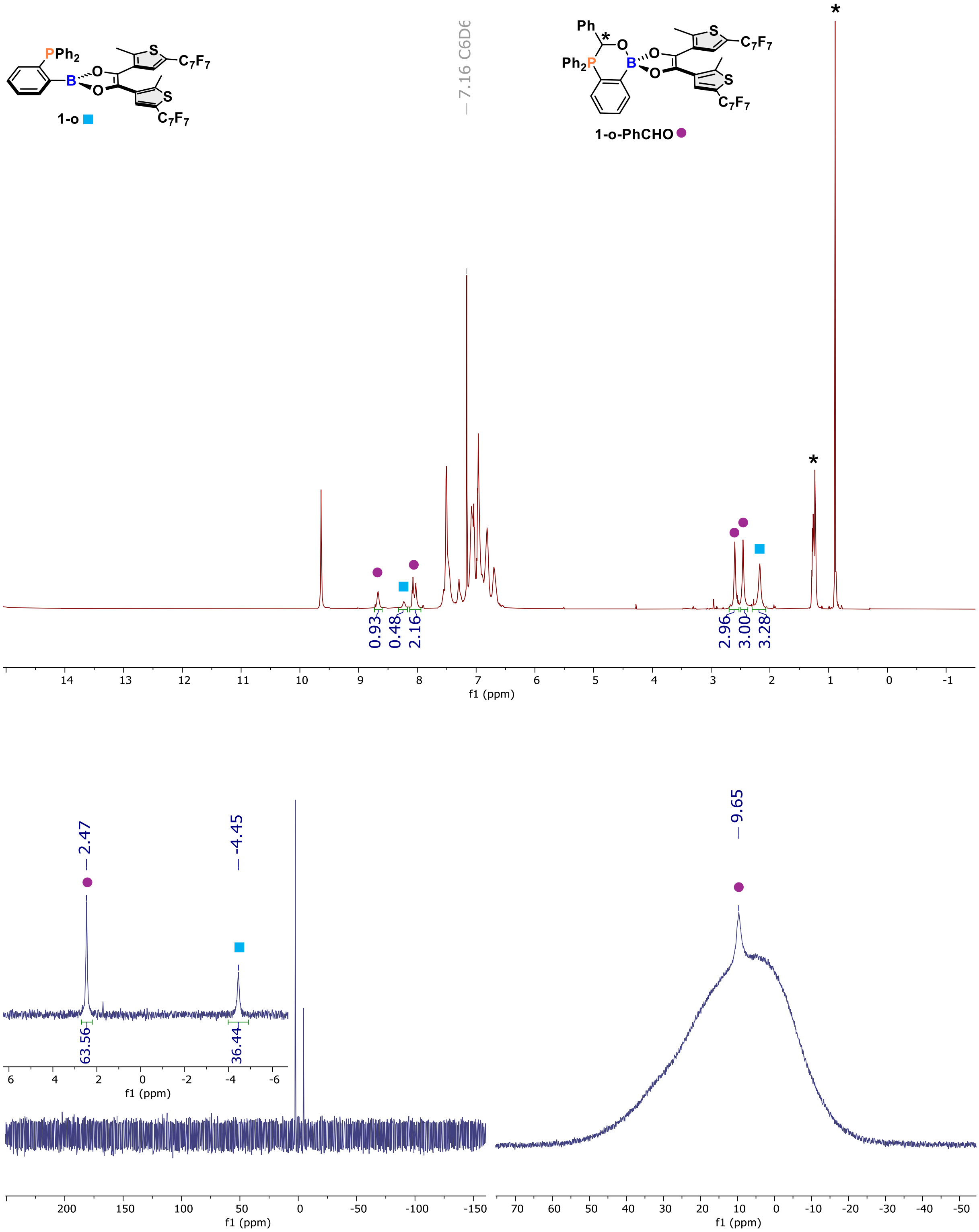

Figure S59. NMR spectra in C_6D_6 after broadband VIS irradiation, restoring **1-o** and the initial equilibirum. Top: ^{1}H NMR (600 MHz), integrals set for **1-o-PhCHO**; Bottom left: ^{31}P NMR (243 MHz), total integral set to 100; Bottom Right: ^{11}B NMR (193 MHz).

4.4.2 Phenyl Isothiocyanate

1-o (9.7 mg) and PhNCS (2 eq) were dissolved in 0.5 ml C_6D_6. NMR spectra were taken pre-irradiation, after conversion to the closed form and again after ring-opening. Cyclization was induced at 300 nm, cycloreversion with broadband visible light.

Before Irradiation

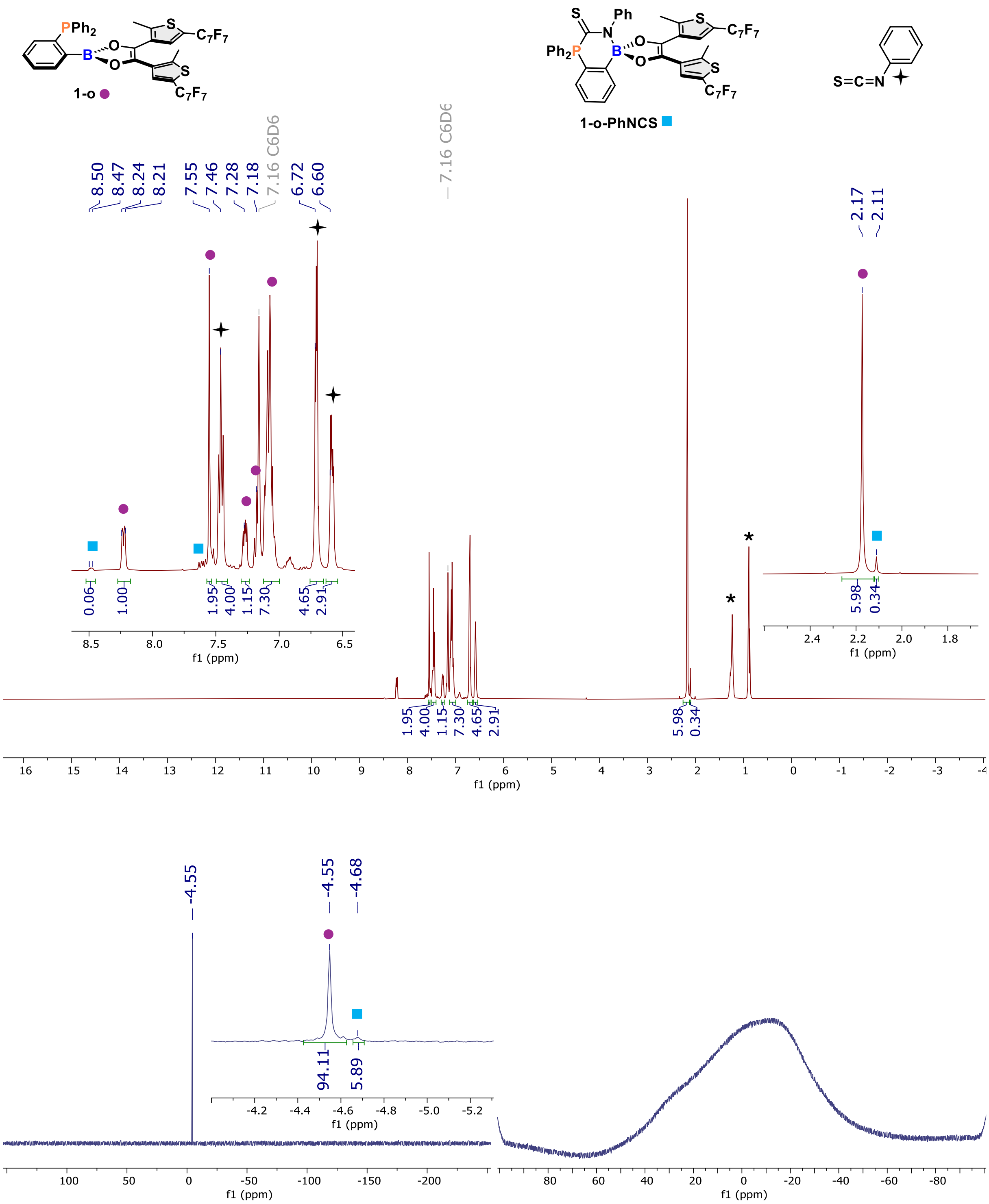

Figure S60. NMR spectra of a mixture of **1-o** and PhNCS in C_6D_6.Top: ^{1}H NMR (600 MHz), integrals set for **1-o**; Bottom left: ^{31}P NMR (243 MHz), total integral set to 100; Bottom Right: ^{11}B NMR (128 MHz). **n*-hexane

2h 300 nm

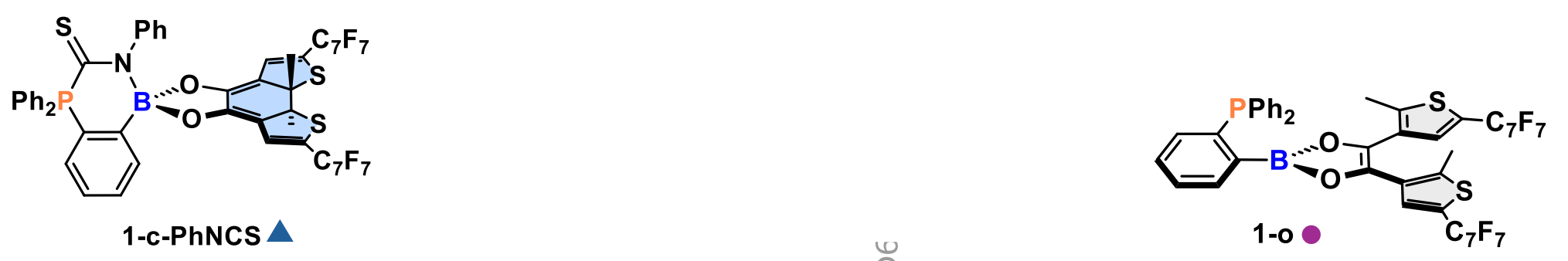

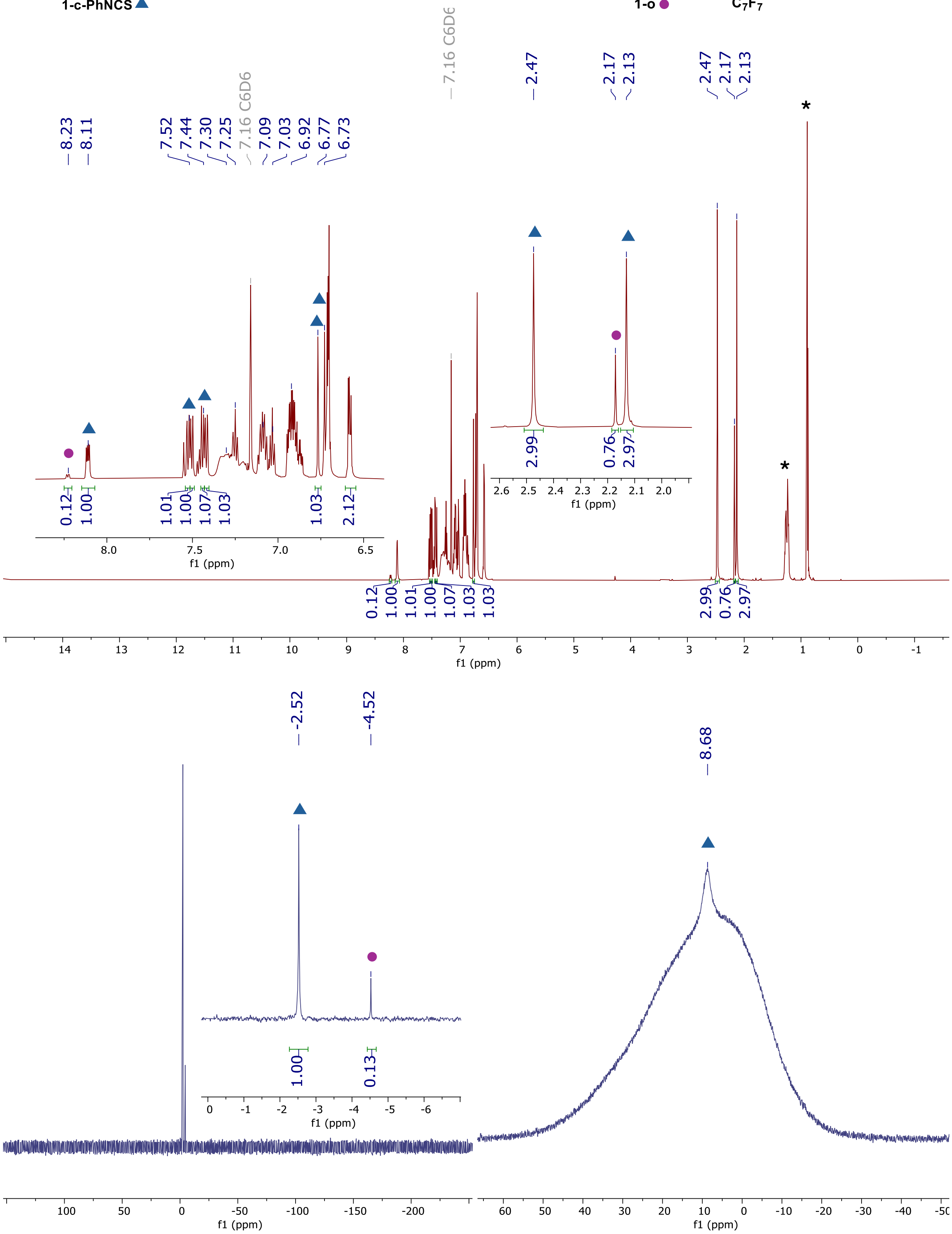

Figure S61. NMR spectra (C_6D_6) after irradiation at 300 nm, giving **1-c-PhNCS** and residual **1-o.** Top: ^{1}H NMR (600 MHz), integrals set for **1-c-PhNCS**; Bottom left: ^{31}P NMR (162 MHz); Bottom Right: ^{11}B NMR (193 MHz). **n*-hexane

30 minutes VIS

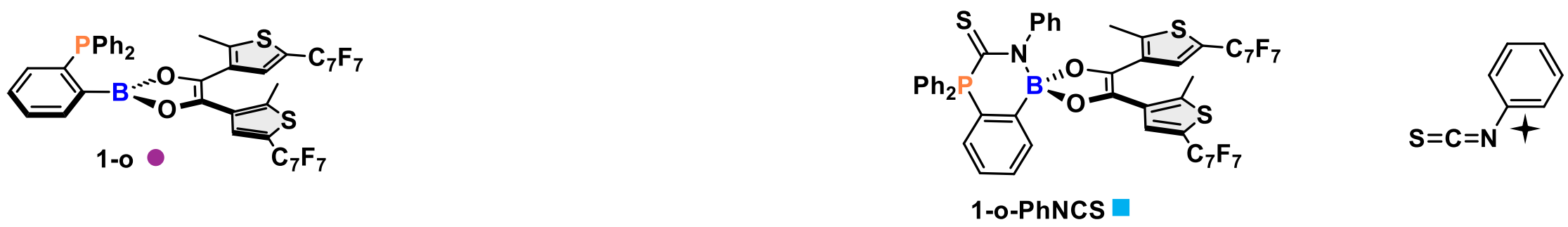

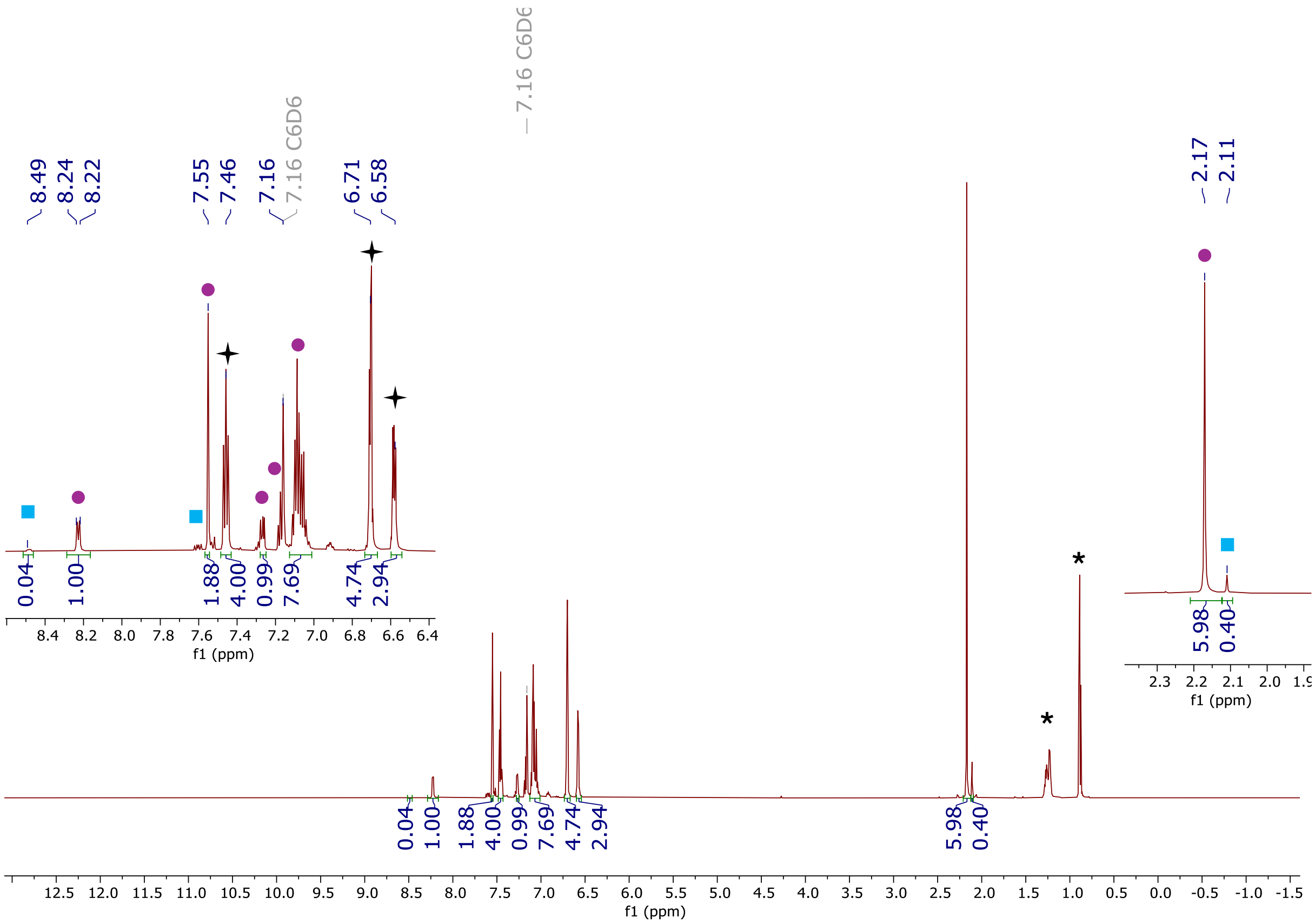

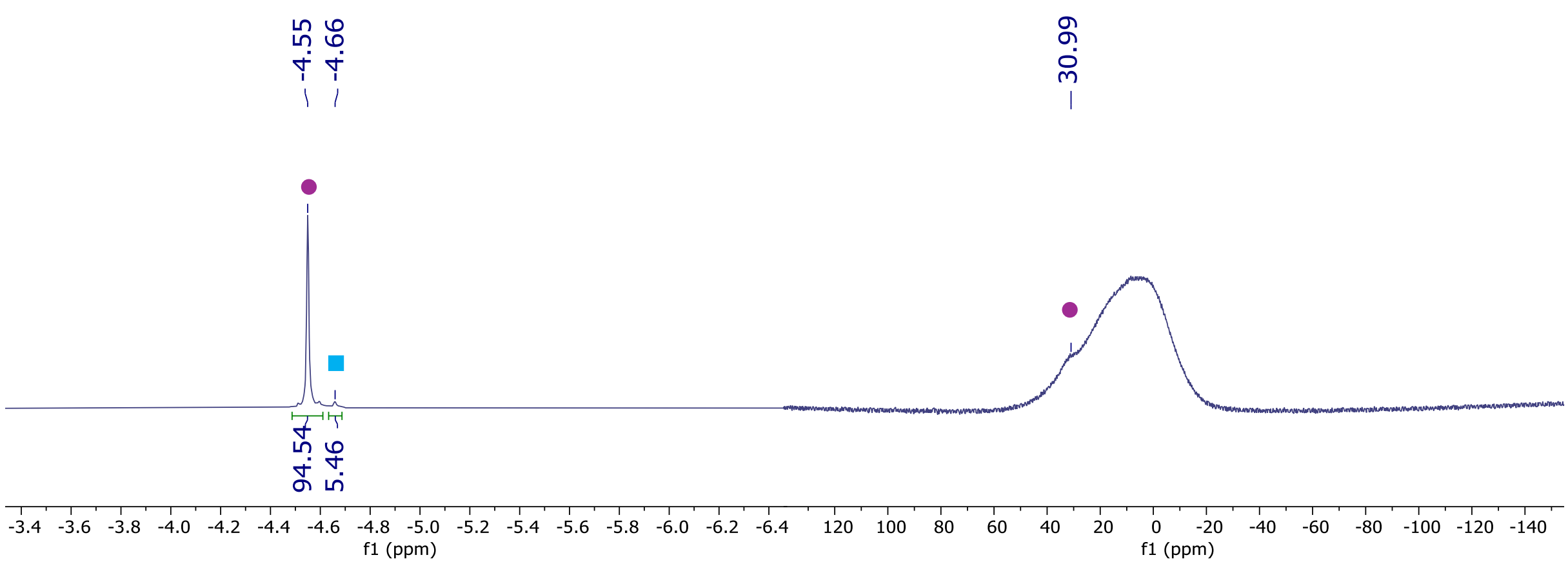

Figure S62. NMR spectra (C_6D_6) after VIS irradiation for 30 minutes, restoring the pre-irradiation equilibrium between **1-o** and **1-o-NCS.** Top: ^{1}H NMR (600 MHz), integrals set for **1-o**; Bottom left: ^{31}P NMR (243 MHz), total integral set to 100; Bottom Right: ^{11}B NMR (193 MHz). **n*-hexane

4.4.3 Propylene Oxide

1-o (7.9 mg) and propylene oxide (1.2 eq) were dissolved in C_6D_6 (0.5 ml). NMR spectra were taken after 24 hours at room temperature, after conversion to the closed form and again after ring-opening. Cyclization was induced at 300 nm, cycloreversion with broadband visible light. Due to the formation of a mixture of **1-o**, **1-o-PO**, and **1-c-PO** as a mixture of 2 diastereomers, complex signal patterns emerge in the ^{1}H NMR spectrum after photocyclization. Characteristic signals are marked below.

1-o + propylene oxide, 24 hours at room temperature

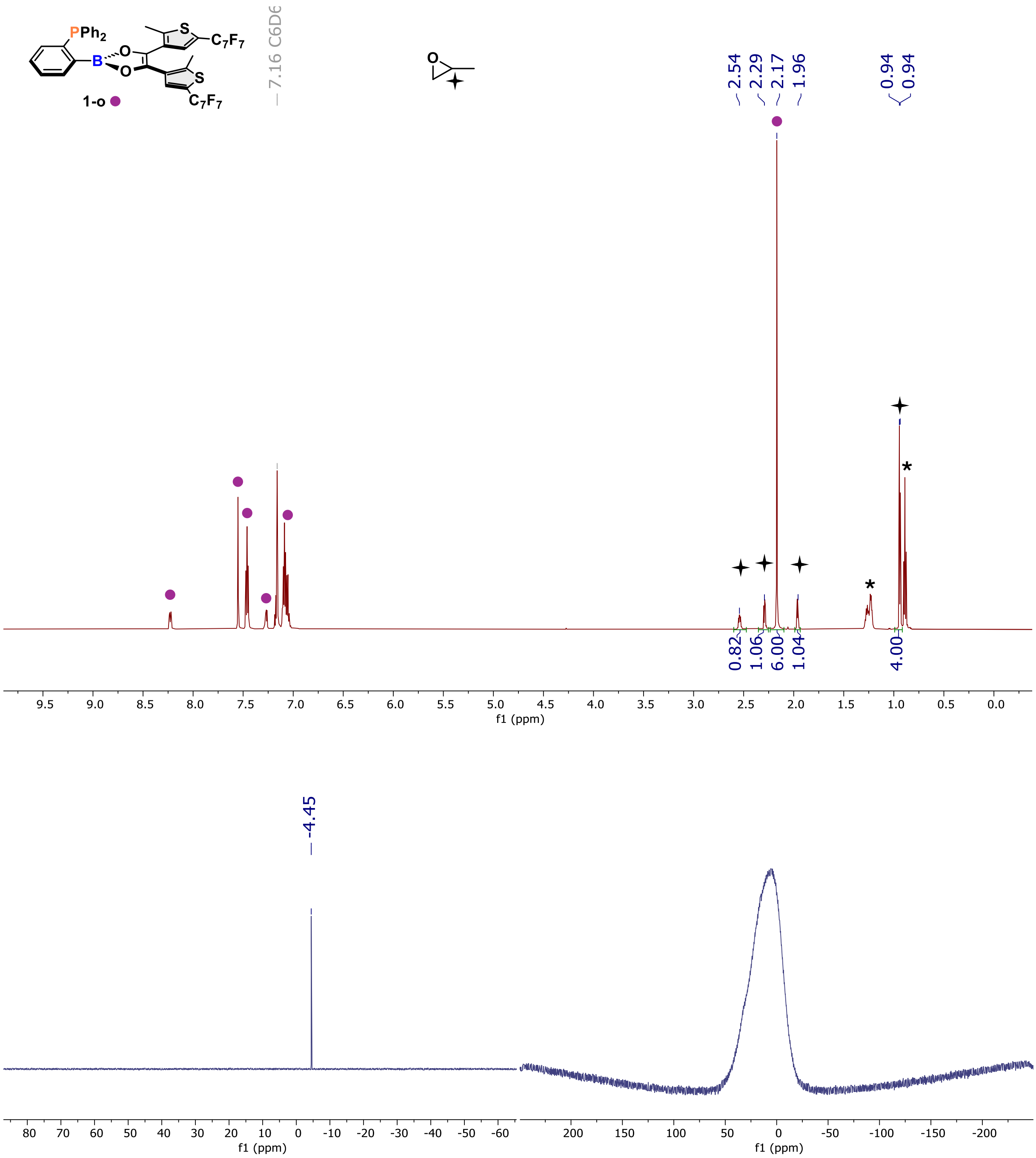

Figure S63. NMR spectra of a mixture of **1-o** and propylene oxide in C_6D_6, after 24 hours at room temperature**.** **Top:** ^{1}H NMR (600 MHz); Bottom left: ^{31}P NMR (243 MHz); Bottom Right: ^{11}B NMR (192 MHz). **n*-hexane

3h 300 nm

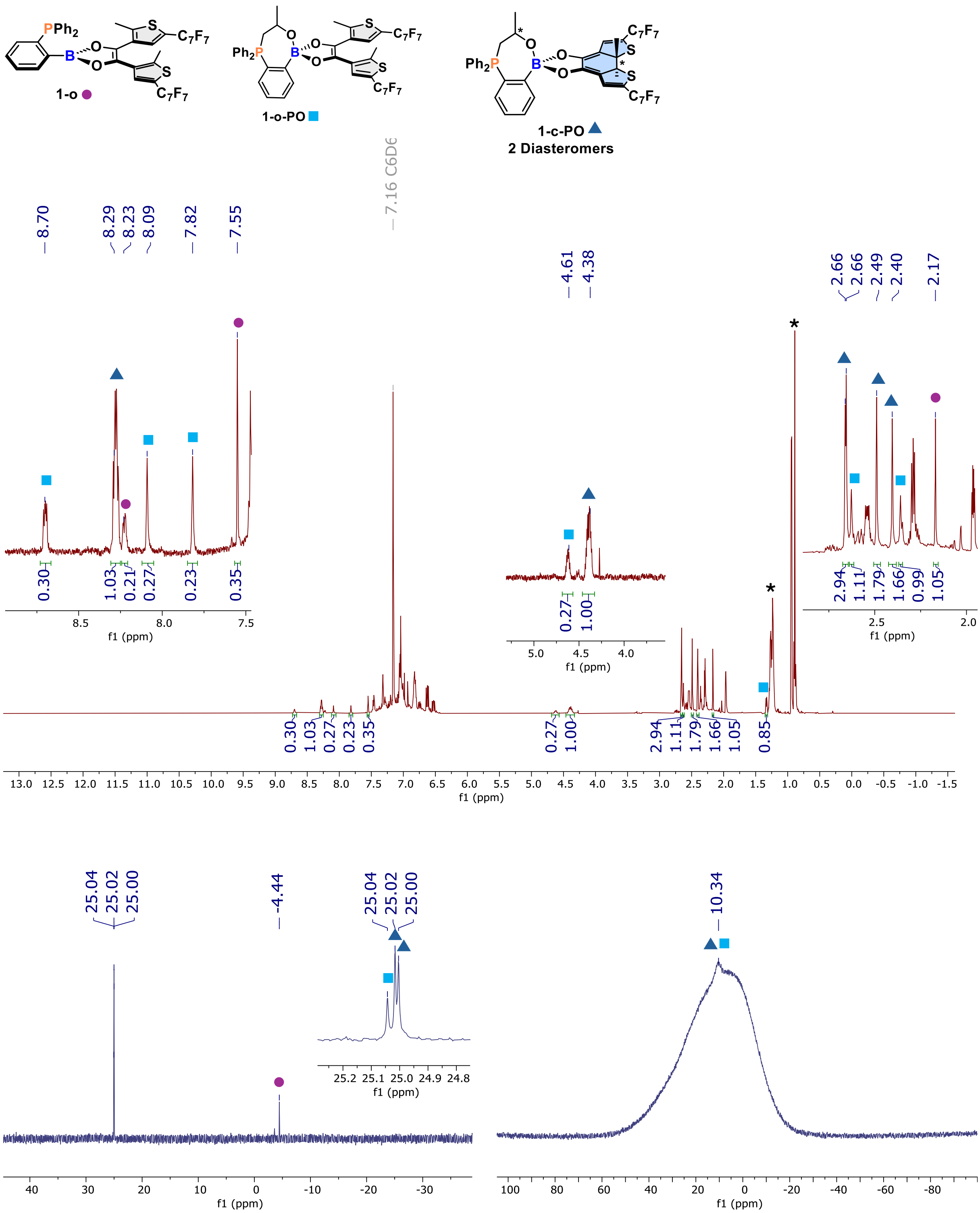

Figure S64. NMR spectra after irradiation at 300 nm, giving a mixture of **1-o, 1-o-PO** and **1-c-PO** as two diastereomers (C_6D_6)**.** Top: ^{1}H NMR (600 MHz). Due to the complexity of the emerging signals, full assignment was not possible. Integrals set for the combined diasteromers of **1-c-PO**; Bottom left: ^{31}P NMR (243 MHz); Bottom Right: ^{11}B NMR (192 MHz). *_n_-hexane

30 minutes VIS

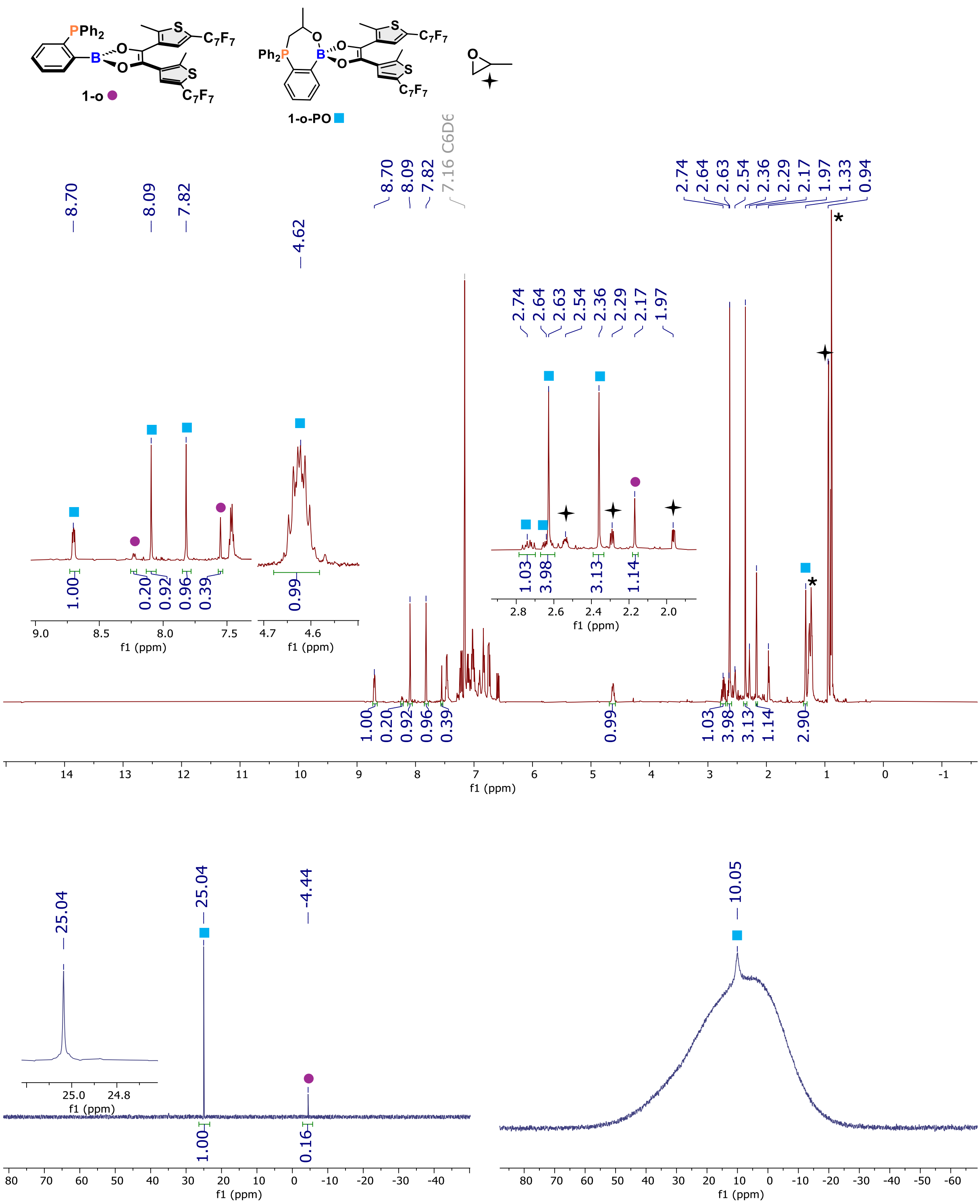

Figure S65. NMR spectra (C_6D_6) after VIS irradiation for 30 minutes, resulting in a mixture of **1-o-PO** and **1-o.** Top: ^{1}H NMR (600 MHz), integrals set for **1-o-PO**; Bottom left: ^{31}P NMR (243 MHz); Bottom Right: ^{11}B NMR (193 MHz). *n-hexane

4.5 Impact of Multiple Switching Cycles

To evaluate the impact of multiple switching cycles on substrate activation capabilities, 1-o (6.2 mg) and PhNCS (4 eq) were dissolved in C_6D_6 and cyclooctane was added as an internal standard. The mixture was subjected to alternating UV (365 nm LEDs, 30 minutes, >75% conversion to the closed form) and VIS (until complete decoloration of the solution) irradiation. This cycle was repeated 5 times. Integration against cyclooctane shows a reduction in signal intensity by 4% after 5 cycles. Due to slightly inconsistent conversions to the closed form after UV irradiation, decomposition was evaluated by integrating open form signals.

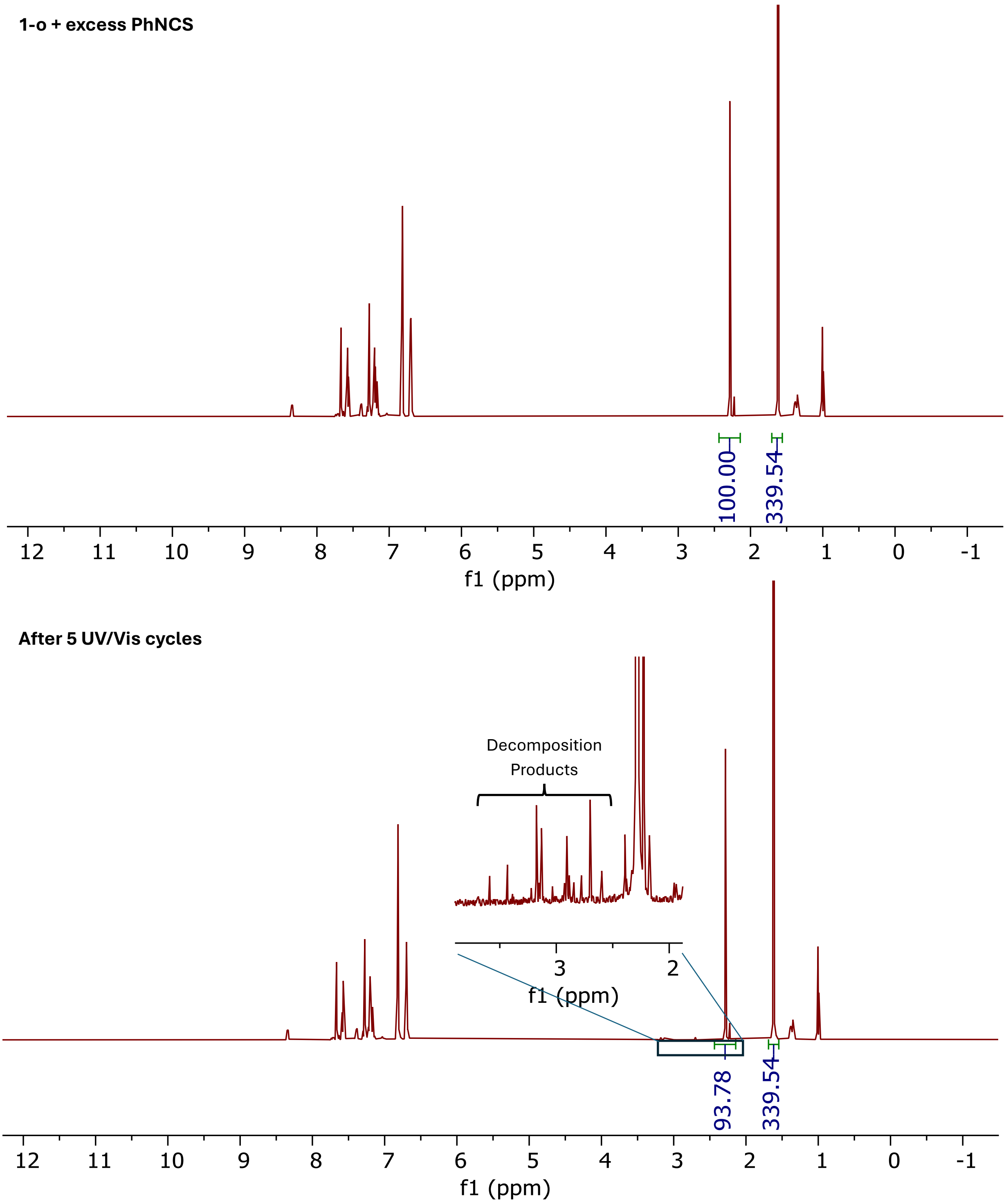

Figure S66. ^{1}H NMR spectra (C_6D_6, 600 MHz) of 1-o and PhNCS, before irradiation (top) and after 5 UV/Vis cycles (bottom). Thiophene CH_3 signals of **1-o** and **1-o-PhNCS** were integrated against cyclooctane.

Complete NMR spectra

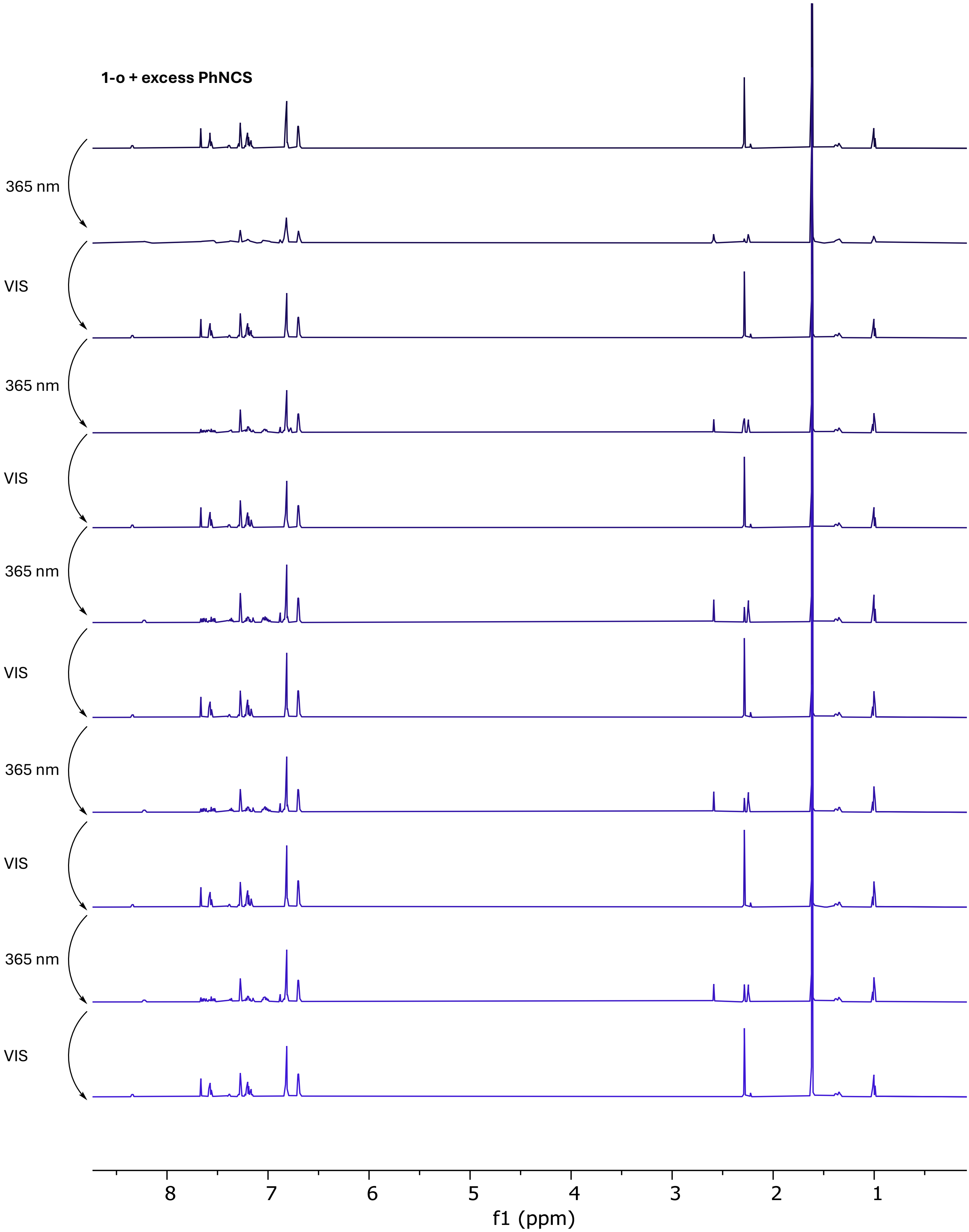

Figure S67. ^{1}H NMR spectra (C_6D_6, 600 MHz) of **1-o** and PhNCS, before irradiation (top) and alternating UV and Vis irradiation.

4.6 Substrate Activation (2)

4.6.1 CO_2

2-o (14.8 mg) was dissolved in CD_2Cl_2. The solution was pressurized with CO_2 (4 atm) and subjected to NMR analysis before irradiation, after conversion to the closed form, and again after cycloreversion. Cyclization was induced with 300 nm light, cycloreversion with broadband VIS irradiation.

1-o + 4 atm CO_2

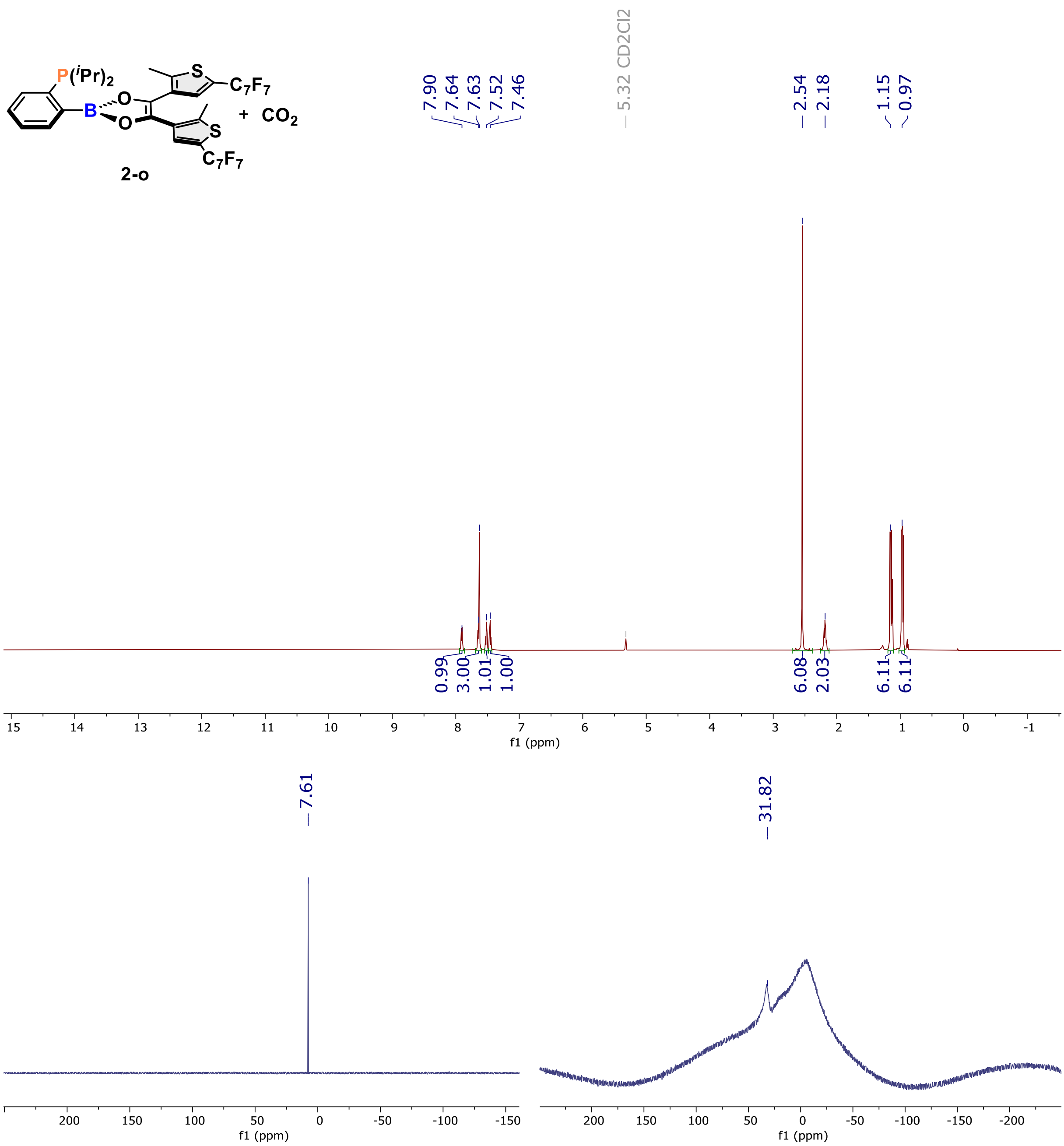

Figure S68. NMR spectra of **2-o** and 4 atm CO_2 in CD_2Cl_2. Top: ^1H NMR (600 MHz); Bottom left: ^{31}P NMR (243 MHz); Bottom Right: ^{11}B NMR (192 MHz). All signals can be assigned to **2-o**.

4h 300 nm

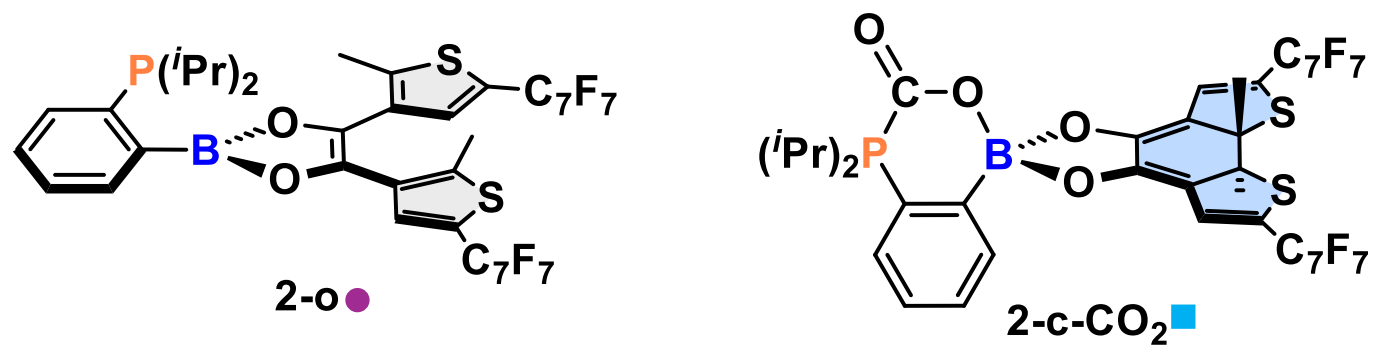

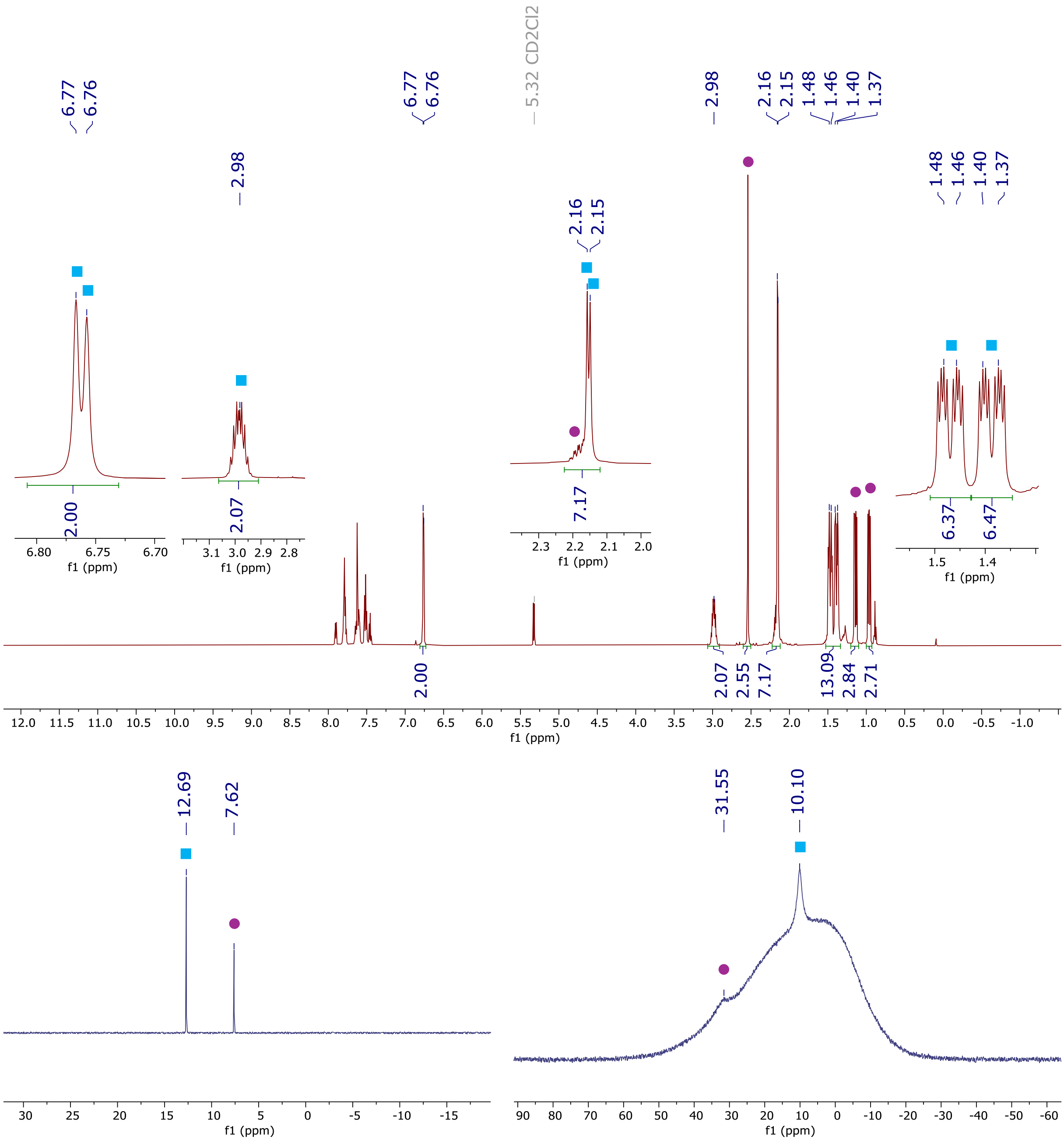

Figure S69. NMR spectra (CD_2Cl_2) after irradiation at 300 nm and 65% conversion to **2-c**. Top: ^{1}H NMR (600 MHz); Bottom left: ^{31}P NMR (243 MHz); Bottom Right: ^{11}B NMR (192 MHz). Only **2-o** and **2-c-CO₂** are detected.

1h VIS

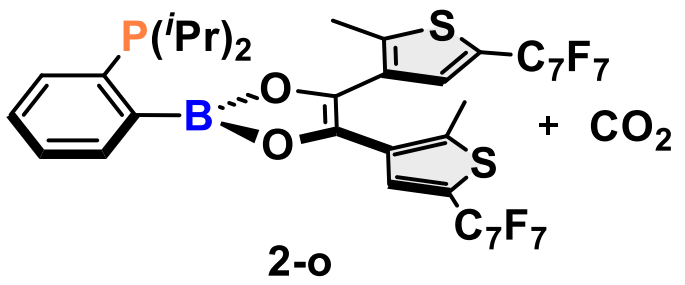

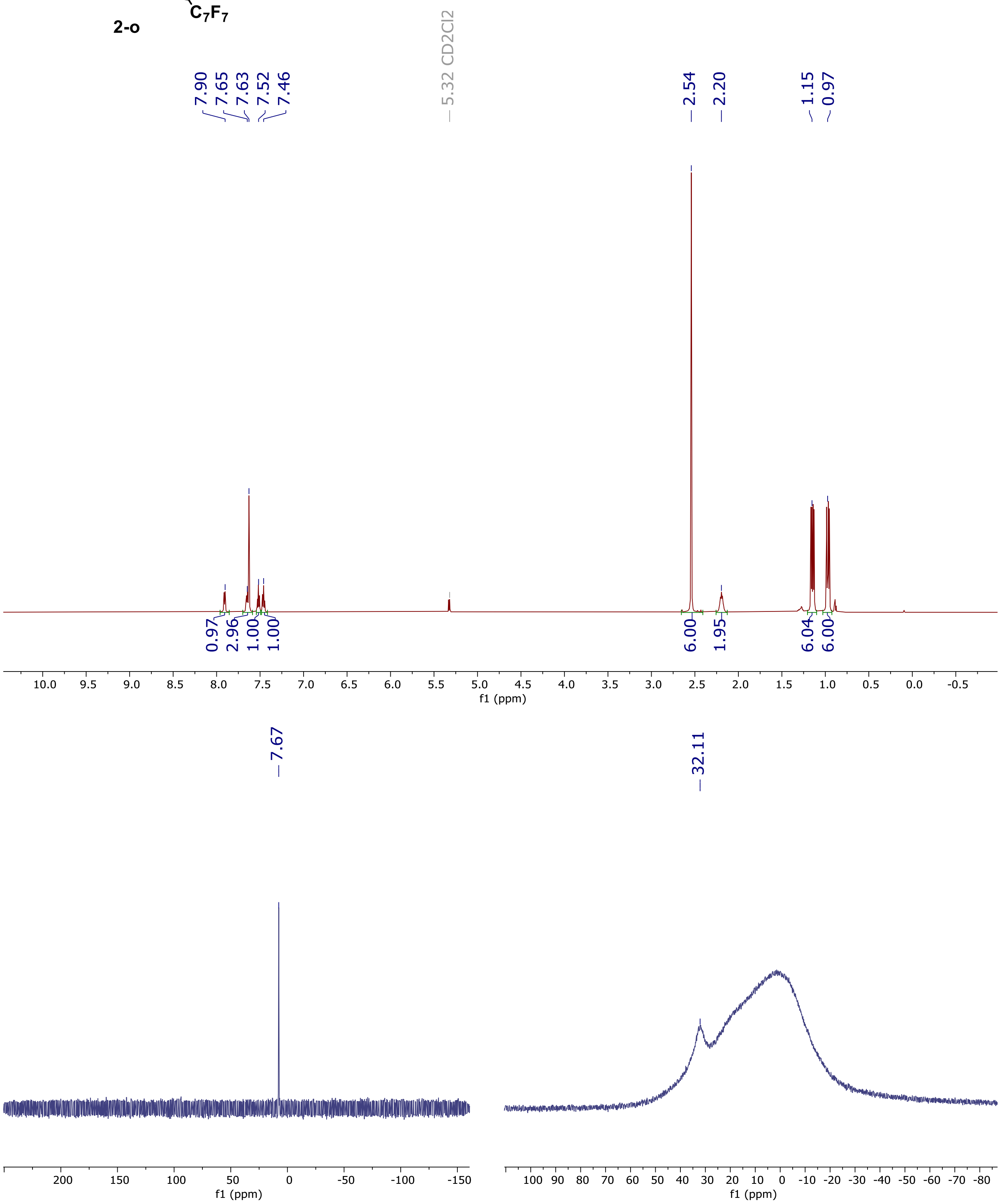

Figure S70. NMR spectra (CD_2Cl_2) after VIS irradiation, restoring **2-o.** Top: ^{1}H NMR (600 MHz); Bottom left: ^{31}P NMR (243 MHz); Bottom Right: ^{11}B NMR (192 MHz). All signals can be assigned to **2-o**.

$^{13}CO_2$

2-o (13.9 mg) was dissolved in CD_2Cl_2 (0.5 ml) and degassed by two freeze-pump-thaw cycles. The mixture was subsequently pressurized by $^{13}CO_2$ (2 atm). Due to the higher water content of the employed $^{13}CO_2$ leading to water dynamically bound to the FLP, line broadening can be observed in the ^{31}P NMR spectrum. NMR spectra were measured before irradiation, after conversion to the closed form and again after cycloreversion.

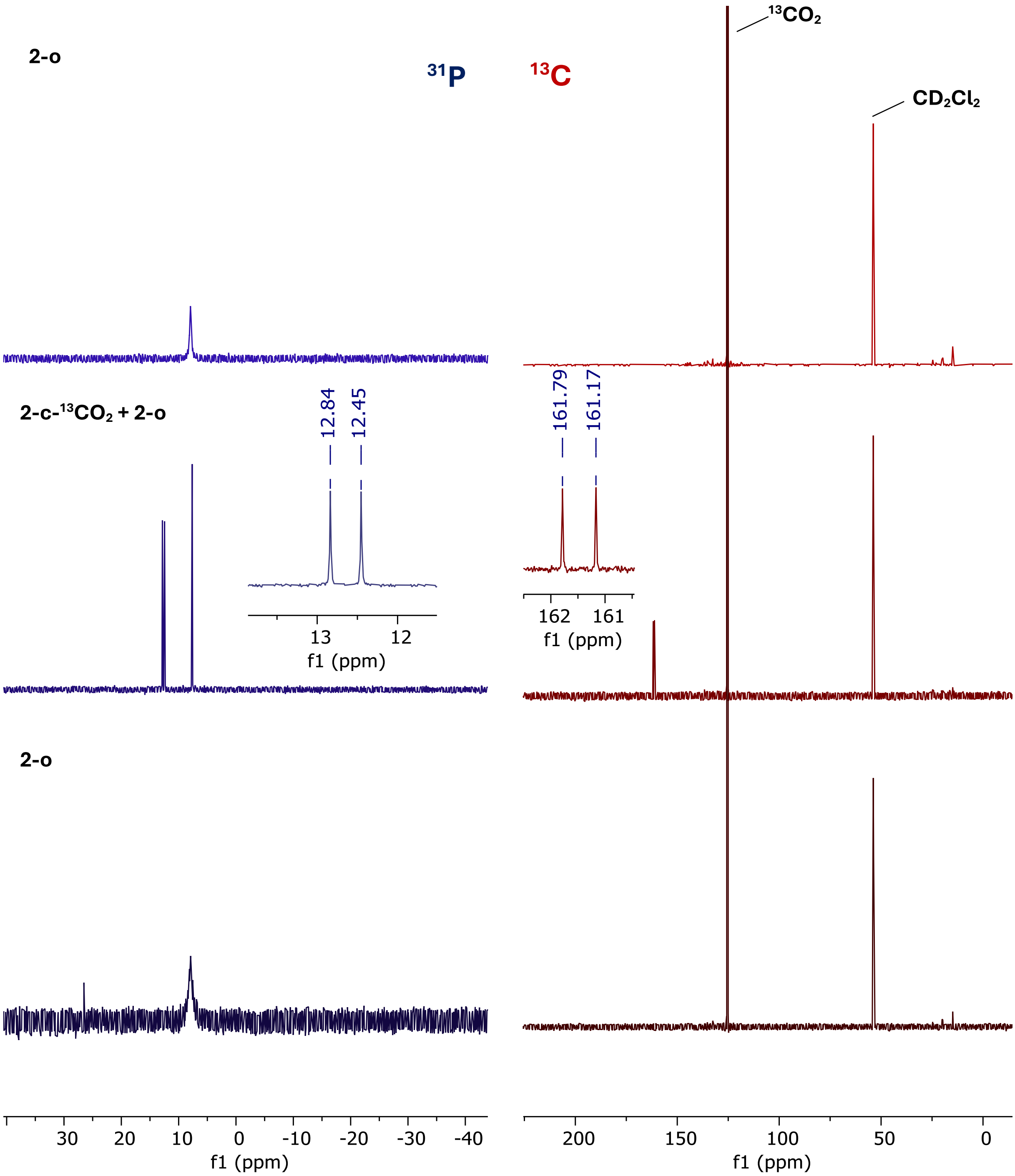

Figure S71. ^{31}P (243 MHz, left) and ^{13}C (151 MHz, right) NMR spectra (CD_2Cl_2) of a mixture of **2** and 2 atm $^{13}CO_2$. Top: before irradiation. Middle: after irradiation at 300 nm. Bottom: after irradiation with broadband visible light.

4.6.2 Benzophenone

2-o (9.9 mg) and benzophenone (2 eq) were dissolved in C_6D_6 (0.5 ml). The mixture was analyzed by NMR spectroscopy before irradiation, after conversion to the closed form and again after ring-opening. Cyclization was induced at 300 nm, cycloreversion with broadband visible light.

Before Irradiation

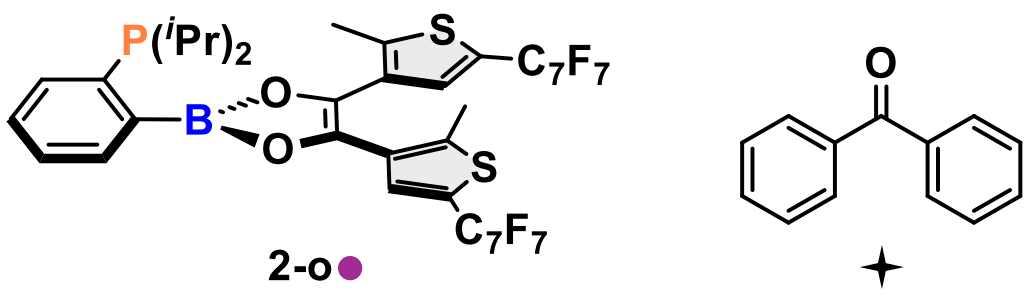

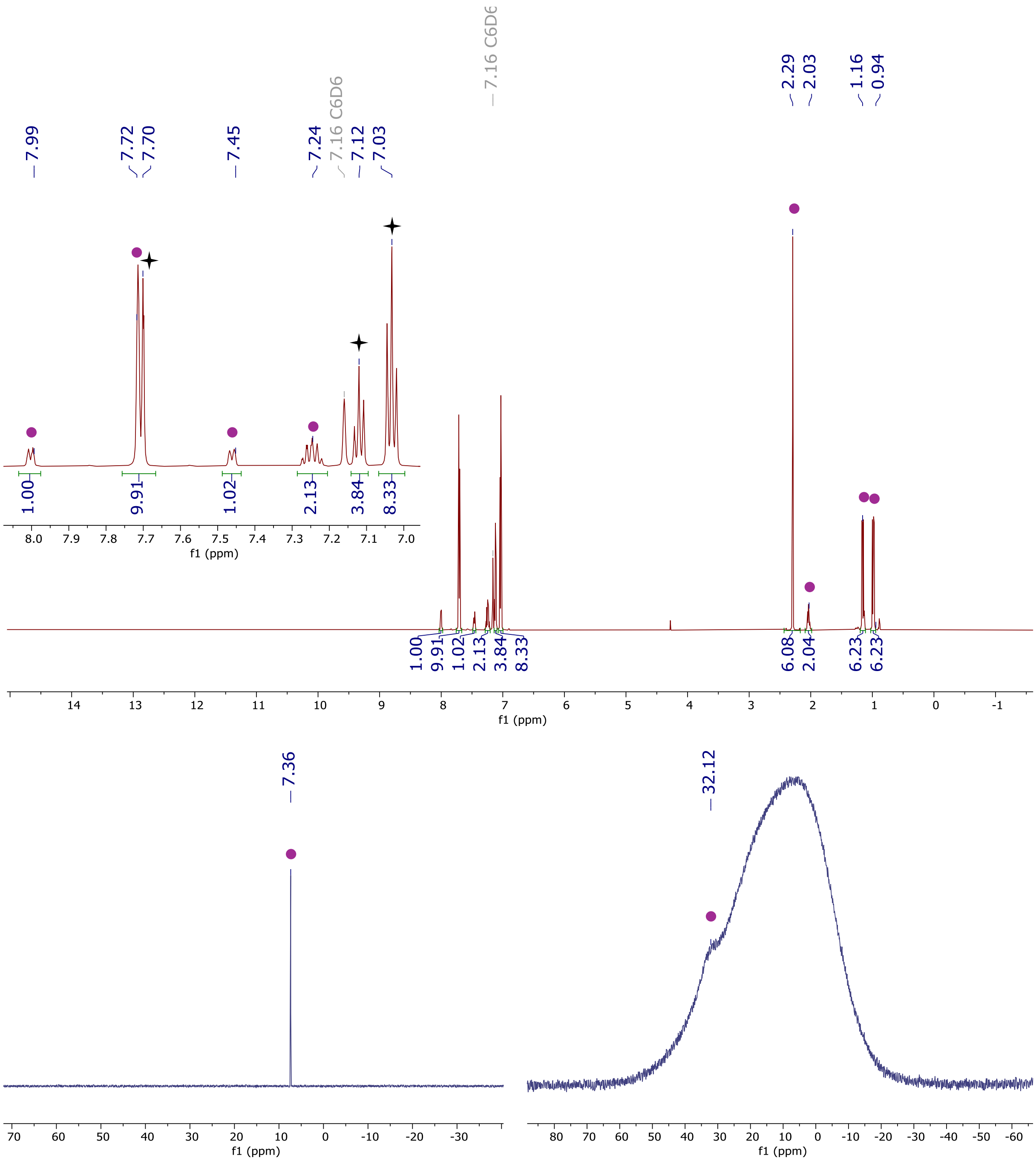

Figure S72. NMR spectra of a mixture of **2-o** and benzophenone in C_6D_6. Top: ^{1}H NMR (600 MHz); Bottom left: ^{31}P NMR (243 MHz); Bottom Right: ^{11}B NMR (193 MHz).

2h 300 nm

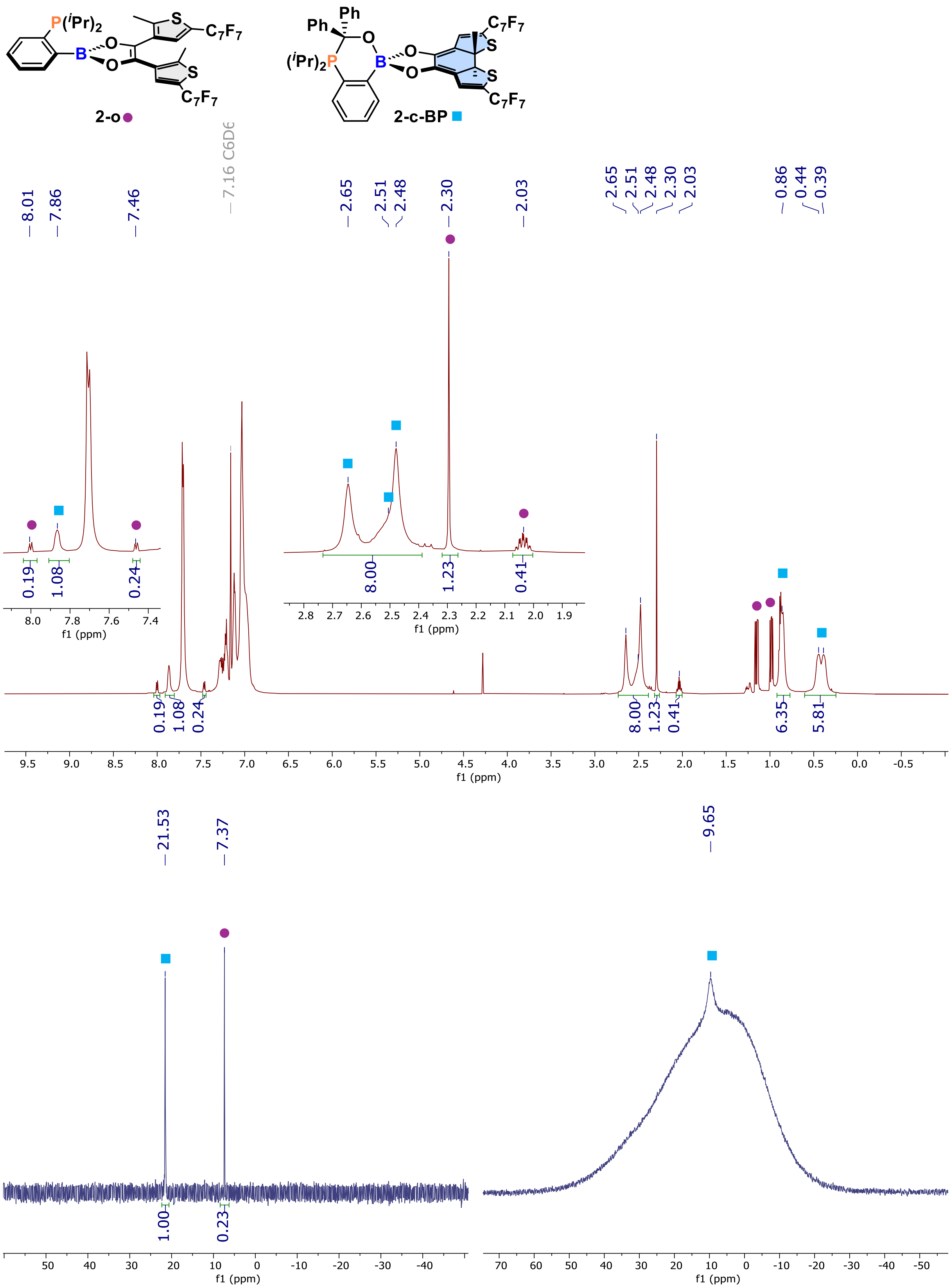

Figure S73. NMR spectra (C_6D_6) after irradiation at 300 nm, giving a mixture of **2-o** and **2-c-BP.** Top: ^{1}H NMR (600 MHz); Bottom left: ^{31}P NMR (243 MHz); Bottom Right: ^{11}B NMR (193 MHz).

30 minutes VIS

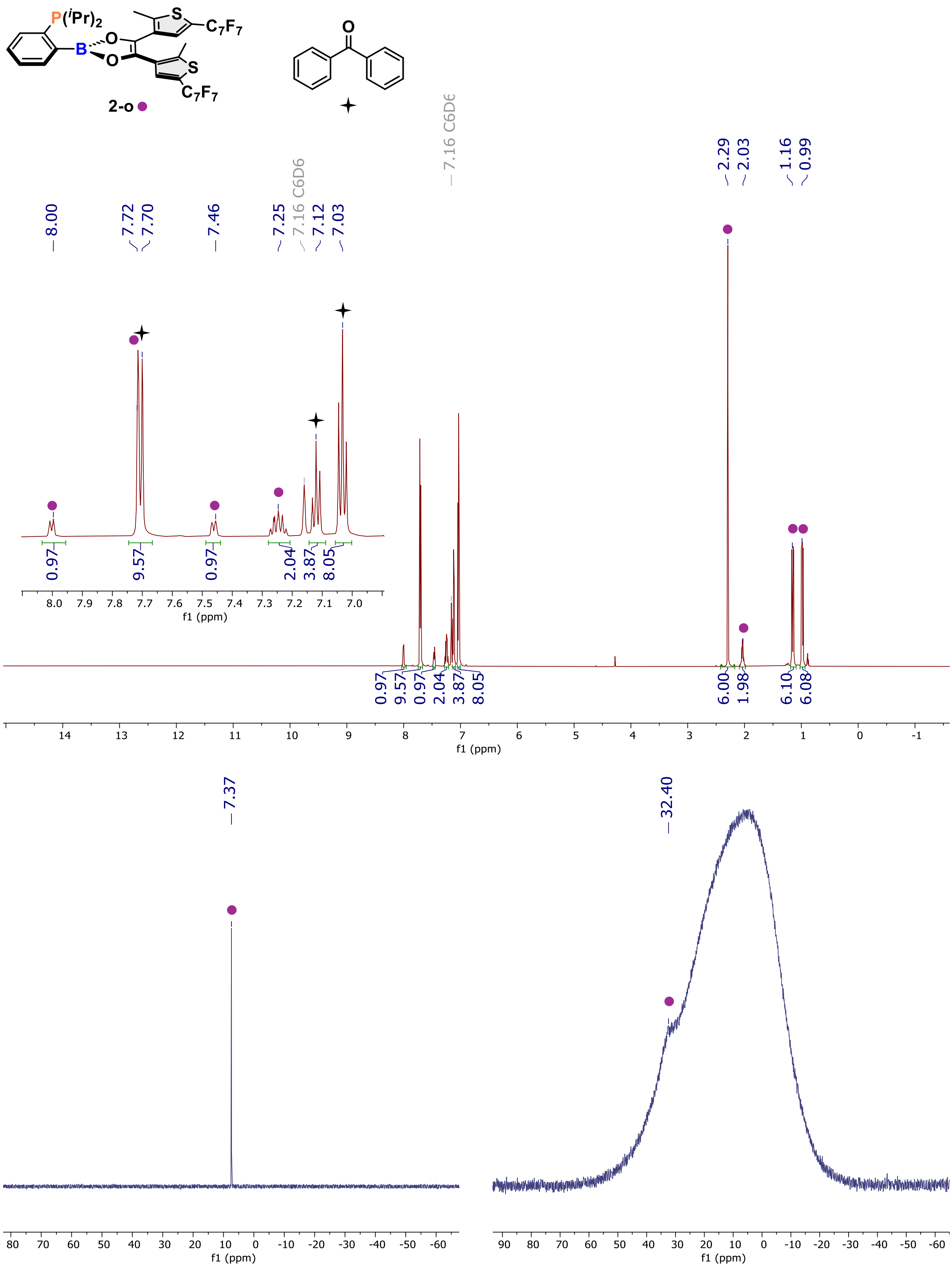

Figure S74. NMR spectra (C_6D_6) after VIS irradiation, restoring **2-o** under release of benzophenone**.** Top: ^{1}H NMR (600 MHz); Bottom left: ^{31}P NMR (243 MHz); Bottom Right: ^{11}B NMR (193 MHz).

4.6.3 tert-Butyl Isonitrile

2-o (12.1 mg) and tert-Butyl isonitrile (1 eq) were dissolved in C_6D_6 (0.5 ml). The mixture was analyzed by NMR spectroscopy before irradiation, after conversion to the closed form and again after ring-opening. Cyclization was induced at 300 nm, cycloreversion with broadband visible light.

Before Irradiation

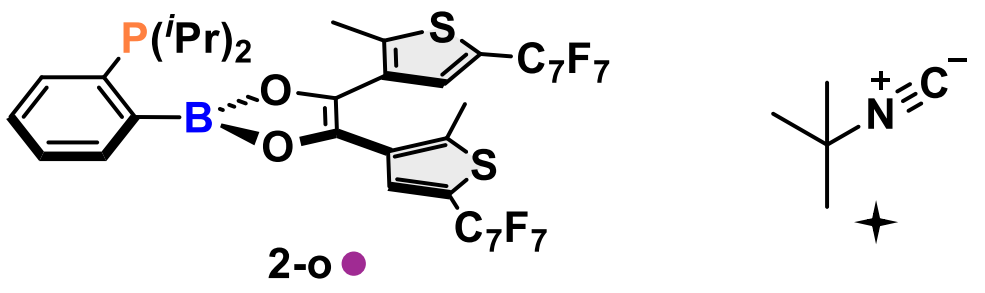

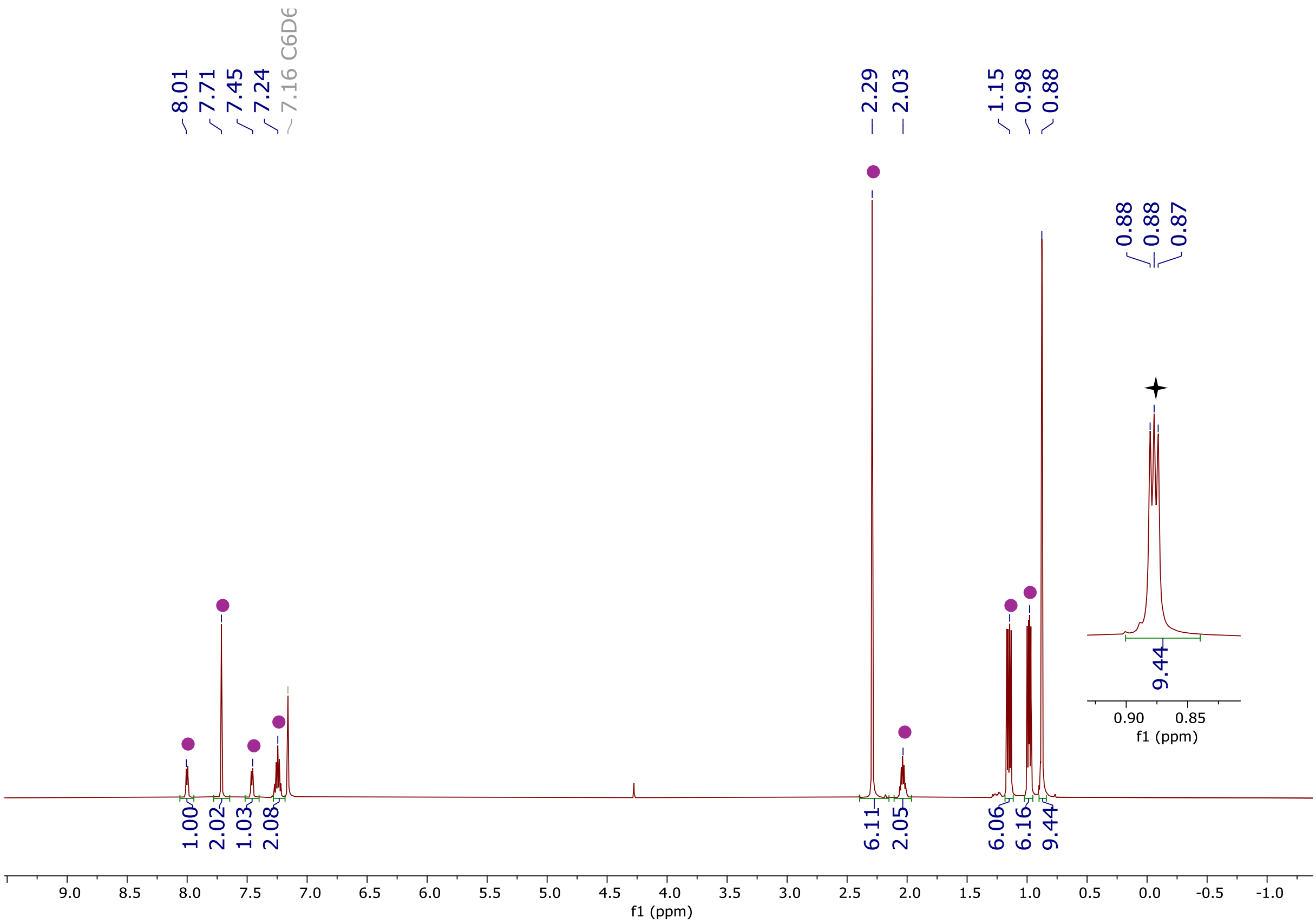

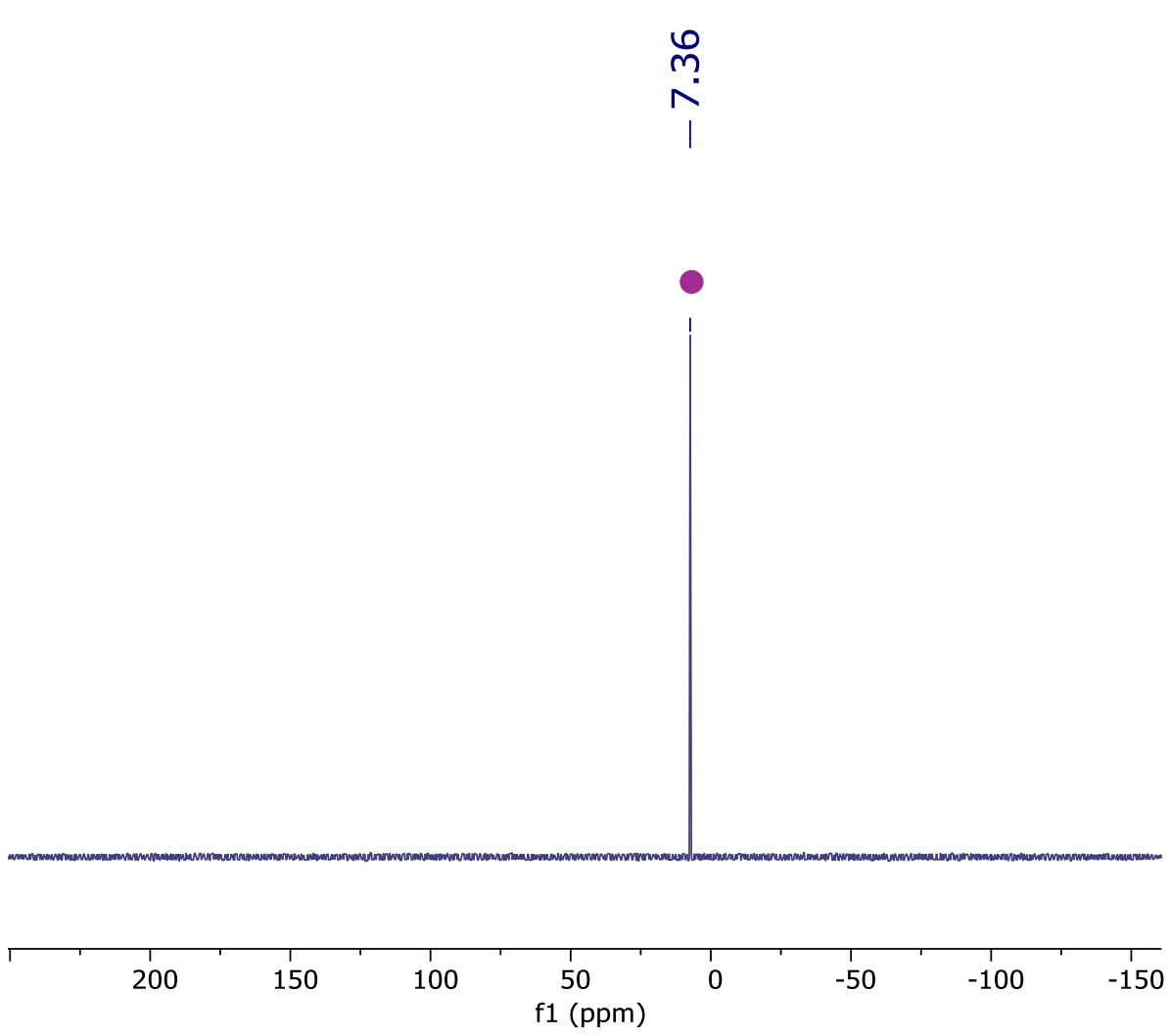

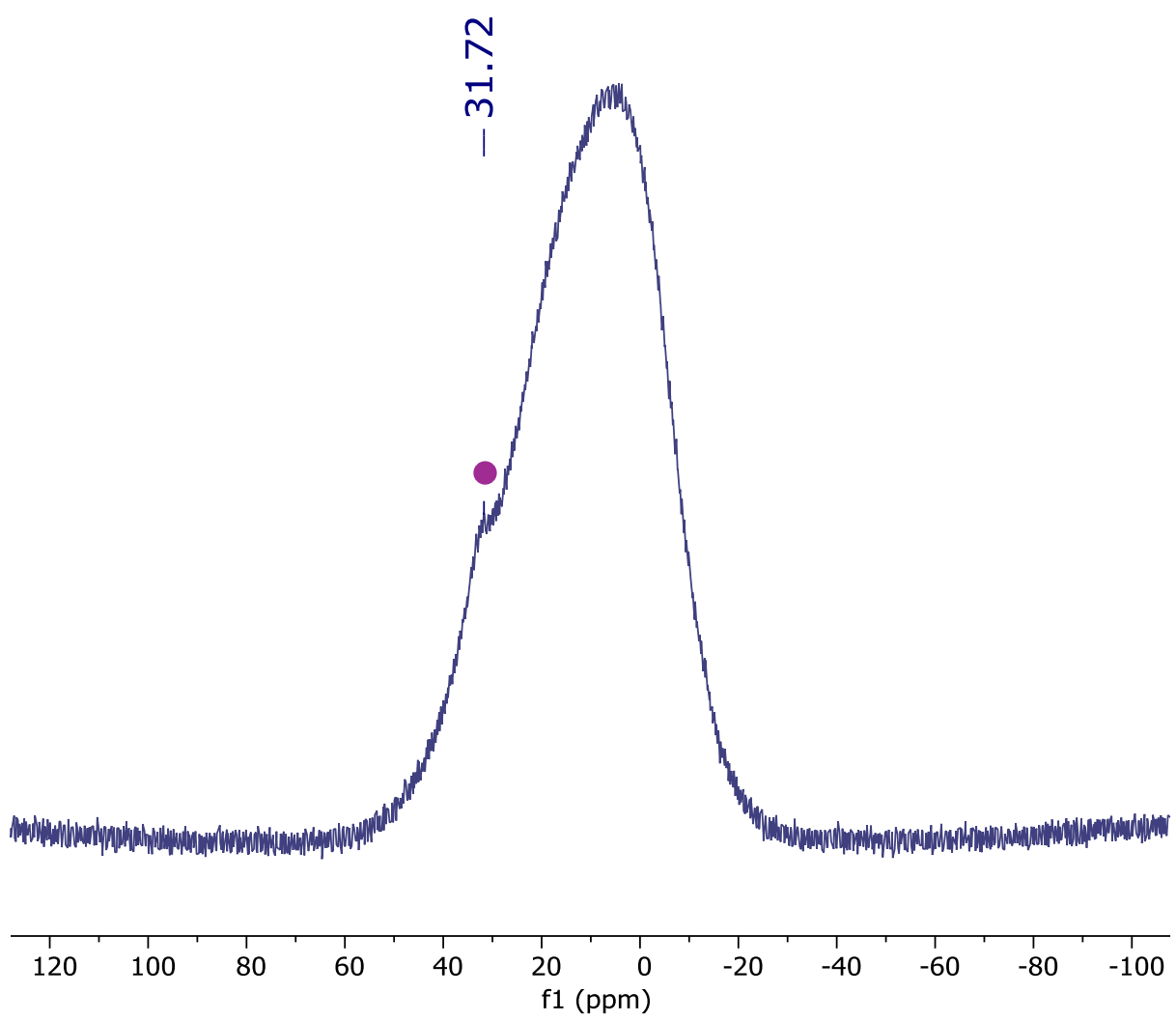

Figure S75. NMR spectra of a mixture of **2-o** and tert-butyl isonitrile in C_6D_6.Top: ^1H NMR (600 MHz); Bottom left: ^{31}P NMR (243 MHz); Bottom Right: ^{11}B NMR (193 MHz).

2h 300 nm

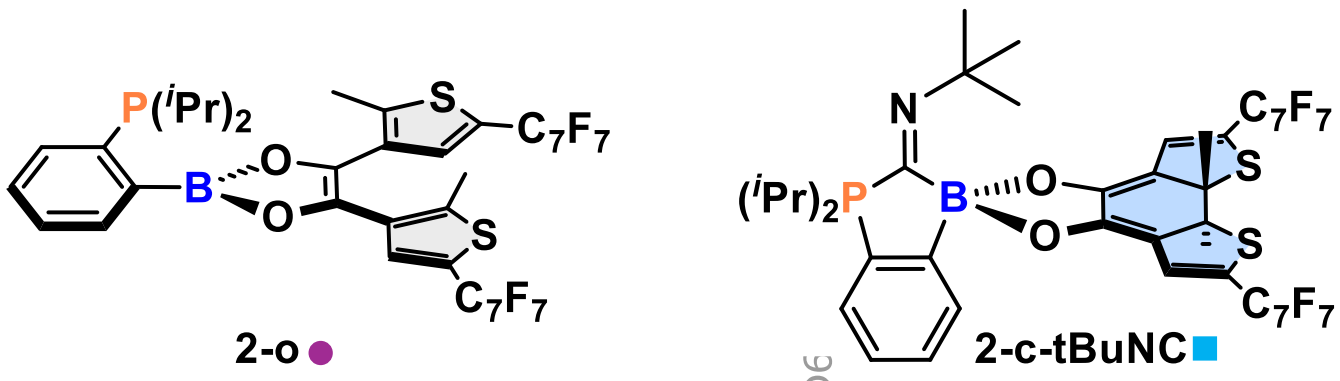

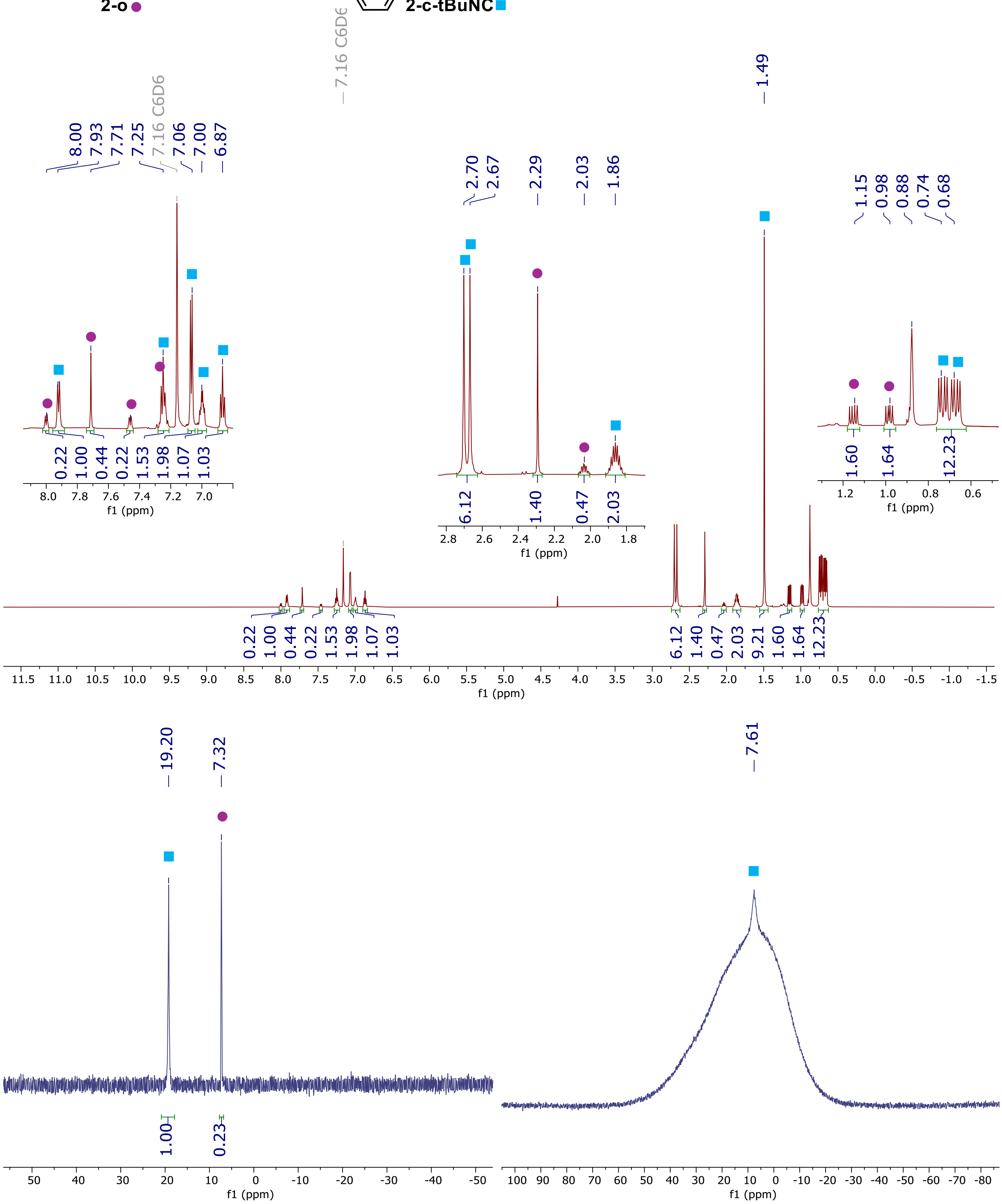

Figure S76. NMR spectra (C_6D_6) after irradiation at 300 nm, giving a mixture of **2-o** and **2-c-tBuNC.** Top: ^{1}H NMR (600 MHz); Bottom left: ^{31}P NMR (162 MHz); Bottom Right: ^{11}B NMR (193 MHz).

30 minutes VIS

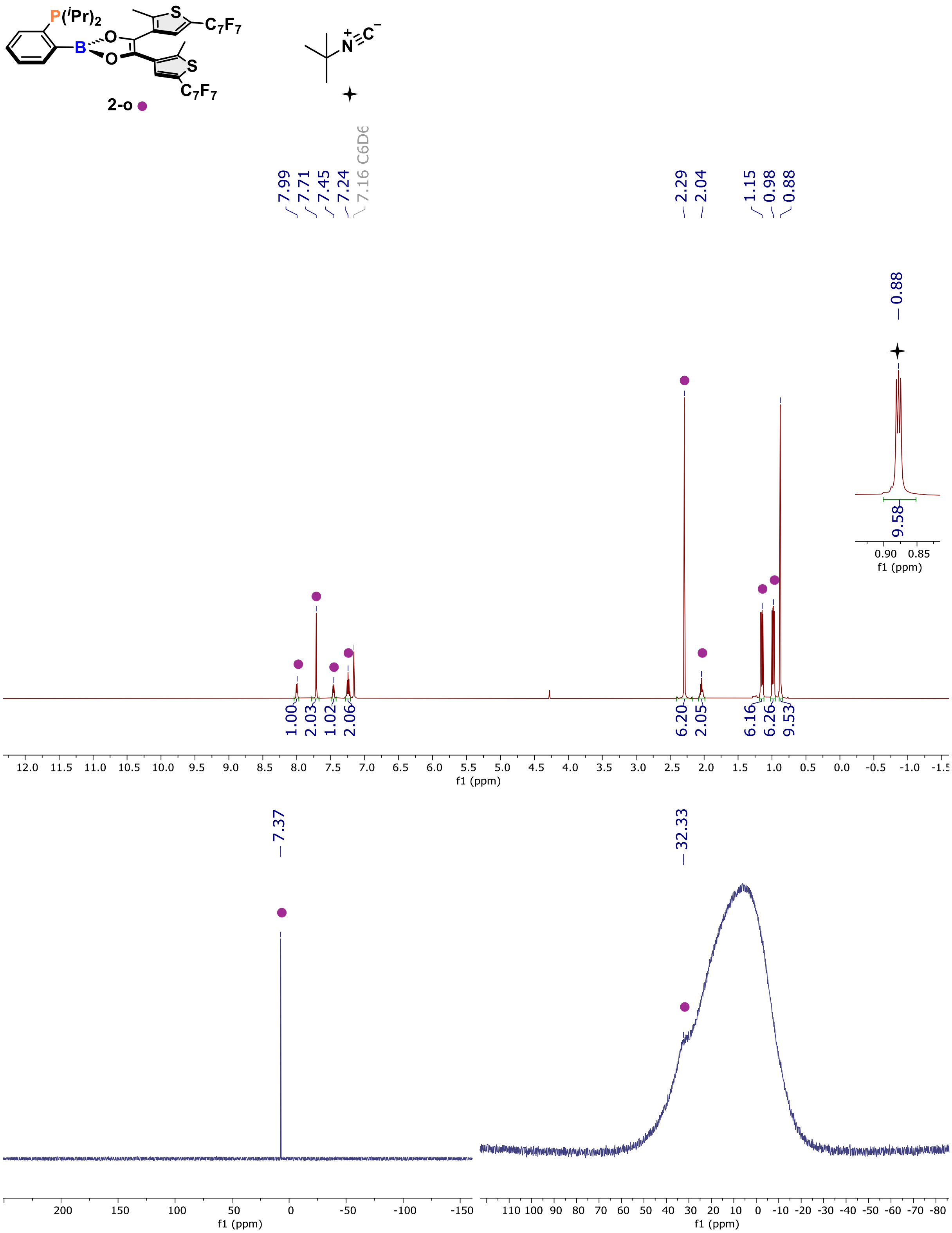

Figure S77. NMR spectra (C_6D_6) after VIS irradiation, restoring **2-o** with release of tBuNC. Top: ^{1}H NMR (600 MHz); Bottom left: ^{31}P NMR (243 MHz); Bottom Right: ^{11}B NMR (193 MHz).

4.6.4 p-Tolylaldehyde/Phenyl Isothiocyanate

2-o was dissolved in CD_2Cl_2 and treated with an excess of *p*-tolylaldehyde or phenyl isothiocyanate. Complete conversion to the substrate bound adduct was observed by ^{1}H and ^{31}P NMR spectroscopy in both cases.

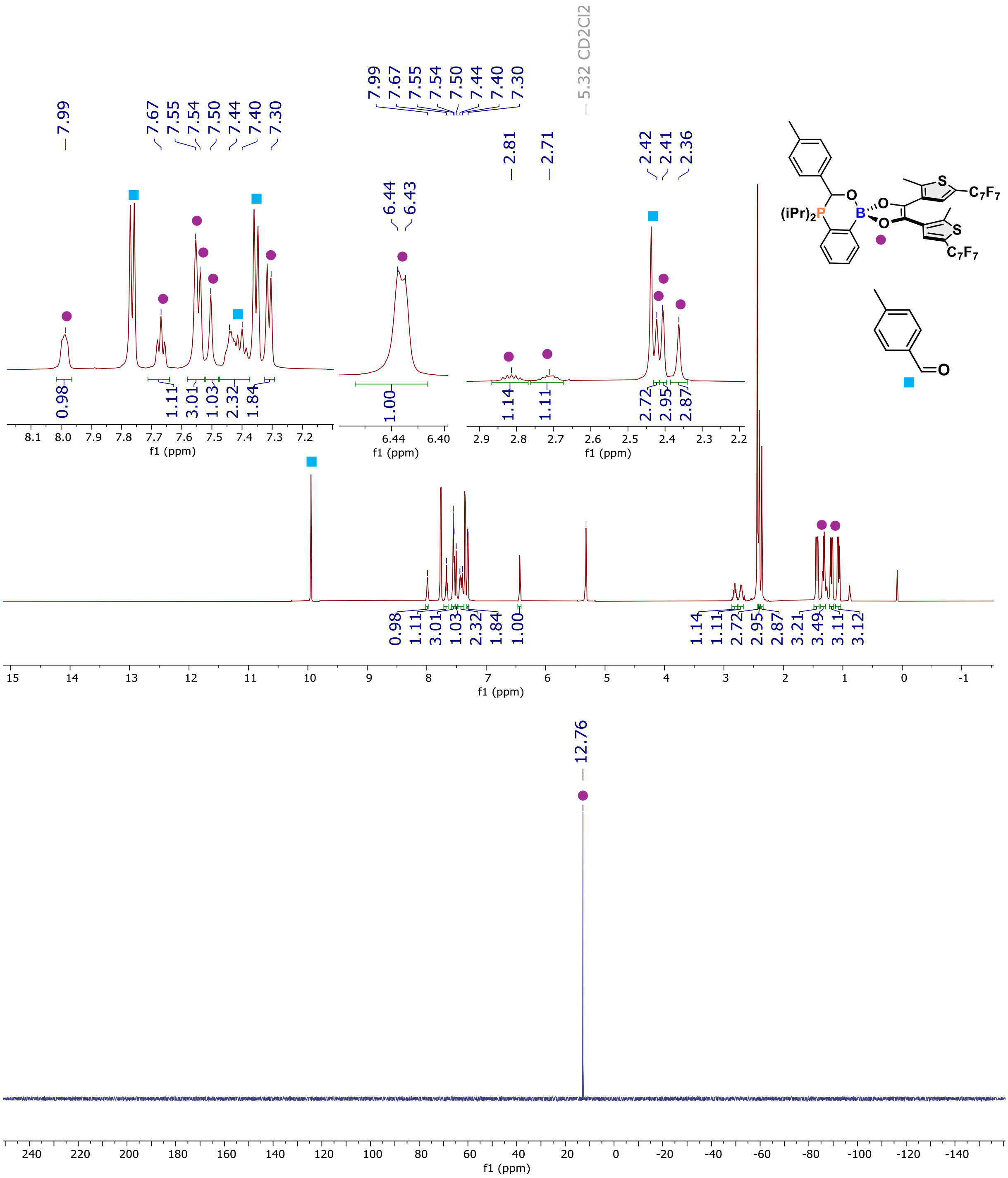

Figure S78. NMR spectra (CD_2Cl_2) of **2-o-TolCHO** and residual p-tolylaldehyde**.** Top: ^{1}H NMR (600 MHz); Bottom: ^{31}P NMR (243 MHz).

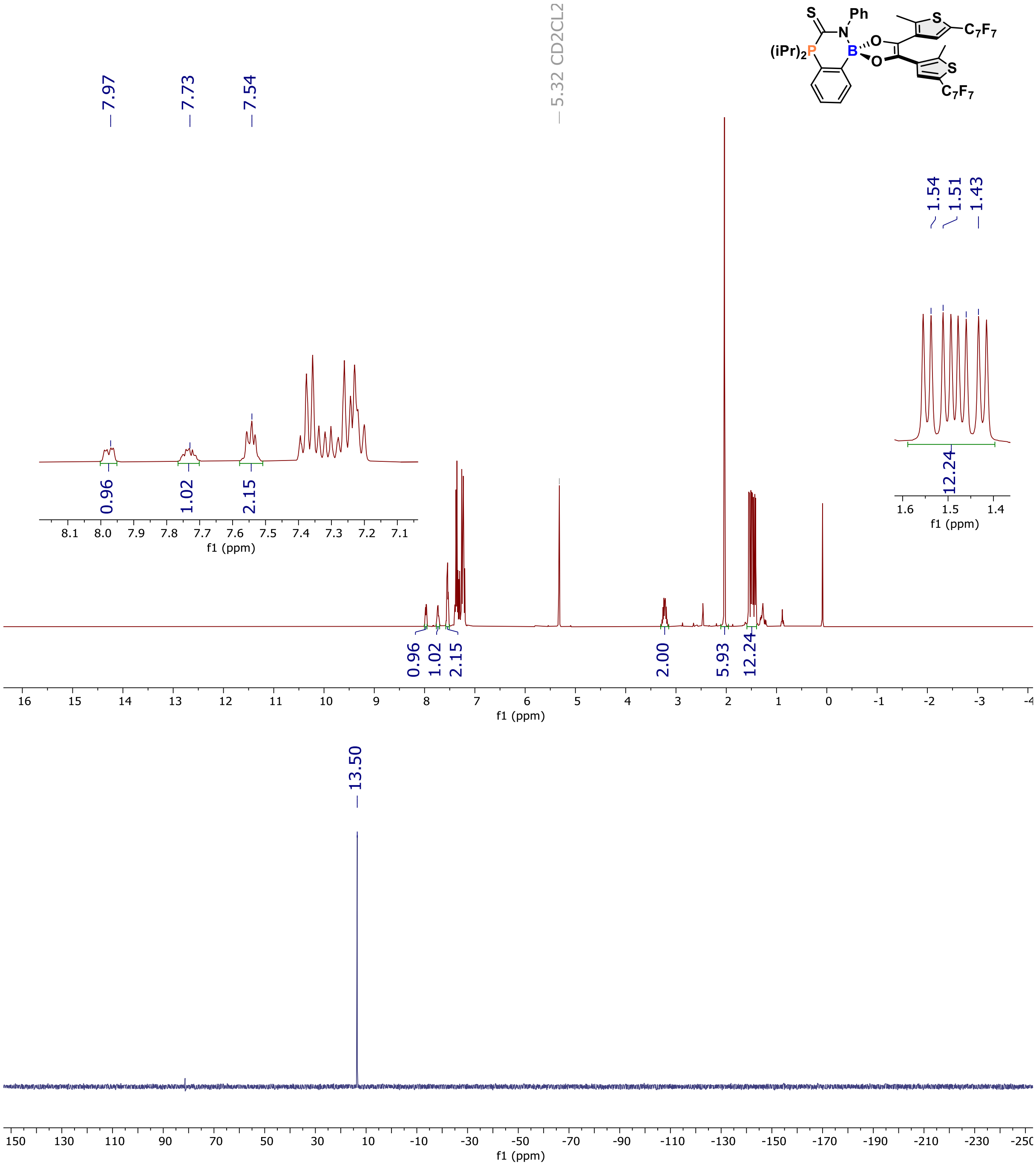

Figure S79. NMR spectra (CD_2Cl_2) of **2-o-PhNCS** and residual PhNCS. Top: ^1H NMR (400 MHz); Bottom: ^{31}P NMR (162 MHz).

4.6.5 Control Experiments with tBuNC and Benzophenone

To exclude the possibility of direct photochemical substrate activation, tBuNC and benzophenone were added to pre-irradiated solutions of **2-c**. For both substrates, identical reaction outcomes were observed compared to in situ irradiation (cf. sections 4.6.2 and 4.6.3).

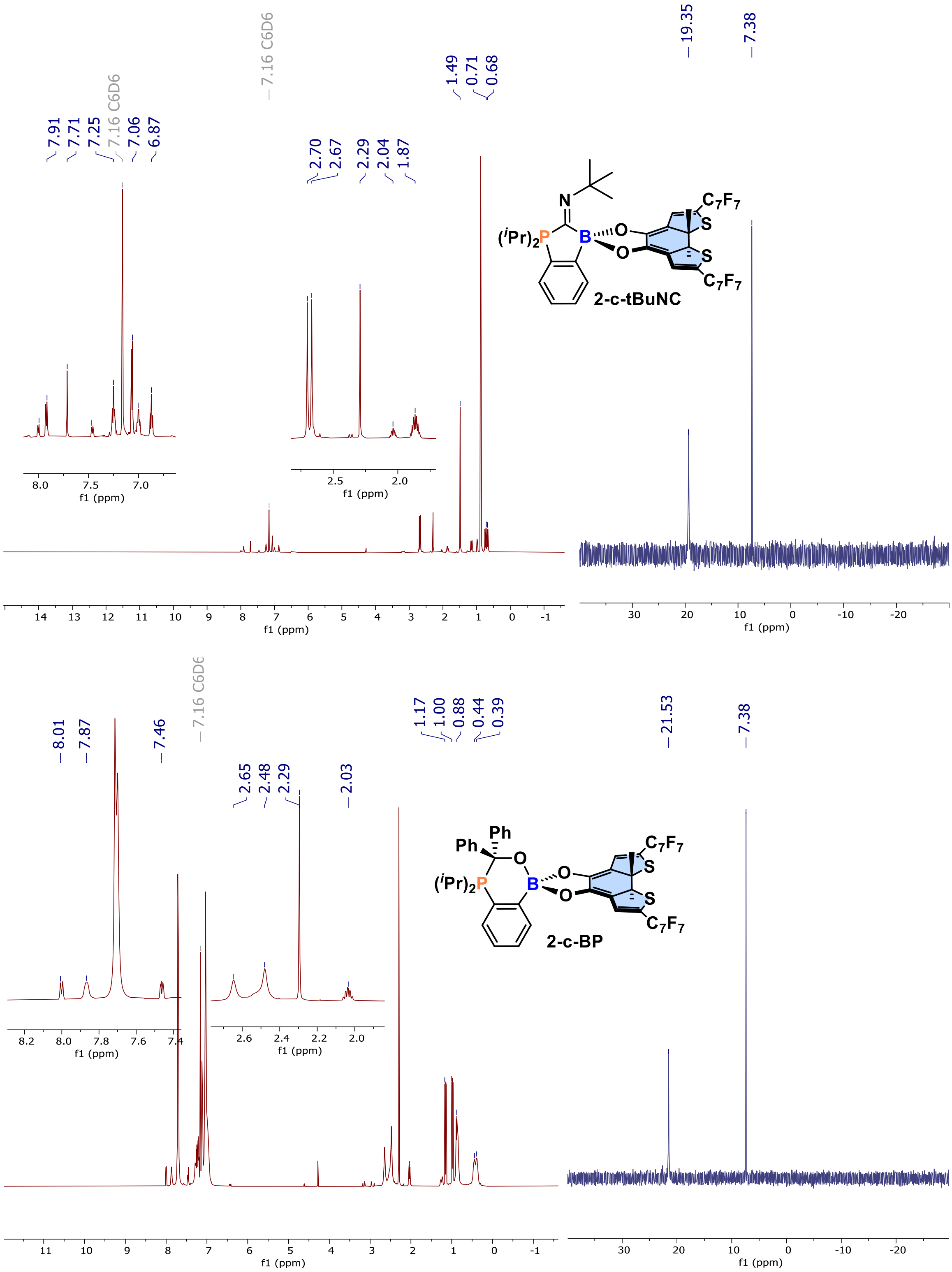

Figure S80. NMR spectra (C_6D_6) after adding excess tBuNC (top) or benzophenone (bottom) to a pre-irradiated solution of **2-c.** Left: ^{1}H NMR (600 MHz); Right: ^{31}P NMR (243 MHz).

4.7 Photocyclization (2-AuCl)

2-o-AuCl (15.4 mg) was dissolved in CD_2Cl_2 (0.5 ml) and analyzed by NMR spectroscopy before and after irradiation at 300 nm.

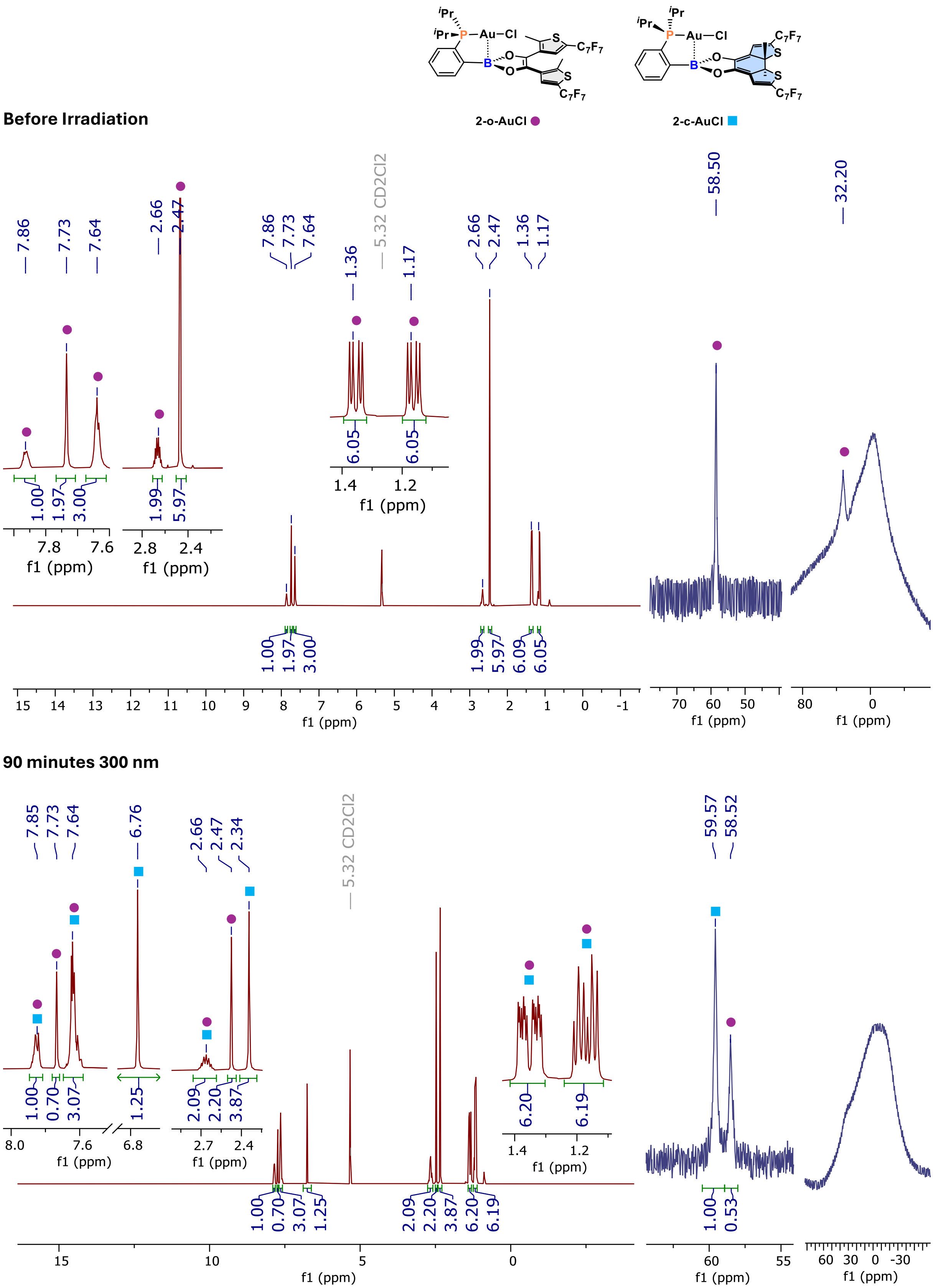

Figure S81. Top: NMR spectra (CD_2Cl_2) of **2-o-AuCl**. Left: ^{1}H NMR (600 MHz); Middle: ^{31}P NMR (243 MHz); Right: ^{11}B NMR (193 MHz). Bottom: NMR spectra (CD_2Cl_2) after 60% conversion to **2-c-AuCl.** Left: ^{1}H NMR (400 MHz); Middle: ^{31}P NMR (162 MHz); Right: ^{11}B NMR (128 MHz).

4.8 Photocyclization (2-Rh(CNCy)$_2$Cl)

2-o-Rh(CNCy)$_2$Cl (14.6 mg) was dissolved in C_6D_6 (0.5 ml) and analyzed by NMR spectroscopy before irradiation, after irradiation at 365 nm, and again after VIS irradiation. The minor isomer (assigned to the cis complex, details in section 1.5) is marked in the spectra below.

Before Irradiation

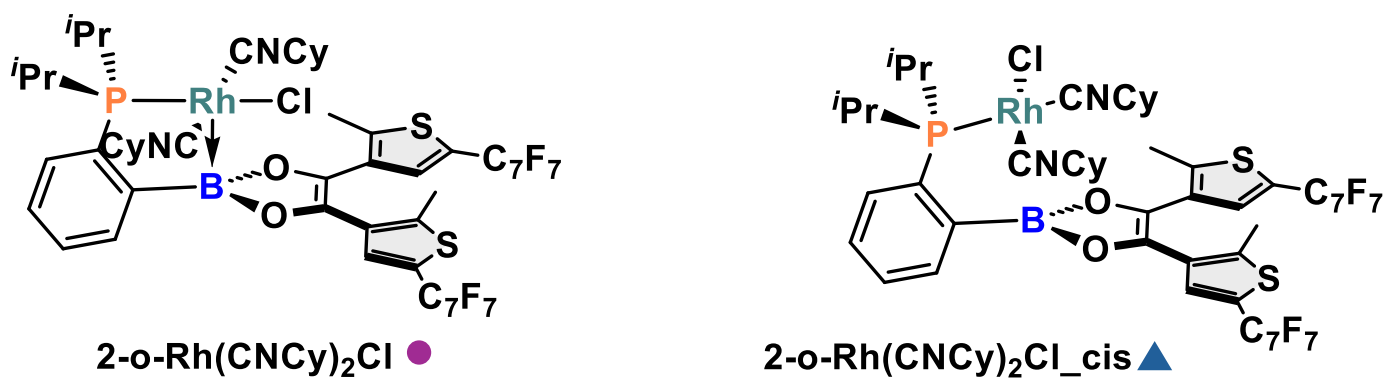

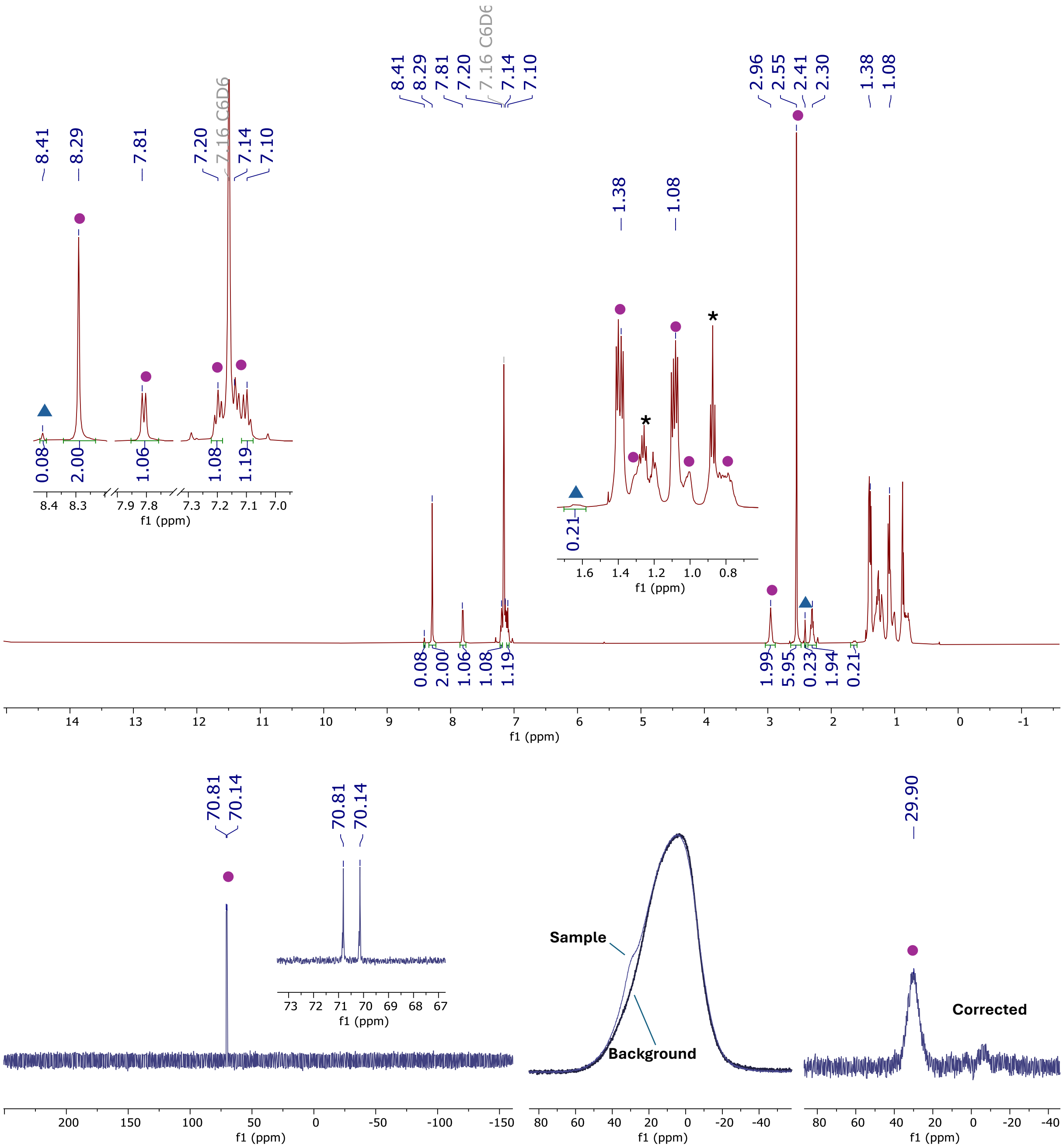

Figure S82. NMR spectra of **2-o-Rh(CNCy)$_2$Cl** as trans (major) and cis (minor) isomers. Top: ^1H NMR (600 MHz); Bottom left: ^{31}P NMR (243 MHz); Bottom Right: ^{11}B NMR (uncorrected and background corrected, 193 MHz). **n*-hexane

1h 365 nm

*Note: Assignment of the ¹H NMR resonances is based on integrals and which signals are plausible for **2-c-Rh(CNCy)₂Cl** cis and trans isomers. Line broadening complicates analysis and prevents full assignment. Thiophene-CH₃ signals (1) and cyclohexyl signals (2) are denoted below.*

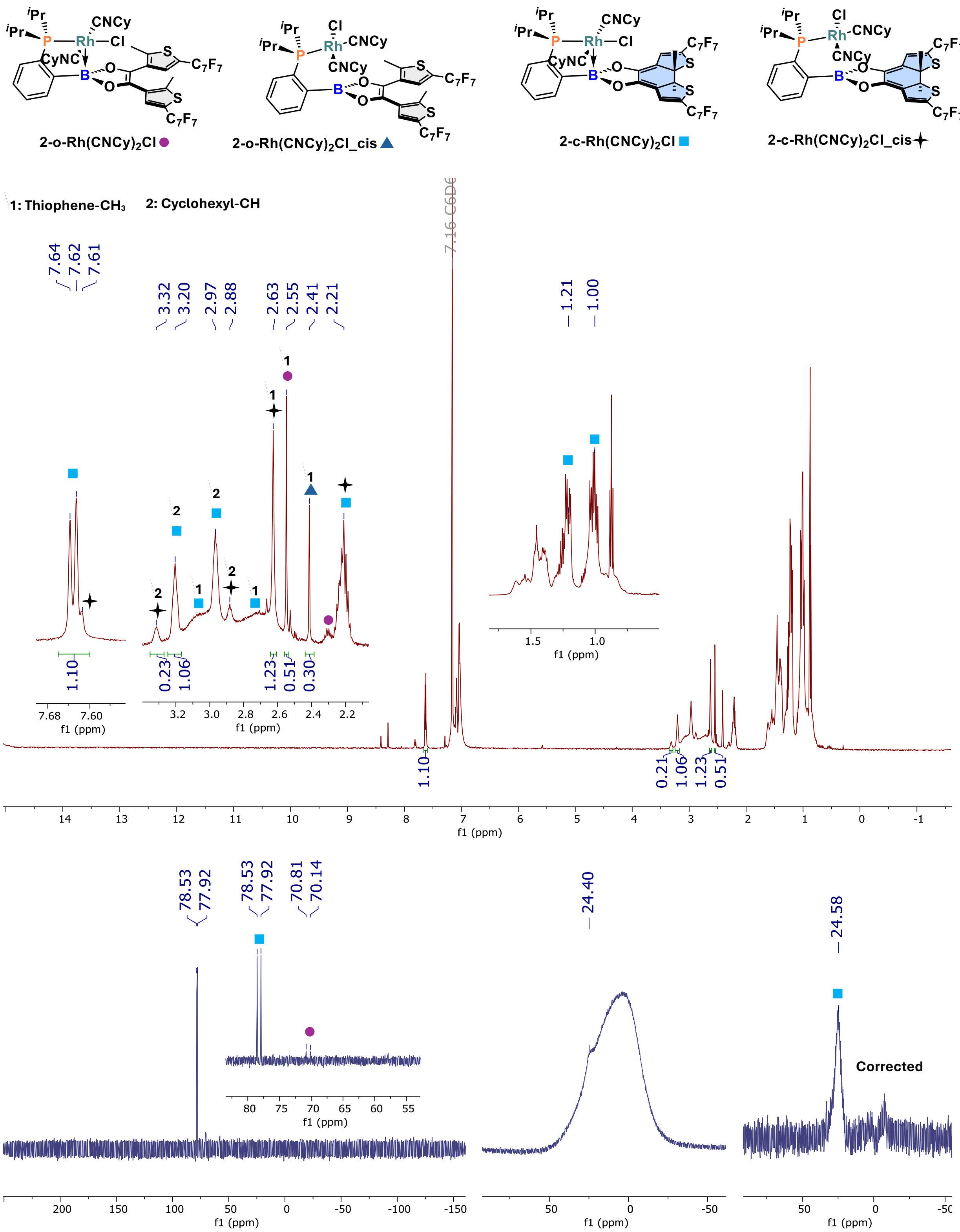

Figure S83. NMR spectra after irradiation at 365 nm, giving **2-c-Rh(CNCy)₂Cl** as trans (major) and cis (minor) isomers. Top: ¹H NMR (600 MHz); Bottom left: ³¹P NMR (243 MHz); Bottom Right: ¹¹B NMR (uncorrected and background corrected, 193 MHz). *n-hexane

1h VIS

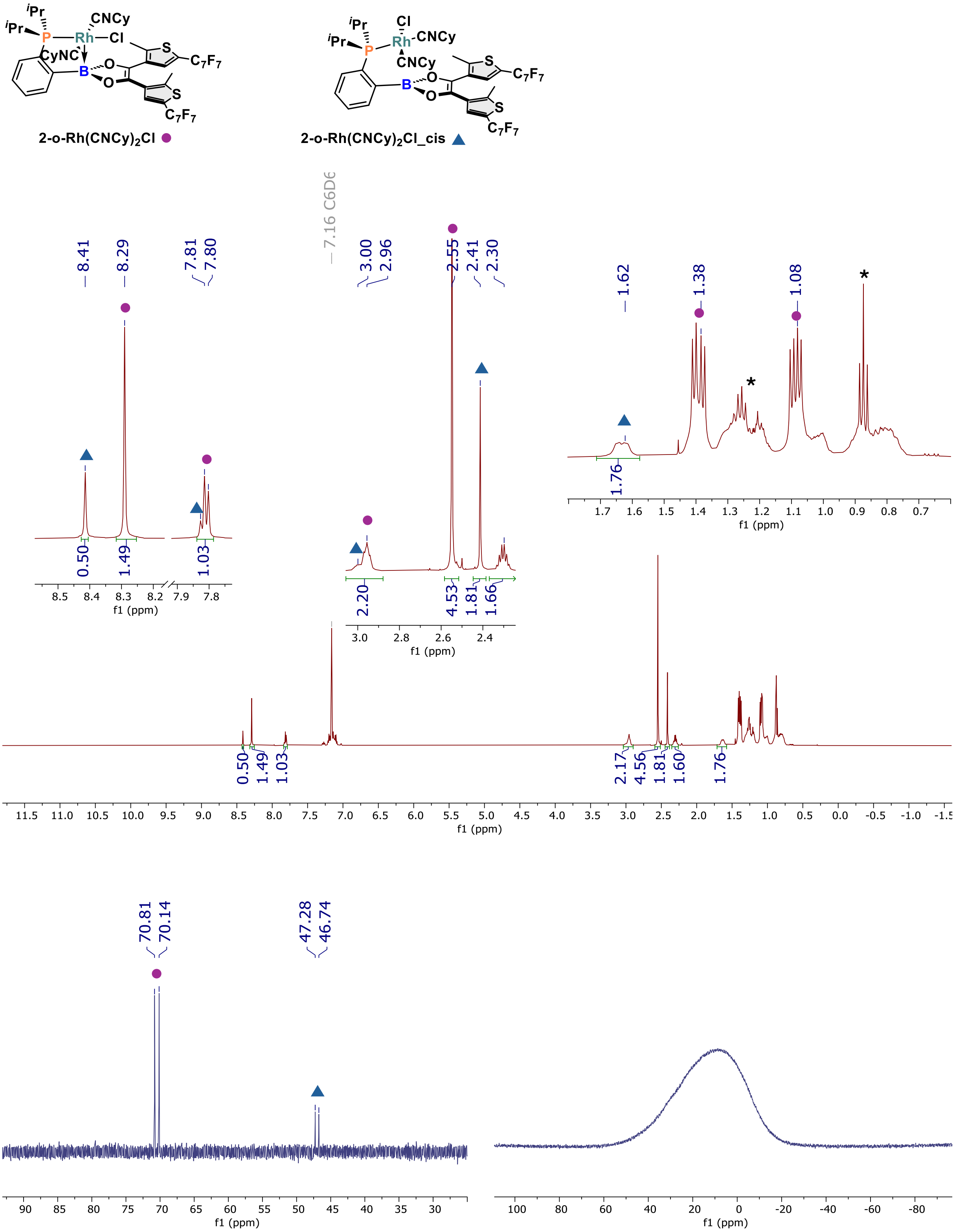

Figure S84. NMR spectra after VIS irradiation, restoring **2-o-Rh(CNCy)₂Cl** as trans (major) and cis (minor) isomers. Top: ^{1}H NMR (600 MHz); Bottom left: ^{31}P NMR (243 MHz); Bottom Right: ^{11}B NMR (193 MHz), no signal detected. **n*-hexane

IR spectra

2-o-Rh(CNCy)$_2$Cl

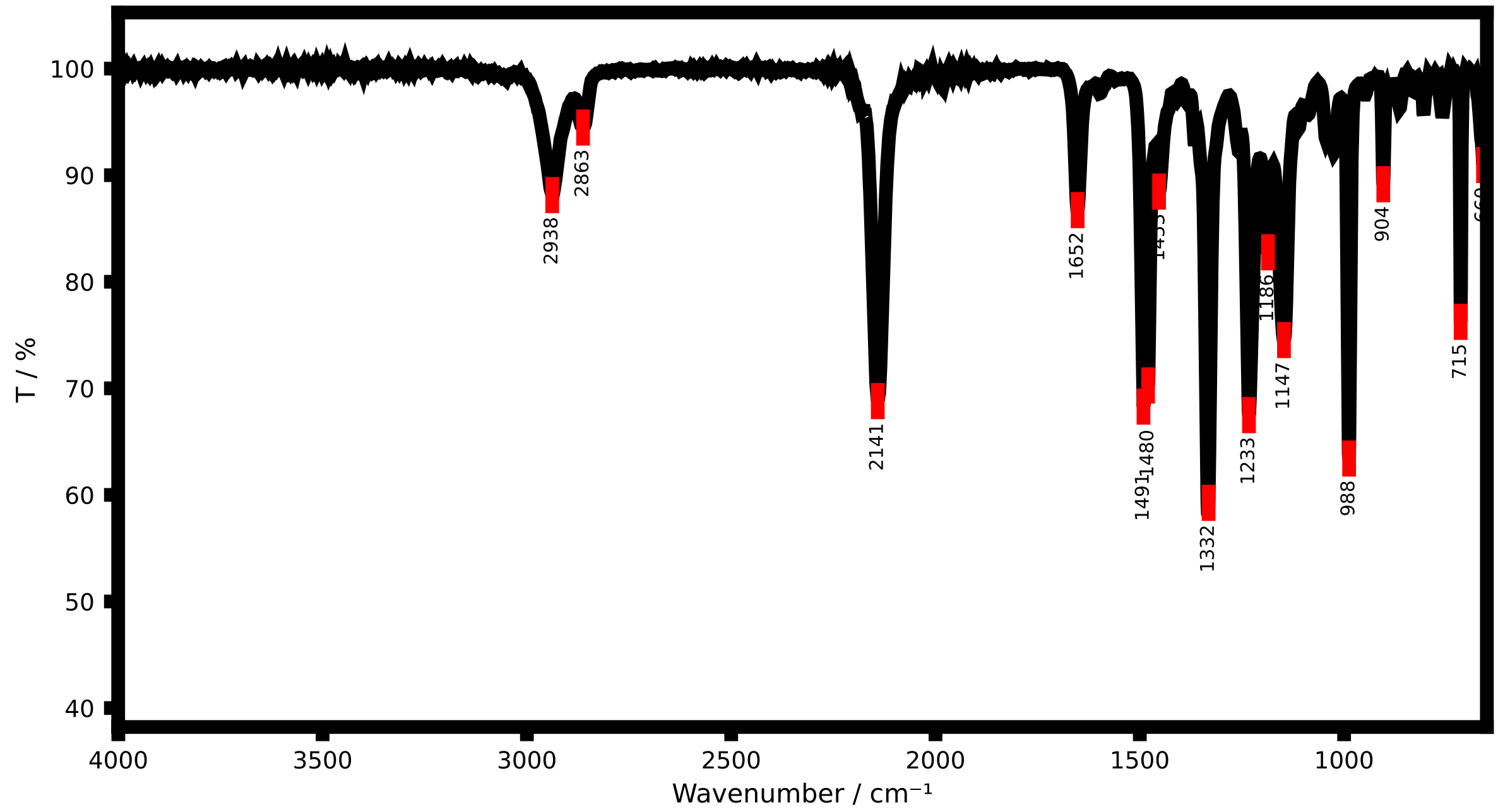

Figure S85. ATR-IR spectrum of **2-o-Rh(CNCy)$_2$Cl** (4% minor isomer)

1h 365 nm

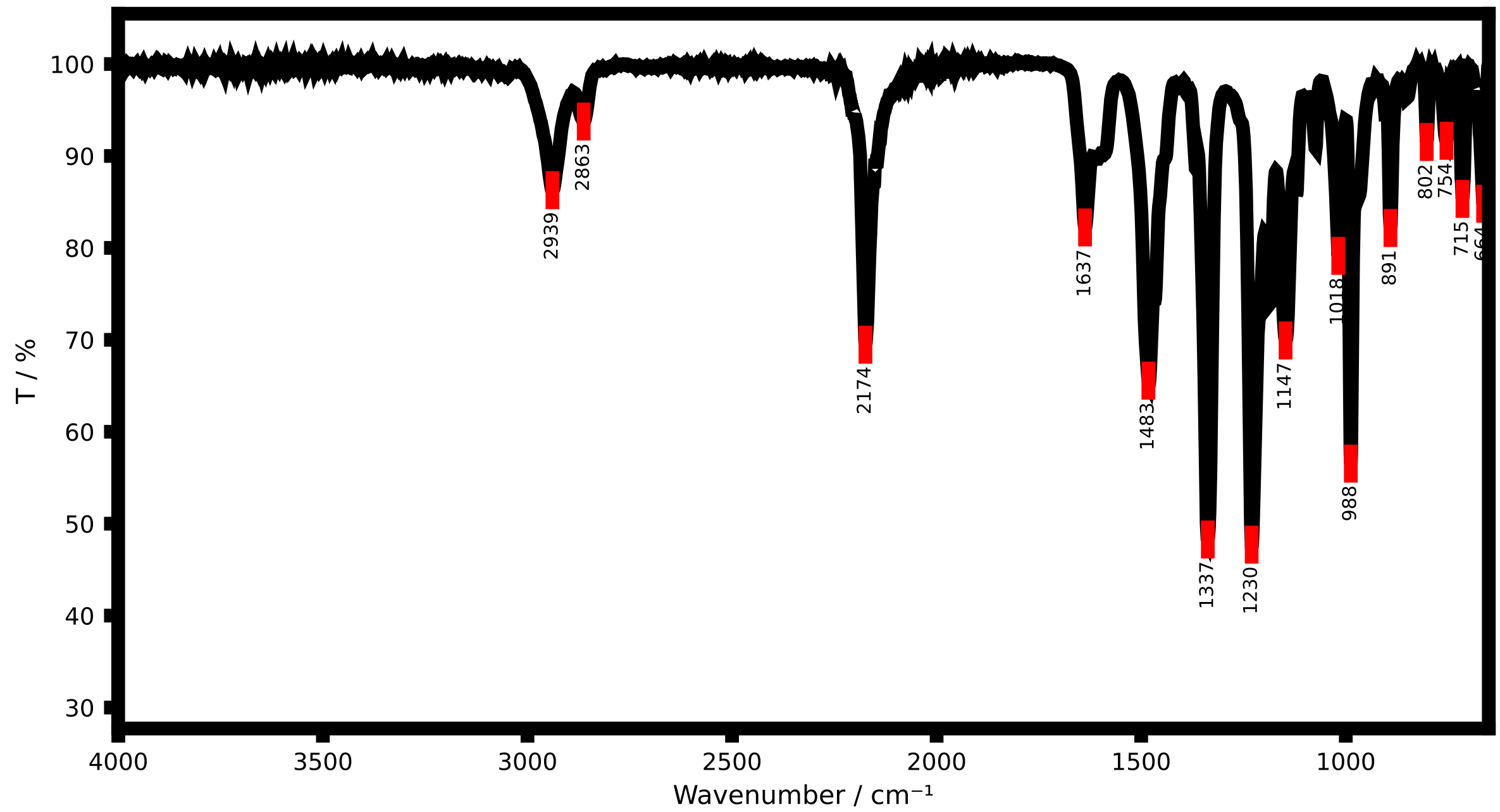

Figure S86. ATR-IR spectrum after irradiation at 365 nm (**2-c-Rh(CNCy)$_2$Cl**).

5 X-Ray Crystallography

Crystals were immersed in perfluorinated polyether oil and fixed on a cryo-loop. Data were collected from a shock-cooled single crystal at 100(2) K on a Bruker D8 VENTURE dual wavelength Mo/Cu four-circle diffractometer with a microfocus sealed X-ray tube using a mirror optics as monochromator and a Bruker PHOTON III detector. The diffractometer was equipped with an Oxford Cryostream 700 low temperature device and used MoK_{α} radiation (λ = 0.71073 Å). All data were integrated with SAINT and a multi-scan absorption correction using SADABS was applied.[14, 15] The structure was solved by direct methods using SHELXT and refined by full-matrix least-squares methods against F^2 by SHELXL-2019/2.[16, 17] All non-hydrogen atoms were refined with anisotropic displacement parameters. All hydrogen atoms were refined isotropic on calculated positions using a riding model with their U_{iso} values constrained to 1.5 times the U_{eq} of their pivot atoms for terminal sp^3 carbon atoms and 1.2 times for all other carbon atoms. Crystallographic data for the structures reported here have been deposited with the Cambridge Crystallographic Data Centre.[18] CCDC 2574720-2574723 contain the supplementary crystallographic data for this paper. These data can be obtained free of charge from The Cambridge Crystallographic Data Centre via www.ccdc.cam.ac.uk/structures. This report and the CIF file were generated using FinalCif. [19]

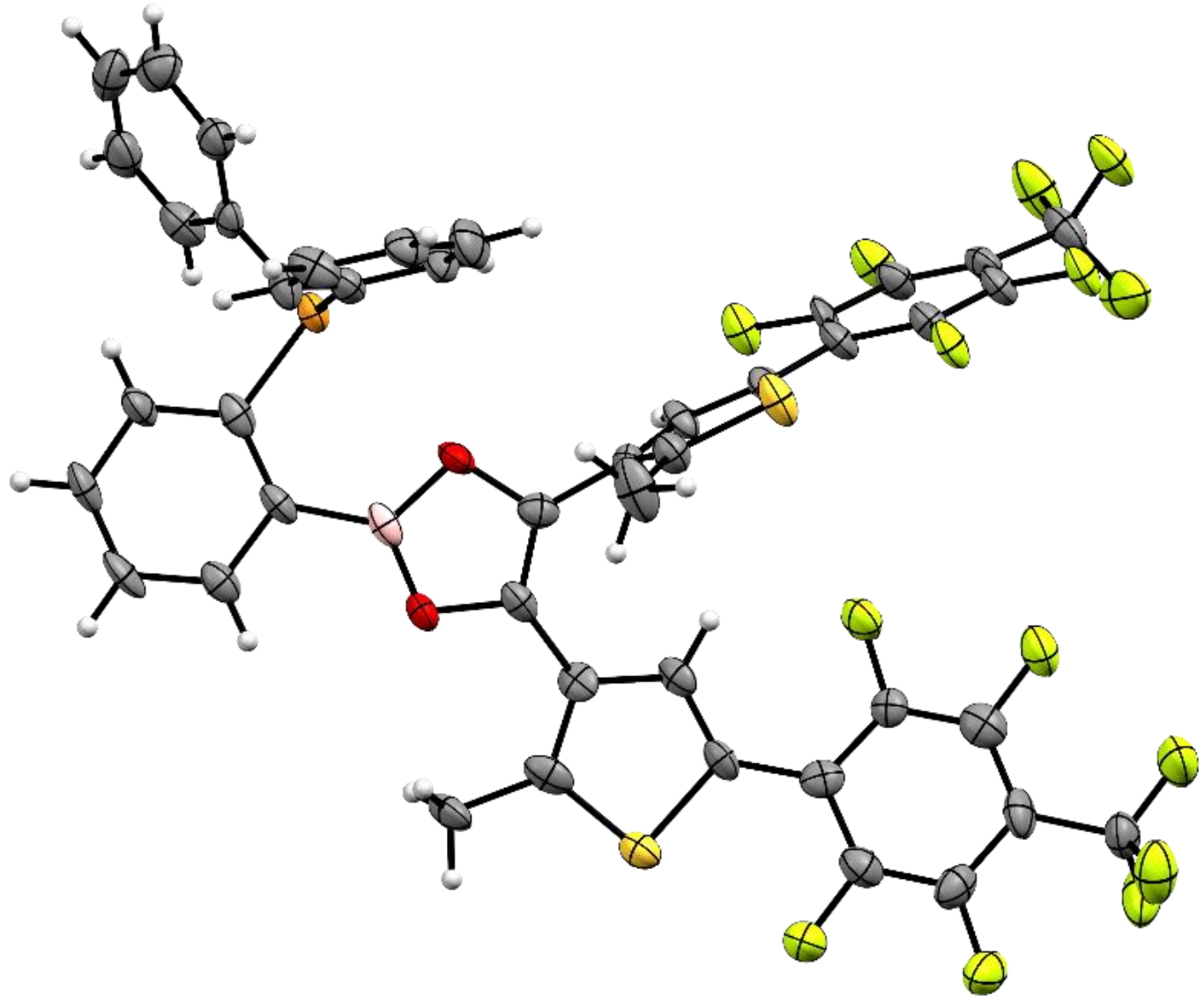

Figure S87. Low-quality solid-state structure of **1-o**, crystallized from n-hexane at –40 °C. Thermal ellipsoids are displayed at the 50% probability level. **Refinement data given for completeness below. R values preclude deposition into CCDC.**

**CCDC number**	------------------------
**Empirical formula**	$C_{43.91}H_{22}BF_{14}O_2PS_2$
**Formula weight**	953.39
**Temperature [K]**	293(2)
**Crystal system**	triclinic
**Space group (number)**	$P\overline{1}$ (2)
***a* [Å]**	11.438(3)
***b* [Å]**	18.560(4)
**c [Å]**	21.680(5)
**α [°]**	94.540(10)
**β [°]**	90.021(10)
**γ [°]**	90.025(9)
**Volume [Å^3]**	4588.3(18)
Z	4
**ρ_{calc} [gcm^{-3}]**	1.380
**μ [mm^{-1}]**	0.243
***F*(000)**	1918
**Crystal size [mm^3]**	0.04×0.11×0.41
**Crystal colour**	colourless
**Crystal shape**	needle
**Radiation**	MoK_α (λ=0.71073 Å)
**2θ range [°]**	4.03 to 50.42 (0.83 Å)
**Index ranges**	−13 ≤ h ≤ 13<br>−22 ≤ k ≤ 22<br>0 ≤ l ≤ 25
**Reflections collected**	16238
**Independent reflections**	16238<br>R_{int} = 0.2992<br>R_{sigma} = 0.2231
**Completeness to θ = 25.208°**	98.1 %
**Data / Restraints / Parameters**	16238 / 240 / 1222
**Absorption correction T_{min}/T_{max} (method)**	0.4549 / 0.7452<br>(multi-scan)
**Goodness-of-fit on F^2**	0.952
**Final *R* indexes [I≥2σ(I)]**	R_1 = 0.0826<br>wR_2 = 0.1761
**Final *R* indexes [all data]**	R_1 = 0.1679<br>wR_2 = 0.2079
**Largest peak/hole [eÅ^{-3}]**	0.67/−0.40

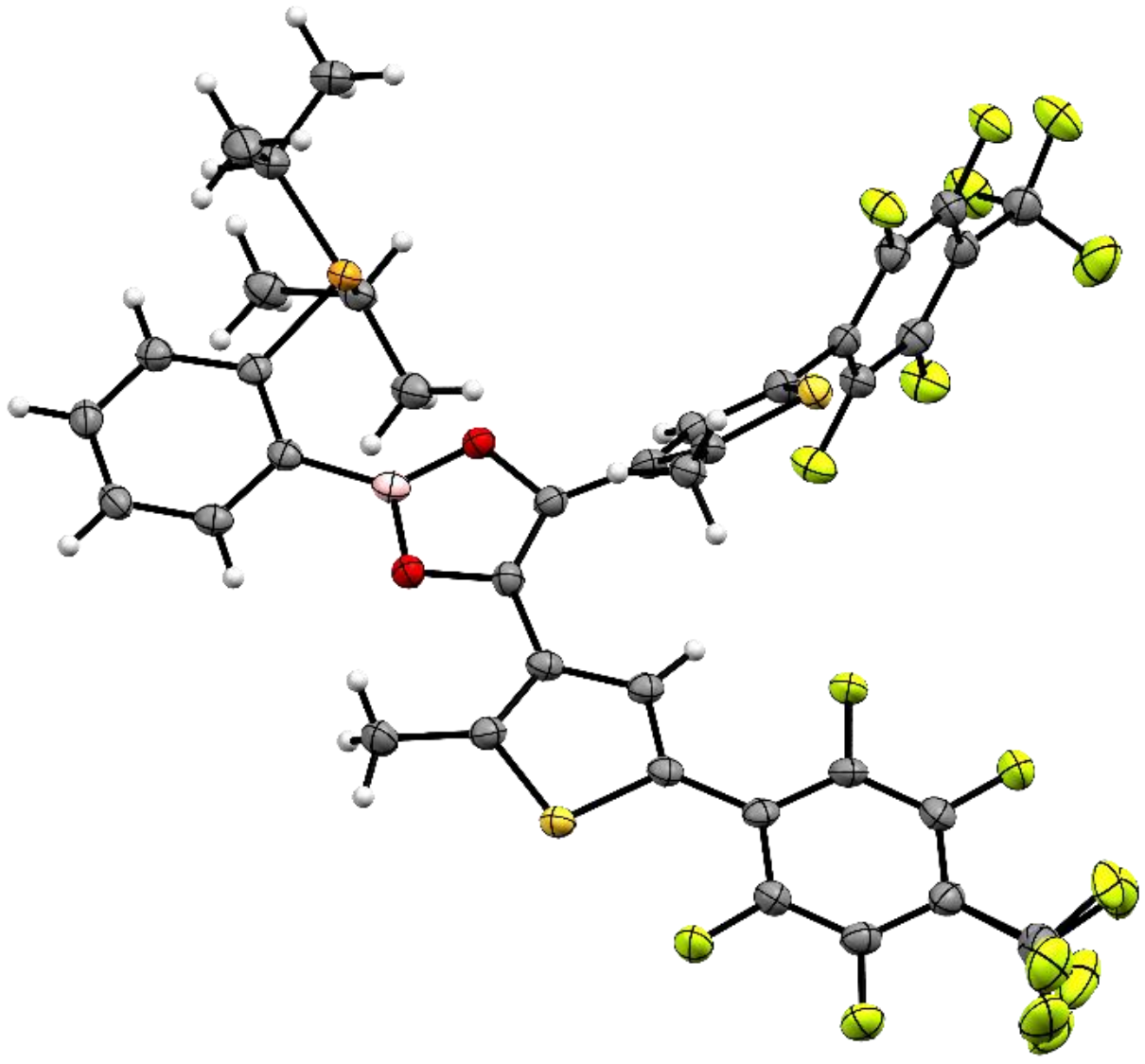

Figure S88. Solid-state structure of **2-o**, crystallized from DCM/n-hexane at –40 °C. Thermal ellipsoids are displayed at the 50% probability level.

**CCDC number**	2574720
**Empirical formula**	$C_{38}H_{26}BF_{14}O_2PS_2$
**Formula weight**	886.49
**Temperature [K]**	100(2)
**Crystal system**	monoclinic
**Space group (number)**	$P2_1/n$ (14)
***a* [Å]**	11.458(3)
***b* [Å]**	12.411(4)
**c [Å]**	25.904(8)
**α [°]**	90
**β [°]**	97.626(10)
**γ [°]**	90
**Volume [Å^3]**	3651.1(19)
Z	4
**ρ_{calc} [gcm^{-3}]**	1.613
**μ [mm^{-1}]**	0.298
***F*(000)**	1792
**Crystal size [mm^3]**	0.13×0.14×0.24
**Crystal colour**	colourless
**Crystal shape**	block
**Radiation**	MoK_α (λ=0.71073 Å)
**2θ range [°]**	4.11 to 50.20 (0.84 Å)
**Index ranges**	−13 ≤ h ≤ 13<br>−14 ≤ k ≤ 14<br>−30 ≤ l ≤ 30
**Reflections collected**	82872
**Independent reflections**	6477<br>R_{int} = 0.0650<br>R_{sigma} = 0.0295
**Completeness to θ = 25.098°**	99.5 %
**Data / Restraints / Parameters**	6477 / 120 / 566
**Absorption correction T_{min}/T_{max} (method)**	0.6668 / 0.7452<br>(multi-scan)
**Goodness-of-fit on F^2**	1.076
**Final *R* indexes [I≥2σ(I)]**	R_1 = 0.0411<br>wR_2 = 0.1012
**Final *R* indexes [all data]**	R_1 = 0.0525<br>wR_2 = 0.1091
**Largest peak/hole [eÅ^{-3}]**	0.37/−0.28

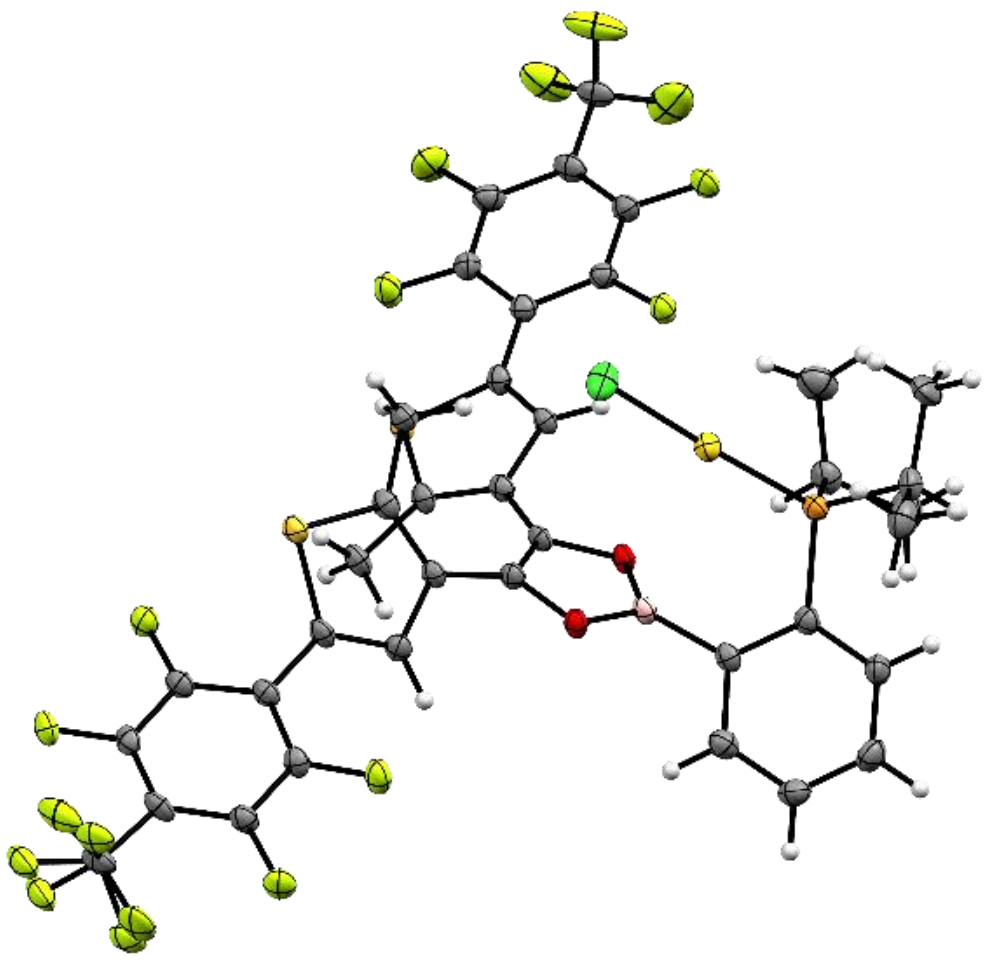

Figure S89. Solid-state structure of **2-o-AuCl**, crystallized from DCM/n-pentane at –40 °C. Thermal ellipsoids are displayed at the 50% probability level.

**CCDC number**	2574721
**Empirical formula**	$C_{38}H_{26}AuBClF_{14}O_2PS_2$
**Formula weight**	1118.90
**Temperature [K]**	100(2)
**Crystal system**	monoclinic
**Space group (number)**	$P2_1/n$ (14)
a **[Å]**	9.6834(7)
b **[Å]**	41.049(3)
**c [Å]**	9.7053(7)
**α [°]**	90
**β [°]**	93.010(2)
**γ [°]**	90
**Volume [Å³]**	3852.4(5)
Z	4
ρ_{calc} **[gcm⁻³]**	1.929
μ **[mm⁻¹]**	4.143
***F*(000)**	2176
**Crystal size [mm³]**	0.09×0.10×0.11
**Crystal colour**	green
**Crystal shape**	block
**Radiation**	MoK_α (λ=0.71073 Å)
**2θ range [°]**	3.97 to 61.47 (0.70 Å)
**Index ranges**	−13 ≤ h ≤ 13<br>−58 ≤ k ≤ 58<br>−13 ≤ l ≤ 13
**Reflections collected**	437275
**Independent reflections**	11918<br>R_{int} = 0.1004<br>R_{sigma} = 0.0282
**Completeness to θ = 25.242°**	100.0 %
**Data / Restraints / Parameters**	11918 / 180 / 576
**Absorption correction T_{min}/T_{max} (method)**	0.6047 / 0.7461 (multi-scan)
**Goodness-of-fit on F^2**	1.202
**Final *R* indexes [I≥2σ(I)]**	R_1 = 0.0575<br>wR_2 = 0.1166
**Final *R* indexes [all data]**	R_1 = 0.0678<br>wR_2 = 0.1208
**Largest peak/hole [eÅ⁻³]**	2.19/−4.00

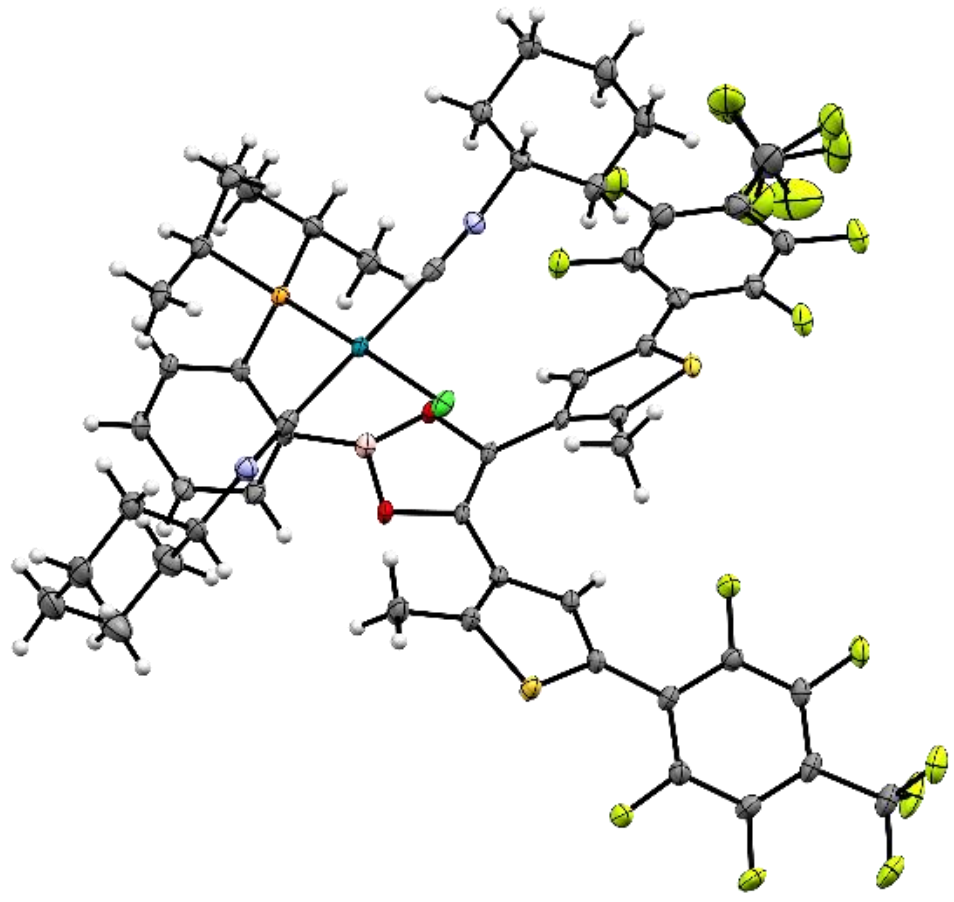

Figure S90. Solid-state structure of **2-o-Rh(CNCy)$_2$Cl**, crystallized from DCM/n-hexane at room temperature. Thermal ellipsoids are displayed at the 50% probability level.

**CCDC number**	2574722
**Empirical formula**	$C_{52}H_{48}BClF_{14}N_2O_2PRhS_2$
**Formula weight**	1243.18
**Temperature [K]**	100(2)
**Crystal system**	triclinic
**Space group (number)**	$P\overline{1}$ (2)
***a* [Å]**	13.3516(12)
***b* [Å]**	14.5974(14)
**c [Å]**	16.6065(17)
**α [°]**	64.753(3)
**β [°]**	69.319(4)
**γ [°]**	81.618(4)
**Volume [Å^3]**	2738.7(5)
Z	2
**ρ_{calc} [gcm^{-3}]**	1.508
**μ [mm^{-1}]**	0.555
***F*(000)**	1260
**Crystal size [mm^3]**	0.07×0.14×0.26
**Crystal colour**	yellow
**Crystal shape**	block
**Radiation**	MoK_α (λ=0.71073 Å)
**2θ range [°]**	4.48 to 52.74 (0.80 Å)
**Index ranges**	−16 ≤ h ≤ 16<br>−18 ≤ k ≤ 18<br>−20 ≤ l ≤ 20
**Reflections collected**	190669
**Independent reflections**	11204<br>R_{int} = 0.0905<br>R_{sigma} = 0.0312
**Completeness to θ = 25.242°**	99.9 %
**Data / Restraints / Parameters**	11204 / 174 / 719
**Absorption correction T_{min}/T_{max} (method)**	0.6894 / 0.7379 (multi-scan)
**Goodness-of-fit on F^2**	1.024
**Final *R* indexes [$I \geq 2\sigma(I)$]**	R_1 = 0.0330<br>wR_2 = 0.0804
**Final *R* indexes [all data]**	R_1 = 0.0389<br>wR_2 = 0.0849
**Largest peak/hole [eÅ^{-3}]**	0.43/−0.53

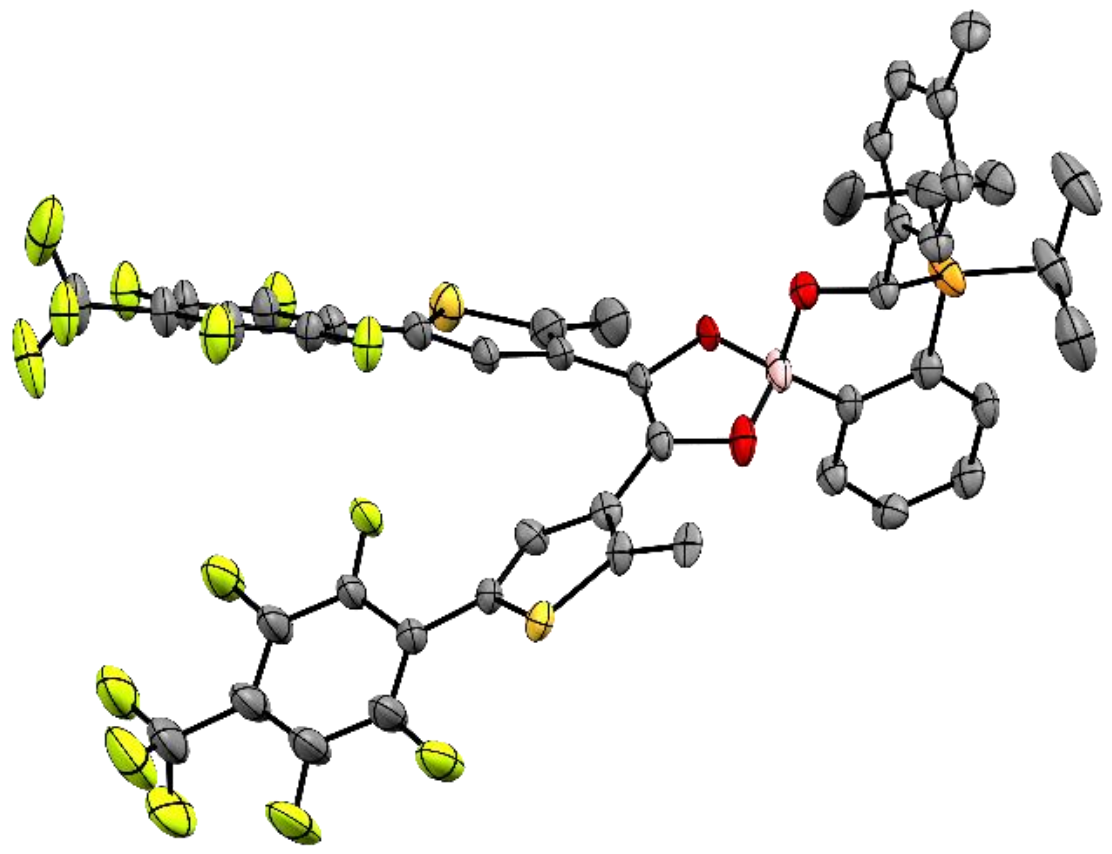

Figure S91. Solid-state structure of **2-o-TolCHO**, crystallized from DCM/n-pentane at -40 °C. Thermal ellipsoids are displayed at the 50% probability level. Disorder and hydrogen atoms are omitted for clarity.

**CCDC number**	2574723
**Empirical formula**	$C_{47.50}H_{37}BCl_3F_{14}O_3PS_2$
**Formula weight**	1134.02
**Temperature [K]**	100(2)
**Crystal system**	triclinic
**Space group (number)**	*P*1 (1)
***a* [Å]**	8.2736(5)
***b* [Å]**	17.4429(12)
***c* [Å]**	17.5188(11)
**α [°]**	73.771(2)
**β [°]**	87.095(2)
**γ [°]**	84.678(2)
**Volume [Å^3]**	2416.2(3)
Z	2
**ρ_{calc} [gcm^{-3}]**	1.559
**μ [mm^{-1}]**	0.406
***F*(000)**	1150
**Crystal size [mm^3]**	0.09×0.12×0.19
**Crystal colour**	red
**Crystal shape**	block
**Radiation**	MoK_α (λ=0.71073 Å)
**2θ range [°]**	3.88 to 54.41 (0.78 Å)
**Index ranges**	−10 ≤ h ≤ 10<br>−22 ≤ k ≤ 22<br>−22 ≤ l ≤ 22
**Reflections collected**	85858
**Independent reflections**	21349<br>R_{int} = 0.0596<br>R_{sigma} = 0.0511
**Completeness to θ = 25.242°**	99.7 %
**Data / Restraints / Parameters**	21349 / 9500 / 2010
**Absorption correction T_{min}/T_{max} (method)**	0.6756 / 0.7455 (multi-scan)
**Goodness-of-fit on *F*^{2}**	1.040
**Final *R* indexes [*I*≥2σ(*I*)]**	R_1 = 0.0661<br>wR_2 = 0.1669
**Final *R* indexes [all data]**	R_1 = 0.0825<br>wR_2 = 0.1788
**Largest peak/hole [eÅ^{-3}]**	0.79/-0.96

6 Coordinates

1-o

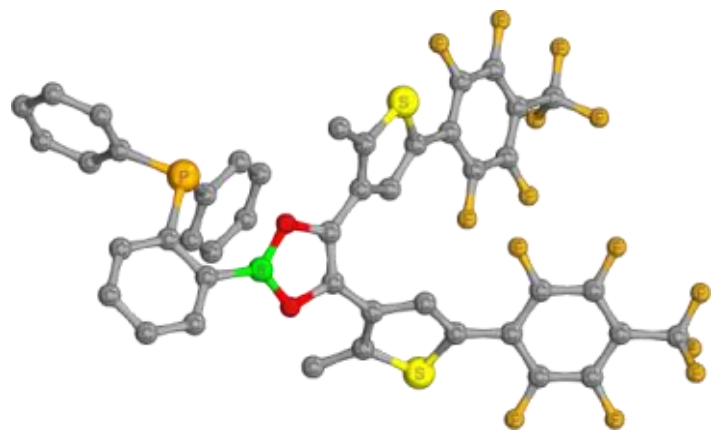

86
xyz, charge: 0, multiplicity: 1
C 14.314289 8.826357 5.098646
C 13.557852 9.886535 5.596009
C 14.803068 7.853946 5.993976
C 14.490660 7.965058 7.345043
C 13.711315 9.013291 7.814359
C 13.244638 9.981478 6.942421
H 13.199261 10.649199 4.915450
H 14.864678 7.236342 8.051586
H 13.481625 9.074561 8.870264
H 12.645698 10.805559 7.306491
P 15.770357 6.410691 5.367450
C 14.849747 9.955537 -5.311107
C 14.862312 11.156901 -6.004722
C 15.052788 9.916041 -3.942125
C 15.274409 11.062570 -3.184303
C 15.285135 12.260255 -3.897308
C 15.087829 12.308131 -5.259534
F 14.652708 8.798436 -5.923348
F 15.032313 8.720654 -3.363049
F 15.492082 13.405820 -3.254732
F 15.110301 13.491566 -5.855870
C 14.650138 11.288006 -7.490985
C 15.495986 11.011266 -1.745766
C 15.065669 10.064648 -0.858858
C 15.504883 10.292270 0.469120
C 16.269963 11.425586 0.586135
S 16.431103 12.210195 -0.920396
H 14.436688 9.228526 -1.120129
C 16.918839 11.978191 1.809143
H 16.194811 12.490516 2.443151
H 17.707107 12.687523 1.561669
H 17.361694 11.176178 2.399902
C 15.206367 9.385858 1.559544
F 14.444942 10.120970 -8.088201
F 15.711168 11.851921 -8.069898
F 13.592034 12.058942 -7.748506
H 13.300655 5.645550 1.536401
S 16.047968 4.864120 -0.589184
C 19.394477 4.888061 -3.663851
C 18.339770 7.300387 -2.889510
C 15.234601 5.743746 0.626838
C 18.500528 4.968487 -2.611116
C 14.219438 5.062786 1.482671
C 15.224399 8.042387 1.614389
C 15.674900 7.044368 0.668746
C 19.785695 6.025372 -4.354558
C 17.938414 6.166704 -2.180224
C 20.754007 6.034242 -5.508840
C 16.678122 7.310022 -0.296825
C 16.989825 6.231516 -1.077245
C 19.232393 7.229924 -3.937015
F 19.858280 3.687420 -3.971698
F 19.555773 8.356329 -4.557372
F 18.176260 3.834110 -1.996131
F 17.863779 8.500249 -2.574446
F 21.211495 4.824808 -5.807089
F 20.175466 6.524148 -6.606102
F 21.809252 6.802448 -5.231277
H 17.167669 8.267156 -0.367591
H 14.582569 4.937239 2.503861
H 13.964532 4.078923 1.091637
O 14.836175 9.882275 2.781913
O 14.805887 7.644710 2.850350
B 14.633030 8.787267 3.587908
C 17.170828 7.246915 4.524760
C 17.619588 8.529164 4.835537
C 17.772993 6.564008 3.470890
C 18.649787 9.109960 4.109257
C 18.803032 7.141914 2.745436
C 19.241991 8.418892 3.061947
H 17.158489 9.085309 5.642610
H 17.417267 5.577238 3.199889
H 18.992322 10.104382 4.366402
H 19.250005 6.599824 1.922630
H 20.041464 8.875528 2.493155
C 16.544529 5.778457 6.903424
C 15.928600 4.682328 7.504684
C 17.695725 6.303023 7.485674
C 16.433172 4.138161 8.676588
C 18.208906 5.751968 8.649483
C 17.576338 4.672767 9.250366
H 15.045680 4.249852 7.048693
H 18.202526 7.141761 7.027121
H 15.939995 3.290463 9.133855
H 19.106780 6.166638 9.088880
H 17.978803 4.245052 10.159232

1-o-H

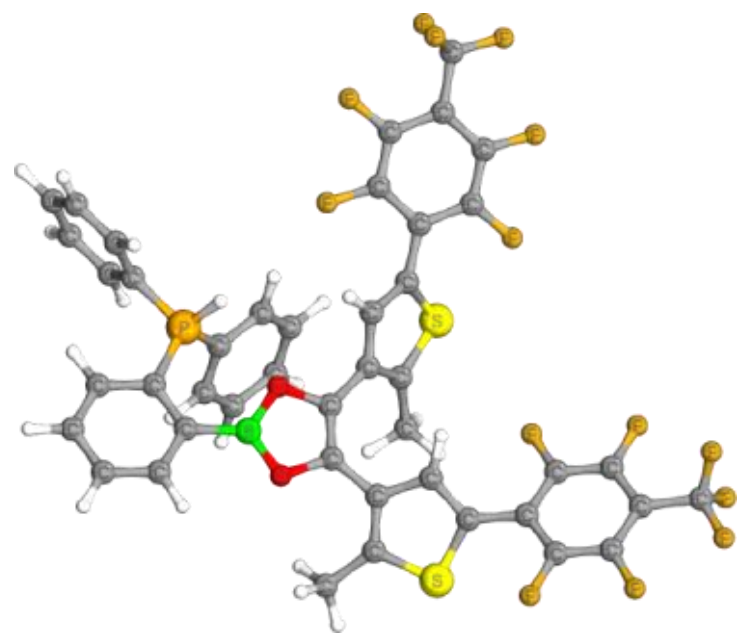

87
xyz, charge: 1, multiplicity: 1
C 15.253817 9.809901 5.784376
C 15.265308 8.909282 6.844749
C 14.847826 11.133244 6.050976
C 14.471173 11.518360 7.333430
C 14.499283 10.596720 8.370415
C 14.897626 9.294557 8.126438
H 15.571168 7.886344 6.667848
H 14.153385 12.531688 7.538807
H 14.207205 10.903056 9.365311
H 14.921352 8.574719 8.933252
P 14.760508 12.385875 4.754066
C 18.164747 15.317418 -0.603511
C 18.680217 15.403129 -1.892475
C 17.552629 14.170386 -0.148543
C 17.426372 13.040435 -0.950021
C 17.937412 13.134925 -2.236030
C 18.551158 14.285834 -2.705314
F 18.244328 16.353621 0.217595
F 17.059838 14.166705 1.092068
F 17.844376 12.093203 -3.051068
F 19.007086 14.264427 -3.943380
C 19.336345 16.696184 -2.319296
C 16.781339 11.828931 -0.456058
C 16.907321 11.264925 0.777222
C 16.107898 10.102513 0.944879
C 15.362851 9.804938 -0.169840
S 15.681914 10.920392 -1.425907
H 17.573729 11.640909 1.539643
C 14.368151 8.715698 -0.364834

H	13.900744	8.456437	0.584514
H	14.836741	7.812637	-0.758631
H	13.582163	9.009280	-1.059486
C	16.014075	9.405755	2.208600
F	19.795804	16.653009	-3.560074
F	20.361169	16.980215	-1.516800
F	18.465536	17.702086	-2.243978
H	13.845326	6.055663	3.293193
S	16.052087	4.524056	0.916506
C	19.109020	3.696187	-2.410650
C	17.859223	6.138943	-2.231532
C	15.561042	5.688504	2.064331
C	18.410025	4.052493	-1.269083
C	14.620847	5.303046	3.157411
C	15.953547	8.111831	2.574282
C	16.150661	6.902950	1.806937
C	19.199521	4.567423	-3.486963
C	17.771493	5.277634	-1.142052
C	19.942808	4.264793	-4.767130
C	16.993348	6.871838	0.663931
C	17.038812	5.650051	0.063220
C	18.557334	5.795353	-3.369353
F	19.683345	2.506881	-2.420746
F	18.596846	6.664941	-4.366840
F	18.358783	3.184249	-0.266171
F	17.250380	7.321293	-2.196999
F	20.517973	3.071395	-4.756564
F	19.110300	4.302901	-5.806657
F	20.897456	5.171346	-4.973192
H	17.561768	7.725857	0.326597
H	15.143731	5.189726	4.107221
H	14.125133	4.358140	2.941527
O	15.887753	10.185633	3.338199
O	15.707559	8.037412	3.925236
B	15.643720	9.325662	4.368058
H	16.025100	12.568047	4.187170
C	13.626576	11.943988	3.447160
C	12.553554	11.097599	3.706865
C	13.845677	12.453587	2.170440
C	11.693385	10.760747	2.673499
C	12.984376	12.104286	1.145393
C	11.911316	11.260133	1.397365
H	12.390825	10.693186	4.698455
H	14.690522	13.097746	1.961594
H	10.855081	10.105257	2.865278
H	13.159164	12.482952	0.147953
H	11.240848	10.988651	0.593100
C	14.327018	13.972595	5.454149
C	13.016176	14.436606	5.390313
C	15.319781	14.731570	6.073338
C	12.699942	15.660530	5.957521
C	14.993878	15.953418	6.634743
C	13.685117	16.414733	6.577818
H	12.247866	13.858298	4.893141
H	16.343177	14.377992	6.118512
H	11.684521	16.028384	5.908134
H	15.760674	16.548668	7.110692
H	13.434326	17.372204	7.013816

1-o-F

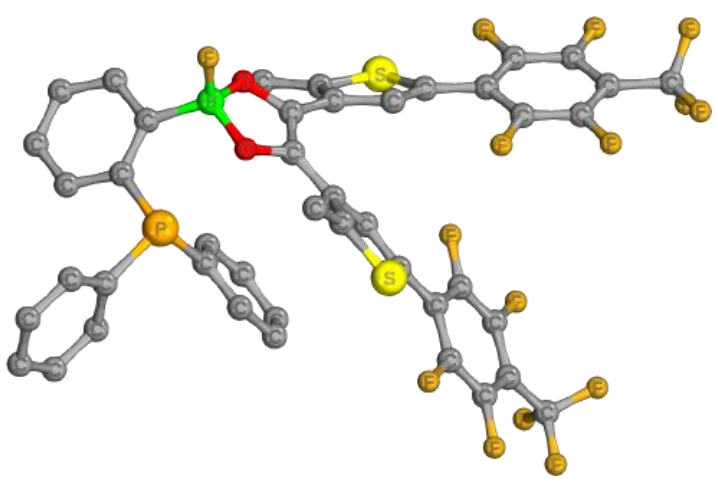

87
xyz, charge: -1, multiplicity: 1

C	12.820872	9.606714	5.056126
C	12.091725	8.465786	5.405598
C	13.764087	10.076215	5.985594
C	13.937260	9.413465	7.204160
C	13.191999	8.291111	7.522301
C	12.260075	7.812853	6.614246
H	11.361173	8.082015	4.702777
H	14.663329	9.769634	7.922105
H	13.344440	7.794689	8.472905
H	11.668517	6.935333	6.847931
P	14.734423	11.605504	5.616314
C	20.644785	12.461431	-1.022043
C	20.801801	11.364974	-1.858425
C	19.465966	12.670815	-0.332097
C	18.363885	11.820292	-0.422339
C	18.538803	10.723937	-1.273989
C	19.712441	10.506258	-1.960270
F	21.618671	13.345068	-0.840839
F	19.409816	13.741646	0.458474
F	17.557674	9.847686	-1.458453
F	19.788017	9.441704	-2.752150
C	22.044348	11.057891	-2.642984
C	17.134375	12.047891	0.307784
C	16.217173	11.114618	0.710640
C	15.128922	11.646041	1.442818
C	15.237823	13.009401	1.603667
S	16.660312	13.613203	0.872397
H	16.334092	10.055573	0.552209
C	14.328728	13.939465	2.331654
H	14.398136	13.785382	3.410034
H	13.290649	13.759184	2.057625
H	14.565595	14.980949	2.113487
C	14.080189	10.836595	2.027384
F	23.012531	11.950232	-2.454943
F	21.791277	11.025459	-3.954958
F	22.542601	9.863363	-2.307595
H	14.451266	6.936132	2.464591
S	14.348032	6.898664	-1.109614
C	14.803432	7.936666	-5.501337
C	15.069638	9.957246	-3.689241
C	14.005450	7.489897	0.456773
C	14.519638	7.721777	-4.172513
C	13.888552	6.556250	1.612229
C	13.577244	9.643721	1.646541
C	13.877627	8.858426	0.462401
C	15.226704	9.174278	-5.971328
C	14.638764	8.721441	-3.202940
C	15.511791	9.334893	-7.436952
C	14.065783	9.412261	-0.826656
C	14.351319	8.489107	-1.799806
C	15.352339	10.181665	-5.024780
F	14.655041	6.923023	-6.348469
F	15.756900	11.399680	-5.359075
F	14.115555	6.503153	-3.820071
F	15.232589	10.986941	-2.867156
F	14.424124	9.070744	-8.169123
F	15.910573	10.560707	-7.759976
F	16.469288	8.491906	-7.835968
H	13.961449	10.469926	-1.004859
H	14.262681	5.562131	1.369238
H	12.852514	6.465939	1.936890
O	13.518282	11.246386	3.178601
O	12.692194	9.172086	2.536475
B	12.524421	10.227359	3.586092
C	15.602764	11.874218	7.224332
C	16.863004	11.379023	7.553518
C	14.946344	12.675594	8.158514
C	17.443928	11.670818	8.779892
C	15.515875	12.956336	9.390562
C	16.771883	12.456399	9.704320
H	17.395839	10.755022	6.847645
H	13.971739	13.081897	7.912644
H	18.424895	11.276813	9.015115
H	14.983373	13.574413	10.102560
H	17.225256	12.681545	10.661419
C	16.137959	10.969754	4.618879
C	16.282199	9.638938	4.244194
C	17.092365	11.890586	4.186866
C	17.368040	9.235656	3.477264
C	18.183096	11.489174	3.434538
C	18.325148	10.154816	3.078588

H 15.542529 8.911396 4.552569
H 16.980015 12.937546 4.445156
H 17.462272 8.195732 3.189726
H 18.908255 12.222380 3.105104
H 19.169028 9.838883 2.477847
F 11.226152 10.748717 3.532807

1-c

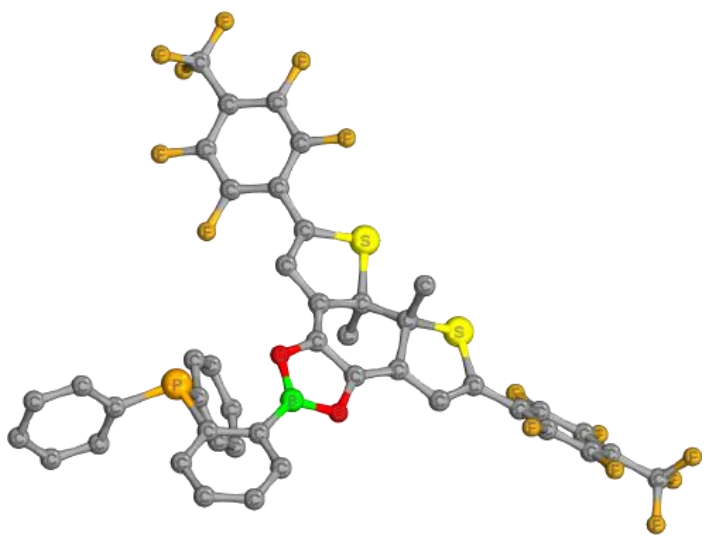

86
xyz, charge: 0, multiplicity: 1
C -2.853874 -0.475739 3.081525
C -2.783990 -1.654217 3.826780
C -3.492462 0.652096 3.638147
C -4.008777 0.559732 4.926755
C -3.902129 -0.613425 5.660454
C -3.291883 -1.728133 5.112845
H -2.315439 -2.530435 3.396172
H -4.511854 1.407201 5.371890
H -4.311315 -0.653076 6.661669
H -3.215677 -2.647235 5.678057
P -3.553461 2.255084 2.725938
C -1.051450 6.264645 -3.508618
C 0.053176 6.575253 -4.294434
C -1.263409 4.989856 -3.029002
C -0.377766 3.950287 -3.303318
C 0.717900 4.267509 -4.094913
C 0.936657 5.544594 -4.582897
F -1.939348 7.203476 -3.213118
F -2.357114 4.767682 -2.307628
F 1.602351 3.324967 -4.392801
F 2.018462 5.735227 -5.319932
C 0.214394 7.994770 -4.778484
C -0.603964 2.597449 -2.804887
C -1.069110 2.277775 -1.577710
C -1.241510 0.878210 -1.389481
C -1.219862 0.103384 -2.700385
S -0.289805 1.207173 -3.843206
H -1.253392 2.996331 -0.792955
C -2.662847 0.006926 -3.212603
H -3.276293 -0.594933 -2.541266
H -2.708565 -0.431996 -4.206762
H -3.097867 1.004247 -3.256151
C -1.513800 0.168925 -0.285031
F 1.309256 8.170700 -5.506358
F 0.281277 8.836844 -3.746411
F -0.827118 8.355319 -5.529412
H 1.327535 -2.162081 -1.922492
S -0.730601 -2.388418 -3.927268
C -0.537069 -7.457879 -3.185960
C -1.581443 -5.409173 -4.659541
C -0.548774 -1.259577 -2.484504
C -0.550046 -6.176718 -2.676951
C 0.943597 -1.167164 -2.143718
C -1.618765 -1.261461 -0.294296
C -1.252565 -2.001053 -1.352746
C -1.047059 -7.749326 -4.446482
C -1.080425 -5.109866 -3.399200
C -0.997760 -9.177330 -4.929584
C -1.343820 -3.402522 -1.587426
C -1.088878 -3.750139 -2.867974
C -1.569303 -6.692571 -5.178687
F -0.011607 -8.423246 -2.444931
F -2.081721 -6.862793 -6.386540
F -0.022407 -5.975787 -1.476167
F -2.106904 -4.442744 -5.401185
F 0.261810 -9.611974 -4.981842
F -1.668527 -9.976321 -4.098357
F -1.522184 -9.331737 -6.138248
H -1.637276 -4.103073 -0.819814
H 1.105520 -0.536723 -1.268753
H 1.520629 -0.755610 -2.968894
O -1.810993 0.603610 0.962750
O -2.110223 -1.653220 0.908503
B -2.256274 -0.498452 1.662740
C -4.471421 1.802357 1.203804
C -5.261398 0.663627 1.063710
C -4.316637 2.648883 0.108497
C -5.873406 0.376450 -0.148693
C -4.936242 2.369747 -1.099119
C -5.712549 1.227839 -1.231627
H -5.400348 -0.009556 1.900744
H -3.696503 3.532839 0.198812
H -6.480761 -0.514226 -0.244416
H -4.798413 3.037352 -1.939478
H -6.187511 1.000078 -2.176898
C -4.820277 3.180136 3.672877
C -6.194345 3.000108 3.532302
C -4.355965 4.133896 4.576192
C -7.081764 3.748689 4.289191
C -5.242860 4.875702 5.342878
C -6.608196 4.683961 5.198998
H -6.576880 2.276683 2.824084
H -3.289698 4.298210 4.678090
H -8.147151 3.602360 4.167983
H -4.867163 5.609952 6.043289
H -7.303325 5.267204 5.788564

1-c-H

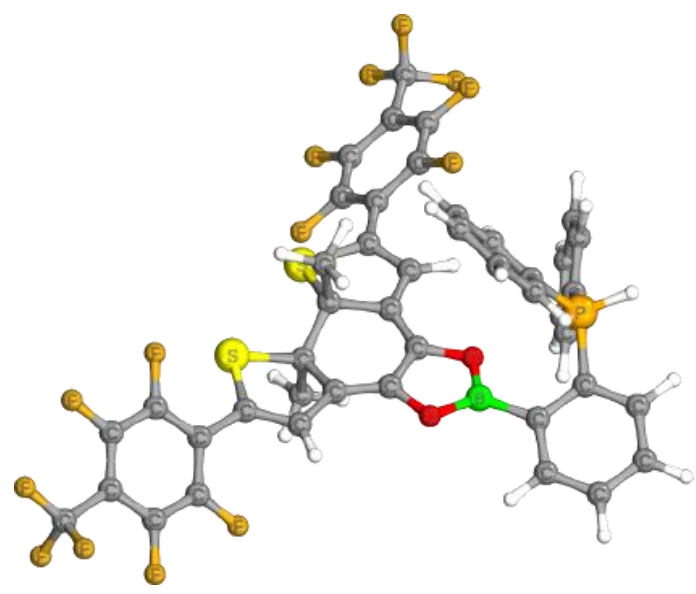

87
xyz, charge: 1, multiplicity: 1
C 16.216763 9.723120 5.971262
C 17.180694 10.186910 6.859512
C 15.030705 9.188581 6.505407
C 14.823291 9.144508 7.880319
C 15.790586 9.640711 8.742471
C 16.971521 10.151175 8.232063
H 18.106211 10.595214 6.474884
H 13.917598 8.717925 8.293809
H 15.622808 9.611415 9.810136
H 17.734177 10.522879 8.902697
P 13.786169 8.383174 5.484329
C 23.468000 11.299514 -0.365006
C 23.858237 10.610993 -1.508471
C 22.168963 11.257695 0.096142
C 21.188259 10.521130 -0.562712
C 21.583934 9.843423 -1.707427
C 22.886472 9.879889 -2.176665
F 24.348007 12.023288 0.307728
F 21.863243 11.954996 1.182741
F 20.695193 9.121855 -2.377674
F 23.154500 9.189273 -3.269917
C 25.302751 10.704498 -1.945122
C 19.808047 10.481949 -0.083926
C 19.421246 10.406601 1.209672
C 18.007552 10.414034 1.374369
C 17.275731 10.870731 0.112655
S 18.484287 10.557286 -1.235922

H 20.107392 10.299009 2.036223
C 17.071452 12.388808 0.216660
H 18.026463 12.872850 0.415094
H 16.387929 12.637889 1.029227
H 16.667798 12.803633 -0.704068
C 17.235480 10.170851 2.444262
F 25.553453 10.001800 -3.039729
F 26.105052 10.252647 -0.982228
F 25.629769 11.971072 -2.193018
H 16.775441 8.124805 0.446434
S 14.865392 10.751541 -1.373445
C 9.785644 9.981801 -0.951283
C 11.788447 11.585149 -1.892399
C 15.966985 10.084355 -0.055917
C 11.062730 9.859664 -0.450793
C 16.179647 8.591624 -0.339193
C 15.807530 10.157736 2.363871
C 15.131779 10.212463 1.209448
C 9.474773 10.909259 -1.939929
C 12.104197 10.671179 -0.896632
C 8.047335 10.985239 -2.431496
C 13.737594 10.304372 0.951761
C 13.451005 10.557415 -0.345482
C 10.507556 11.708422 -2.408316
F 8.845960 9.181360 -0.467488
F 10.319221 12.619008 -3.344279
F 11.283802 8.924700 0.472064
F 12.732335 12.386307 -2.364014
F 7.669735 9.814410 -2.940026
F 7.229622 11.276003 -1.418652
F 7.874581 11.907092 -3.365129
H 12.992053 10.251056 1.732495
H 16.682643 8.428728 -1.289504
H 15.212405 8.092397 -0.381568
O 17.589112 9.955106 3.754726
O 15.316776 10.048489 3.634828
B 16.414543 9.893680 4.447362
H 13.100340 7.586741 6.412846
C 14.530933 7.236085 4.326325
C 14.163357 7.194290 2.985553
C 15.481522 6.348759 4.831558
C 14.746983 6.253479 2.152771
C 16.063815 5.420092 3.987441
C 15.694173 5.371497 2.649969
H 13.435059 7.883962 2.580545
H 15.775178 6.380327 5.874210
H 14.461712 6.214769 1.110952
H 16.803164 4.732653 4.374160
H 16.147391 4.641570 1.993054
C 12.541594 9.443956 4.763039
C 11.444407 8.846766 4.141911
C 12.641132 10.828582 4.849552
C 10.462794 9.643045 3.577130
C 11.647397 11.615605 4.291547
C 10.566390 11.025414 3.652053
H 11.348947 7.768341 4.090162
H 13.482216 11.295163 5.344108
H 9.614957 9.184549 3.087661
H 11.718181 12.692443 4.356480
H 9.795120 11.646084 3.216289

1-c-F

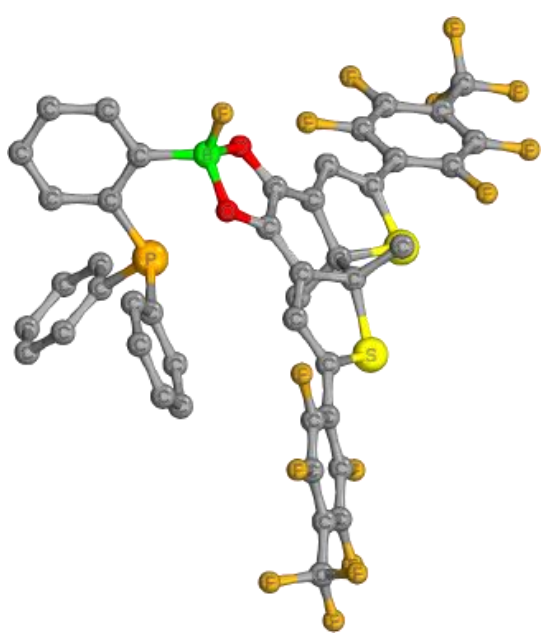

87

xyz, charge: -1, multiplicity: 1
C 12.069335 9.339886 3.780810
C 10.787834 9.228576 4.322418
C 13.086623 8.572284 4.375506
C 12.805917 7.740971 5.458921
C 11.521709 7.649544 5.970157
C 10.506671 8.395613 5.394478
H 9.986067 9.813929 3.891741
H 13.592778 7.149715 5.909068
H 11.319686 6.997819 6.811157
H 9.496525 8.331493 5.780782
P 14.771326 8.617849 3.640520
C 20.844651 14.017921 3.084312
C 20.509981 14.967862 4.039952
C 19.870511 13.332288 2.383360
C 18.502826 13.540506 2.572705
C 18.184799 14.519235 3.523849
C 19.149914 15.195928 4.232158
F 22.110070 13.716422 2.817286
F 20.285390 12.418710 1.509730
F 16.918540 14.845613 3.757900
F 18.757942 16.110838 5.115378
C 21.498560 15.748306 4.854175
C 17.490958 12.802269 1.849948
C 16.245651 12.512277 2.308463
C 15.478559 11.710523 1.435269
C 16.049668 11.620549 0.031527
S 17.821211 12.083548 0.258740
H 15.882536 12.776987 3.290195
C 15.386664 12.693708 -0.836754
H 14.327951 12.471174 -0.977425
H 15.852286 12.769401 -1.817780
H 15.470052 13.659922 -0.340415
C 14.312342 11.077665 1.706631
F 21.308577 15.551525 6.163866
F 21.378488 17.062462 4.634352
F 22.761960 15.424873 4.592673
H 16.524503 9.166174 1.285416
S 16.162885 10.040808 -2.312782
C 14.933480 7.832478 -6.098083
C 13.977358 6.901862 -3.701764
C 15.855238 10.192914 -0.502018
C 15.079416 8.575430 -4.942177
C 16.721728 9.151758 0.212692
C 13.731572 10.125916 0.776056
C 14.397052 9.776673 -0.355517
C 14.291252 6.601166 -6.085308
C 14.609379 8.151503 -3.698067
C 14.080270 5.733984 -7.291253
C 13.968039 8.934844 -1.405870
C 14.785648 8.932746 -2.492891
C 13.821534 6.161528 -4.850728
F 15.416698 8.352803 -7.219982
F 13.217951 4.979380 -4.762057
F 15.681509 9.756332 -5.049854
F 13.527949 6.372835 -2.567842
F 14.676683 4.545703 -7.143734
F 12.779635 5.497170 -7.494883
F 14.556960 6.271194 -8.410746
H 13.035638 8.393236 -1.349913
H 17.783477 9.323423 0.047023
H 16.470211 8.158208 -0.157172
O 13.562230 11.170845 2.774808
O 12.588620 9.692158 1.237020
B 12.306488 10.372121 2.556717
F 11.229263 11.229811 2.398223
C 15.501481 7.051517 4.258167
C 16.261576 6.927347 5.418877
C 15.284681 5.914838 3.480137
C 16.784274 5.697771 5.794125
C 15.794105 4.683494 3.860212
C 16.549204 4.572059 5.019130
H 16.446855 7.796784 6.036613
H 14.710675 6.000295 2.564764
H 17.374179 5.619962 6.698742
H 15.610895 3.812271 3.244456
H 16.957118 3.613381 5.313090

C 15.679764 9.860895 4.630014
C 15.066643 10.722156 5.533725
C 17.038471 10.029443 4.361250
C 15.799240 11.720428 6.161804
C 17.774148 11.013597 5.000766
C 17.153904 11.868045 5.901803
H 14.009460 10.620668 5.740444
H 17.527122 9.391201 3.633998
H 15.305182 12.389363 6.854852
H 18.827345 11.127148 4.777504
H 17.721739 12.648285 6.392769

2-o

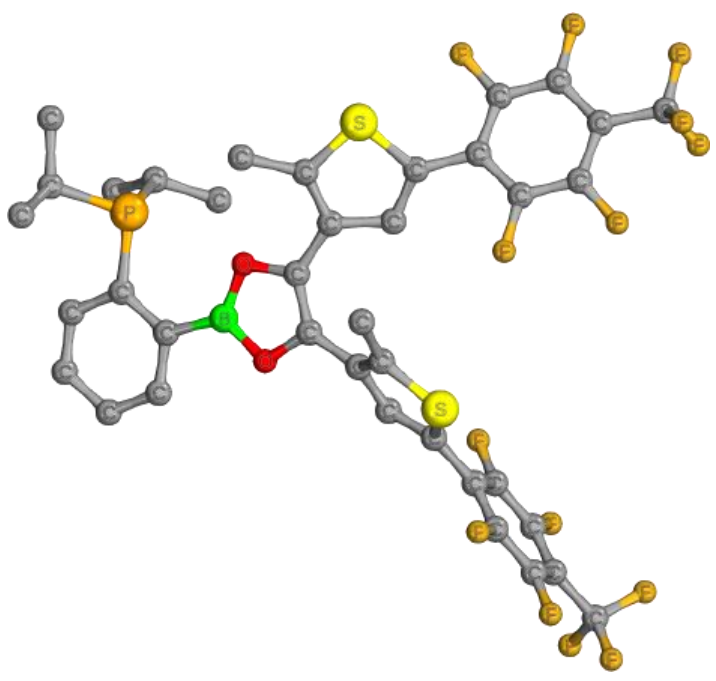

84
xyz, charge: 0, multiplicity: 1
C 13.564827 8.028747 3.742697
C 12.216245 8.046640 3.366091
C 13.896337 8.274737 5.090610
C 12.863412 8.509961 5.997298
C 11.534681 8.514535 5.605557
C 11.206560 8.285330 4.279966
H 11.949079 7.860298 2.333855
H 13.089039 8.697440 7.038170
H 10.759320 8.700653 6.337547
H 10.172681 8.287488 3.961179
P 15.661596 8.274493 5.640460
C 15.547472 7.803609 7.440108
C 16.923235 7.982219 8.078574
H 17.686466 7.414461 7.542531
H 16.912122 7.625797 9.110325
H 17.239265 9.025593 8.101695
C 15.091036 6.351063 7.546187
H 14.840761 8.436529 7.984357
H 15.781395 5.685648 7.024579
H 14.099702 6.196669 7.119904
H 15.053617 6.036248 8.590998
C 16.021629 10.115847 5.708468
C 16.073909 10.681640 4.292189
H 16.780408 10.142685 3.662234
H 16.379221 11.729602 4.315404
H 15.094495 10.641788 3.811444
C 15.107054 10.951016 6.593136
H 17.037001 10.160508 6.117494
H 15.448811 11.988439 6.614864
H 15.085565 10.600370 7.625633
H 14.083386 10.955483 6.217055
C 12.573227 7.217668 -6.254248
C 12.346345 8.575499 -6.428661
C 13.244620 6.748836 -5.138260
C 13.726720 7.593485 -4.146485
C 13.502733 8.954791 -4.338018
C 12.828510 9.429413 -5.442928
F 12.154205 6.319323 -7.131018
F 13.421862 5.437290 -5.024807
F 13.952183 9.836047 -3.452810
F 12.654389 10.738510 -5.557896
C 11.621421 9.173402 -7.607764
C 14.430647 7.090923 -2.972799
C 14.373379 7.565289 -1.695680
C 15.220461 6.862274 -0.798304
C 15.907377 5.841650 -1.408836
S 15.528981 5.762910 -3.074159
H 13.744044 8.384861 -1.383814
C 16.849617 4.857598 -0.808492
H 16.738126 3.872520 -1.260609
H 16.664301 4.761583 0.260241
H 17.888368 5.166673 -0.934853
C 15.328649 7.206121 0.605491
F 12.414854 10.016948 -8.269380
F 10.550810 9.858294 -7.202364
F 11.203074 8.259615 -8.474025
H 17.904873 5.573126 3.470291
S 20.331209 7.082951 1.418504
C 22.714620 8.842191 -2.089726
C 20.282140 7.639819 -2.525757
C 18.760687 6.949578 2.078851
C 21.867471 8.554064 -1.033630
C 18.571877 6.435858 3.466710
C 16.387637 7.368095 1.417824
C 17.812792 7.345319 1.165649
C 22.352887 8.538208 -3.394655
C 20.629324 7.951119 -1.213170
C 23.209177 8.819451 -4.603312
C 18.379548 7.743899 -0.072076
C 19.740210 7.650742 -0.098985
C 21.116652 7.930008 -3.583767
F 23.867873 9.423122 -1.800147
F 20.720052 7.601845 -4.805272
F 22.266370 8.876761 0.191600
F 19.128450 7.033921 -2.785603
F 24.357431 9.409204 -4.296471
F 23.501284 7.687692 -5.246228
F 22.567205 9.616573 -5.458566
H 17.786711 8.112043 -0.896227
H 18.118503 7.183998 4.117905
H 19.517068 6.124072 3.908631
O 14.183802 7.442574 1.315287
O 15.943259 7.663255 2.674076
B 14.574754 7.716992 2.609451

2-o-H

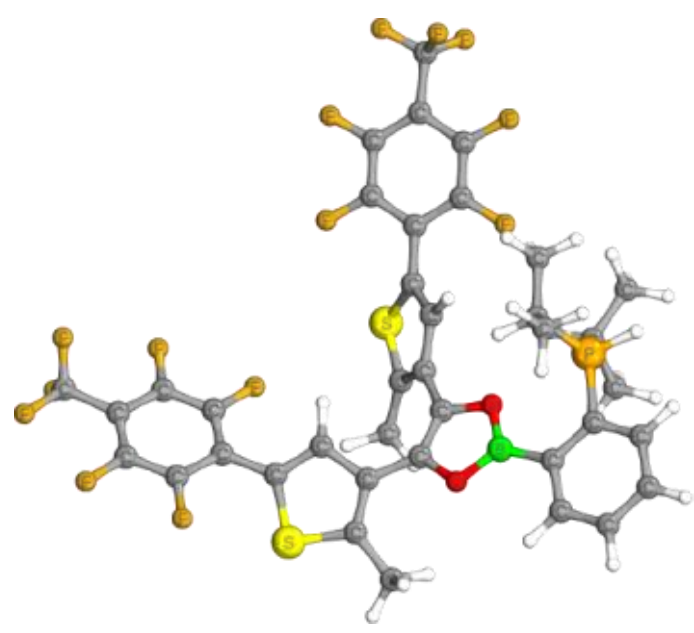

85
xyz, charge: 1, multiplicity: 1
C 13.829935 11.173439 5.085643
C 12.912973 12.048672 5.663360
C 14.366852 10.156458 5.896655
C 13.973502 10.032582 7.226971
C 13.047482 10.910603 7.770180
C 12.521893 11.923497 6.988361
H 12.488932 12.843216 5.063378
H 14.386357 9.256208 7.858485
H 12.747451 10.801634 8.803174
H 11.805833 12.617020 7.407565
P 15.646617 9.003495 5.362616
C 17.215775 9.863534 4.967410
C 18.331261 8.858255 4.690042
H 19.252350 9.403094 4.483358
H 18.131388 8.227052 3.826268
H 18.526605 8.215404 5.549400
C 17.564634 10.803321 6.120742
H 17.028199 10.449892 4.066828
H 18.489841 11.328111 5.883633
H 17.731576 10.259621 7.051712
H 16.798128 11.556414 6.297400
C 15.137598 7.672538 4.197758

C	15.819512	6.363415	4.600772
H	15.466549	6.007456	5.569134
H	16.905346	6.431836	4.637744
H	15.575093	5.599474	3.863411
C	13.618035	7.527819	4.186792
H	15.484145	7.974473	3.208252
H	13.224970	7.268907	5.170610
H	13.347458	6.721505	3.505496
H	13.113022	8.428542	3.845612
C	16.439774	16.183574	-4.204921
C	17.078465	15.215771	-4.967182
C	15.714224	15.831726	-3.078626
C	15.586575	14.515417	-2.659293
C	16.221293	13.552049	-3.437570
C	16.950258	13.893226	-4.556662
F	16.499552	17.467587	-4.507915
F	15.127334	16.797054	-2.381368
F	16.123382	12.264854	-3.115854
F	17.526374	12.922946	-5.248705
C	17.893918	15.508265	-6.205304
C	14.824351	14.149975	-1.470218
C	15.127939	13.191904	-0.550452
C	14.148466	13.066101	0.470003
C	13.101067	13.941432	0.311921
S	13.331477	14.913328	-1.071821
H	16.033448	12.604214	-0.575449
C	11.884833	14.123728	1.157177
H	12.120503	14.655280	2.079402
H	11.446748	13.163579	1.427495
H	11.118711	14.695415	0.636371
C	14.274308	12.094152	1.533346
F	17.949022	16.801005	-6.489362
F	17.374152	14.883871	-7.260887
F	19.144506	15.075780	-6.047549
H	13.782309	11.175223	-1.883607
S	15.891725	8.730008	-1.598940
C	20.120181	6.872920	-1.674704
C	18.564019	6.843507	0.588660
C	14.898633	9.786265	-0.693752
C	19.008683	7.695401	-1.569080
C	13.620935	10.288603	-1.268932
C	14.861958	10.884008	1.553243
C	15.453184	10.039113	0.538879
C	20.475918	6.023801	-0.636229
C	18.206407	7.709354	-0.438438
C	21.673896	5.101859	-0.661750
C	16.670603	9.334826	0.744709
C	17.038979	8.577300	-0.324739
C	19.670673	6.028994	0.497886
F	20.819339	6.943948	-2.791089
F	19.950405	5.234606	1.520828
F	18.716231	8.495988	-2.583596
F	17.819376	6.780513	1.695696
F	22.497968	5.392400	0.345003
F	21.285395	3.835820	-0.521288
F	22.365358	5.190278	-1.786690
H	17.268753	9.425512	1.640249
H	12.926481	10.561316	-0.475906
H	13.140080	9.535870	-1.892321
O	13.803472	12.388126	2.790309
O	14.832888	10.404343	2.851439
B	14.159004	11.339822	3.585152
H	15.938991	8.322867	6.553837

2-o-F

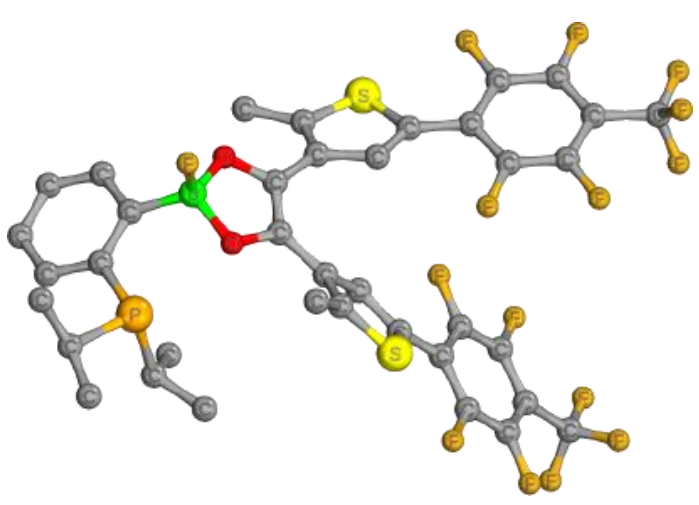

85
xyz, charge: -1, multiplicity: 1

C	13.867959	8.665121	4.745917
C	13.702743	7.275747	4.788479
C	14.410024	9.281542	5.885342
C	14.770859	8.508852	6.992503
C	14.589767	7.137099	7.008559
C	14.044793	6.516518	5.894018
H	13.290180	6.774344	3.921161
H	15.211626	8.978398	7.863927
H	14.875863	6.559800	7.879646
H	13.893426	5.443228	5.886249
P	14.670642	11.110896	5.890507
C	16.557527	11.224123	5.787491
C	16.948203	12.560921	5.159340
H	18.031494	12.705773	5.192950
H	16.484603	13.414527	5.655281
H	16.646331	12.587145	4.111322
C	17.191878	10.086104	4.995144
H	16.946330	11.185992	6.812939
H	16.785151	10.034530	3.985603
H	17.032603	9.116636	5.463357
H	18.270441	10.247173	4.911018
C	14.414897	11.518298	7.703609
C	14.765347	12.982176	7.955867
H	14.199162	13.642305	7.295128
H	15.825291	13.185896	7.799484
H	14.530373	13.262627	8.986148
C	12.959863	11.250060	8.074285
H	15.063661	10.907965	8.341765
H	12.285733	11.834984	7.445290
H	12.766104	11.522660	9.115377
H	12.694213	10.201101	7.946531
C	20.118208	11.236880	-2.545611
C	20.983387	12.322358	-2.539135
C	19.009946	11.192870	-1.719158
C	18.686962	12.217894	-0.828003
C	19.570295	13.301036	-0.837983
C	20.672074	13.353412	-1.660258
F	20.311880	10.200757	-3.350919
F	18.240944	10.113270	-1.813913
F	19.358244	14.334362	-0.026544
F	21.456909	14.424179	-1.593595
C	22.206709	12.448154	-3.400773
C	17.517373	12.169696	0.028321
C	16.816762	11.066798	0.444766
C	15.686348	11.365078	1.242381
C	15.542242	12.719159	1.446783
S	16.784566	13.601527	0.672686
H	17.102576	10.051079	0.224627
C	14.470029	13.425281	2.204414
H	14.539137	14.506300	2.085671
H	14.511809	13.184908	3.267290
H	13.488250	13.104398	1.856003
C	14.800450	10.368290	1.813141
F	23.309333	12.572432	-2.654044
F	22.136102	13.530763	-4.181702
F	22.392723	11.401979	-4.198946
H	15.209864	6.369890	1.634702
S	14.989645	6.940626	-1.891382
C	15.295847	8.683719	-6.074428
C	15.625351	10.390491	-3.954463
C	14.711318	7.265573	-0.235987
C	15.060533	8.261953	-4.779881
C	14.625822	6.145058	0.743348
C	14.350201	9.200819	1.307281
C	14.594195	8.615533	0.001945
C	15.701230	9.983303	-6.342770
C	15.211982	9.085495	-3.664054
C	15.976158	10.528630	-7.714623
C	14.732693	9.372187	-1.187509
C	14.975909	8.622353	-2.309639
C	15.857909	10.818833	-5.242563
F	15.109393	7.796708	-7.043984
F	16.252910	12.074365	-5.422934
F	14.670082	6.998901	-4.618105
F	15.817512	11.274354	-2.982360

F	15.787858	9.631376	-8.678119
F	15.180905	11.568193	-7.986780
F	17.237171	10.957525	-7.819233
H	14.618876	10.443544	-1.190921
H	14.992841	5.210819	0.318827
H	13.597384	5.992430	1.070009
O	14.331192	10.572751	3.052593
O	13.579759	8.550552	2.191022
B	13.426154	9.441205	3.387388
F	12.100414	9.882911	3.482549

2-c

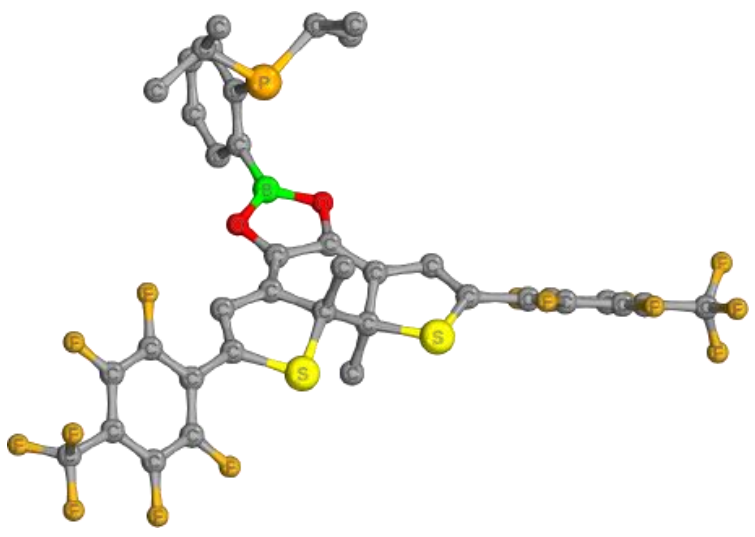

84
xyz, charge: 0, multiplicity: 1

C	15.752905	5.976840	3.946214
C	15.075280	4.801660	4.266139
C	16.579438	6.576770	4.898997
C	16.719248	5.997203	6.156603
C	16.040762	4.830259	6.466373
C	15.218378	4.231736	5.520087
H	14.432423	4.325500	3.535947
H	17.356354	6.458120	6.902404
H	16.150918	4.384126	7.446240
H	14.689761	3.319405	5.763023
P	17.417061	8.108859	4.337119
C	19.184810	7.694234	4.767042
C	20.107954	8.898212	4.617263
H	20.031032	9.342095	3.622879
H	21.146592	8.594105	4.760408
H	19.898695	9.675358	5.351489
C	19.639044	6.549114	3.865544
H	19.220090	7.358630	5.809334
H	19.601269	6.838297	2.813856
H	19.021690	5.659101	3.986371
H	20.668815	6.270021	4.096681
C	16.892702	9.370070	5.613387
C	17.186795	10.774188	5.085030
H	16.910765	11.524848	5.828154
H	16.607771	10.972949	4.181877
H	18.235780	10.930308	4.838674
C	15.400211	9.239009	5.907552
H	17.457291	9.204164	6.538043
H	15.151162	8.303421	6.404688
H	14.810804	9.290964	4.989512
H	15.073000	10.056138	6.553513
C	9.508426	12.144105	-0.112495
C	9.689618	13.413025	-0.651887
C	10.525693	11.213882	-0.089485
C	11.789068	11.501561	-0.601481
C	11.958943	12.768726	-1.144125
C	10.941490	13.707123	-1.173487
F	8.330039	11.799396	0.387469
F	10.266694	10.016499	0.420118
F	13.138097	13.112918	-1.645385
F	11.218892	14.885810	-1.706776
C	8.526990	14.373606	-0.636478
C	12.866961	10.517315	-0.587041
C	13.140588	9.657837	0.419087
C	14.236152	8.792237	0.140075
C	14.607398	8.774168	-1.337793
S	13.941655	10.367292	-1.975901
H	12.619945	9.651365	1.365212
C	13.829129	7.635869	-2.009786
H	12.768385	7.740377	-1.785800
H	14.161993	6.665479	-1.639573
H	13.951230	7.645986	-3.090540
C	14.920635	7.957842	0.938199
F	8.824302	15.552618	-1.166987
F	8.115284	14.590924	0.613581
F	7.497070	13.875437	-1.321666
H	16.678275	10.767696	-1.568561
S	16.665325	8.324388	-3.216205
C	19.600212	5.965547	-5.788593
C	20.206870	6.480044	-3.155829
C	16.129662	8.653738	-1.486591
C	18.625591	6.407645	-4.910306
C	16.899222	9.880543	-0.979367
C	16.059328	7.214980	0.479398
C	16.629347	7.438565	-0.714896
C	20.904825	5.766927	-5.359386
C	18.891915	6.673735	-3.573459
C	22.027348	5.283922	-6.243295
C	17.711405	6.783684	-1.368420
C	17.857430	7.150836	-2.660749
C	21.182157	6.037205	-4.023725
F	19.231130	5.735259	-7.038126
F	22.413615	5.880647	-3.558424
F	17.393375	6.565975	-5.375718
F	20.556457	6.739878	-1.902423
F	22.544036	4.150069	-5.767173
F	21.638858	5.050342	-7.490022
F	23.007670	6.186748	-6.292777
H	18.317100	6.033646	-0.881771
H	17.969330	9.687611	-1.041471
H	16.652599	10.091092	0.061981
O	14.690446	7.621674	2.231393
O	16.431119	6.359957	1.463716
B	15.645777	6.673639	2.559885

2-c-H

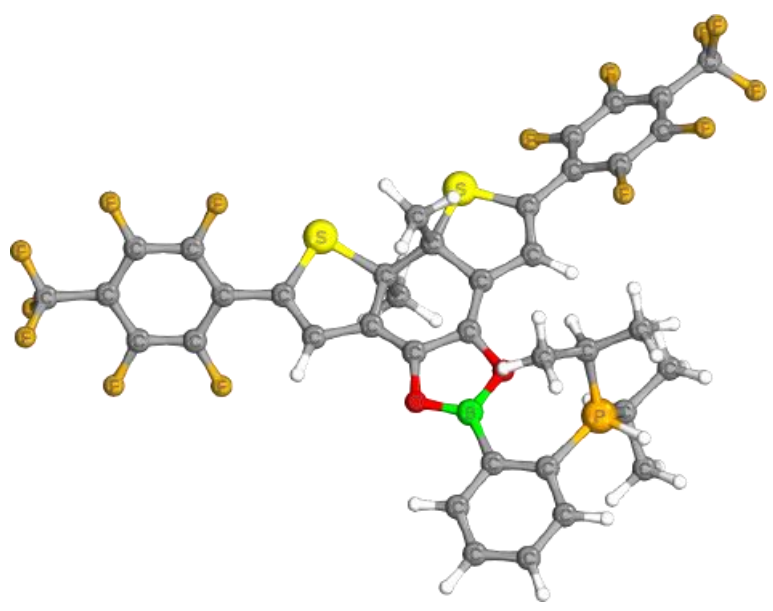

85
xyz, charge: 1, multiplicity: 1

C	16.186042	8.362104	5.281490
C	16.970845	8.733710	6.370142
C	15.397414	7.202116	5.400853
C	15.412339	6.460535	6.579298
C	16.197097	6.860786	7.651391
C	16.976924	7.997931	7.546516
H	17.590377	9.618119	6.299670
H	14.815327	5.563051	6.679565
H	16.196793	6.277093	8.561547
H	17.593635	8.312656	8.377139
P	14.320182	6.557607	4.103734
C	15.155166	6.002074	2.558379
C	14.482557	4.720454	2.059816
H	14.631945	3.892345	2.753269
H	14.932719	4.434212	1.109681
H	13.413737	4.829585	1.884851
C	16.646393	5.778761	2.798528
H	15.016839	6.795250	1.822191
H	17.175857	6.687738	3.075475
H	17.098210	5.407080	1.879196
H	16.828311	5.035181	3.575124
C	12.789914	7.545332	3.894258
C	11.890871	6.948076	2.814469
H	11.564117	5.937330	3.061940
H	12.362418	6.927299	1.832103
H	10.994364	7.561226	2.725250

C	12.084504	7.636027	5.246784
H	13.109972	8.540500	3.586052
H	11.195012	8.256933	5.142564
H	12.707781	8.090323	6.016117
H	11.755890	6.658273	5.601935
C	21.795855	15.457593	0.007567
C	22.022778	16.263105	1.114299
C	20.773028	14.523658	0.012544
C	19.937546	14.344055	1.106239
C	20.168872	15.162499	2.208678
C	21.184571	16.095245	2.211161
F	22.536293	15.536783	-1.082754
F	20.603977	13.772378	-1.067280
F	19.395387	15.070339	3.282872
F	21.344888	16.842976	3.290923
C	23.116058	17.304355	1.198693
C	18.856616	13.360566	1.095354
C	18.486183	12.580803	2.136839
C	17.381307	11.735504	1.838397
C	16.650456	12.155026	0.565767
S	17.900719	13.133414	-0.359793
H	19.004481	12.557479	3.083489
C	15.515353	13.106383	0.970730
H	15.029543	13.545372	0.102451
H	15.917116	13.915943	1.578108
H	14.756877	12.585655	1.556581
C	16.847316	10.701545	2.505949
F	23.931979	17.033444	2.216354
F	22.590421	18.510586	1.401910
F	23.851512	17.365003	0.098774
H	18.002875	9.755906	0.039407
S	15.038224	11.307944	-1.594256
C	11.307047	10.010325	-3.840002
C	12.534441	8.199996	-2.175959
C	16.171248	10.913051	-0.200111
C	12.291206	10.416443	-2.952425
C	17.314131	10.050442	-0.753427
C	15.773425	9.921183	1.974837
C	15.326708	10.039450	0.717423
C	10.916219	8.681409	-3.905679
C	12.932819	9.533804	-2.092386
C	9.848275	8.150911	-4.835330
C	14.231838	9.431913	0.045766
C	13.974961	9.973496	-1.168557
C	11.556411	7.787892	-3.053882
F	10.762514	10.939692	-4.601567
F	11.236153	6.502995	-3.073319
F	12.611704	11.702165	-2.927937
F	13.108695	7.275258	-1.407961
F	9.310853	9.096121	-5.590041
F	10.358810	7.225025	-5.644148
F	8.866993	7.586827	-4.131381
H	13.625881	8.659909	0.494388
H	17.884306	10.578076	-1.514375
H	16.904582	9.148058	-1.205427
O	17.148531	10.217098	3.754552
O	15.368527	9.046674	2.955373
B	16.241806	9.228861	4.005074
H	13.861988	5.362893	4.678620

2-c-F

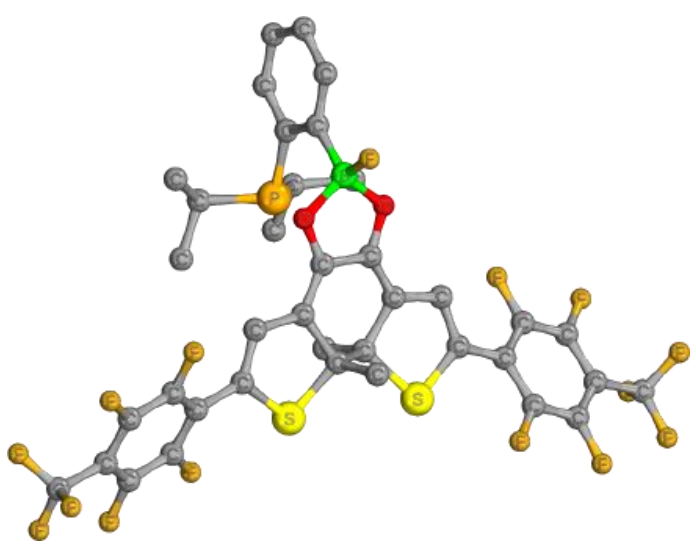

85
xyz, charge: -1, multiplicity: 1

C	14.336177	6.880573	4.145468
C	13.614382	5.967063	4.920021
C	15.720994	6.682215	4.040858
C	16.330639	5.615366	4.702602
C	15.593097	4.727177	5.465546
C	14.221853	4.905608	5.570340
H	12.544355	6.094249	5.018040
H	17.404141	5.477714	4.632421
H	16.083738	3.906150	5.974065
H	13.627158	4.219735	6.161768
P	16.735535	7.851606	3.041771
C	17.690297	6.627970	1.997195
C	18.873608	7.277551	1.289137
H	18.563631	8.151174	0.712165
H	19.332232	6.571449	0.592445
H	19.650170	7.593346	1.986126
C	16.717590	6.036248	0.979926
H	18.072594	5.819667	2.629626
H	17.215939	5.285439	0.361699
H	16.329801	6.811279	0.315628
H	15.863336	5.560671	1.461595
C	17.993289	8.449476	4.300915
C	18.666153	9.712252	3.761343
H	19.151643	9.561518	2.797831
H	19.426020	10.073171	4.459314
H	17.931270	10.508905	3.629025
C	17.312244	8.772274	5.628843
H	18.753258	7.676125	4.467824
H	18.017984	9.259909	6.306330
H	16.928371	7.883549	6.125853
H	16.469480	9.450498	5.482000
C	17.488734	16.587945	3.830571
C	16.904952	16.948433	5.037928
C	17.070456	15.466223	3.140203
C	16.046288	14.631693	3.590381
C	15.459901	15.018183	4.801725
C	15.877790	16.128987	5.497687
F	18.480153	17.293558	3.299601
F	17.702643	15.179018	2.006261
F	14.444311	14.325728	5.307710
F	15.258353	16.429972	6.635691
C	17.301570	18.141346	5.857116
C	15.607220	13.469141	2.847723
C	15.073184	12.333332	3.370165
C	14.714101	11.361167	2.409029
C	14.664587	11.917536	0.994053
S	15.746199	13.408469	1.078503
H	14.974056	12.143223	4.428505
C	13.236741	12.387254	0.700586
H	12.552538	11.538314	0.670001
H	13.169373	12.914218	-0.249563
H	12.909654	13.060727	1.492133
C	14.339078	10.073106	2.614551
F	18.303297	18.833096	5.321400
F	17.690447	17.777376	7.083981
F	16.277332	18.988843	6.005509
H	16.937011	9.736190	-0.508064
S	14.841916	11.263908	-1.754579
C	13.891229	9.486773	-5.839020
C	14.603983	7.770487	-3.820927
C	15.174268	10.853630	0.012320
C	13.968462	9.926556	-4.531334
C	16.670103	10.560131	0.154237
C	14.109131	9.176576	1.493055
C	14.403754	9.559699	0.224329
C	14.160429	8.165047	-6.169926
C	14.321997	9.095615	-3.467377
C	14.091608	7.600252	-7.558248
C	14.201208	8.845539	-0.978110
C	14.417120	9.574672	-2.105269
C	14.520592	7.324845	-5.119883
F	13.533406	10.374827	-6.758959
F	14.815673	6.052153	-5.370289
F	13.667519	11.200654	-4.297643
F	14.996347	6.897725	-2.898043
F	13.217932	6.589710	-7.626243
F	13.720716	8.496064	-8.468770
F	15.278413	7.119202	-7.944896

H 13.853184 7.823205 -0.976571
H 16.902861 10.258638 1.176152
H 17.280535 11.422766 -0.106307
O 14.120698 9.446199 3.739341
O 13.651878 8.032340 1.922311
B 13.534895 8.088192 3.427007
F 12.192479 8.089765 3.776201

TMS

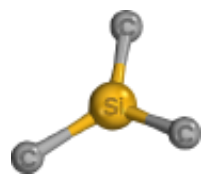

13
xyz, charge: 1, multiplicity: 1
C -2.991743 0.724986 -0.626092
Si -1.284744 0.144071 -0.301590
H -3.116623 1.797099 -0.484882
H -3.701781 0.204147 0.024400
H -3.293288 0.471642 -1.647538
C 0.007757 1.338157 0.208534
H 0.118888 2.124708 -0.544809
H 0.980224 0.877315 0.372523
H -0.291036 1.854712 1.126356
C -0.870203 -1.630753 -0.488037
H -1.717608 -2.241726 -0.793929
H -0.474508 -2.030828 0.451030
H -0.066825 -1.759720 -1.220507

TMSF

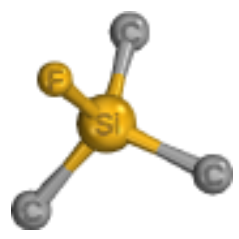

14
xyz, charge: 0, multiplicity: 1
C -2.783810 0.809545 -1.162693
Si -1.494014 0.399954 0.131390
H -3.048038 1.867701 -1.153411
H -3.702554 0.240352 -1.016306
H -2.417157 0.577800 -2.164615
C 0.048334 1.434245 -0.105577
H -0.171131 2.502409 -0.083471
H 0.516535 1.221071 -1.068485
H 0.793084 1.236128 0.666152
C -1.089830 -1.427435 0.125456
H -1.977454 -2.039575 0.289859
H -0.365698 -1.685223 0.899206
H -0.661502 -1.730121 -0.831932
F -2.116715 0.761907 1.592945

1-o-PhCHO

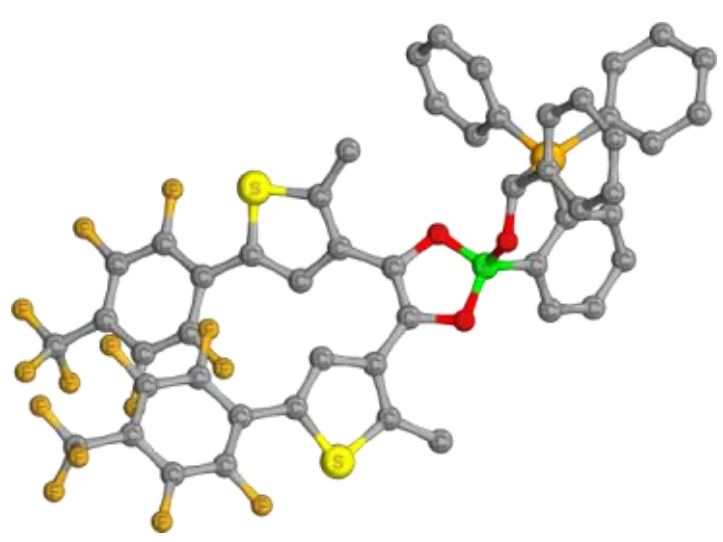

100
xyz, charge: 0, multiplicity: 1
C 17.870731 8.469937 5.382132
C 17.416551 7.339623 6.060732
C 17.972440 9.645598 6.132891
C 17.649828 9.711712 7.489611
C 17.210666 8.568584 8.128008
C 17.094150 7.384313 7.406851
H 17.309667 6.410884 5.515240
H 17.734051 10.642277 8.037459
H 16.956243 8.597626 9.178902
H 16.743472 6.489426 7.905557
P 18.575885 11.086860 5.276967
C 26.438636 4.073711 1.285198
C 25.949185 2.830027 0.913533
C 25.579702 5.120327 1.566736
C 24.194339 5.007005 1.494804
C 23.718153 3.750694 1.114853
C 24.567210 2.701434 0.836771
F 27.737457 4.310669 1.395013
F 26.125363 6.281445 1.921705
F 22.414037 3.526803 1.003086
F 24.031486 1.541955 0.478398
C 26.806775 1.634909 0.592996
C 23.303698 6.121448 1.778872
C 22.004778 6.076190 2.203962
C 21.396549 7.350857 2.331241
C 22.258433 8.368463 2.006067
S 23.794745 7.766438 1.562533
H 21.479964 5.166313 2.447177
C 21.984998 9.832922 1.994377
H 21.914884 10.226495 3.007742
H 21.036745 10.036731 1.496102
H 22.763540 10.386831 1.471255
C 20.018548 7.539327 2.748812
F 26.590515 1.216620 -0.655270
F 26.526214 0.618512 1.411907
F 28.105831 1.885873 0.706530
H 16.619156 3.343239 2.748360
S 18.108652 3.521603 0.391087
C 19.912950 2.750351 -3.706083
C 21.202688 4.478164 -2.033250
C 17.968365 4.630572 1.683169
C 19.313477 3.167796 -2.538943
C 17.037009 4.347021 2.813015
C 18.925056 6.822945 2.431588
C 18.797603 5.711525 1.506335
C 21.178827 3.189029 -4.075750
C 19.932320 4.050012 -1.652807
C 21.767808 2.693414 -5.369291
C 19.543925 5.618346 0.304319
C 19.304557 4.478319 -0.412609
C 21.810964 4.066014 -3.206120
F 19.246445 1.913022 -4.490000
F 23.023443 4.539579 -3.453733
F 18.098766 2.701503 -2.263170
F 21.893072 5.311752 -1.261778
F 20.991605 3.029971 -6.402322
F 22.977787 3.184591 -5.609108
F 21.872815 1.363001 -5.363597
H 20.212756 6.400744 -0.015356
H 16.212417 5.058497 2.828776
H 17.557023 4.429747 3.767657
O 19.713042 8.533495 3.620962
O 17.834933 7.263301 3.105059
B 18.248710 8.464061 3.806614
O 17.585561 9.651822 3.198766
C 18.100825 10.880546 3.405637
C 17.147005 11.975208 3.013318
C 17.602644 13.280786 2.869981
C 16.725708 14.306531 2.563335
C 15.377189 14.032417 2.384439
C 14.921188 12.728627 2.502621
C 15.799796 11.701176 2.815425
H 15.443992 10.683601 2.898708
H 18.656843 13.503820 2.994510
H 17.094187 15.318162 2.457971
H 14.688398 14.830589 2.141500
H 13.874024 12.505950 2.344624
H 19.076608 11.045932 2.920395
C 17.858508 12.549227 6.045459
C 18.628188 13.531696 6.659236
C 16.468340 12.666386 6.030122
C 18.012433 14.635793 7.229966
C 15.861556 13.771887 6.596606

C	16.633235	14.760716	7.191588
H	19.704516	13.440181	6.707395
H	15.856922	11.896748	5.578441
H	18.615032	15.395957	7.708386
H	14.784330	13.861240	6.572213
H	16.156476	15.625906	7.632738
C	20.369493	11.197381	5.380432
C	21.102691	10.169309	5.960834
C	21.029199	12.285680	4.809114
C	22.486972	10.242323	5.986967
C	22.411034	12.357552	4.847190
C	23.140313	11.334138	5.437368
H	20.599527	9.307754	6.377223
H	20.470532	13.084665	4.336035
H	23.055210	9.435807	6.429349
H	22.918128	13.205202	4.406621
H	24.220721	11.383996	5.458435

1-c-PhCHO

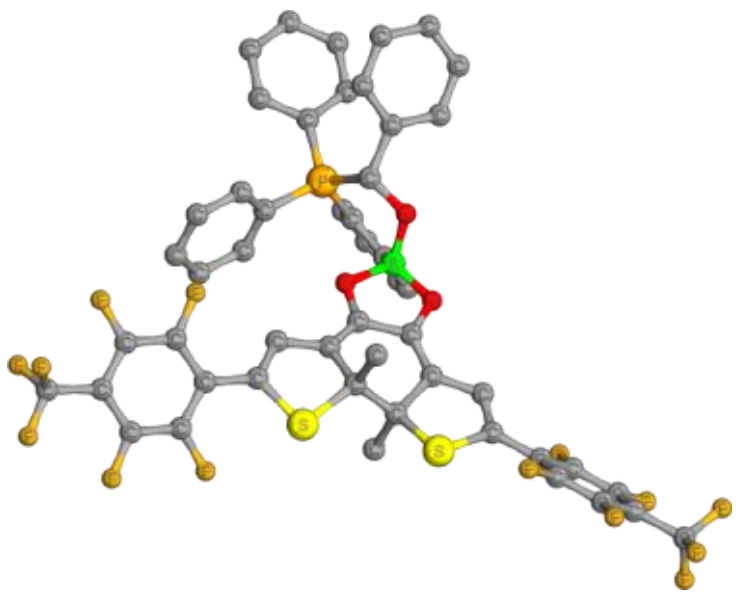

100
xyz, charge: 0, multiplicity: 1

C	-4.479258	-1.511499	-0.195866
C	-5.162665	-2.688575	-0.489608
C	-4.135318	-1.301239	1.145214
C	-4.439501	-2.213695	2.155370
C	-5.111465	-3.375485	1.824579
C	-5.472355	-3.606596	0.501751
H	-5.458426	-2.885216	-1.511959
H	-4.160605	-2.020194	3.183821
H	-5.355514	-4.097261	2.592244
H	-6.003317	-4.514459	0.245118
P	-3.240161	0.200934	1.491041
C	4.352854	0.889137	-2.319627
C	5.294213	0.157665	-3.036351
C	3.004815	0.795854	-2.581692
C	2.498614	-0.053951	-3.567695
C	3.450828	-0.771961	-4.285152
C	4.809027	-0.673641	-4.036291
F	4.749702	1.717263	-1.358510
F	2.185300	1.565467	-1.869098
F	3.059552	-1.607831	-5.238903
F	5.616211	-1.416367	-4.777809
C	6.749480	0.312961	-2.685208
C	1.073701	-0.171821	-3.830837
C	0.091403	-0.088635	-2.902902
C	-1.215108	-0.234291	-3.436569
C	-1.258842	-0.085962	-4.948244
S	0.468760	-0.472416	-5.468035
H	0.266995	0.015135	-1.841961
C	-1.535761	1.382312	-5.287084
H	-2.538677	1.669294	-4.968249
H	-1.450135	1.575941	-6.354371
H	-0.817278	2.014513	-4.766558
C	-2.381808	-0.400364	-2.781986
F	6.964673	-0.021967	-1.409353
F	7.143176	1.578974	-2.837595
F	7.546411	-0.442069	-3.430467
H	-2.735704	-3.157892	-5.713475
S	-2.687778	-0.746448	-7.293498
C	-7.262111	-2.247619	-9.125797
C	-5.171060	-0.506075	-9.323808
C	-2.302027	-1.056248	-5.519305
C	-6.403475	-2.074591	-8.062290
C	-1.911062	-2.531270	-5.375725
C	-3.626069	-0.624810	-3.486163
C	-3.638660	-0.844435	-4.816686
C	-7.099466	-1.554868	-10.320860
C	-5.327719	-1.188486	-8.122362
C	-8.082367	-1.799424	-11.435208
C	-4.745323	-1.019031	-5.693221
C	-4.412571	-1.007297	-7.003956
C	-6.025845	-0.678846	-10.398372
F	-8.259473	-3.113664	-8.994824
F	-5.781708	0.032657	-11.488478
F	-6.615659	-2.799427	-6.969636
F	-4.175378	0.361369	-9.459432
F	-7.831071	-1.071982	-12.516820
F	-8.068111	-3.081381	-11.806781
F	-9.324769	-1.509243	-11.041958
H	-5.759488	-1.098565	-5.330548
H	-1.700659	-2.775098	-4.333529
H	-1.031194	-2.776876	-5.966808
O	-2.608984	-0.378404	-1.477882
O	-4.638121	-0.621069	-2.624495
B	-4.092342	-0.388764	-1.287830
O	-4.539149	0.915093	-0.787815
C	-3.865791	1.516770	0.225793
C	-4.716001	2.518600	0.955384
C	-6.101774	2.455926	0.887280
C	-6.872362	3.350282	1.616505
C	-6.268771	4.309334	2.415159
C	-4.884236	4.384192	2.472328
C	-4.115506	3.495247	1.741782
H	-3.033948	3.564586	1.789707
H	-6.574966	1.714819	0.258020
H	-7.951491	3.299965	1.554013
H	-6.873622	5.004620	2.981954
H	-4.404207	5.137990	3.081744
H	-2.928512	1.993349	-0.101734
C	-1.469190	-0.025163	1.266376
C	-0.634383	1.089284	1.220734
C	-0.937964	-1.298991	1.108336
C	0.726905	0.928324	1.026510
C	0.425869	-1.454989	0.916733
C	1.256860	-0.345661	0.875541
H	-1.037582	2.089608	1.328322
H	-1.585869	-2.165346	1.112451
H	1.371796	1.794828	0.983016
H	0.837236	-2.446257	0.784388
H	2.319796	-0.471147	0.717554
C	-3.535262	0.702169	3.192698
C	-4.856072	0.824628	3.625616
C	-2.490064	1.002543	4.059802
C	-5.122735	1.263435	4.909049
C	-2.765578	1.439525	5.346741
C	-4.078195	1.576218	5.768611
H	-5.676781	0.583749	2.963171
H	-1.461766	0.892481	3.743117
H	-6.147992	1.365092	5.236952
H	-1.950549	1.670189	6.019181
H	-4.289625	1.922375	6.771563

1-o-PhNCS

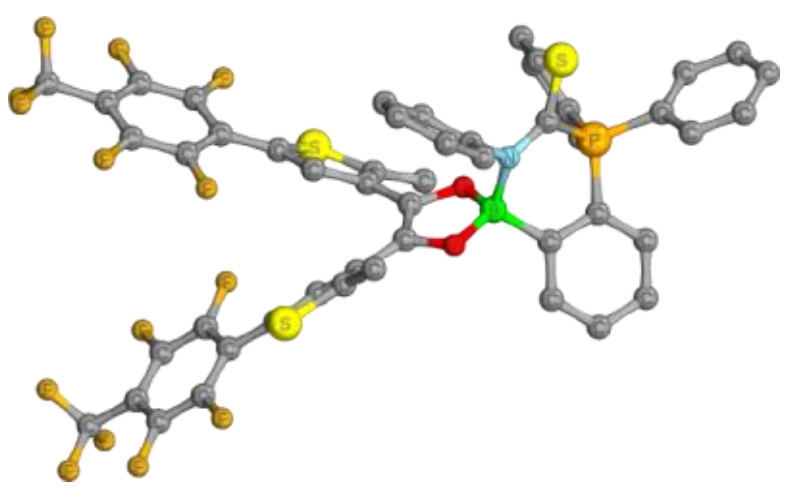

100
xyz, charge: 0, multiplicity: 1

C	-4.610905	-2.365847	-1.090036
C	-5.272960	-3.185233	-2.003542
C	-4.440516	-2.878302	0.197848

C	-4.895600	-4.140334	0.580408
C	-5.538504	-4.929325	-0.355252
C	-5.726368	-4.445162	-1.644828
H	-5.439351	-2.823326	-3.009640
H	-4.762628	-4.497117	1.594576
H	-5.899792	-5.911158	-0.080452
H	-6.238769	-5.058099	-2.375430
P	-3.620298	-1.788247	1.342484
C	3.240380	3.521060	-5.869868
C	2.523336	4.363945	-6.710807
C	2.626644	2.513924	-5.158910
C	1.254834	2.269355	-5.235162
C	0.551086	3.110578	-6.095942
C	1.159098	4.130445	-6.807955
F	4.549444	3.681627	-5.727972
F	3.387703	1.757009	-4.372312
F	-0.757768	2.960333	-6.266667
F	0.381536	4.866051	-7.588108
C	3.258556	5.455518	-7.442706
C	0.606362	1.210143	-4.476796
C	-0.714133	1.109572	-4.131672
C	-1.035075	-0.075262	-3.420562
C	0.065573	-0.869442	-3.212420
S	1.473429	-0.167143	-3.881457
H	-1.456753	1.860386	-4.347899
C	0.134501	-2.179703	-2.505985
H	0.047993	-2.046815	-1.427893
H	1.070046	-2.699637	-2.708730
H	-0.685381	-2.826363	-2.816584
C	-2.383332	-0.379370	-2.977087
F	4.199710	4.943134	-8.237659
F	3.862920	6.276137	-6.579918
F	2.462239	6.199136	-8.200865
H	-5.480407	2.260660	-3.585994
S	-4.788323	1.510679	-6.986377
C	-2.802571	0.342329	-10.944167
C	-1.325604	0.276570	-8.630435
C	-4.838362	1.179448	-5.308654
C	-3.412830	0.472841	-9.708964
C	-5.909698	1.772100	-4.459296
C	-3.554789	-0.121430	-3.584770
C	-3.801357	0.359831	-4.931563
C	-1.429791	0.165587	-11.046676
C	-2.706595	0.439523	-8.510691
C	-0.684183	-0.003919	-12.344359
C	-2.969485	0.012293	-6.028748
C	-3.356773	0.568650	-7.215077
C	-0.711412	0.138953	-9.856490
F	-3.583527	0.381234	-12.013237
F	0.606335	-0.014319	-9.883424
F	-4.733354	0.628710	-9.687197
F	-0.553735	0.252326	-7.549766
F	-0.051407	-1.179357	-12.373978
F	-1.477610	0.043909	-13.407706
F	0.236119	0.950537	-12.492388
H	-2.129375	-0.656563	-5.925862
H	-6.593578	1.005530	-4.096647
H	-6.491392	2.514301	-5.004399
O	-2.579090	-0.955440	-1.761325
O	-4.601151	-0.448053	-2.779474
B	-4.031200	-0.926142	-1.537905
S	-3.965941	0.994169	2.175839
N	-4.304298	0.152886	-0.365963
C	-4.581565	1.497183	-0.752799
C	-3.552112	2.317670	-1.189860
C	-3.830442	3.621994	-1.568815
C	-5.127784	4.108950	-1.500910
C	-6.151278	3.283560	-1.058138
C	-5.880216	1.975859	-0.687091
H	-6.672800	1.324476	-0.343754
H	-5.340613	5.128667	-1.792937
H	-7.165379	3.656749	-1.004340
H	-2.539312	1.938914	-1.225044
H	-3.027794	4.261126	-1.912140
C	-4.036149	-0.056784	0.903730
C	-1.836414	-2.016360	1.287046
C	-0.967711	-0.968809	1.576685
C	-1.340807	-3.286068	1.002279
C	0.398645	-1.200233	1.585386
C	0.025930	-3.510384	1.028192
C	0.894676	-2.468555	1.320259
H	-1.353947	0.019048	1.792353
H	-2.013427	-4.095238	0.747532
H	1.076512	-0.385300	1.799432
H	0.411974	-4.496045	0.806451
H	1.962316	-2.643384	1.330982
C	-4.200851	-2.135023	3.002696
C	-3.314902	-2.451349	4.024336
C	-5.573278	-2.102756	3.245532
C	-3.802646	-2.735378	5.291537
C	-6.051464	-2.384265	4.511591
C	-5.165983	-2.700578	5.534695
H	-2.248969	-2.472650	3.840900
H	-6.265655	-1.853457	2.451117
H	-3.113572	-2.978952	6.088606
H	-7.115286	-2.353468	4.703276
H	-5.543255	-2.919466	6.524728

1-c-PhNCS

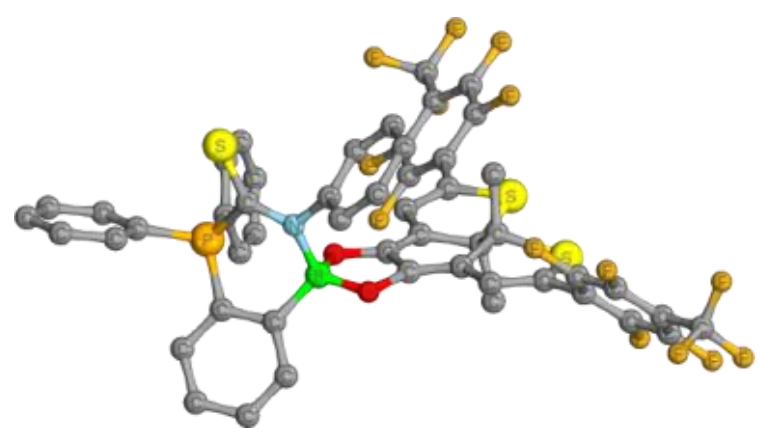

100
xyz, charge: 0, multiplicity: 1

C	-0.089672	-0.582681	1.144875
C	1.224936	-1.036764	1.087967
C	-0.821150	-0.900225	2.292654
C	-0.287145	-1.638924	3.346514
C	1.020095	-2.082339	3.254536
C	1.771774	-1.775820	2.126744
H	1.827612	-0.807410	0.218915
H	-0.876068	-1.857037	4.228756
H	1.454254	-2.657638	4.060950
H	2.797210	-2.116035	2.060286
P	-2.480685	-0.247044	2.352451
C	3.164941	4.603897	-6.389961
C	3.885292	4.094333	-7.465504
C	2.270836	3.828279	-5.682847
C	2.042525	2.494139	-6.013400
C	2.758728	1.996046	-7.094089
C	3.659429	2.767685	-7.807424
F	3.319949	5.872322	-6.030984
F	1.605774	4.393787	-4.682595
F	2.593735	0.730926	-7.458989
F	4.295089	2.185643	-8.812626
C	4.842457	5.006230	-8.188293
C	1.083041	1.673398	-5.283629
C	0.925419	1.639147	-3.943453
C	-0.125366	0.775583	-3.515472
C	-1.074998	0.420177	-4.657962
S	-0.027900	0.618543	-6.164107
H	1.548727	2.183195	-3.248581
C	-2.173517	1.486987	-4.713811
H	-2.794405	1.380208	-5.600598
H	-1.719716	2.477231	-4.724821
H	-2.815471	1.421585	-3.834840
C	-0.432767	0.342230	-2.280359
F	5.517676	4.389446	-9.150183
F	5.737428	5.518641	-7.341251
F	4.190170	6.025769	-8.750490
H	0.294966	-1.921788	-4.007831
S	-3.044618	-1.399338	-5.580635
C	-6.638862	-4.748118	-3.933041
C	-6.089133	-2.539836	-5.440515
C	-1.617812	-1.005293	-4.472080
C	-5.461593	-4.058665	-3.746521
C	-0.559696	-2.095780	-4.662983

C	-1.550494	-0.546941	-2.056880
C	-2.207786	-1.132511	-3.075655
C	-7.579860	-4.352448	-4.878204
C	-5.151654	-2.914803	-4.484029
C	-8.847648	-5.154455	-5.012027
C	-3.383587	-1.927661	-3.034709
C	-3.930851	-2.157330	-4.249463
C	-7.272404	-3.231934	-5.637976
F	-6.861994	-5.824246	-3.186251
F	-8.099590	-2.768748	-6.562072
F	-4.608879	-4.526070	-2.838242
F	-5.871057	-1.461799	-6.182790
F	-9.683897	-4.657476	-5.914372
F	-8.577469	-6.412063	-5.366933
F	-9.502507	-5.198374	-3.848310
H	-3.824469	-2.248643	-2.101777
H	-0.197791	-2.136660	-5.688479
H	-0.990215	-3.064999	-4.414387
O	0.128152	0.649628	-1.105518
O	-1.766496	-0.667440	-0.752952
B	-0.785712	0.209757	-0.062104
S	-3.591229	2.457576	2.021712
N	-1.608067	1.461972	0.487744
C	-1.600830	2.637883	-0.320196
C	-0.504047	3.484217	-0.279975
C	-0.453864	4.582703	-1.123279
C	-1.495935	4.834612	-2.003330
C	-2.593503	3.987595	-2.033139
C	-2.648851	2.886850	-1.193425
H	-3.499541	2.218491	-1.218121
H	-1.451183	5.688425	-2.665944
H	-3.407783	4.177089	-2.719701
H	0.309780	3.275387	0.401338
H	0.404908	5.240056	-1.096910
C	-2.477279	1.373070	1.475011
C	-3.657119	-1.339481	1.544107
C	-3.260608	-2.614637	1.155470
C	-4.951935	-0.891504	1.290210
C	-4.165448	-3.446167	0.515467
C	-5.847384	-1.729630	0.646412
C	-5.455430	-3.004139	0.259279
H	-2.247095	-2.951400	1.327930
H	-5.252389	0.108122	1.580091
H	-3.857942	-4.434525	0.202963
H	-6.850765	-1.382979	0.439896
H	-6.155612	-3.654399	-0.248552
C	-2.955925	-0.012788	4.063118
C	-3.944853	-0.802364	4.636750
C	-2.261267	0.918153	4.834956
C	-4.246457	-0.653738	5.983356
C	-2.567336	1.059067	6.175010
C	-3.561107	0.274569	6.748872
H	-4.484613	-1.527784	4.042599
H	-1.493043	1.537186	4.390010
H	-5.019863	-1.263953	6.429329
H	-2.035485	1.786012	6.773275
H	-3.800076	0.390979	7.797540

1-o-PO

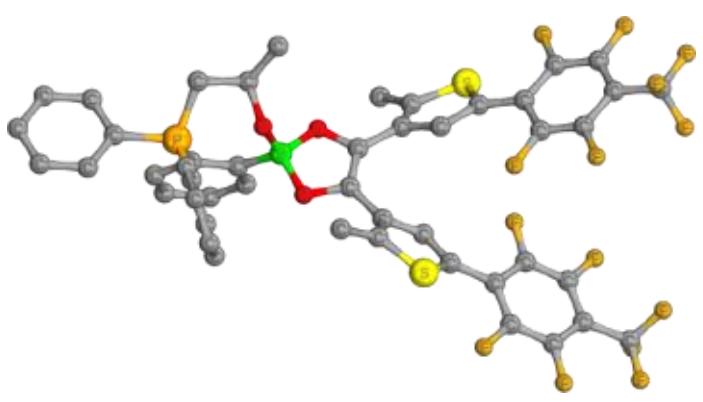

96

xyz, charge: 0, multiplicity: 1

C	-4.116458	-2.453009	-0.514834
C	-4.359406	-3.624110	-1.231801
C	-4.339888	-2.503986	0.868664
C	-4.758056	-3.678943	1.504436
C	-4.979346	-4.820237	0.757763
C	-4.783367	-4.789168	-0.616754
H	-4.205108	-3.614016	-2.303381
H	-4.910508	-3.716410	2.574148
H	-5.303062	-5.728795	1.247472
H	-4.959324	-5.680510	-1.205689
P	-4.040886	-1.047795	1.893219
C	1.199375	-1.106794	-9.010448
C	2.568073	-1.287156	-9.175341
C	0.629410	-0.928422	-7.768308
C	1.391696	-0.922863	-6.598167
C	2.760286	-1.103528	-6.779131
C	3.340373	-1.278583	-8.022610
F	0.402885	-1.094497	-10.071620
F	-0.686242	-0.752766	-7.719345
F	3.566552	-1.112433	-5.720526
F	4.654309	-1.445047	-8.059419
C	3.111383	-1.474894	-10.566808
C	0.796361	-0.724997	-5.285527
C	-0.481187	-1.008852	-4.886994
C	-0.761403	-0.634615	-3.547715
C	0.326490	-0.068513	-2.929344
S	1.677979	-0.012331	-3.976439
H	-1.216879	-1.489675	-5.511954
C	0.419055	0.462929	-1.540196
H	-0.507937	0.959147	-1.257100
H	1.236524	1.175369	-1.434118
H	0.575980	-0.340209	-0.820454
C	-2.063522	-0.801269	-2.923931
F	4.429017	-1.639503	-10.589799
F	2.829848	-0.419704	-11.333292
F	2.567979	-2.548365	-11.145918
H	-5.331720	-2.354100	-4.777848
S	-4.856517	0.429055	-6.944420
C	-0.955214	3.443582	-8.558065
C	-3.614470	2.865330	-8.576722
C	-4.758026	-0.414378	-5.460179
C	-1.478290	2.564772	-7.634393
C	-5.796504	-1.424118	-5.104029
C	-3.285065	-0.568071	-3.438859
C	-3.651949	-0.033337	-4.738082
C	-1.745464	4.060302	-9.521364
C	-2.837012	2.239975	-7.604691
C	-1.096101	5.007581	-10.494369
C	-2.890093	0.953479	-5.416143
C	-3.394452	1.305021	-6.639404
C	-3.096614	3.745582	-9.509524
F	0.347808	3.691344	-8.516263
F	-3.945759	4.275190	-10.377732
F	-0.632643	2.024502	-6.763690
F	-4.921459	2.619616	-8.630358
F	-0.515003	6.022968	-9.850391
F	-0.149842	4.387585	-11.201912
F	-1.957158	5.530903	-11.359392
H	-2.011000	1.396528	-4.977392
H	-6.443182	-1.651917	-5.950457
H	-6.421719	-1.073322	-4.283758
O	-2.144417	-1.259323	-1.652017
O	-4.256534	-0.899645	-2.550188
B	-3.555601	-1.131645	-1.278224
O	-3.669755	0.016415	-0.356744
C	-2.288916	-0.664999	2.039202
C	-1.345127	-1.556212	1.544621
C	-1.882731	0.487375	2.705882
C	0.004378	-1.293734	1.726311
C	-0.533561	0.748104	2.873440
C	0.411116	-0.145095	2.386380
H	-1.652240	-2.444010	1.009061
H	-2.610207	1.184545	3.103406
H	0.737838	-1.988485	1.339762
H	-0.219761	1.647799	3.385259
H	1.465440	0.058034	2.519189
C	-4.559172	-1.479120	3.583572
C	-3.623418	-1.784082	4.568089
C	-5.918459	-1.549513	3.892001
C	-4.040958	-2.140210	5.842362
C	-6.331837	-1.905711	5.164123
C	-5.392137	-2.198760	6.142817
H	-2.565096	-1.745595	4.348152

H -6.666982 -1.344798 3.136712
H -3.304849 -2.372533 6.600023
H -7.388190 -1.956607 5.390824
H -5.715296 -2.474890 7.137643
C -5.049326 0.407949 1.452155
C -4.946604 0.504411 -0.070809
C -5.137229 1.916534 -0.589084
H -6.113595 2.315257 -0.307984
H -4.366929 2.580925 -0.194695
H -5.068645 1.922300 -1.675890
H -5.732942 -0.130737 -0.500634
H -4.601070 1.272542 1.950727
H -6.066839 0.308336 1.831133

1-c-PO

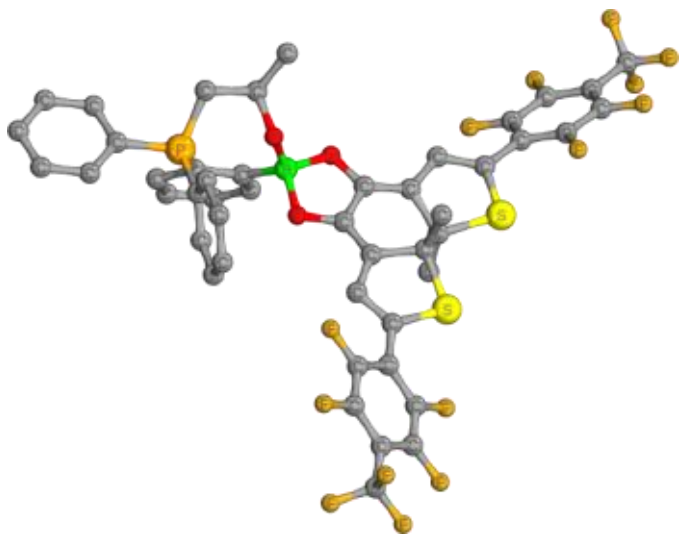

96
xyz, charge: 0, multiplicity: 1
C -4.071063 -1.659171 -0.197236
C -4.347411 -2.953575 -0.633297
C -4.117415 -1.441763 1.190042
C -4.384488 -2.474827 2.092006
C -4.639741 -3.748592 1.617701
C -4.629343 -3.983178 0.250413
H -4.338574 -3.158858 -1.695987
H -4.391159 -2.299259 3.159261
H -4.846407 -4.550968 2.312932
H -4.836634 -4.976317 -0.127128
P -3.809658 0.211742 1.838179
C 4.782066 0.824432 -2.414627
C 5.724702 0.186615 -3.214755
C 3.429449 0.683208 -2.627850
C 2.918087 -0.117102 -3.653486
C 3.872828 -0.745190 -4.449877
C 5.234465 -0.601382 -4.246979
F 5.182102 1.607641 -1.418214
F 2.608856 1.360978 -1.827256
F 3.481486 -1.535406 -5.442613
F 6.041639 -1.257283 -5.066884
C 7.185096 0.390354 -2.914133
C 1.491101 -0.270009 -3.884732
C 0.521178 -0.224395 -2.939163
C -0.793439 -0.369335 -3.452626
C -0.861234 -0.171597 -4.958736
S 0.856077 -0.546068 -5.518498
H 0.707178 -0.129344 -1.879808
C -1.136361 1.308309 -5.246209
H -0.406253 1.920240 -4.717716
H -2.131874 1.590080 -4.900707
H -1.067639 1.534866 -6.308305
C -1.950361 -0.550199 -2.781318
F 7.982610 -0.277105 -3.738924
F 7.473436 -0.010927 -1.672708
F 7.513358 1.681494 -3.002420
H -0.664825 -2.829531 -6.064722
S -2.329267 -0.749517 -7.303643
C -5.707913 -0.573384 -10.360748
C -6.087210 -2.000538 -8.044384
C -1.919050 -1.117378 -5.544590
C -4.833584 -0.441275 -9.295969
C -1.535197 -2.598195 -5.453961
C -3.209046 -0.733316 -3.476769
C -3.243137 -0.914399 -4.814636
C -6.803054 -1.422856 -10.283362
C -4.989091 -1.140613 -8.103066
C -7.808212 -1.621576 -11.386565
C -4.364388 -1.049149 -5.679094
C -4.053924 -1.002262 -6.995780
C -6.965271 -2.132624 -9.097863
F -5.461399 0.151504 -11.441732
F -7.982522 -2.975848 -8.967072
F -3.819289 0.404581 -9.432469
F -6.303320 -2.740879 -6.962376
F -7.832482 -2.896011 -11.783085
F -9.037446 -1.308441 -10.968919
F -7.555544 -0.879616 -12.458111
H -5.373181 -1.130514 -5.301888
H -2.368062 -3.209283 -5.800213
H -1.310876 -2.876094 -4.423239
O -2.155026 -0.569591 -1.477111
O -4.204004 -0.735797 -2.600783
B -3.619409 -0.504625 -1.249419
O -3.898709 0.820826 -0.738658
C -2.112244 0.717201 1.540945
C -1.145454 -0.266523 1.367816
C -1.756378 2.061850 1.508434
C 0.175370 0.098517 1.160118
C -0.436792 2.418245 1.294573
C 0.529647 1.437261 1.116046
H -1.418501 -1.313474 1.367735
H -2.499305 2.838382 1.634427
H 0.924537 -0.668081 1.015147
H -0.163287 3.463623 1.252783
H 1.557913 1.718964 0.933864
C -4.033849 0.181449 3.635359
C -2.947918 0.330454 4.492339
C -5.311158 0.002064 4.169544
C -3.138777 0.304876 5.866504
C -5.496074 -0.024717 5.540576
C -4.408804 0.128801 6.390788
H -1.951705 0.469931 4.094381
H -6.167395 -0.133962 3.520015
H -2.289826 0.424107 6.525801
H -6.488738 -0.166059 5.945946
H -4.554681 0.110399 7.462574
C -5.038426 1.402887 1.209065
C -5.179627 1.146913 -0.295623
C -5.725265 2.355342 -1.032392
H -5.833238 2.126501 -2.091566
H -6.703347 2.646974 -0.646525
H -5.049316 3.206132 -0.937160
H -5.884119 0.314389 -0.427959
H -4.664729 2.409073 1.418865
H -5.973002 1.289261 1.760900

2-o-PhCHO

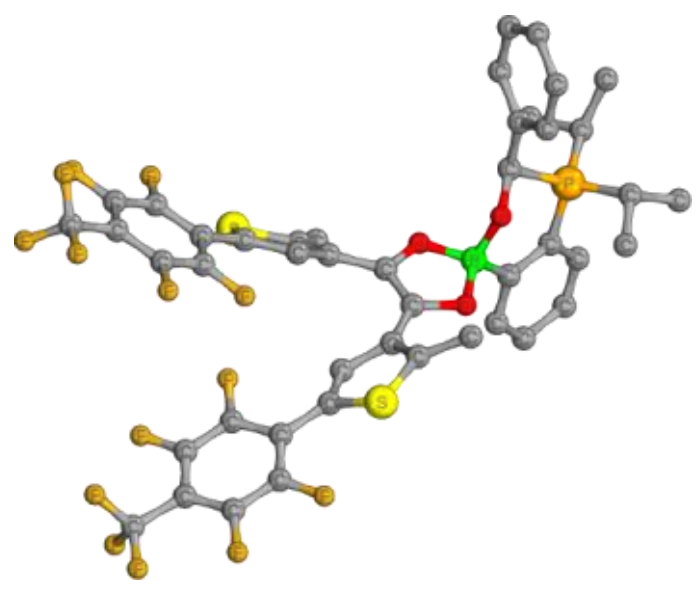

98
xyz, charge: 0, multiplicity: 1
C 14.132504 7.247195 3.977259
C 13.762962 6.088208 3.290448
C 14.328935 7.116827 5.355862
C 14.165898 5.896463 6.019832
C 13.795361 4.774532 5.306350
C 13.590330 4.876832 3.934706
H 13.598725 6.147107 2.221899
H 14.332095 5.809576 7.087115
H 13.668148 3.826922 5.812288
H 13.296068 4.001331 3.369971

P	14.768139	8.585012	6.286184
C	16.032549	8.130503	7.539427
C	16.315472	9.247168	8.542457
H	16.765154	10.118216	8.070907
H	17.013420	8.881890	9.296678
H	15.420773	9.581815	9.066426
C	17.304821	7.638742	6.850485
H	15.595974	7.286519	8.082960
H	17.823451	8.447003	6.335404
H	17.106693	6.849003	6.126381
H	17.991552	7.239532	7.597232
C	13.276142	9.234666	7.144192
C	12.098693	9.403559	6.183831
H	11.801262	8.446144	5.756189
H	12.309870	10.074070	5.356872
H	11.247608	9.799593	6.739625
C	12.875930	8.316103	8.299648
H	13.567974	10.212177	7.541487
H	13.655074	8.190904	9.050868
H	12.582311	7.329834	7.938827
H	12.009359	8.742186	8.806128
C	19.144915	12.441022	-4.212437
C	18.119674	13.142641	-4.835393
C	18.898720	11.558370	-3.184906
C	17.613022	11.306653	-2.705067
C	16.594188	12.013217	-3.341212
C	16.833472	12.904634	-4.372715
F	20.401371	12.619826	-4.599512
F	19.939125	10.932026	-2.641622
F	15.327717	11.847392	-2.975058
F	15.780415	13.511011	-4.900484
C	18.466247	14.094841	-5.948406
C	17.352728	10.362251	-1.629888
C	16.288069	10.332189	-0.771633
C	16.306608	9.239156	0.131637
C	17.413567	8.445117	-0.041915
S	18.415306	9.038767	-1.293355
H	15.509457	11.077356	-0.749342
C	17.791481	7.208410	0.700791
H	18.561588	6.643523	0.177075
H	16.926595	6.559574	0.830445
H	18.165382	7.445939	1.696566
C	15.261094	8.998363	1.110110
F	19.075286	13.454233	-6.948291
F	19.298494	15.045495	-5.515731
F	17.403034	14.704598	-6.458509
H	10.803844	11.897229	0.852067
S	11.404287	10.713728	-1.637080
C	13.885668	9.137629	-5.905561
C	11.540232	9.758183	-4.673250
C	12.059605	10.429539	-0.084626
C	13.838358	9.268068	-4.534754
C	11.446583	11.059431	1.119908
C	13.931961	9.176074	1.000533
C	13.150236	9.597262	-0.147757
C	12.761479	9.314620	-6.703654
C	12.656884	9.583890	-3.859895
C	12.898105	9.154860	-8.193941
C	13.443922	9.202269	-1.477248
C	12.602521	9.736252	-2.414580
C	11.580029	9.632521	-6.049904
F	15.051780	8.840803	-6.465058
F	10.445412	9.820445	-6.707474
F	14.972601	9.086567	-3.867370
F	10.367634	10.061473	-4.121843
F	13.327204	7.928318	-8.501282
F	11.754607	9.346156	-8.841481
F	13.783056	10.024181	-8.685850
H	14.248126	8.521937	-1.705716
H	12.220227	11.425366	1.793274
H	10.848680	10.338813	1.678052
O	15.588985	8.529554	2.339826
O	13.299247	8.873617	2.161713
B	14.350596	8.623364	3.134565
O	14.405782	9.799933	4.047545
C	15.392217	9.779608	4.993540
C	15.689050	11.140054	5.563107
C	14.680267	12.082549	5.733416
C	14.968289	13.325393	6.275644
C	16.267036	13.648154	6.640791
C	17.282881	12.725028	6.445646
C	16.992872	11.482184	5.904675
H	17.797679	10.777368	5.728327
H	13.670743	11.857173	5.417693
H	14.175838	14.051531	6.399994
H	16.489265	14.621531	7.057195
H	18.304233	12.977668	6.697617
H	16.335776	9.339439	4.631722

2-c-PhCHO

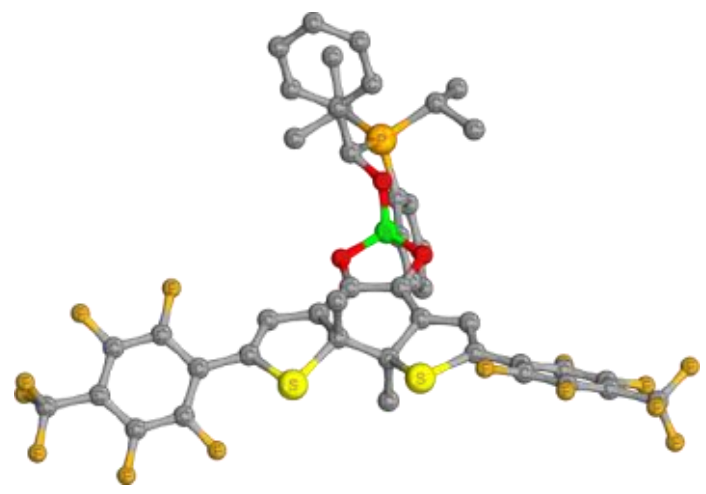

98

xyz, charge: 0, multiplicity: 1

C	14.804437	7.501088	3.066141
C	15.166874	6.510086	2.151184
C	14.572355	7.081967	4.380073
C	14.687952	5.742612	4.764951
C	15.041681	4.791077	3.829878
C	15.280658	5.181446	2.517105
H	15.359261	6.787266	1.122155
H	14.512980	5.434645	5.788881
H	15.134368	3.753223	4.119928
H	15.558690	4.440396	1.778869
P	14.097131	8.320369	5.585166
C	14.953522	7.961615	7.169789
C	14.452333	8.817575	8.331654
H	14.946040	8.496455	9.249408
H	13.379012	8.724221	8.493736
H	14.673982	9.873019	8.189604
C	16.467967	8.060424	6.991011
H	14.707291	6.916962	7.385342
H	16.790536	9.089498	6.835121
H	16.829363	7.461574	6.155559
H	16.963250	7.701966	7.893515
C	12.278908	8.262307	5.850627
C	11.508908	8.325494	4.531477
H	11.702422	9.228196	3.960603
H	10.440790	8.281732	4.748289
H	11.752431	7.477205	3.891906
C	11.893321	6.997198	6.619254
H	12.042246	9.142749	6.456642
H	12.371218	6.913579	7.594805
H	12.120781	6.098702	6.044613
H	10.816597	7.002834	6.790443
C	22.087562	13.249024	0.996830
C	22.965373	13.158220	-0.078348
C	20.791718	12.788848	0.919511
C	20.286909	12.196661	-0.239418
C	21.172321	12.117133	-1.309719
C	22.473531	12.583151	-1.241768
F	22.490955	13.804908	2.133403
F	20.015606	12.945396	1.986973
F	20.776268	11.559604	-2.447684
F	23.225172	12.443641	-2.323404
C	24.366664	13.682167	0.088082
C	18.916819	11.714251	-0.329411
C	18.202743	11.150175	0.672326
C	16.874041	10.801473	0.308313
C	16.431818	11.462159	-0.989540
S	18.018022	11.838238	-1.848999
H	18.605561	10.931856	1.650061
C	15.752978	12.793734	-0.651449
H	16.413388	13.384505	-0.017738

H 14.821032 12.625595 -0.110314
H 15.528501 13.372783 -1.544924
C 15.966262 10.067521 0.984829
F 24.991699 13.052670 1.086592
F 24.355209 14.984511 0.380975
F 25.110382 13.529017 -1.000792
H 16.775713 8.735324 -1.481868
S 14.677974 11.286837 -3.197597
C 10.777451 11.440572 -5.552548
C 11.271070 9.273089 -3.938934
C 15.534105 10.494867 -1.771427
C 11.802142 11.468198 -4.622955
C 16.272980 9.258865 -2.296360
C 14.671135 9.759319 0.413358
C 14.392031 10.030928 -0.878219
C 9.967833 10.321462 -5.687969
C 12.084876 10.396002 -3.783091
C 8.827353 10.207036 -6.663956
C 13.176334 9.857404 -1.594780
C 13.174825 10.433746 -2.818830
C 10.245203 9.240461 -4.857698
F 10.598011 12.525834 -6.290458
F 9.518437 8.133237 -4.950500
F 12.524243 12.578624 -4.533300
F 11.483285 8.183692 -3.208729
F 9.028779 9.197118 -7.513266
F 7.679260 9.969861 -6.024297
F 8.654104 11.304807 -7.390102
H 12.323878 9.351828 -1.165843
H 17.019879 9.519090 -3.043546
H 15.556317 8.576685 -2.752416
O 16.051808 9.540343 2.198076
O 13.912010 9.160123 1.321669
B 14.693793 9.041330 2.560304
O 14.073784 9.916626 3.557013
C 14.634761 9.928517 4.808492
C 14.220732 11.127723 5.615976
C 12.938211 11.656104 5.511173
C 12.568866 12.750269 6.277791
C 13.476919 13.337143 7.146852
C 14.765774 12.833993 7.234382
C 15.133851 11.739363 6.467808
H 16.152176 11.371086 6.520966
H 12.232224 11.233142 4.809467
H 11.570335 13.156025 6.184166
H 13.186986 14.195215 7.738264
H 15.491060 13.303583 7.885322
H 15.735514 9.880508 4.793866

2-o-PhNCS

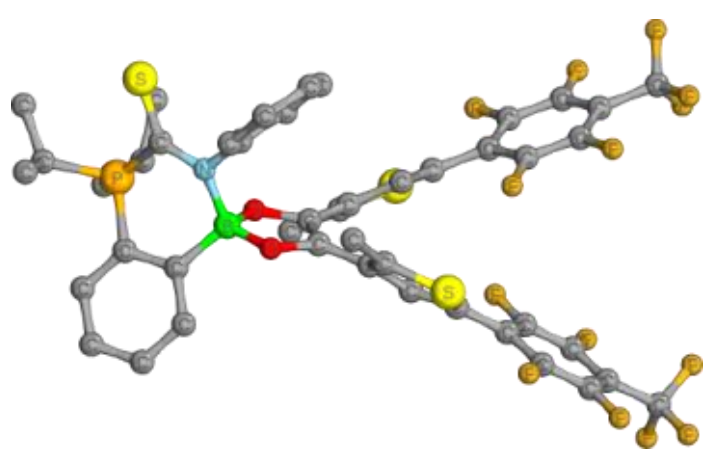

98
xyz, charge: 0, multiplicity: 1
C -5.284319 4.873592 8.956851
C -4.209409 5.326572 9.721410
C -6.454156 4.537959 9.642858
C -6.549604 4.631347 11.031841
C -5.463233 5.076586 11.761142
C -4.293949 5.428585 11.099910
H -3.292284 5.608502 9.221219
H -7.459399 4.368263 11.556482
H -5.529732 5.153467 12.838013
H -3.443799 5.785910 11.666752
P -7.815540 3.976353 8.618610
C -9.370232 4.415200 9.491041
C -10.643736 3.730780 9.005342
H -10.569465 2.644873 9.024995
H -10.901068 4.041113 7.997011
H -11.459685 4.009597 9.673562
C -9.525226 5.933748 9.564710
H -9.168225 4.040893 10.500918
H -10.348168 6.175646 10.237884
H -9.758499 6.345012 8.584363
H -8.631833 6.429238 9.944402
C -7.621068 2.164716 8.371220
C -7.875129 1.431199 9.686306
H -7.268759 1.822304 10.503898
H -7.607436 0.381246 9.564140
H -8.923022 1.462218 9.985588
C -8.416054 1.594792 7.202480
H -6.558240 2.083561 8.128733
H -9.492349 1.683276 7.336486
H -8.178467 0.534154 7.108069
H -8.158150 2.075595 6.261736
C 2.988485 2.151796 4.275582
C 4.109712 2.939055 4.040875
C 1.751313 2.699597 4.535698
C 1.547127 4.080233 4.583673
C 2.679123 4.854905 4.346253
C 3.923696 4.313129 4.080496
F 3.091952 0.829349 4.244155
F 0.742767 1.857955 4.736466
F 2.582441 6.181699 4.370444
F 4.920804 5.160267 3.875602
C 5.425233 2.261751 3.762865
C 0.241320 4.665365 4.845465
C -0.817306 4.103599 5.504117
C -1.975768 4.921245 5.535977
C -1.776328 6.127317 4.908726
S -0.188714 6.246301 4.290215
H -0.783591 3.140001 5.985882
C -2.737403 7.250357 4.720760
H -2.961841 7.738321 5.668546
H -3.679530 6.886531 4.314222
H -2.347337 8.002900 4.036741
C -3.214603 4.497320 6.161400
F 5.339608 1.486236 2.680398
F 5.781934 1.483701 4.787442
F 6.415609 3.121419 3.556254
H -3.594926 1.198761 8.281101
S -2.767805 -0.417349 5.142708
C -0.406618 0.838883 0.705327
C -1.940670 -0.945403 2.105056
C -3.361064 0.788713 6.199657
C -1.010973 1.208739 1.894750
C -3.843299 0.420470 7.561876
C -3.746086 3.265655 6.242555
C -3.316153 2.029775 5.614465
C -0.557441 -0.440081 0.189296
C -1.801093 0.335456 2.638167
C 0.063107 -0.922333 -1.095622
C -2.788210 1.981990 4.297972
C -2.423331 0.728752 3.893961
C -1.340110 -1.322335 0.924573
F 0.324651 1.756432 0.091289
F -1.531331 -2.560511 0.489843
F -0.802457 2.451772 2.314145
F -2.677321 -1.848339 2.746141
F 0.785446 0.010644 -1.703235
F 0.865292 -1.964330 -0.870981
F -0.879148 -1.317615 -1.954680
H -2.709625 2.863523 3.681755
H -3.398479 -0.511893 7.907660
H -4.927569 0.293412 7.577090
O -3.980343 5.404309 6.821691
O -4.870288 3.271272 7.008675
B -5.112790 4.683346 7.363983
S -9.090228 4.952778 6.155635
N -6.454569 5.142835 6.616038
C -6.317635 5.728491 5.322631
C -6.487420 7.094798 5.167496
C -6.358430 7.665228 3.911466
C -6.053961 6.873002 2.813798
C -5.879730 5.506841 2.977344

C -6.015821 4.929334 4.230528
H -5.896392 3.861854 4.359208
H -5.951365 7.319562 1.833804
H -5.642697 4.883869 2.125169
H -6.722242 7.705173 6.029227
H -6.493005 8.731912 3.790860
C -7.668725 4.805523 6.991773

2-c-PhNCS

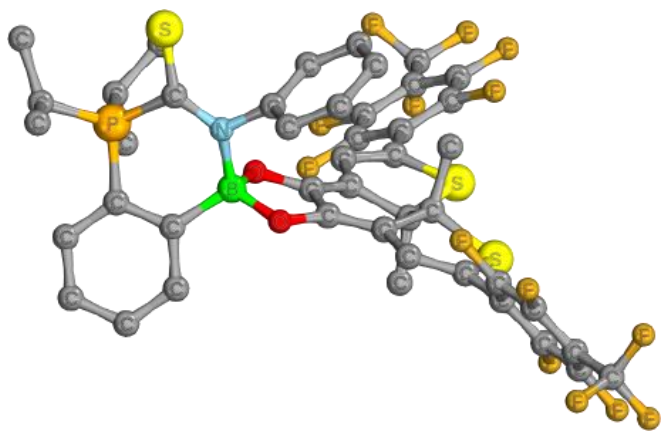

98
xyz, charge: 0, multiplicity: 1
C -1.588398 -0.954792 1.263354
C -0.812770 -2.114790 1.291289
C -2.403434 -0.711363 2.369716
C -2.463437 -1.591715 3.451125
C -1.687801 -2.734439 3.445243
C -0.858632 -2.991430 2.361141
H -0.156807 -2.332014 0.459006
H -3.105368 -1.399812 4.301566
H -1.726920 -3.419366 4.281386
H -0.244218 -3.882329 2.353710
P -3.402151 0.775957 2.315383
C -3.578967 1.376112 4.042434
C -4.647688 2.439106 4.280792
H -5.612662 2.181132 3.845128
H -4.343901 3.401511 3.881791
H -4.798489 2.539010 5.356335
C -2.224522 1.780624 4.618772
H -3.915379 0.463372 4.549131
H -1.469220 1.004399 4.498979
H -2.332427 1.974943 5.686121
H -1.862025 2.692055 4.146397
C -5.090567 0.401983 1.633157
C -5.508493 1.370228 0.530845
H -4.876267 1.257781 -0.349555
H -5.471979 2.410210 0.847792
H -6.532522 1.140830 0.234585
C -5.206932 -1.052112 1.182480
H -5.750347 0.547930 2.493992
H -4.556629 -1.267006 0.337206
H -6.235985 -1.233963 0.869822
H -4.974757 -1.760240 1.976214
C 5.374193 -0.954977 -6.186196
C 6.165567 0.071934 -5.690843
C 4.056168 -1.090984 -5.786294
C 3.453187 -0.222937 -4.883605
C 4.255425 0.811453 -4.402601
C 5.569899 0.950289 -4.791882
F 5.843264 -1.847508 -7.044403
F 3.358721 -2.104865 -6.283731
F 3.757802 1.712373 -3.562674
F 6.269161 1.963330 -4.295688
C 7.608487 0.291534 -6.063684
C 2.060238 -0.369498 -4.482361
C 1.558825 -0.124042 -3.251810
C 0.149765 -0.305209 -3.163115
C -0.516514 -0.353407 -4.530750
S 0.842628 -0.892407 -5.653419
H 2.159561 0.149193 -2.397120
C -0.912867 1.072102 -4.926875
H -1.260538 1.123525 -5.956532
H -0.052707 1.731576 -4.818382
H -1.704063 1.445390 -4.276670
C -0.641376 -0.343232 -2.076447
F 7.773857 1.488771 -6.629421
F 8.066635 -0.615943 -6.916941
F 8.387433 0.247314 -4.980520
H -2.176071 -3.410972 -4.198932
S -2.777562 -1.224520 -5.981269
C -7.730260 -2.598916 -5.562882
C -5.866123 -1.083804 -6.856137
C -1.699837 -1.329936 -4.490185
C -6.508794 -2.354588 -4.974595
C -1.285347 -2.794391 -4.313626
C -2.065435 -0.563183 -2.185095
C -2.630170 -0.947520 -3.346322
C -8.055484 -2.093191 -6.817009
C -5.531936 -1.579344 -5.600335
C -9.413469 -2.403904 -7.389717
C -4.001240 -1.148892 -3.666101
C -4.235904 -1.323981 -4.986644
C -7.088777 -1.329970 -7.456380
F -8.605387 -3.350135 -4.905810
F -7.295884 -0.797702 -8.650799
F -6.273560 -2.901142 -3.786337
F -4.995781 -0.329077 -7.515485
F -9.609642 -1.857072 -8.582883
F -9.580907 -3.720991 -7.522967
F -10.378850 -1.955394 -6.583661
H -4.778638 -1.116376 -2.917147
H -0.667319 -2.916899 -3.423327
H -0.723389 -3.162685 -5.169528
O -0.315837 -0.166731 -0.795281
O -2.634945 -0.357671 -0.999035
B -1.550899 -0.009809 -0.035054
S -2.917957 3.574675 1.562700
N -1.729353 1.516181 0.333104
C -1.141965 2.442202 -0.584078
C 0.200250 2.764953 -0.470019
C 0.782445 3.607317 -1.403356
C 0.025107 4.127781 -2.442138
C -1.319190 3.805221 -2.545803
C -1.905668 2.958374 -1.619467
H -2.952948 2.698076 -1.698642
H 0.483387 4.781804 -3.171761
H -1.913458 4.205844 -3.356116
H 0.785337 2.350684 0.339491
H 1.832475 3.853836 -1.321201
C -2.534628 1.996260 1.257660

2-o-tBuNC

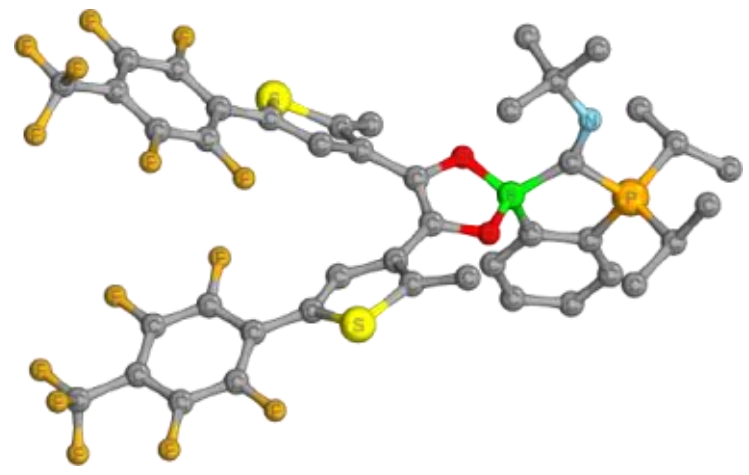

99
xyz, charge: 0, multiplicity: 1
C 14.215875 11.755084 5.166603
C 13.185192 10.817237 5.262733
C 14.196516 12.805861 6.087550
C 13.204318 12.949773 7.057871
C 12.201155 12.003456 7.123157
C 12.197753 10.937077 6.224231
H 13.152117 9.986989 4.567512
H 13.207582 13.784031 7.749292
H 11.420152 12.089154 7.866817
H 11.407106 10.199413 6.279975
P 15.592257 13.873589 5.819627
C 16.690337 14.032980 7.270891
C 17.030853 12.643742 7.805174
H 16.147537 12.134727 8.191282
H 17.748573 12.733327 8.621394
H 17.478527 12.010125 7.040600
C 16.114043 14.936362 8.356081
H 17.598519 14.479751 6.852109
H 15.881950 15.938895 7.996172

H 16.837468 15.044086 9.165140
H 15.208309 14.513004 8.791909
C 15.080739 15.525731 5.215016
C 16.295634 16.417345 4.968690
H 16.975468 15.969995 4.244594
H 16.862536 16.616298 5.878265
H 15.966621 17.379086 4.573811
C 14.227599 15.361856 3.958207
H 14.461794 15.959935 6.008206
H 13.878979 16.341268 3.628857
H 13.353077 14.735668 4.129014
H 14.797096 14.917424 3.141837
C 17.672108 4.321536 -1.787140
C 17.292800 4.849023 -3.012496
C 17.435770 5.011707 -0.612136
C 16.806746 6.253975 -0.568590
C 16.436358 6.771273 -1.812966
C 16.671711 6.092278 -2.988671
F 18.275878 3.146918 -1.687383
F 17.831535 4.436454 0.521308
F 15.827857 7.948838 -1.906237
F 16.278278 6.654907 -4.124104
C 17.504822 4.170658 -4.339250
C 16.528782 6.944536 0.681613
C 16.227440 8.266508 0.872301
C 15.920558 8.601600 2.216296
C 16.021676 7.516700 3.054757
S 16.477478 6.109607 2.199363
H 16.237123 9.007481 0.090309
C 15.790264 7.448647 4.526907
H 14.872500 7.967625 4.799731
H 16.601997 7.925819 5.075225
H 15.708835 6.418980 4.872763
C 15.536519 9.937529 2.635674
F 16.341862 3.983194 -4.965898
F 18.088307 2.983467 -4.225586
F 18.274587 4.917780 -5.134082
H 14.014682 13.784061 0.664424
S 13.149777 11.346538 -1.583866
C 10.666477 8.062726 -3.503625
C 12.481850 7.354269 -1.592765
C 14.024226 11.824562 -0.194876
C 11.305149 9.027967 -2.757453
C 14.554678 13.214794 -0.091894
C 14.820003 10.876174 1.985611
C 14.132940 10.793964 0.708129
C 10.914127 6.708331 -3.313269
C 12.238941 8.715185 -1.768849
C 10.174681 5.710446 -4.164472
C 13.490092 9.614996 0.248978
C 12.922144 9.737017 -0.989855
C 11.841831 6.377374 -2.335977
F 9.789223 8.454617 -4.418009
F 12.161728 5.118554 -2.076117
F 11.007320 10.300785 -3.003091
F 13.361589 6.936980 -0.687741
F 10.432976 5.908598 -5.458415
F 10.495528 4.453420 -3.883900
F 8.855711 5.830467 -3.995541
H 13.440908 8.720053 0.847912
H 14.472886 13.747293 -1.038558
H 15.605147 13.204931 0.196653
O 15.963185 10.394205 3.842261
O 14.771592 12.033575 2.689595
B 15.373264 11.729064 4.005589
C 16.435146 12.910262 4.503535
N 17.619359 13.326895 4.345531
C 18.603410 12.766892 3.418598
C 19.696798 13.830584 3.298728
H 20.106814 14.079045 4.278113
H 19.302714 14.748561 2.858483
C 19.188448 11.504293 4.055775
H 19.585556 11.717754 5.049898
H 18.435359 10.722654 4.139942
C 18.046747 12.466683 2.029249
H 18.868155 12.235835 1.349033
H 17.371574 11.616473 2.017631
H 17.518201 13.332544 1.627087
H 20.009432 11.125215 3.445456
H 20.513338 13.481355 2.665729

2-c-tBuNC_E

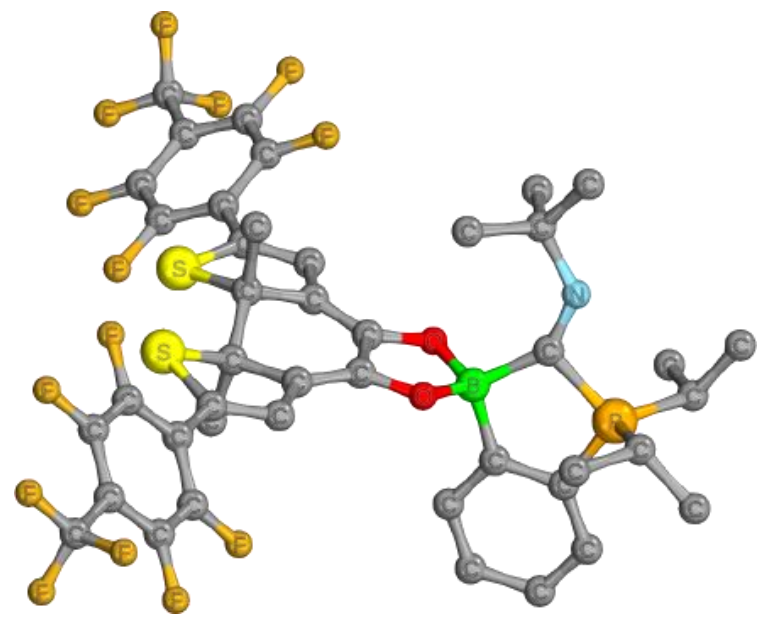

99
xyz, charge: 0, multiplicity: 1
C 14.891260 9.382559 4.864785
C 15.382377 8.095314 4.641538
C 14.177186 9.592341 6.047480
C 13.954395 8.585506 6.986616
C 14.450928 7.322287 6.732331
C 15.162896 7.082081 5.557740
H 15.946534 7.882068 3.741250
H 13.411541 8.780470 7.904014
H 14.292456 6.522684 7.443412
H 15.551667 6.090137 5.366222
P 13.673520 11.292196 6.169859
C 14.287142 12.051794 7.719512
C 13.779352 13.480372 7.900049
H 14.182933 13.893521 8.825056
H 12.692709 13.532847 7.969985
H 14.096278 14.114474 7.074239
C 15.812655 11.976746 7.748136
H 13.889554 11.421389 8.523425
H 16.252666 12.598833 6.969192
H 16.181244 10.959604 7.618241
H 16.176257 12.340624 8.709428
C 11.865236 11.515552 6.016539
C 11.374206 10.865038 4.725123
H 11.875989 11.257235 3.841774
H 10.306153 11.052609 4.609999
H 11.523338 9.785282 4.739675
C 11.119520 10.985820 7.237485
H 11.735103 12.600642 5.941065
H 10.052028 11.179323 7.127149
H 11.437639 11.455833 8.168555
H 11.240939 9.906531 7.339126
C 23.243707 11.509432 1.113434
C 24.033171 10.573110 0.453893
C 21.867927 11.432927 1.109781
C 21.189928 10.405732 0.451842
C 21.989027 9.482401 -0.214279
C 23.371278 9.555778 -0.219484
F 23.816498 12.516788 1.760607
F 21.192563 12.385303 1.743838
F 21.422130 8.471226 -0.860998
F 24.026149 8.611000 -0.876949
C 25.530610 10.721226 0.517265
C 19.736252 10.309792 0.456115
C 18.924322 10.620956 1.492270
C 17.541186 10.427258 1.219270
C 17.253519 10.268848 -0.268178
S 18.869503 9.725854 -0.971168
H 19.281252 10.930482 2.463565
C 16.926663 11.646198 -0.854341
H 16.845098 11.616734 -1.939005
H 17.716789 12.348217 -0.590115
H 15.984171 12.022875 -0.454315
C 16.492467 10.436212 2.067314
F 25.953044 10.696131 1.783687
F 25.915818 11.884597 -0.011196
F 26.174048 9.759100 -0.132434

H	15.676546	7.154456	-0.143428
S	15.469909	9.188844	-2.179754
C	10.748954	7.283346	-2.886431
C	12.683705	8.982743	-3.790479
C	16.133763	9.236947	-0.460390
C	11.742617	7.715263	-2.035075
C	16.544156	7.810147	-0.078671
C	15.149783	10.124235	1.615774
C	14.925382	9.630459	0.380803
C	10.693524	7.689155	-4.215929
C	12.747105	8.585265	-2.459432
C	9.574028	7.172565	-5.081342
C	13.695525	9.296921	-0.252705
C	13.816733	9.018669	-1.570697
C	11.690900	8.550409	-4.652820
F	9.836092	6.444629	-2.411999
F	11.729544	8.999939	-5.897964
F	11.734985	7.256333	-0.788453
F	13.597174	9.819959	-4.266683
F	8.387620	7.516025	-4.573739
F	9.621504	7.638576	-6.323319
F	9.608635	5.840487	-5.155291
H	12.753800	9.304914	0.276254
H	16.922324	7.778812	0.944505
H	17.317699	7.419978	-0.736716
O	16.469813	10.740621	3.361055
O	14.283448	10.366021	2.591709
B	15.071642	10.622784	3.828874
C	14.506256	11.935974	4.665881
N	14.466332	13.199564	4.642034
C	15.051299	14.067459	3.621545
C	14.338053	15.412121	3.779385
H	14.433178	15.786802	4.799151
H	13.274368	15.319616	3.554095
C	16.535827	14.231530	3.958232
H	16.669970	14.602046	4.976019
H	17.066139	13.285018	3.860363
H	16.994965	14.951288	3.279413
H	14.758650	16.156776	3.102951
C	14.876899	13.575587	2.186180
H	15.555494	12.766488	1.934746
H	13.855543	13.245350	1.996097
H	15.095158	14.390663	1.494697

2-c-tBuNC_Z

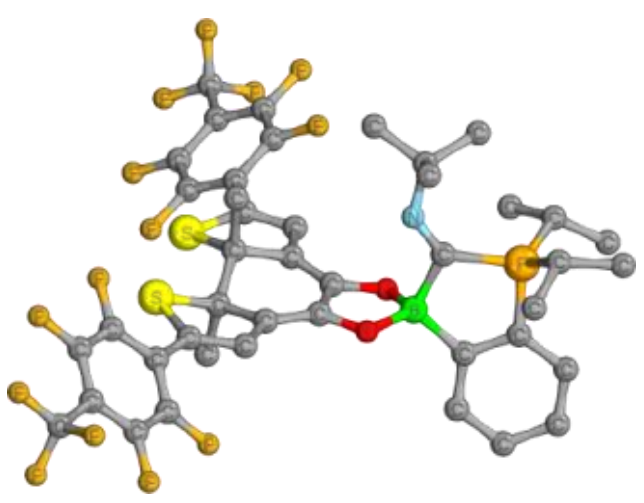

99
xyz, charge: 0, multiplicity: 1

C	14.105540	8.896232	4.923373
C	13.789926	7.630808	5.415779
C	13.927528	9.123821	3.557228
C	13.450830	8.130569	2.698330
C	13.147969	6.886045	3.215678
C	13.318913	6.638051	4.575891
H	13.922527	7.424631	6.470445
H	13.312545	8.314448	1.640817
H	12.779742	6.105494	2.563390
H	13.081809	5.659598	4.973515
P	14.410098	10.777382	3.050400
C	12.911508	11.723470	2.531303
C	12.145571	12.149235	3.785108
H	11.264425	12.719907	3.489715
H	12.736591	12.769815	4.455729
H	11.804492	11.285896	4.356936
C	11.995096	10.936357	1.596921
H	13.280503	12.611141	2.010609
H	11.563196	10.073362	2.102174
H	12.488332	10.590782	0.690247
H	11.169796	11.580017	1.289548
C	15.717827	10.663294	1.752092
C	16.782938	9.676125	2.228712
H	17.154421	9.909970	3.226804
H	17.632468	9.704983	1.545529
H	16.402511	8.655405	2.247991
C	15.214710	10.328539	0.352474
H	16.165120	11.659422	1.717662
H	16.063972	10.296266	-0.331462
H	14.521301	11.075220	-0.032099
H	14.733777	9.351961	0.308715
C	9.463276	15.415042	10.195816
C	9.016654	15.372955	11.512718
C	10.551076	14.683620	9.772570
C	11.263003	13.851180	10.638209
C	10.811202	13.822684	11.954079
C	9.722837	14.559534	12.388289
F	8.842759	16.188279	9.312106
F	10.925197	14.810515	8.502873
F	11.426717	13.050796	12.841481
F	9.381207	14.443356	13.662583
C	7.818351	16.200432	11.895081
C	12.417017	13.081012	10.197720
C	12.574830	12.508225	8.981117
C	13.819510	11.842815	8.812896
C	14.833932	12.226044	9.882389
S	13.792090	12.820062	11.282132
H	11.812956	12.503594	8.215594
C	15.650612	13.415330	9.365223
H	16.316755	13.813739	10.127777
H	14.973023	14.209369	9.052579
H	16.250442	13.119522	8.503229
C	14.233610	11.032601	7.816640
F	6.754243	15.858102	11.164325
F	8.049816	17.497855	11.681685
F	7.480141	16.064411	13.171429
H	15.553285	9.024162	11.078326
S	17.152546	11.417409	11.279586
C	21.289566	8.340087	11.075484
C	20.303636	10.840112	11.545367
C	15.689647	11.002317	10.236890
C	20.042989	8.691262	10.605263
C	14.908054	9.891986	10.946452
C	15.536533	10.395264	7.854650
C	16.303578	10.432497	8.964160
C	22.080505	9.228804	11.796753
C	19.505666	9.962107	10.818136
C	23.432261	8.768379	12.273704
C	17.618540	9.931382	9.170865
C	18.186072	10.336781	10.330786
C	21.553870	10.492461	12.026470
F	21.728562	7.110055	10.836584
F	22.224255	11.417118	12.697730
F	19.345316	7.768209	9.952397
F	19.872141	12.073060	11.784592
F	24.197568	8.399832	11.243040
F	24.094288	9.706862	12.939815
F	23.316511	7.713859	13.083812
H	18.122834	9.322833	8.434768
H	14.043687	9.589258	10.353860
H	14.554516	10.206923	11.926241
O	13.614610	10.705863	6.689730
O	15.767563	9.788426	6.698117
B	14.651629	10.118925	5.797875
C	15.101186	11.293908	4.726088
N	15.762289	12.283732	5.105305
C	16.247565	13.478961	4.443321
C	16.282859	14.559021	5.528300
H	16.902214	14.239701	6.365190
H	15.282529	14.755728	5.915822
C	17.676326	13.184463	3.979457
H	18.280925	12.816351	4.807661
H	17.698612	12.431482	3.189583

H 18.143715 14.090779 3.593067
H 16.685883 15.493480 5.135745
C 15.395733 13.990380 3.281933
H 15.784962 14.949444 2.939108
H 15.401944 13.334670 2.412254
H 14.362157 14.154147 3.589595

2-o-BP

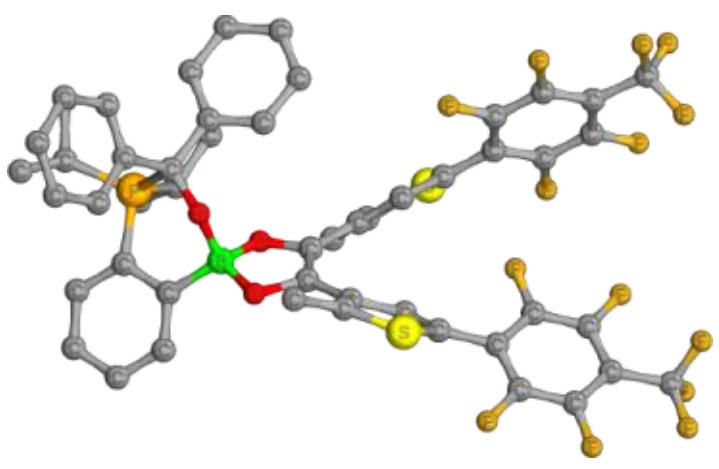

108
xyz, charge: 0, multiplicity: 1
C -2.804863 -0.353030 3.419964
C -2.244956 -1.424383 4.113504
C -3.754659 0.422271 4.096862
C -4.144679 0.125450 5.403177
C -3.573129 -0.948214 6.061565
C -2.619877 -1.721835 5.413327
H -1.496264 -2.030393 3.619957
H -4.887951 0.716747 5.922051
H -3.870802 -1.179308 7.075622
H -2.168845 -2.560969 5.927564
P -4.533839 1.722561 3.118031
C -5.422326 2.825994 4.318210
C -6.334687 3.876804 3.694228
H -6.955336 3.491769 2.886533
H -5.764512 4.727175 3.322778
H -7.004918 4.260268 4.464516
C -4.553965 3.458616 5.404818
H -6.074394 2.092259 4.803107
H -5.207751 3.792742 6.212659
H -4.016077 4.328918 5.037402
H -3.824814 2.774657 5.831515
C -5.828205 0.762992 2.173343
C -6.945175 0.331028 3.123464
H -7.607125 1.155380 3.391956
H -6.573261 -0.133446 4.036915
H -7.557296 -0.416500 2.617431
C -6.393590 1.356897 0.890194
H -5.264109 -0.126456 1.891001
H -6.887416 2.316424 1.034337
H -7.147399 0.666681 0.505473
H -5.633987 1.470964 0.122319
C 0.809791 -4.293063 -5.451599
C 1.915326 -5.136579 -5.475996
C 0.554630 -3.465201 -4.379928
C 1.384121 -3.437269 -3.261379
C 2.488612 -4.278193 -3.296411
C 2.755981 -5.108209 -4.371508
F -0.022594 -4.260192 -6.484093
F -0.505223 -2.666733 -4.443836
F 3.324615 -4.303652 -2.264877
F 3.833666 -5.874083 -4.293595
C 2.127069 -6.010338 -6.685155
C 1.104615 -2.559983 -2.133205
C -0.106419 -2.300810 -1.563432
C -0.049458 -1.347597 -0.511281
C 1.231520 -0.900416 -0.287511
S 2.343100 -1.649479 -1.353372
H -1.020417 -2.792577 -1.861530
C 1.717448 0.100746 0.706682
H 1.048046 0.957750 0.753304
H 2.713561 0.461818 0.453241
H 1.758849 -0.327192 1.707985
C -1.212451 -0.899596 0.232262
F 2.265965 -5.268706 -7.785499
F 1.081031 -6.817512 -6.872272
F 3.205740 -6.777810 -6.586579
H -4.788118 -2.507050 0.113127
S -4.677550 -1.555433 -3.380262
C -1.658061 0.748055 -6.873819
C -3.099696 -1.488102 -6.206095
C -4.328245 -1.491276 -1.705019
C -2.059193 0.528891 -5.567355
C -5.306043 -2.029272 -0.716419
C -2.494080 -0.763084 -0.161942
C -3.091855 -0.941089 -1.473669
C -1.976206 -0.153836 -7.879339
C -2.785981 -0.592708 -5.186752
C -1.583469 0.007177 -9.324815
C -2.449375 -0.550170 -2.678608
C -3.170078 -0.830893 -3.802532
C -2.710581 -1.275278 -7.510349
F -0.973222 1.855280 -7.118927
F -3.045827 -2.176839 -8.422529
F -1.730487 1.450057 -4.665004
F -3.792127 -2.586439 -5.927716
F -2.666031 0.058268 -10.104250
F -0.882195 1.111880 -9.550137
F -0.842282 -1.024070 -9.731539
H -1.469997 -0.096354 -2.692577
H -5.969482 -2.766086 -1.167996
H -5.928057 -1.235020 -0.301142
O -1.066049 -0.569310 1.536088
O -3.274495 -0.366921 0.878443
B -2.323878 0.039917 1.933455
O -2.107802 1.514761 1.918571
C -3.041067 2.481123 2.075802
C -3.551649 3.010923 0.742230
C -2.911738 2.615936 -0.426830
C -4.618854 3.897463 0.640336
C -3.349939 3.072983 -1.661178
C -5.047716 4.370605 -0.588201
C -4.417544 3.951183 -1.749375
H -2.068428 1.943531 -0.381868
H -5.149365 4.218687 1.524983
H -2.854341 2.730199 -2.559008
H -5.883879 5.055944 -0.636532
H -4.756848 4.304572 -2.713927
C -2.435768 3.562542 2.976006
C -2.641467 4.926763 2.799845
C -1.596742 3.146777 4.010728
C -2.061641 5.849288 3.660368
C -1.025088 4.067312 4.872083
C -1.261436 5.424348 4.707663
H -3.228069 5.294453 1.971116
H -1.378314 2.096408 4.140204
H -2.227982 6.905940 3.496706
H -0.381262 3.721235 5.669972
H -0.809377 6.144049 5.377267

2-c-BP

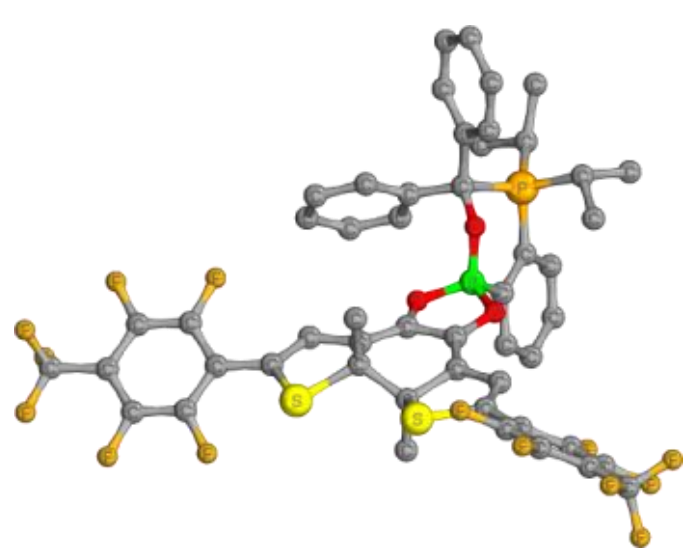

108
xyz, charge: 0, multiplicity: 1
C 14.638688 8.777429 4.151899
C 14.911354 7.427402 3.932036
C 14.289000 9.142492 5.457273
C 14.207123 8.206664 6.490750
C 14.479521 6.876718 6.232764
C 14.831857 6.489304 4.946400
H 15.192660 7.102714 2.938116

H	13.940182	8.500824	7.498258
H	14.419840	6.147459	7.029368
H	15.046959	5.449205	4.738237
P	13.905469	10.873002	5.751684
C	14.201638	11.283459	7.532092
C	13.629945	12.639183	7.950992
H	14.268053	13.459809	7.637591
H	13.567166	12.668813	9.039341
H	12.632852	12.838735	7.562627
C	15.643629	11.135325	8.003863
H	13.613885	10.512402	8.039933
H	16.040596	10.136326	7.832182
H	15.677344	11.317693	9.078867
H	16.309420	11.851701	7.525924
C	12.075433	11.072742	5.506220
C	11.557084	10.471751	4.200039
H	11.782586	9.408007	4.130192
H	11.949972	10.951126	3.312105
H	10.471317	10.578431	4.187316
C	11.326717	10.423496	6.674964
H	11.888076	12.150187	5.506752
H	11.519825	10.885992	7.641001
H	11.545799	9.357056	6.748087
H	10.256002	10.514462	6.491750
C	22.639152	9.995759	-2.091162
C	23.295919	10.908115	-1.277339
C	21.282878	9.760831	-1.948037
C	20.499041	10.414124	-1.001316
C	21.172287	11.338754	-0.200077
C	22.522694	11.572423	-0.331001
F	23.278992	9.305111	-3.022667
F	20.728350	8.857202	-2.747275
F	20.511397	12.047540	0.711947
F	23.085174	12.472006	0.468484
C	24.766427	11.218572	-1.355295
C	19.072581	10.164137	-0.868531
C	18.359177	10.211858	0.281274
C	16.973989	9.953210	0.107811
C	16.533653	10.043958	-1.344346
S	18.090984	9.749860	-2.285174
H	18.787007	10.381272	1.258598
C	16.077551	11.479554	-1.626154
H	15.173977	11.714976	-1.062220
H	15.871544	11.640327	-2.682428
H	16.860902	12.172445	-1.320970
C	16.027054	9.739190	1.043714
F	25.396825	10.513257	-2.286470
F	25.366753	10.962440	-0.189825
F	24.965571	12.509372	-1.632192
H	16.448990	7.358169	-0.584092
S	14.605359	9.211643	-3.233876
C	9.809273	7.396808	-3.790058
C	11.684461	9.215559	-4.575490
C	15.449115	8.990917	-1.610710
C	10.897380	7.658524	-2.986850
C	15.963649	7.549142	-1.542189
C	14.660102	9.428932	0.681159
C	14.323536	9.151681	-0.595791
C	9.626850	8.036383	-5.011733
C	11.874439	8.586916	-3.349110
C	8.409395	7.696216	-5.829198
C	13.044410	8.883124	-1.155163
C	13.029909	8.862759	-2.507998
C	10.597472	8.953216	-5.390659
F	8.925392	6.495743	-3.378185
F	10.519554	9.621358	-6.531788
F	11.005904	6.978316	-1.850792
F	12.563218	10.119720	-4.991491
F	8.395605	6.399067	-6.143694
F	7.292313	7.954350	-5.144175
F	8.340789	8.378698	-6.965853
H	12.164591	8.757094	-0.541712
H	16.679771	7.335879	-2.333151
H	15.125117	6.861250	-1.645016
O	16.134402	9.784903	2.360568
O	13.903449	9.426126	1.771071
B	14.759642	9.814892	2.912748
O	14.345517	11.183180	3.267977
C	14.824319	11.822416	4.388715
C	16.348875	11.794241	4.429801
C	14.303587	13.251194	4.432170
C	17.000608	12.674005	3.565403
C	18.372720	12.629182	3.407472
C	19.126160	11.700231	4.112260
C	18.488777	10.815702	4.963091
C	17.108938	10.855626	5.109950
H	16.645057	10.109334	5.736709
H	16.429184	13.392635	2.992949
H	18.855100	13.315322	2.724844
H	20.200355	11.663408	3.987965
H	19.059279	10.072474	5.503928
C	13.262667	13.655575	3.604653
C	12.784633	14.957624	3.664995
C	13.343909	15.871266	4.543028
C	14.399028	15.480105	5.355000
C	14.876543	14.182103	5.294754
H	15.727286	13.899877	5.903435
H	12.839092	12.963741	2.891367
H	11.978736	15.259289	3.009162
H	12.973533	16.886901	4.582993
H	14.862853	16.191809	6.025104

2-o-CO_2

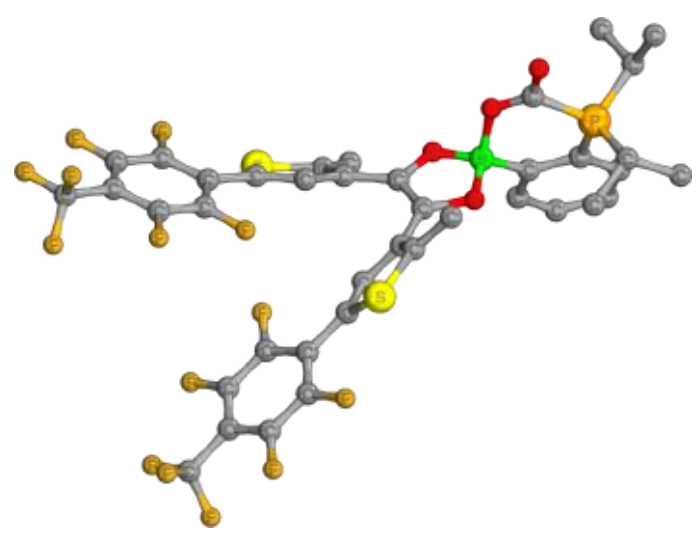

87
xyz, charge: 0, multiplicity: 1

C	9.442584	-1.545102	1.294169
C	10.775830	-1.830753	1.037551
C	9.026460	-0.247733	1.534013
C	9.893631	0.841584	1.531538
C	11.230703	0.537099	1.266555
C	11.653955	-0.752992	1.030215
F	8.517965	-2.492840	1.327921
F	7.732145	-0.055290	1.774816
F	12.149001	1.496527	1.226984
F	12.941881	-0.952258	0.781093
C	11.318357	-3.209270	0.767940
F	11.885385	-3.267628	-0.438705
F	10.382887	-4.150469	0.811291
F	12.250737	-3.534745	1.666106
C	9.438584	2.203149	1.770774
C	10.164858	3.264264	2.237994
C	9.431916	4.477420	2.307839
C	8.128479	4.317295	1.902195
S	7.813802	2.700734	1.444966
H	11.191361	3.195622	2.559608
C	7.044049	5.338430	1.819878
H	7.376482	6.208738	1.253772
H	6.755825	5.686203	2.810995
H	6.156467	4.943113	1.327734
H	14.541113	7.044026	3.517713
S	14.678792	4.715277	1.173621
C	12.221241	4.993327	0.569743
C	14.173307	1.509571	-4.881286
C	13.372493	4.452866	0.068748
C	14.946732	2.974953	-3.019526
C	14.772283	3.682147	-1.850579
C	12.378473	5.595531	1.845425
C	12.688355	2.277290	-2.938927
C	14.226598	6.001972	3.595354

C	13.908954	2.253879	-3.598804
C	13.669759	5.509876	2.303367
C	12.522192	2.987487	-1.761885
C	13.552870	3.717696	-1.174675
F	11.635182	1.618194	-3.398196
F	11.320953	2.943575	-1.196447
F	13.107294	0.859409	-5.331665
F	14.561237	2.350233	-5.843355
F	15.146573	0.612174	-4.715623
F	15.814174	4.350333	-1.363900
F	16.140131	3.001179	-3.597886
H	15.092303	5.419930	3.909325
H	11.274939	4.988641	0.052508
H	13.477410	5.939381	4.383103
C	10.020705	5.730284	2.743841
C	11.272471	6.190775	2.577163
O	9.262824	6.630479	3.426808
O	11.426188	7.402009	3.180117
B	10.173140	7.648100	3.880150
P	10.969262	9.957919	5.880900
C	12.683252	10.564354	5.679683
C	10.180197	10.720058	7.350604
C	13.338056	9.893897	4.475529
H	14.363830	10.252294	4.382998
H	12.815683	10.126493	3.547972
H	13.373061	8.810387	4.569175
C	12.723810	12.086035	5.572517
H	13.193805	10.242881	6.592930
H	12.267930	12.588391	6.426583
H	12.231760	12.434275	4.663992
H	13.762022	12.415244	5.523283
C	8.830607	10.050642	7.606101
H	8.190311	10.052753	6.724449
H	8.305859	10.587202	8.396687
H	8.953968	9.019525	7.937432
C	11.074190	10.707865	8.587212
H	10.001452	11.759820	7.054570
H	11.302511	9.694368	8.909149
H	10.555812	11.211555	9.403611
H	12.015189	11.234957	8.430453
C	9.954906	10.258281	4.447930
C	9.625545	9.153160	3.657360
C	9.489150	11.546960	4.169077
C	8.680049	11.754598	3.070642
C	8.336478	10.673633	2.265917
C	8.799981	9.403284	2.557915
H	8.512616	8.576884	1.921493
H	9.747400	12.385290	4.805562
H	8.313984	12.746802	2.844004
H	7.696958	10.828209	1.406389
C	11.059077	8.111186	6.197351
O	10.442107	7.354328	5.387747
O	11.663120	7.818530	7.196704

2-c-CO_2

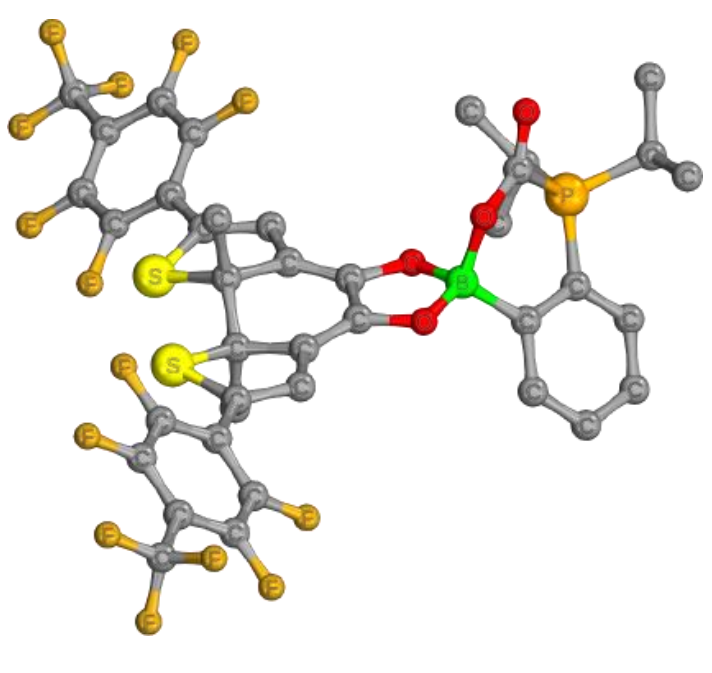

87
xyz, charge: 0, multiplicity: 1

C	13.437548	7.712097	3.426284
C	12.949047	6.439085	3.137764
C	13.697413	8.000942	4.769095
C	13.501676	7.065482	5.784653
C	13.021230	5.810348	5.463875
C	12.741211	5.503330	4.137716
H	12.724745	6.179944	2.111487
H	13.713060	7.306102	6.819899
H	12.861110	5.075980	6.241530
H	12.357089	4.523600	3.885092
P	14.289145	9.649461	5.119834
C	16.130049	9.740750	5.300932
C	16.709223	10.927031	4.533118
H	16.587599	10.797613	3.458365
H	17.777435	10.995660	4.739992
H	16.256149	11.876723	4.813268
C	16.806819	8.423999	4.932957
H	16.272483	9.912383	6.373187
H	16.669518	8.179051	3.882603
H	16.440463	7.587107	5.525729
H	17.876614	8.519490	5.122831
C	13.521349	10.256631	6.670845
C	14.066310	11.609752	7.124447
H	15.150834	11.617005	7.232378
H	13.647717	11.845092	8.103209
H	13.785759	12.404565	6.438522
C	11.998987	10.261719	6.554175
H	13.817162	9.503114	7.411099
H	11.565128	10.504304	7.524409
H	11.598744	9.296489	6.246301
H	11.659194	11.016727	5.846292
C	22.023086	10.748177	0.676246
C	22.973725	10.097784	-0.103286
C	20.675055	10.503658	0.533858
C	20.186816	9.583651	-0.394941
C	21.146253	8.943966	-1.172602
C	22.502194	9.188742	-1.039661
F	22.407698	11.640807	1.580273
F	19.837408	11.191662	1.303606
F	20.767263	8.047068	-2.074386
F	23.320435	8.512072	-1.830133
C	24.428880	10.418503	0.117019
C	18.762249	9.324739	-0.553805
C	17.838464	9.334908	0.434565
C	16.513772	9.077746	-0.013738
C	16.371054	9.212717	-1.522750
S	18.086708	8.960656	-2.146221
H	18.078771	9.472536	1.478379
C	15.960891	10.654473	-1.842347
H	14.960889	10.865812	-1.461826
H	15.965564	10.848898	-2.912774
H	16.658735	11.343094	-1.367485
C	15.397765	8.831172	0.697408
F	24.784750	10.150151	1.375766
F	24.666281	11.713286	-0.100402
F	25.237124	9.728476	-0.677581
H	15.093866	6.031756	-2.197555
S	14.881649	8.426451	-3.789001
C	10.349584	6.587901	-5.385030
C	12.278487	8.495857	-5.681179
C	15.378354	8.161522	-2.035553
C	11.246683	6.827913	-4.367006
C	15.885090	6.720789	-1.904502
C	14.140960	8.516810	0.057483
C	14.068949	8.281463	-1.266196
C	10.391805	7.295455	-6.581677
C	12.241152	7.799007	-4.479024
C	9.373879	6.974055	-7.644734
C	12.932095	8.024737	-2.083381
C	13.197497	8.051075	-3.408493
C	11.384158	8.257066	-6.710221
F	9.434635	5.643350	-5.207581
F	11.511004	8.988822	-7.806300
F	11.153540	6.086876	-3.268882
F	13.192562	9.440913	-5.861938
F	9.507632	7.722346	-8.732686
F	9.473777	5.698626	-8.024471
F	8.135354	7.160961	-7.183669
H	11.945294	7.877674	-1.669925
H	16.165226	6.501376	-0.873392

H 16.749964 6.535803 -2.538134
O 15.223500 8.847378 2.020507
O 13.172741 8.460890 0.974435
B 13.763299 8.770441 2.260285
C 13.653096 10.751441 3.723110
O 13.566233 11.919272 3.982482
O 13.308157 10.166020 2.641121

PhCHO

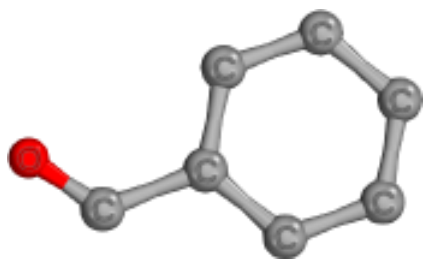

14
xyz, charge: 0, multiplicity: 1
C -5.252695 2.869016 -0.000001
C -5.155392 1.485601 -0.000000
C -4.104341 3.653437 -0.000000
C -2.857535 3.056361 0.000002
C -2.754335 1.666691 0.000003
C -3.905482 0.887065 0.000002
H -6.226765 3.340442 -0.000003
H -6.050037 0.877685 -0.000001
H -4.187465 4.732050 -0.000001
H -1.956998 3.656425 0.000002
H -3.821891 -0.193916 0.000003
C -1.435419 1.006080 0.000005
O -0.379933 1.581260 -0.000005
H -1.482952 -0.101718 -0.000006

PhNCS

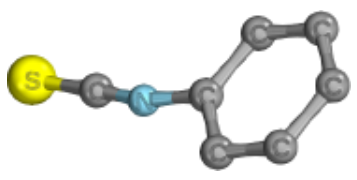

14
xyz, charge: 0, multiplicity: 1
C -2.762760 0.502345 0.163183
C -3.369370 0.393145 1.412265
C -4.720646 0.663960 1.540879
C -5.470535 1.042014 0.436531
C -4.860795 1.149259 -0.805648
C -3.510947 0.881954 -0.950789
H -3.030759 0.963939 -1.916470
H -2.778032 0.097915 2.268120
H -5.189790 0.578549 2.511735
H -6.525968 1.252365 0.542938
H -5.439572 1.443536 -1.670835
N -1.429483 0.234996 0.035666
C -0.330897 0.068734 -0.353894
S 1.152464 -0.169070 -0.822443

Propylene Oxide

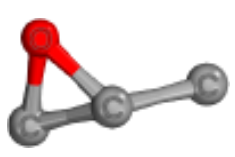

10
xyz, charge: 0, multiplicity: 1
C -3.352440 1.098367 0.103558
O -2.549072 1.616067 -0.934614
C -1.899883 0.955125 0.130400
H -3.969576 0.238740 -0.150722
H -3.840904 1.828917 0.744284
C -1.221152 -0.345577 -0.182769
H -1.343002 1.616526 0.794794
H -1.844560 -0.977634 -0.815203
H -1.003439 -0.892727 0.734990
H -0.276993 -0.178963 -0.702297

Benzophenone

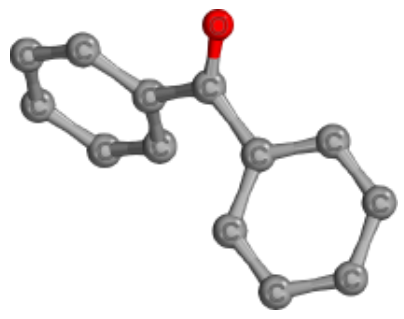

24
xyz, charge: 0, multiplicity: 1
C -4.937121 2.432255 -0.030836
C -4.962404 1.103199 -0.424224
C -3.746469 3.006722 0.394221
C -2.585135 2.255320 0.422358
C -2.607145 0.911256 0.054431
C -3.804284 0.342669 -0.374068
H -5.843266 3.023026 -0.060393
H -5.884482 0.656982 -0.772101
H -3.723803 4.045105 0.697055
H -1.652234 2.704506 0.735876
H -3.830676 -0.691056 -0.693551
C -1.325415 0.149381 0.054436
O -0.277353 0.714775 -0.157446
C -1.351100 -1.317898 0.318637
C -0.363429 -2.117036 -0.254266
C -0.328663 -3.476258 0.001512
C -1.261666 -4.047746 0.856635
C -2.231085 -3.255529 1.451958
C -2.281572 -1.897389 1.177981
H -3.034861 -1.284428 1.655672
H -1.228598 -5.109564 1.062611
H -2.949034 -3.694292 2.131950
H 0.372791 -1.665552 -0.905810
H 0.431555 -4.092116 -0.460279

tBuNC

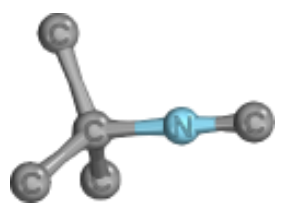

15
xyz, charge: 0, multiplicity: 1
N -0.626741 -0.029445 -2.553429
C 0.169455 0.780226 -2.289520
C -1.607596 -1.015582 -2.882844
C -1.453329 -2.189508 -1.916498
H -2.191686 -2.957997 -2.145629
H -0.461941 -2.634788 -1.995823
H -1.602321 -1.870325 -0.885311
C -2.991687 -0.383060 -2.742386
H -3.103968 0.467614 -3.414070
H -3.760995 -1.115245 -2.988052
H -3.163352 -0.037483 -1.723293
C -1.368538 -1.469990 -4.322331
H -0.375145 -1.902951 -4.436833
H -2.103883 -2.225421 -4.599543
H -1.458745 -0.635285 -5.016972

CO_2

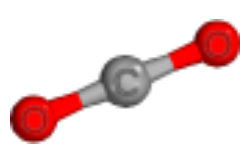

3
xyz, charge: 0, multiplicity: 1
O 0.000000 -0.000000 -0.155328
C -0.000000 0.000000 1.000000
O 0.000000 -0.000000 2.155328

2-o-AuCl

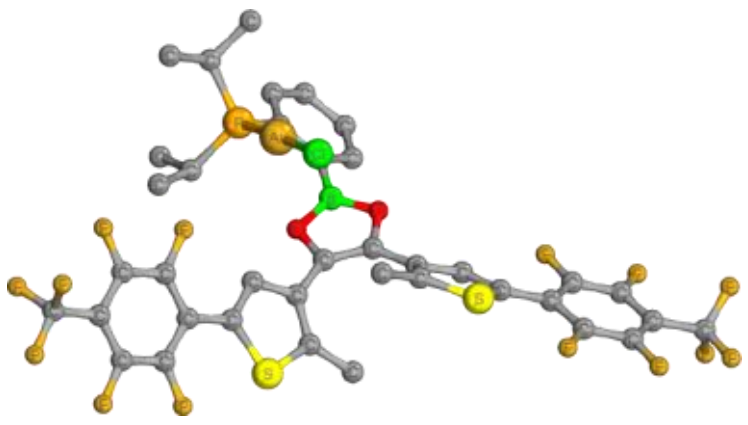

86
xyz, charge: 0, multiplicity: 1
Au -0.024997 2.308503 0.044809
Cl 1.414775 1.449088 -1.522813
S -1.205707 -1.908919 -5.548383
S -0.514860 -4.174050 0.026268
P -1.462163 3.094774 1.591366
F -5.142801 0.370398 -6.211656
F -6.557873 0.196142 -8.420939
F -3.622181 -3.184029 -10.052517
F -2.218594 -3.025676 -7.855488
F 2.029467 -0.824494 1.916763
F 3.973271 -1.279519 3.623388
F 5.858157 -3.354988 4.126121
F 2.934539 -5.929681 3.591718
F 0.984524 -5.481766 1.912403
F 4.867631 -5.064085 5.053211
F 4.472909 -3.049566 5.794091
O -3.025822 1.035036 -2.051170
O -2.392375 0.357021 0.027226
C -3.603388 -1.333180 -6.902544
C -4.743688 -0.531794 -7.130385
C -5.492582 -0.613260 -8.297364
C -5.164897 -1.498393 -9.334165
C -4.034153 -2.298159 -9.133339
C -3.288790 -2.209658 -7.958845
C -2.821589 -1.249733 -5.678225
C -1.013623 -1.377889 -3.922055
C -2.169694 -0.740885 -3.476036
C -3.174157 -0.656862 -4.477627
H -4.130734 -0.176810 -4.301709
C -6.032526 -1.527236 -10.572194
F -5.616613 -2.423908 -11.490102
F -6.055948 -0.309079 -11.179120
F -7.319460 -1.840566 -10.256052
C 0.282221 -1.592540 -3.216911
H 1.113024 -1.673833 -3.930200
H 0.270106 -2.512141 -2.610401
H 0.490983 -0.750300 -2.539869
C -2.363558 -0.188347 -2.151369
C -2.008109 -0.606186 -0.906515
C -3.659021 2.658880 -0.128939
C -3.125439 3.461737 0.909268
C -3.863741 4.553543 1.387299
H -3.466289 5.178836 2.186289
C -5.108570 4.874195 0.844153
H -5.663250 5.730275 1.231058
C -5.628815 4.107583 -0.198295
H -6.593023 4.361348 -0.640902
C -4.907047 3.013812 -0.673846
H -5.315141 2.413328 -1.488737
C -1.315339 -1.774099 -0.407376
C -0.274614 -1.669706 0.552160
H 0.058701 -0.711073 0.933565
C 0.284512 -2.884858 0.905116
C -1.564612 -3.084469 -0.796892
C -0.861990 4.684589 2.336584
H -1.627817 5.031754 3.049112
C 0.451326 4.461909 3.098575
H 1.205307 3.984033 2.455629
H 0.317410 3.838956 3.991453
H 0.852892 5.432387 3.425834
C 1.380974 -3.131389 1.829859
C 2.208462 -2.096203 2.318650
C 3.243678 -2.330629 3.215146
C 3.544123 -3.614910 3.690390
C 2.744506 -4.657819 3.209758
C 1.705576 -4.412989 2.313525
C -2.580020 -3.587545 -1.767872
H -2.182337 -3.608334 -2.795407
H -3.452790 -2.919957 -1.776209
H -2.914165 -4.602849 -1.515913
C -1.836209 1.869740 2.946974
H -2.449423 1.137454 2.396121
C -2.676355 2.472484 4.076404
H -2.105659 3.216197 4.652003
H -2.979572 1.680134 4.777056
H -3.591257 2.951439 3.701252
C -0.679086 5.728354 1.225620
H 0.094631 5.401002 0.515750
H -0.356194 6.683584 1.665758
H -1.601576 5.907879 0.658324
C 4.686075 -3.784296 4.667200
C -0.591772 1.137929 3.462464
H 0.054759 0.806765 2.639802
H -0.898630 0.250906 4.036133
H 0.012427 1.768914 4.127457
B -2.984780 1.383102 -0.706636

2-c-AuCl

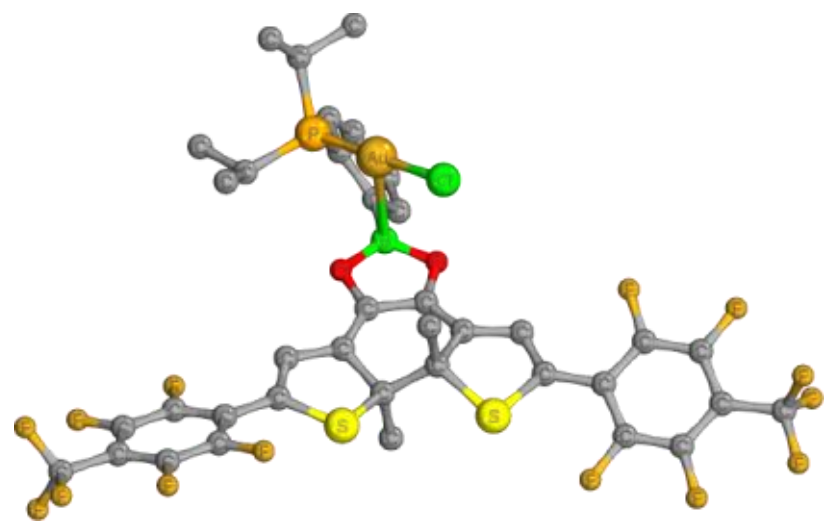

86
xyz, charge: 0, multiplicity: 1
Au -0.858585 3.139712 -0.744355
Cl -0.250142 2.593340 -2.883269
S -1.719228 -2.818028 -4.237695
S 0.218844 -3.541440 -1.211816
P -1.498224 3.715171 1.338174
F -3.578004 0.687814 -6.509897
F -4.414123 0.500070 -9.001316
F -3.783076 -4.221663 -8.869715
F -2.979021 -4.046683 -6.388856
F 0.260743 -2.831265 3.337181
F 1.962519 -4.018343 4.951307
F 4.726036 -4.671591 4.949767
F 4.292974 -5.814408 1.202795
F 2.629356 -4.614632 -0.410607
F 5.118135 -6.328298 3.584328
F 3.448201 -6.449468 4.983990
O -3.477310 1.066512 -1.378820
O -2.295431 0.572993 0.552496
C -3.241702 -1.676161 -6.307054
C -3.639254 -0.542044 -7.050815
C -4.071402 -0.633249 -8.367581
C -4.144416 -1.860318 -9.044039
C -3.749227 -2.995744 -8.327044
C -3.314264 -2.897242 -7.006072
C -2.777929 -1.579901 -4.931438
C -1.324557 -1.728238 -2.781256
C -2.468589 -0.703964 -2.784561
C -3.084480 -0.567505 -4.043308
H -3.767561 0.244294 -4.274143
C -4.633900 -1.874388 -10.475698
F -4.677481 -3.112402 -11.009178
F -3.828984 -1.119219 -11.271485
F -5.888103 -1.352496 -10.563566
C -0.010191 -0.985025 -3.097795
H -0.132775 -0.410937 -4.023471
H 0.821802 -1.688776 -3.215677
H 0.231095 -0.271506 -2.299075
C -2.673055 -0.032826 -1.612120
C -1.990033 -0.367343 -0.408941
C -3.956173 2.577733 0.707760
C -3.317302 3.584507 1.468156

C -4.079283 4.469374 2.239575
H -3.593901 5.251110 2.824689
C -5.473053 4.379719 2.251697
H -6.055661 5.079160 2.852921
C -6.114522 3.408670 1.482871
H -7.203618 3.347449 1.473948
C -5.358975 2.515699 0.720514
H -5.864898 1.754711 0.123462
C -1.205234 -1.479116 -0.281576
C -0.374794 -1.904561 0.770663
H -0.331293 -1.387830 1.724163
C 0.422648 -2.990968 0.461689
C -1.295760 -2.485432 -1.435785
C -1.074345 5.473294 1.741535
H -1.504474 5.693671 2.732634
C 0.447637 5.657415 1.809768
H 0.925023 5.340664 0.870667
H 0.902759 5.092615 2.633267
H 0.681928 6.720917 1.964808
C 1.355987 -3.646022 1.361910
C 1.259052 -3.538293 2.769044
C 2.146570 -4.169939 3.628500
C 3.205563 -4.960967 3.157564
C 3.317686 -5.088483 1.768014
C 2.422378 -4.451095 0.910009
C -2.532475 -3.379420 -1.198741
H -2.586870 -4.189290 -1.935507
H -3.453052 -2.782434 -1.268845
H -2.473041 -3.815981 -0.193803
C -0.878549 2.600197 2.697079
H -1.533014 1.723546 2.566655
C -1.113324 3.182892 4.093675
H -0.476655 4.058896 4.284091
H -0.868157 2.428094 4.855584
H -2.161745 3.476227 4.246135
C -1.695261 6.405294 0.691821
H -1.271161 6.202790 -0.302479
H -1.474125 7.451508 0.950363
H -2.785031 6.293802 0.621372
C 4.133322 -5.609563 4.160191
C 0.564160 2.136596 2.466412
H 0.688433 1.711326 1.461136
H 0.823454 1.362046 3.203662
H 1.288120 2.954977 2.577513
B -3.177747 1.476645 -0.076178

2-o-Rh(CNCy)$_2$Cl_cis

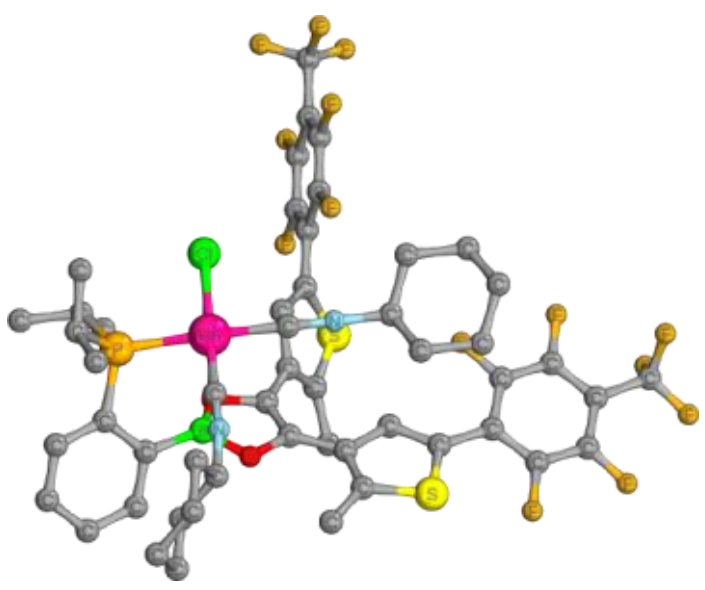

124
xyz, charge: 0, multiplicity: 1
C -0.757488 -4.384261 1.164830
C -1.160620 -5.718948 1.246102
C 0.554480 -4.061271 1.546999
C 1.411967 -5.067200 1.985723
C 0.993452 -6.387417 2.051211
C -0.300812 -6.714492 1.682602
H -2.169035 -5.990828 0.957395
H 2.428073 -4.828312 2.272661
H 1.677560 -7.154656 2.389645
H -0.639796 -7.741181 1.731094
P 1.101055 -2.317365 1.419427
C 2.924316 -2.377957 1.073539
C 3.480395 -0.955449 1.114784
H 4.542411 -0.963513 0.862769
H 3.382227 -0.494662 2.097246
H 2.967605 -0.311233 0.399272
C 3.227746 -3.034917 -0.266408
H 3.407180 -2.963581 1.860926
H 4.307905 -3.127303 -0.396136
H 2.845468 -2.435252 -1.091927
H 2.798878 -4.034723 -0.345048
C 1.014585 -1.670636 3.163011
C -0.405144 -1.730649 3.711146
H -0.769315 -2.755825 3.791487
H -1.094438 -1.160622 3.096871
H -0.419203 -1.298863 4.713786
C 1.986836 -2.350854 4.120343
H 1.278668 -0.615273 3.054544
H 3.031027 -2.243786 3.827015
H 1.768208 -3.414244 4.231089
H 1.889761 -1.901197 5.110304
C -9.270758 -0.417002 -4.734380
C -9.428109 0.867396 -4.234526
C -8.228569 -1.223304 -4.310628
C -7.284564 -0.814892 -3.372325
C -7.456703 0.481944 -2.884119
C -8.489857 1.293101 -3.301467
F -10.106024 -0.928217 -5.624147
F -8.145399 -2.442023 -4.836781
F -6.600561 0.992504 -2.002868
F -8.568826 2.513986 -2.792689
C -10.529861 1.814153 -4.637578
C -6.199359 -1.682360 -2.931107
C -5.495121 -1.618688 -1.760206
C -4.488011 -2.613934 -1.630462
C -4.423248 -3.422631 -2.743547
S -5.607110 -2.985204 -3.900047
H -5.678687 -0.883438 -0.994825
C -3.514946 -4.566012 -3.058018
H -3.581034 -5.347007 -2.302111
H -2.476956 -4.237887 -3.099161
H -3.764584 -5.013128 -4.019666
C -3.633124 -2.722658 -0.465351
F -11.250463 2.176359 -3.575716
F -11.365325 1.286067 -5.522800
F -10.021811 2.922584 -5.178939
H -7.320555 -2.157231 0.466855
S -6.388727 0.353896 2.184311
C -4.992599 4.281808 4.145246
C -3.887064 3.394723 1.803140
C -5.774891 -1.106898 1.532897
C -5.202607 2.986548 3.725427
C -6.665537 -2.283829 1.331420
C -3.607729 -2.052947 0.703748
C -4.434861 -0.995277 1.261989
C -4.216135 5.168352 3.406728
C -4.649462 2.501394 2.544752
C -4.027193 6.566105 3.938168
C -3.900930 0.279021 1.601785
C -4.841823 1.122443 2.109544
C -3.666284 4.697103 2.222221
F -5.541398 4.672788 5.287938
F -2.926790 5.468986 1.441453
F -5.946350 2.186353 4.480532
F -3.359484 3.016654 0.647528
F -3.451492 6.539477 5.141574
F -3.269603 7.322382 3.153946
F -5.204397 7.182216 4.068238
H -2.853763 0.531621 1.478467
H -7.298580 -2.461557 2.200601
H -6.068771 -3.178142 1.159261
O -2.591924 -3.607573 -0.490693
O -2.538115 -2.467360 1.438532
B -1.849231 -3.374239 0.658542
Rh -0.138404 -1.244718 -0.227289
Cl -0.281986 0.708723 1.111610
C 0.125514 -2.671270 -1.398072
C -1.348855 -0.383151 -1.483711
N -2.129876 0.126280 -2.170734
N 0.300503 -3.529452 -2.169062
C 0.558754 -4.698616 -2.924583

H -0.174695 -4.729877 -3.739291
C -3.092404 0.773531 -2.984352
H -4.053860 0.660497 -2.476299
C -3.178002 0.092129 -4.348669
H -3.424382 -0.963440 -4.217004
H -2.194945 0.137254 -4.827866
C -2.782697 2.263709 -3.114008
H -2.746562 2.718509 -2.123219
H -1.790141 2.384608 -3.558269
C -4.217458 0.781986 -5.224024
H -4.240173 0.307743 -6.207114
H -5.211927 0.631646 -4.791236
C -3.835921 2.939490 -3.985526
H -3.596374 3.998940 -4.092122
H -4.807035 2.894970 -3.481103
C -3.941110 2.275921 -5.354679
H -4.724636 2.753378 -5.946795
H -3.004984 2.425161 -5.902883
C 1.956883 -4.651974 -3.542064
H 2.692742 -4.542395 -2.740347
H 2.048854 -3.769869 -4.178841
C 0.371313 -5.940454 -2.052595
H -0.645144 -5.956657 -1.656258
H 1.037637 -5.869225 -1.186865
C 2.237069 -5.925156 -4.332280
H 3.247887 -5.885401 -4.742581
H 1.560225 -5.979464 -5.191807
C 0.668291 -7.207862 -2.844900
H 0.565516 -8.076368 -2.191489
H -0.079828 -7.329534 -3.635969
C 2.059709 -7.167890 -3.466765
H 2.812578 -7.166281 -2.671650
H 2.236355 -8.068621 -4.058213

2-o-Rh(CNCy)$_2$Cl_trans

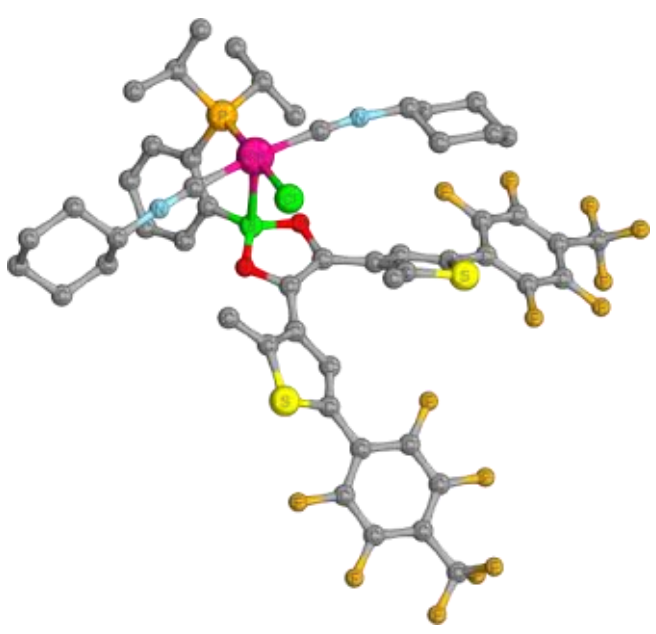

124
xyz, charge: 0, multiplicity: 1
Rh -1.938535 1.062840 3.945568
Cl -0.272391 1.065921 5.637585
S 0.097246 -3.603986 8.708558
S 3.370237 -2.993885 3.029809
P -3.531357 1.091703 2.372289
F 3.104820 -5.217120 5.962010
F 4.728782 -7.211802 6.523535
F 4.513204 -9.780967 7.648137
F 2.110000 -8.268667 10.303264
F 0.490792 -6.303755 9.718402
F 1.254511 -3.221222 -0.826607
F 2.224193 -4.301698 -2.982086
F 5.640207 -6.321652 -0.431106
F 4.639870 -5.230466 1.750970
F 5.703253 -8.454272 8.856709
F 4.173919 -9.654388 9.761625
O -2.267204 -2.002853 5.052466
O -1.405796 -1.885016 2.975686
N -3.993735 0.643601 6.223544
N 0.443897 1.272796 1.962641
C -4.928500 0.237798 7.206970
H -4.365833 0.072693 8.132516
C -5.967827 1.330075 7.455080
H -5.470779 2.248695 7.772741
H -6.478187 1.552217 6.512904
C -6.979653 0.870743 8.499159
H -6.476378 0.745121 9.463705
H -7.730720 1.649027 8.646567
C -7.642369 -0.442338 8.096409
H -8.242646 -0.283957 7.194387
H -8.334736 -0.768900 8.875018
C -6.602004 -1.524019 7.826903
H -7.084814 -2.444137 7.492537
H -6.083410 -1.772463 8.759121
C -5.586608 -1.075791 6.782633
H -4.818900 -1.835915 6.626808
H -6.076895 -0.927451 5.815199
C -3.254165 0.845506 5.354119
C -4.586757 -0.381527 2.585015
C -4.003269 -1.483828 3.223249
C -4.789092 -2.622015 3.417182
H -4.364821 -3.487001 3.912525
C -6.110792 -2.670310 3.000987
H -6.699046 -3.562952 3.170417
C -6.677455 -1.571010 2.375497
H -7.710437 -1.596218 2.053715
C -5.913821 -0.432990 2.166751
H -6.370086 0.423994 1.686958
C -0.475055 1.208512 2.665990
C 1.622775 1.189744 1.177403
H 1.800546 2.185218 0.755893
C 1.423810 0.192511 0.036716
H 1.164015 -0.778890 0.466195
H 0.581577 0.502662 -0.585572
C 2.694977 0.072489 -0.795501
H 2.895846 1.024738 -1.298355
H 2.541045 -0.663373 -1.586883
C 3.891657 -0.310698 0.067288
H 3.738589 -1.315629 0.472560
H 4.797666 -0.354681 -0.540729
C 4.077165 0.668465 1.221197
H 4.360881 1.650371 0.827025
H 4.899794 0.346853 1.862525
C 2.809159 0.798680 2.057259
H 2.578275 -0.155022 2.538627
H 2.936219 1.534007 2.853714
C -4.672193 2.556782 2.420879
H -5.426317 2.417458 1.640895
C -3.872938 3.820297 2.109563
H -4.523870 4.695645 2.144600
H -3.414271 3.797414 1.120475
H -3.077070 3.965153 2.841929
C -3.025332 1.017927 0.573323
H -2.268280 1.803200 0.475693
C -2.375433 -0.320529 0.238182
H -1.994430 -0.294306 -0.784840
H -3.098299 -1.135255 0.296214
H -1.546510 -0.568973 0.893922
C -0.984464 -2.461500 5.070168
C -0.458150 -2.361025 3.836383
C -5.392333 2.695693 3.755959
H -6.132833 3.495266 3.690717
H -4.698086 2.955490 4.553882
H -5.915928 1.783657 4.043935
C -4.155524 1.298762 -0.412072
H -3.778274 1.212400 -1.432847
H -4.577026 2.298296 -0.313973
H -4.964297 0.573304 -0.310885
C -0.449269 -3.053647 6.280126
C -0.705372 -2.622765 7.559552
C 0.809774 -4.597053 7.489627
C 0.412994 -4.181137 6.251508
H 0.703312 -4.669859 5.333334
C 0.854493 -2.677373 3.310123
C 2.046438 -2.390214 3.934560
C 2.347746 -3.572485 1.760466
C 1.037632 -3.327941 2.059006
H 0.210606 -3.620423 1.429557
C -1.538748 -1.473748 8.012305
H -1.305261 -0.584006 7.425647
H -1.364025 -1.243612 9.062671
H -2.600252 -1.694002 7.890101
C 1.716532 -5.690268 7.811050

C 2.831687 -5.968877 7.022429
C 3.687435 -7.008426 7.318773
C 3.490500 -7.826376 8.426533
C 2.390257 -7.550332 9.226731
C 1.531351 -6.509797 8.917912
C 2.289125 -1.645079 5.199136
H 1.476178 -0.942608 5.386509
H 3.218426 -1.077322 5.150471
H 2.359206 -2.318775 6.054979
C 2.897412 -4.189322 0.560856
C 2.327604 -3.995932 -0.693267
C 2.846411 -4.566253 -1.843004
C 3.978343 -5.368075 -1.795290
C 4.559057 -5.563983 -0.546939
C 4.034559 -4.994189 0.593231
C 4.472737 -8.940126 8.683431
C 4.607663 -6.027865 -2.995413
F 4.632670 -7.353037 -2.846033
F 5.866562 -5.615151 -3.153526
F 3.960859 -5.768815 -4.125344
B -2.516265 -1.564404 3.753429

2-c-Rh(CNCy)$_2$Cl_cis

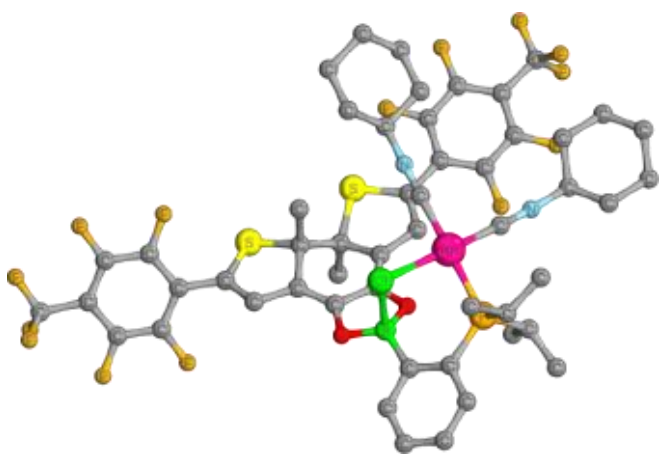

124
xyz, charge: 0, multiplicity: 1
C -1.071184 -4.172425 -0.412576
C -1.427360 -5.316099 -1.128993
C -1.741768 -3.909239 0.795301
C -2.746064 -4.779503 1.219266
C -3.085172 -5.906843 0.489441
C -2.416819 -6.179911 -0.690117
H -0.923517 -5.532874 -2.061678
H -3.290457 -4.584926 2.132971
H -3.869418 -6.562436 0.844475
H -2.670061 -7.055734 -1.273181
P -1.354621 -2.457800 1.858914
C -0.516917 -3.196846 3.349830
C 0.840006 -3.768469 2.951916
H 1.471468 -3.024844 2.469786
H 0.731737 -4.606017 2.261799
H 1.363387 -4.134489 3.837276
C -1.329591 -4.221327 4.130685
H -0.342245 -2.332852 3.999189
H -0.792597 -4.495340 5.040995
H -1.478687 -5.136554 3.558421
H -2.306838 -3.845861 4.436974
C -3.014152 -1.886684 2.462996
C -2.856587 -0.815311 3.537727
H -2.296330 -1.167008 4.405439
H -3.840223 -0.502805 3.892939
H -2.350092 0.065986 3.143110
C -3.836842 -1.369156 1.286931
H -3.536266 -2.737714 2.908323
H -3.362973 -0.500280 0.829275
H -4.829277 -1.070878 1.629997
H -3.964205 -2.121125 0.508949
C 3.225065 -4.160332 -9.098330
C 3.483180 -3.112764 -9.976058
C 3.127268 -3.955986 -7.737995
C 3.278752 -2.689499 -7.183304
C 3.534799 -1.646718 -8.062484
C 3.637520 -1.846031 -9.429085
F 3.076574 -5.393229 -9.563988
F 2.897376 -5.000809 -6.954970
F 3.662809 -0.412084 -7.598390
F 3.874560 -0.783030 -10.182271
C 3.575381 -3.418653 -11.449624
C 3.189588 -2.475876 -5.740628
C 2.180957 -2.875990 -4.940296
C 2.376958 -2.536711 -3.569529
C 3.477123 -1.491316 -3.390322
S 4.514579 -1.679215 -4.898884
H 1.308653 -3.406536 -5.294555
C 2.792399 -0.120232 -3.469017
H 2.106763 0.010250 -2.630292
H 2.211420 -0.057070 -4.388295
H 3.515620 0.692987 -3.467239
C 1.676838 -2.888608 -2.476942
F 3.816415 -2.342528 -12.187749
F 2.436862 -3.956692 -11.890463
F 4.554585 -4.292624 -11.688580
H 4.516041 -3.860500 -2.355207
S 5.306486 -0.296535 -1.597316
C 6.550397 2.514385 1.764237
C 5.082082 0.313319 2.477104
C 4.244929 -1.723924 -2.080394
C 6.067304 1.641114 0.806024
C 5.113069 -2.985141 -2.095910
C 2.105072 -2.494908 -1.158422
C 3.253657 -1.825772 -0.937848
C 6.300177 2.309605 3.112771
C 5.290963 0.520553 1.106306
C 6.760039 3.217991 4.219565
C 3.672306 -1.181522 0.248580
C 4.727279 -0.342242 0.085252
C 5.562740 1.175162 3.434648
F 7.241135 3.558420 1.333300
F 5.303154 0.896286 4.709284
F 6.341437 1.937419 -0.462477
F 4.403342 -0.745793 2.910705
F 7.456813 4.257489 3.779527
F 5.711270 3.701341 4.896005
F 7.527370 2.561554 5.090554
H 3.116087 -1.297438 1.164328
H 5.539443 -3.143445 -1.106220
H 5.931076 -2.908508 -2.809738
O 0.504493 -3.530678 -2.373471
O 1.178662 -2.860220 -0.278581
B 0.033679 -3.254240 -1.051253
Rh -0.167402 -0.659417 0.912094
Cl -0.977604 -1.345806 -1.274182
C 0.501094 -0.142481 2.564271
C 0.858727 0.788203 0.130374
N 1.563719 1.598707 -0.304933
N 0.989476 0.197953 3.566642
C 1.534517 0.703134 4.773187
H 2.469857 0.164012 4.958284
C 0.578457 0.445336 5.937905
C 1.849295 2.193218 4.631235
H 2.553685 2.344070 3.809756
H 0.929364 2.720418 4.360296
H -0.383499 0.916690 5.713367
H 0.393563 -0.627425 6.030493
C 2.484557 2.557255 -0.801000
C 1.898158 3.297721 -2.001569
C 2.882783 3.525800 0.313460
H 1.980091 4.013535 0.694219
H 3.318041 2.968193 1.146762
H 1.641459 2.582524 -2.785047
H 0.967454 3.786580 -1.697816
C 2.409779 2.752658 5.934036
H 3.381286 2.295256 6.140325
H 2.594859 3.822119 5.820282
C 1.147793 1.009632 7.234616
H 0.440055 0.840016 8.048118
H 2.058684 0.463592 7.501267
C 3.860493 4.573141 -0.208351
H 4.100160 5.277066 0.590924
H 4.802376 4.091943 -0.485824
C 2.891409 4.332917 -2.516335
H 3.781229 3.823444 -2.900840
H 2.455162 4.867337 -3.362017
C 3.296988 5.310018 -1.418428

H 2.424577 5.897886 -1.114441
C 1.465827 2.494962 7.102865
H 0.536138 3.053606 6.952255
H 1.904322 2.869435 8.029816
H 4.032157 6.021609 -1.798982
H 3.369126 1.998122 -1.127717

2-c-Rh(CNCy)$_2$Cl_trans

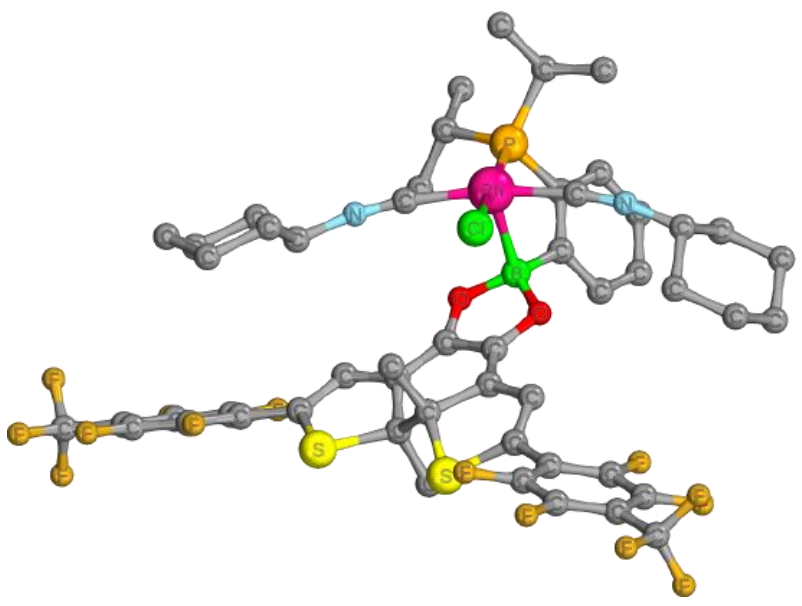

124
xyz, charge: 0, multiplicity: 1
C -2.223092 -6.406605 -0.999344
C -2.704699 -6.824945 -2.239100
C -2.657507 -7.090964 0.138869
C -3.546043 -8.156784 0.043474
C -4.019508 -8.550245 -1.198087
C -3.596417 -7.882531 -2.339057
H -2.386359 -6.313795 -3.139558
H -3.889143 -8.672625 0.932283
H -4.719897 -9.371448 -1.275559
H -3.966973 -8.186730 -3.309646
P -1.987979 -6.418589 1.683974
C -0.694692 -7.636897 2.254510
C 0.322429 -7.926011 1.155365
H 0.805240 -7.031468 0.775614
H -0.141377 -8.433476 0.308989
H 1.099941 -8.583875 1.548433
C -1.271265 -8.950305 2.776018
H -0.181304 -7.122204 3.073886
H -0.455826 -9.616198 3.063644
H -1.846255 -9.467538 2.006231
H -1.904542 -8.828118 3.653445
C -3.314871 -6.572869 2.969762
C -2.774388 -6.102827 4.318498
H -1.915420 -6.682687 4.657210
H -3.546673 -6.190758 5.084267
H -2.471652 -5.055144 4.273695
C -4.587239 -5.817622 2.606882
H -3.563215 -7.636223 3.038250
H -4.440085 -4.740247 2.663789
H -5.376129 -6.076590 3.315294
H -4.948261 -6.060322 1.607420
C -2.495245 1.970450 -5.416458
C -1.895480 3.220395 -5.306071
C -1.913829 0.839243 -4.887690
C -0.698079 0.888493 -4.207081
C -0.106181 2.141375 -4.098212
C -0.682983 3.281468 -4.634020
F -3.653176 1.844787 -6.053299
F -2.542581 -0.319953 -5.054576
F 1.036546 2.278526 -3.442815
F -0.036491 4.424190 -4.464061
C -2.594461 4.409541 -5.912382
C -0.087185 -0.315803 -3.654519
C -0.740452 -1.292869 -2.991767
C 0.103698 -2.359303 -2.574301
C 1.578325 -1.983516 -2.619568
S 1.635947 -0.629063 -3.870693
H -1.797954 -1.274665 -2.775762
C 1.950893 -1.375528 -1.262557
H 2.956719 -0.961106 -1.271785
H 1.881060 -2.124617 -0.473836
H 1.248785 -0.583220 -1.010988
C -0.217921 -3.562119 -2.072815
F -1.928647 5.544604 -5.742521
F -3.803384 4.572307 -5.370109
F -2.762072 4.239381 -7.225342
H 2.780630 -4.593549 -4.611444
S 4.228596 -2.953446 -2.719511
C 8.050176 -4.895117 -1.136385
C 5.970386 -6.509509 -1.923061
C 2.422702 -3.208476 -2.999411
C 6.800194 -4.363303 -1.399854
C 2.226992 -3.669948 -4.446687
C 0.790989 -4.528877 -1.728263
C 2.084046 -4.366531 -2.071718
C 8.283449 -6.257853 -1.255728
C 5.716335 -5.143682 -1.789965
C 9.605761 -6.924417 -0.978958
C 3.214406 -5.165730 -1.752009
C 4.399609 -4.573385 -2.034494
C 7.211163 -7.046996 -1.658763
F 8.997857 -4.054072 -0.752648
F 7.373655 -8.354964 -1.807897
F 6.640795 -3.054367 -1.233619
F 5.010367 -7.334105 -2.325335
F 10.545725 -6.068922 -0.597034
F 9.479413 -7.834645 -0.010260
F 10.056172 -7.552658 -2.065767
H 3.119759 -6.132023 -1.277591
H 2.580663 -2.928846 -5.160676
H 1.172829 -3.862023 -4.650372
O -1.417867 -4.062312 -1.753245
O 0.216080 -5.537961 -1.075320
B -1.181472 -5.201767 -0.911906
Rh -1.373834 -4.301537 1.200379
Cl -0.869059 -2.011022 0.916329
C 0.528313 -4.490553 1.619257
C -3.146983 -3.836006 0.539328
N -4.117029 -3.491337 0.012846
N 1.679324 -4.458486 1.728007
C 3.088289 -4.282501 1.678434
H 3.338385 -4.187463 0.616426
C 3.813006 -5.499857 2.245622
C 3.500622 -2.995342 2.387374
H 2.961870 -2.150588 1.954518
H 3.211133 -3.054803 3.440987
H 3.523679 -5.633327 3.292693
H 3.501518 -6.400050 1.710423
C -5.146367 -3.075255 -0.871661
C -4.551329 -2.182114 -1.961385
C -5.848544 -4.295224 -1.467572
H -5.101558 -4.920117 -1.966302
H -6.283820 -4.899951 -0.668972
H -4.072088 -1.316321 -1.499937
H -3.768316 -2.745912 -2.475023
C 5.008376 -2.806459 2.261224
H 5.267535 -2.652551 1.208788
H 5.307343 -1.897053 2.785141
C 5.321480 -5.304259 2.137812
H 5.835447 -6.161226 2.576977
H 5.605078 -5.285625 1.080438
C -6.919885 -3.856399 -2.460099
H -7.390394 -4.738254 -2.898855
H -7.712738 -3.318779 -1.928991
C -5.627084 -1.754358 -2.952150
H -6.355312 -1.107899 -2.451058
H -5.171145 -1.150641 -3.739170
C -6.341352 -2.961099 -3.550831
H -5.633583 -3.537090 -4.155611
C 5.770701 -4.009518 2.807233
H 5.606566 -4.084182 3.887340
H 6.844508 -3.869780 2.667633
H -7.132524 -2.633860 -4.228223
H -5.866583 -2.496873 -0.283774

7 References

1. R. Declercq, G. Bouhadir, D. Bourissou, et al., "Hydroboration of Carbon Dioxide Using Ambiphilic Phosphine–Borane Catalysts: On the Role of the Formaldehyde Adduct," *ACS Catalysis* 5 (2015): 2513-2520, https://doi.org/10.1021/acscatal.5b00189.

2. L. Stoess, J. A. Davis, L. Greb, "Photoswitching Lewis Acid Catalysis with Highly Fatigue Resistant Photochromic Boronates," *Journal of the American Chemical Society* 148 (2026): 7951-7960, https://doi.org/10.1021/jacs.5c22962.

3. F. Neese, F. Wennmohs, U. Becker, C. Riplinger, "The ORCA quantum chemistry program package," *The Journal of Chemical Physics* 152 (2020): 224108, https://doi.org/10.1063/5.0004608.

4. F. Weigend, "Accurate Coulomb-fitting basis sets for H to Rn," *Physical Chemistry Chemical Physics* 8 (2006): 1057-1065, https://doi.org/10.1039/b515623h.

5. F. Weigend, R. Ahlrichs, "Balanced basis sets of split valence, triple zeta valence and quadruple zeta valence quality for H to Rn: Design and assessment of accuracy," *Physical Chemistry Chemical Physics* 7 (2005): 3297-3305, https://doi.org/10.1039/b508541a.

6. A. V. Marenich, C. J. Cramer, D. G. Truhlar, "Universal Solvation Model Based on Solute Electron Density and on a Continuum Model of the Solvent Defined by the Bulk Dielectric Constant and Atomic Surface Tensions," *The Journal of Physical Chemistry B* 113 (2009): 6378-6396, https://doi.org/10.1021/jp810292n.

7. S. Grimme, J. G. Brandenburg, C. Bannwarth, A. Hansen, "Consistent structures and interactions by density functional theory with small atomic orbital basis sets," *The Journal of Chemical Physics* 143 (2015): 054107, https://doi.org/10.1063/1.4927476.

8. S. Grimme, "Supramolecular Binding Thermodynamics by Dispersion-Corrected Density Functional Theory," *Chemistry – A European Journal* 18 (2012): 9955-9964, https://doi.org/10.1002/chem.201200497.

9. S. Hirata, M. Head-Gordon, "Time-dependent density functional theory within the Tamm–Dancoff approximation," *Chemical Physics Letters* 314 (1999): 291-299, https://doi.org/10.1016/S0009-2614(99)01149-5.

10. Y.-S. Lin, G.-D. Li, S.-P. Mao, J.-D. Chai, "Long-Range Corrected Hybrid Density Functionals with Improved Dispersion Corrections," *Journal of Chemical Theory and Computation* 9 (2013): 263-272, https://doi.org/10.1021/ct300715s.

11. H. Böhrer, N. Trapp, D. Himmel, M. Schleep, I. Krossing, "From unsuccessful H_2-activation with FLPs containing $B(Ohfip)_3$ to a systematic evaluation of the Lewis acidity of 33 Lewis acids based on fluoride, chloride, hydride and methyl ion affinities," *Dalton Transactions* 44 (2015): 7489-7499, https://doi.org/10.1039/c4dt02822h.

12. P. Erdmann, L. Greb, "Multidimensional Lewis Acidity: A Consistent Data Set of Chloride, Hydride, Methide, Water and Ammonia Affinities for 183 p-Block Element Lewis Acids," *ChemPhysChem* 22 (2021): 935-943, https://doi.org/10.1002/cphc.202100150.

13. P. Erdmann, J. Leitner, J. Schwarz, L. Greb, "An Extensive Set of Accurate Fluoride Ion Affinities for p-Block Element Lewis Acids and Basic Design Principles for Strong Fluoride Ion Acceptors," *ChemPhysChem* 21 (2020): 987-994, https://doi.org/10.1002/cphc.202000244.

14. L. Krause, R. Herbst-Irmer, G. M. Sheldrick, D. Stalke, "Comparison of silver and molybdenum microfocus X-ray sources for single-crystal structure determination," *Journal of Applied Crystallography* 48 (2015): 3-10, https://doi.org/10.1107/S1600576714022985.

15. *V. B. S.* Bruker, Bruker AXS Inc., Madison, Wisconsin, USA.

16. G. M. Sheldrick, "Crystal structure refinement with SHELXL," *Acta Crystallographica Section C: Structural Chemistry* 71 (2015): 3-8, https://doi.org/10.1107/S2053229614024218.

17. G. M. Sheldrick, "SHELXT – Integrated space-group and crystal-structure determination," *Acta Crystallographica Section A: Foundations and Advances* 71 (2015): 3-8, https://doi.org/10.1107/S2053273314026370.

18. C. R. Groom, I. J. Bruno, M. P. Lightfoot, S. C. Ward, "The Cambridge Structural Database," *Acta Crystallographica Section B: Structural Science, Crystal Engineering and Materials* 72 (2016): 171-179, https://doi.org/10.1107/S2052520616003954.

19. V. F. D. Kratzert, FinalCif, v147, https://dkratzert.de/finalcif.html.
